# Generic revision of the ant subfamily Dorylinae (Hymenoptera, Formicidae)

**DOI:** 10.3897/zookeys.608.9427

**Published:** 2016-08-04

**Authors:** Marek L. Borowiec

**Affiliations:** 1 Department of Entomology and Nematology, One Shields Avenue, University of California at Davis, Davis, California, 95616, USA

**Keywords:** Taxonomy, systematics, morphology, dorylomorphs, doryline section, army ants

## Abstract

The generic classification of the ant subfamily Dorylinae is revised, with the aim of facilitating identification of easily-diagnosable monophyletic genera. The new classification is based on recent molecular phylogenetic evidence and a critical reappraisal of doryline morphology. New keys and diagnoses based on workers and males are provided, along with reviews of natural history and phylogenetic relationships, distribution maps, and a list of valid species for each lineage. Twenty-eight genera (27 extant and 1 extinct) are recognized within the subfamily, an increase from 20 in the previous classification scheme. Species classified in the polyphyletic *Cerapachys* and *Sphinctomyrmex* prior to this publication are here distributed among 9 and 3 different genera, respectively. *Amyrmex* and *Asphinctanilloides* are synonymized under *Leptanilloides* and the currently recognized subgenera are synonymized for *Dorylus*. No tribal classification is proposed for the subfamily, but several apparently monophyletic genus-groups are discussed. Valid generic names recognized here include: *Acanthostichus* (= *Ctenopyga*), *Aenictogiton*, *Aenictus* (= *Paraenictus*, *Typhlatta*), *Cerapachys* (= *Ceratopachys*), *Cheliomyrmex*, *Chrysapace* **gen. rev.**, *Cylindromyrmex* (= *Holcoponera*, *Hypocylindromyrmex*, *Metacylindromyrmex*), *Dorylus* (= *Alaopone* **syn. n.**, *Anomma* **syn. n.**, *Cosmaecetes*, *Dichthadia* **syn. n.**, *Rhogmus* **syn. n.**, *Shuckardia*, *Sphecomyrmex*, *Sphegomyrmex*, *Typhlopone* **syn. n.**), *Eburopone* **gen. n.**, *Eciton* (= *Camptognatha*, *Holopone*, *Mayromyrmex*), *Eusphinctus* **gen. rev.**, *Labidus* (= *Nycteresia*, *Pseudodichthadia*), *Leptanilloides* (= *Amyrmex* **syn. n.**, *Asphinctanilloides* **syn. n.**), *Lioponera* **gen. rev.** (= *Neophyracaces* **syn. n.**, *Phyracaces* **syn. n.**), *Lividopone*, *Neivamyrmex* (= *Acamatus*, *Woitkowskia*), *Neocerapachys* **gen. n.**, *Nomamyrmex*, *Ooceraea* **gen. rev.** (= *Cysias* **syn. n.**), *Parasyscia* **gen. rev.**, †*Procerapachys*, *Simopone*, *Sphinctomyrmex*, *Syscia* **gen. rev.**, *Tanipone*, *Vicinopone*, *Yunodorylus* **gen. rev.**, *Zasphinctus* **gen. rev.** (= *Aethiopopone* **syn. n.**, *Nothosphinctus* **syn. n.**).

## Preface

The ant subfamily Dorylinae is a monophyletic group of predatory ants, occurring throughout most of the tropical and subtropical regions of the world, with an appreciable number of species in warm temperate environments. The relatively few dorylines for which foraging biology is known usually prey on other ants or social insects, although notable exceptions occur and several of the charismatic ‘army ants’ evolved more generalized predatory habits. There are about 680 described species with an estimate of the total diversity being at least 1,000. The diversity of both habits and morphology within the subfamily is high and nesting can be subterranean or arboreal, with colony sizes ranging from a few dozen to millions of workers. These workers vary from having well-developed compound eyes to being entirely blind, having very short to very long slender appendages, and with the cuticle varying from coarsely sculptured to polished and shiny, with dull or conspicuous coloration.

Although numerous studies focusing on the biology of the few conspicuous species have been published, our overall knowledge of this clade is poor. A likely contributing factor is that many species are subterranean or occur at low abundances. It is also likely that comparative studies of doryline biology have been thwarted by poor taxonomic knowledge, lack of identification resources, and a classification that does not reflect evolutionary relationships.

The taxonomic limits of the Dorylinae have been in considerable flux since its establishment and, as currently circumscribed, the group has never received a focused treatment at the genus level. Until recently, our understanding of doryline morphology and phylogeny was insufficient to provide a stable classification based on easilydiagnosed monophyletic groupings. The aim of this study is to highlight the diversity of dorylines and provide a more natural genus-level classification, along with new identification resources. It is my hope that this effort will foster renewed interest in this highly diverse but neglected group of ants.

## Material and methods

The taxonomic decisions of the present work reflect the evidence from examination of morphological characters in most doryline species and from recently published molecular phylogenetic research (Brady et al. 2014, Borowiec in prep.). The rationale and taxonomic philosophy behind this reclassification is identical to that of Schmidt and Shattuck (2014), who recently produced a genus-level revision of the ant subfamily Ponerinae; firstly, classifications should reflect evolutionary relationships and non-monophyletic taxa should be avoided. Secondly, higher classifications are arbitrary and therefore their value lies in facilitating identification. Finally, taxonomic changes should be conservative to minimize confusion resulting from name changes. The generic definitions I propose here are designed to reflect this perspective. Because of poor resolution in the deeper parts of the doryline phylogeny, I follow Brady et al. (2014) and do not propose a tribal classification within the subfamily.

Specimens used in the course of this study come from the following institutions and individuals:

American Museum of Natural History, New York, USA.

Andreas Schulz personal collection, Leverkusen, Germany.

Bohart Museum of Entomology, Davis, California, USA.

California Academy of Sciences, San Francisco, California, USA.

John T. Longino personal collection, Salt Lake City, Utah, USA.

Los Angeles County Museum of Natural History, Los Angeles, California, USA.

Lund Zoological Museum, University of Lund, Lund, Sweden.

Marek L. Borowiec personal collection, Davis, California, USA.

Muséum d’Histoire Naturelle, Geneva, Switzerland.

Muséum National d’Histoire Naturelle, Paris, France.

Museum of Comparative Zoology, Cambridge, Massachusetts, USA.

Phil S. Ward personal collection, Davis, California, USA.

Senckenberg Forschungsinstitut und Naturmuseum, Frankfurt am Main, Germany.

Smithsonian National Museum of Natural History, Washington, D.C., USA.

The Natural History Museum, London, UK.

Color photographs were prepared using a Leica MZ 16 stereomicroscope with a JVC digital video camera. All images were processed using Syncroscopy Automontage and Zerene Systems Zerene Stacker software and cleaned and adjusted using Adobe Photoshop. Wing venation images were prepared in Adobe Illustrator, based on wing automontage photographs.

Representative specimens imaged for each genus were assigned unique CASENT identifiers and their collection data is available on AntWeb (www.antweb.org). Along with the name and the original description reference, species lists give the country of type locality or verbatim type locality if the country could not be determined with confidence.

Distribution maps were collated from the published records and material examined by the author. For each genus every valid species and subspecies is listed, along with the country of its type locality; where the country could not be confidently identified, the type locality is listed verbatim from the original description.

### Brief introduction to doryline ants

Below I provide an outline of doryline biology and diversity. More information on the natural history of each lineage can be found under individual genus accounts.

The 28 genera of the Dorylinae recognized here form a well-supported monophyletic group that is in turn a part of a more inclusive formicoid clade (Brady et al. 2006, Moreau et al. 2006). The formicoids hold almost 90% of all extant ant species. Within this group, the Dorylinae is sister to all other lineages. These include the subfamilies Aneuretinae, Dolichoderinae, Ectatomminae, Formicinae, Heteroponerinae, Myrmeciinae, Myrmicinae, and Pseudomyrmecinae (Ward 2014). Recent dating analyses place the origins of the crown group (that is all extant species including their common ancestor) dorylines at 70–100 million years and indicate that the subfamily has undergone a rapid radiation early in its history (Brady et al. 2014, Borowiec, in prep.). The rapid initial diversification can be perhaps attributed to specialization on other social insects as prey. This adaptation is currently not unique to this clade, but doryline ants were likely the first that evolved it. It is probably a fair generalization to say that dorylines use preformed cavities for nesting and move their colonies often relative to other ants, particularly in the case of the ‘true army ants’ (see below).

Dorylines occur on all continents except Antarctica but are the most prominent in tropical regions of the world. A few species range into the warm temperate zone, as far as the state of New Jersey in northeastern United States and western Turkey and the Dodecanese in the Mediterranean. In the southern hemisphere, they reach southern Australia and Tasmania, South Africa, and at least as far as Chubut province in southern Argentina.

Within the subfamily, a group of genera sometimes termed ‘the true army ants’ (or ‘AenEcDo’ army ants; Kronauer 2009) can be distinguished. The true army ants include the genera *Aenictus*, *Aenictogiton*, *Cheliomyrmex*, *Dorylus*, *Eciton*, *Labidus*, *Neivamyrmex*, and *Nomamyrmex*. These ants possess what has been termed the ‘army ant syndrome’: a set of morphological and behavioral characteristics that are common to all species of the group (Brady 2003). The syndrome includes collective foraging, frequent colony relocations, and specialized queen morphology. The true army ants always forage in highly coordinated swarms with no scouts or leading foragers. There are no permanent nests and colonies move periodically, a behavior that is thought to be an adaptation to local depletion of resources. The queens are also highly specialized, wingless and with large abdomens (dubbed ‘dichthadiigynes’), capable of producing large numbers of eggs. Colony foundation in army ants is also unusual. Young queens are unable to start a new colony independently as in most ant species, and instead they rely on a retinue of workers departing with them from their parent nest. An extensive body of literature exists concerning army ant biology and excellent, readable overviews of the subject have been published (Gotwald 1995, Kronauer 2009).

The true army ants currently account for the majority of doryline diversity. More than 45% of the described species of Dorylinae are classified in just two true army ant genera: Aenictus (Figure 9, occurring in the Old World) and Neivamyrmex (Figure 37, New World). As noted above, the army ants are where the largest colony sizes and most conspicuous foraging behaviors evolved. Several species became adapted to taking a variety of invertebrate prey in addition to the more standard social insect diet and during foraging they form the trails that are such a memorable sight to most visitors to the tropics. These formidable columns of ants are observed in the genera *Eciton* (Figure 24) and *Labidus* (Figure 28), in the New World and *Dorylus* (Figure 20) in Sub-Saharan Africa and Southeast Asia. In addition to conspicuous predators with varied diets, *Dorylus* and *Eciton* contain species that retain the ancestral condition of preying upon other ants. The latter specialization seems to be a rule for most other species and genera (*Aenictus* in the Old World, *Neivamyrmex* and *Nomamyrmex* (Figure 42) in the New World) of the true army ants for which we have foraging observations.

The sexual dimorphism of the true army ants is remarkable, even compared to other ants, and has contributed to a complex early taxonomic history and the establishment of a ‘dual taxonomy’ in which descriptions of new species were often based on males unassociated with any females (see History of taxonomic and phylogenetic research below).

*Aenictogiton*, an African lineage now recognized as the sister group to *Dorylus* and thus in phylogenetic terms nested within the true army ant clade (Brady et al. 2014), deserves a separate mention. The genus has been originally described from a male (Emery 1901b) and its worker remained a mystery for over one hundred years, until the first workers were collected in Uganda in 2006. Since then more workers have been uncovered and their conspecificity with *Aenictogiton* males from the same area was confirmed through molecular phylogenetics (unpublished data). The present study is the first to formally describe the worker caste of this elusive genus. The worker morphology of *Aenictogiton* is reminiscent of certain species of the distantly-related *Leptanilloides* and suggests that these ants are strictly subterranean. This is the only lineage of the true army ants for which no behavioral observations have ever been published.

In addition to the true army ants, the subfamily Dorylinae comprises a variety of forms that prior to the study of Brady et al. (2014) had been classified in the erstwhile subfamilies Cerapachyinae and Leptanilloidinae. The relationships among these lineages are still not entirely understood, but it is now certain that they do not represent a monophyletic group (Borowiec, in prep.). Many of the genera discussed below have been treated as a single genus *Cerapachys* prior to this revision (Brown 1975). The scarce information on foraging habits indicates that most species prey on other ants or on termites. It is unclear if any species of this assemblage evolved the complete suite of the army ant syndrome, although collective foraging and specialized queens similar to those found in true army ants are known in some lineages.

### History of taxonomic and phylogenetic research

In much of the myrmecological literature, the true army ants and other ants classified here as dorylines (sometimes referred to as ‘non-army ant dorylines’; all other genera of the former subfamilies Cerapachyinae and Leptanilloidinae) have not been universally recognized as close relatives. As a result of this, the research on the taxonomy and phylogeny of these assemblages has followed largely separate paths. Because of this I keep these histories separate in the account that follows, concluding with a review of how phylogenetic considerations brought these groups together. The summary presented here focuses on the classification at the genus level and above. Remarks on species-level taxonomy can be found under the individual genus accounts.

The first taxon that would eventually be included in the Dorylinae is *Dorylus helvolus*, which was described from a male by Linnaeus in 1764 as *Vespa helvola*. The convoluted taxonomic history of this species and the true army ants in general has been vividly described by Gotwald (1982, 1995). Male army ants are very distinctive insects and early entomologists were confused about the identity of these forms because of the absence of worker-male associations. It was not until 1849 when Thomas Savage, a missionary in Africa, was able to observe the strange males together with workers in the field, that this first ‘army ant mystery’ was solved (Savage 1849).

The descriptive work on the true army ants continued with unfortunate proliferation of taxa described based on workers, males, or even gynes unassociated with other sexes or castes. This practice has been especially common in *Aenictus* and *Dorylus* but has impacted most true army ant lineages. The result of this approach is a ‘dual taxonomy’ with many forms known from only the worker caste or from males, but not both. In the genus *Aenictus*, for example, about 50 out of the 180 currently recognized species are known only from the male and no species is known from worker, gyne, and male.

Morphologically distinct lineages of the true army ants exist in the Old and New World and the two regions have not shared a genus-level taxon since at least 1840 (Shuckard 1840a). The affinity of the these ants, however, has long been recognized and the family-level taxon Dorylidae was first erected by Leach (1815) to hold the then-known males of Old World *Dorylus* and New World *Labidus*. The scope and rank of the Dorylinae have been in a flux throughout of most of its history. In Emery’s landmark Genera Insectorum (1910) treatment the Dorylinae was a subfamily of the Formicidae consisting of three tribes: ‘Ecitini’ (correct spelling Ecitonini) to accommodate the New World forms and the genus *Aenictus*, the Dorylini for *Dorylus* of the Old World, and the Leptanillini for the ants, now considered unrelated, that independently acquired queen morphologies similar to those of certain dorylines. Ecitonines were later treated as a subfamily (Brown 1973, Snelling 1981) and most subsequent authors followed suit (e.g. Gotwald 1982, 1995). *Aenictus* was first treated as separate from Ecitonini by Borgmeier (1955) who placed them in Aenictini, later given subfamily status by Bolton (1990b). The higher classification of the true army ant clade thus followed a trend of proliferation of subfamilies that culminated in the recognition of four subfamilies: Aenictinae, Aenictogitoninae, Dorylinae, and Ecitoninae (Baroni Urbani et al. 1992). This was reversed only recently with a synonymization of all these names plus Cerapachyinae and Leptanilloidinae under Dorylinae (Brady et al. 2014).

At the genus level, the current classification of the New World army ants was firmly established by Borgmeier in his monographic studies on these taxa (Borgmeier 1953, 1955), where he treated all five genera recognized in the present study. In the Old World, the generic classification has also been stable, with *Aenictus* and *Dorylus* both recognized in Emery’s Genera Insectorum (1910). *Dorylus* has traditionally been divided into a number of subgenera, including the non-monophyletic *Anomma* to accommodate the above-surface generalist foragers (Kronauer et al. 2007). Because the Old World genus *Aenictogiton* was known only from males, it has not always been associated with the army ants, but its generic concept has not changed since its original description (Emery 1901b).

The taxonomic history of non-army ant dorylines began with Alfred Russell Wallace collecting a worker specimen later described by Fredrick Smith of the British Museum (Smith 1857) as *Cerapachys antennatus*. Subsequently Roger (1861, 1862) introduced two new names: *Syscia* and *Ooceraea*. Roger considered the former among his ‘*Ponera*-like ants’ but he gave no discussion of the affinities of the latter. The small postpetiole and large abdominal segment IV characteristic for *Ooceraea* led Mayr (1865) to include the genus in Myrmicinae. Several other generic names were added later, including *Sphinctomyrmex*, *Cylindromyrmex*, *Lioponera*, *Parasyscia*, *Acanthostichus*, and *Simopone*. Finally, Forel (1893) recognized their affinity and placed all of the mentioned above (including *Syscia* and *Ooceraea*) under his tribe ‘Cerapachysii’ within Ponerinae. Wheeler (1902) proposed a classification where he recognized a subfamily Cerapachyinae separate from Dorylinae and Ponerinae, and with three tribes: ‘Acanthostichii’, ‘Cerapachyi’, and ‘Cylindromyrmii’.

The general trend in genus-level taxonomy can be summarized as a progressive addition of new names coupled with increasing number of *Cerapachys* synonyms. This lumping of names with *Cerapachys* began with Emery (Emery 1902), who published an early reclassification of the genus. He recognized five subgenera: *Cerapachys* *s. str.*, *Parasyscia*, *Ooceraea*, *Syscia*, and *Cysias*. His classification was largely based on the number of antennal segments and abdominal morphology. In the same work he also erected a new genus, *Phyracaces*, and considered *Lioponera* as separate from *Cerapachys*. Emery’s Genera Insectorum (Emery 1911) treatment of the Ponerinae stabilized the classification of cerapachyines under three tribes and with *Sphinctomyrmex* (with two subgenera), *Cerapachys* (four subgenera), *Phyracaces*, *Lioponera*, *Acanthostichus* (two subgenera), *Cylindromyrmex*, and *Simopone* as constituent genera. Although this classification remained largely unchallenged for years to come, new genus-level names continued to be added, including *Procerapachys*, *Nothosphinctus*, *Zasphinctus*, *Chrysapace*, *Hypocylindromyrmex*, *Metacylindromyrmex*, *Aethiopopone*, and *Neophyracaces*. Most of these were introduced as subgenera and in general have not been widely used. Brown’s landmark revision (Brown 1975) established a generic classification that has not been overturned until the present treatment. Brown recognized only four genera and no valid subgenera in his Cerapachyini: *Cerapachys* (with nine synonyms), *Simopone*, *Sphinctomyrmex* (four synonyms), and *Leptanilloides*. He also recognized *Acanthostichus*, *Ctenopyga*, and *Cylindromyrmex* (three synonyms) as good genera but relegated them to two other tribes, the Acanthostichini and Cylindromyrmecini. Subsequent changes to this scheme included synonymy of *Ctenopyga* under *Acanthostichus* (MacKay 1996) and the classification and taxonomy of *Leptanilloides* and its close relatives (see below). Brown’s treatment of *Cerapachys* was very broad and although he recognized the morphological disparity of different forms, he preferred informal species groups to subgenera. Much later Xu (2000) described a very distinctive ant from Yunnan, China, for which he introduced a new generic name, *Yunodorylus*. This short-lived genus was soon also synonymized with *Cerapachys* (Bolton 2003). Such an all-encompassing definition of *Cerapachys* made it a polyphyletic taxon, as revealed through recent molecular phylogenetic research (Brady 2003, Brady et al. 2014; discussed below). The most recent advancement in genus-level taxonomy was the study of Bolton and Fisher (2012), focusing on ‘*Simopone* genus-group’, an assemblage of likely non-monophyletic taxa (Brady et al. 2014) characterized by similar morphologies. This work introduced two new non-army ant genera, *Tanipone* and *Vicinopone*.

In 1923 a genus of distinctive, minute and blind ants, *Leptanilloides*, was first described (Mann 1923) from Bolivia. This taxon was first classified as Dorylinae, Ecitonini and later transferred to its own subfamily (Baroni Urbani et al. 1992, Brandão et al. 1999a). Another genus, *Asphinctanilloides*, was added to the subfamily and molecular phylogenetics revealed that the male-based name *Amyrmex* also belonged to this lineage and not to Dolichoderinae where it had been long placed (Ward and Brady 2009). Following additions of multiple new species to *Leptanilloides* (Longino 2003, Donoso et al. 2006, Borowiec et al. 2011, Silva et al. 2013), Brady et al. (2014) synonymized Leptanilloidinae under Dorylinae. This present revision goes one step further and considers *Asphinctanilloides* and *Amyrmex* synonyms of *Leptanilloides*.

The study of doryline phylogeny and evolution is intertwined with taxonomic considerations, although a summary somewhat independent from the above account, which focuses primarily on the nomenclature, is possible. The work on relationships within the true army ants centers on the controversy of whether they evolved once (the monophyly hypothesis) or more times independently (the polyphyly hypotheses; Gotwald 1979). The affinity of the New World and Old World army ants has been recognized very early on because of similar overall morphologies of males, but this hypothesis of monophyletic origins later became a matter of contention. Certain authors suggested that these lineages were derived independently; perhaps even three times (Brown 1975, Gotwald 1979). This line of thought prompted separating the true army ants into separate subfamilies, as mentioned above. This view persisted until 1990s and in Bolton’s important morphological study (Bolton 1990b), the phylogenetic tree he drew suggested independent evolution of ecitonines and dorylines plus aenictines, with the clade comprising *Dorylus* and *Aenictus* more closely related to cerapachyines (considered monophyletic). Later cladistic work, however, supported the monophyletic hypothesis (Baroni Urbani et al. 1992, Brady and Ward 2005). Brady (2003) was the first molecular phylogenetic study to investigate this problem and recovered monophyletic true army ants. This result was recently repeated in a much more comprehensive molecular phylogenetic study, although statistical support was low (Brady et al. 2014). Genomic data, however, reveals that the monophyly hypothesis is likely driven by bias and strongly suggests that New World and Old World army ants evolved independently (see below; Borowiec, in prep.).

Early views on the relationship of the ‘cerapachyines’ to the true army ants were conflicting; these opposing perspectives were succinctly summarized by William Morton Wheeler (1902). He first presented Carlo Emery’s view, which represented an early recognition that these ants are related (Emery 1895b, 1901b), and then contrasted it with the views of Auguste Forel and his own, stating that Cerapachyinae is ‘the most archaic and generalized of existing Formicidae’ and stressing affinities to the Ponerinae. Emery’s early considerations recognize the similarities of doryline and cerapachyine males, which in both groups have retractable genitalia, forked subgenital plate (abdominal sternite IX) and no cerci. Forel (1899, 1901b) and Wheeler (1902), on the other hand, focus on the differences in ‘general habitus’, behavior, and colony sizes, pointing out that cerapachyines appear to have small colonies that are nothing like those of dorylines. Emery seems to have yielded under the pressure from his peers and included cerapachyines in his treatment of the Ponerinae for Genera Insectorum (Emery 1911). In his treatment of the Dorylinae for Genera Insectorum published a year prior (Emery 1910), he remarks: ‘All agree against me that is best to leave the groups of the type of *Cerapachys*, *Acanthostichus*, and *Cylindromyrmex* with the ponerines. I will not insist on being right against the unanimity of my colleagues in myrmecology’. At the same time, Emery managed to preserve an implication of doryline affinity by considering cerapachyines as a part of ‘sectio Prodorylinae’.

Wheeler (1920) later pointed out that Prodorylinae and Cerapachyinae are essentially equivalent and that the latter should be used. At the same time he changed his mind and recognized that cerapachyines and true army ants are related, following his observation of raiding behavior of *Lioponera* (then *Phyracaces*) and studies on larval morphology. Despite this, however, the early views of Forel and Wheeler prevailed, and cerapachyine ants were either considered as members of the Ponerinae or a separate group with affinities to the latter. This was where Brown (1975) placed them in his study of the group, although he discusses Emery’s original ideas at some length. It wasn’t until 1990 when Bolton (1990a, 1990b) returned to the ideas of cerapachyine-doryline affinity and, through a careful morphological investigation, convincingly showed that the then recognized subfamilies Aenictinae, Cerapachyinae (including *Leptanilloides*; see below), Dorylinae, and Ecitoninae form a monophyletic group.

Modern research on the phylogeny of the Dorylinae began with Bolton’s two influential works, as already signaled above (Bolton 1990a, 1990b). Dissections of abdominal sclerites and the morphology of the mesosoma led him to conclude that the ‘cerapachyines’ are distinct from the Ponerinae and he then resurrected Emery’s ideas about their close relationship with the true army ants. These views held to scrutiny with a more formal cladistic analysis of morphological characters conducted by Baroni Urbani et al. (1992), who also included *Aenictogiton* in their considerations. This landmark study was the first to focus on a formal reconstruction of relationships among all ant subfamilies using a matrix of morphological characters analyzed with maximum parsimony. It recognized a monophyletic grouping of six subfamilies that now constitute the Dorylinae, in subsequent literature referred to as the ‘doryline section’ or the ‘dorylomorph group’. Later research by Seán Brady and Phil Ward (Brady 2003, Brady and Ward 2005) focused on the phylogeny of this clade, with particular emphasis on the true army ants. The 2005 study confirmed a number of synapomorphies for the doryline group of subfamilies originally pointed out by Bolton (1990b), and cemented it as a monophyletic group that can be recognized using morphology as well as molecules. The speciose and morphologically diverse ‘*Cerapachys*’ was not extensively sampled in these phylogenies, but the several species that were included already suggested that the genus was not monophyletic.

### Current views on doryline classification and evolution

The current limits of the subfamily were not established until the molecular study of Brady et al. published in 2014. As explained above, prior to that, the genera of this group had been classified in as many as six subfamilies. The affinities of those subfamilies had not always been recognized, but a close relationship has since been convincingly demonstrated through a series of independent morphological (Bolton 1990b, Baroni Urbani et al. 1992, Brady and Ward 2005) and molecular phylogenetic studies (Brady 2003, Brady et al. 2006).

The above mentioned study of Brady and colleagues (Brady et al. 2014) was a major advancement of our understanding of doryline phylogeny. These authors sampled representatives of all of Brown’s *Cerapachys* species groups and 26 out of the 27 extant doryline genera treated here. This work showed that a number of well-circumscribed monophyletic lineages exists within what was called *Cerapachys*. The phylogeny also shows that these lineages taken together do not form a clade. General patterns recovered in this phylogeny show the importance of geography, with most of the strongly supported monophyletic groupings comprising either Old World or New World lineages. There is moderate support for the monophyly of the true army ants, as well as for a major split between the New World genera (*Cheliomyrmex*, *Eciton*, *Labidus*, *Neivamyrmex*, *Nomamyrmex*) and Old World army ants (*Aenictus*, *Aenictogiton*, *Dorylus*), a result also recovered in an earlier molecular phylogeny (Brady 2003). The phylogeny contains many short internodes and a time-calibrated analysis suggests that most of the lineages recognized here as genera diversified in the first 20 million years of the group’s evolution. This apparently rapid initial divergence was perhaps spurred by the transition to preying on brood of other ants (Brady et al. 2014). Following synonymization of the six subfamilies, Brady et al. (2014) did not propose a tribal classification within the group, reflecting the lack of understanding of many relationships.

**Figure 1.**
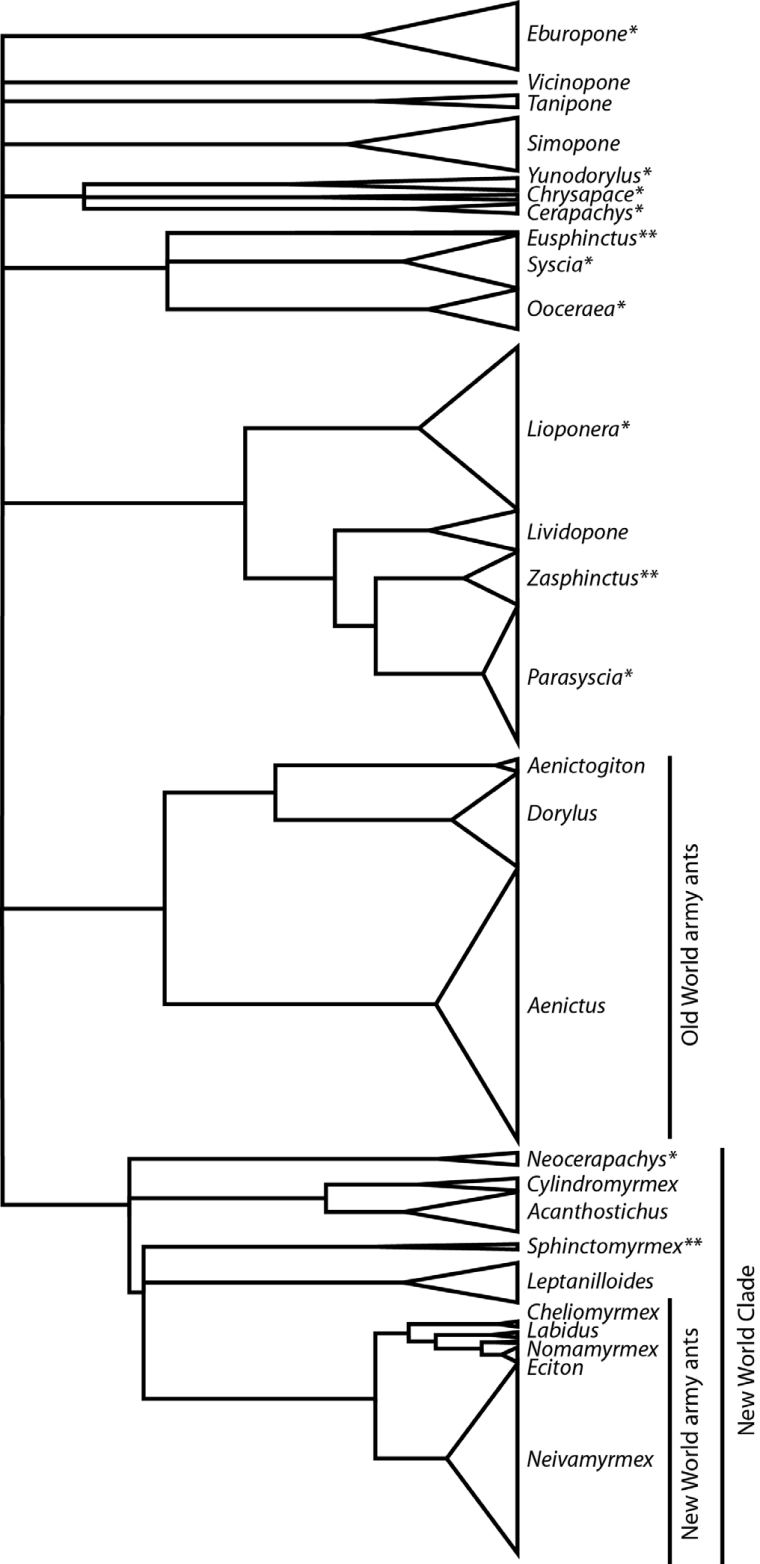
Phylogenetic relationships among the genera of Dorylinae based on Brady et al. 2014 and unpublished genomic data (Borowiec, in prep.). Taxa marked with asterisk (*) were classified in *Cerapachys* prior to this revision and those with double asterisk (**) were included in *Sphinctomyrmex*. Size of the triangles is proportional to estimated species diversity, i.e. include described and undescribed species. ‘New World army ants’ and ‘*Eciton* genus-group’ are equivalent and I use these names interchangeably.

Recent advances in DNA sequencing techniques provide orders of magnitude more data than has been used in phylogeny reconstruction in previous studies (Brady 2003, Brady et al. 2014), and this approach has been applied to the doryline phylogeny (Borowiec, in prep.). The results reveal that army ant monophyly is likely an artifact driven by systematic bias resulting from model mis-specification. The new findings also show an even stronger signal of broad biogeographic patterns. Even the genomic data, however, do not provide confidence in all relationships at the backbone of the doryline tree. The phylogeny derived from this unpublished genomic data is summarized in Figure 1. All the genera recognized here appear monophyletic and a few well-supported clades that include more than one genus are recovered. These clades include (1) a clade of *Chrysapace*, *Cerapachys*, and *Yunodorylus*, (2) a clade comprising *Eusphinctus*, *Ooceraea*, and *Syscia*, (3) a well-defined clade that was already strongly supported by earlier analyses (Brady et al. 2014), containing *Lioponera*, *Lividopone*, *Parasyscia*, and *Zasphinctus*, (4) a clade uniting all New World dorylines except for the Central and North American species of *Syscia*, and (5) Old World army ants clade, which comprises *Aenictogiton*, *Aenictus*, and *Dorylus*. The genera *Eburopone*, *Simopone*, *Tanipone*, and *Vicinopone* cannot be placed with confidence at any particular position on the doryline tree.

Morphological characters uniting morphologically disparate genera in clade (1) are not obvious, although all these ants currently occur in the Indomalayan region (at least one *Chrysapace* is known from Eocene Baltic amber). The members of the three genera in clade (2) are mostly soil- and leaf litter-dwelling species and are characterized by small or entirely absent eyes in the worker and reduced antennal segment count in both females (from 12 to 11 or 9) and males (from 13 to 12 or 11). This antennal count reduction is universal across species of this group, although independent reductions occurred elsewhere in the Dorylinae. Clade (3) is well-defined and phylogenetic relationships within it are also very well-supported. The members of this group share the loss of the forewing costal vein, a trait which is present in many other dorylines. Genomic data also strongly suggest a ‘New World Clade’ (4). Within that large clade, the termite hunters *Acanthostichus* and *Cylindromyrmex* form are sister genera, and New World army ants (equivalent of the former Ecitoninae) form another well-supported monophyletic group. It is unclear which genus is sister to New World army ants but the data suggest either *Leptanilloides* or *Sphinctomyrmex*. Morphological synapomorphies for both the large New World clade and the clade uniting New World army ants with *Leptanilloides* and *Sphinctomyrmex* remain elusive. The New World army ants, or *Eciton* genus-group, which by themselves undoubtedly form a clade, however, possess a highly derived morphology and are well-differentiated from other dorylines. Many of the characters found in New World army ants appear to be independently derived in the Old World army ants clade (clade 5), which comprises *Aenictogiton*, *Aenictus*, and *Dorylus*. Within that group *Aenictogiton* is sister to *Dorylus*, forming a clade from which *Aenictus* apparently diverged a long time ago (Borowiec, in prep.).

A pattern emerges when combining morphological scrutiny of doryline ants with the molecular phylogenetic results: most groupings for which a strong signal of monophyly was recovered in the molecular phylogenies are also easily diagnosed using morphology. This is especially true for all the genera recognized here, but less so for most above-genus groupings. The present revision builds upon this fact and resurrects a number of names hitherto treated as synonyms of *Cerapachys* or *Sphinctomyrmex* to better reflect evolutionary relationships. It also introduces two new generic names for taxa previously placed in *Cerapachys*, and it synonymizes others. The total number of recognized doryline genera is raised from 19 (Brady et al. 2014) to 28. Species previously classified under *Cerapachys* are here treated in 9 genera, and species of the former *Sphinctomyrmex* are placed in three genera. I do not propose a tribal classification for the subfamily. As explained above, several lineages cannot be conclusively placed on the backbone of the doryline tree. This means that in order to effectively apply tribal names to monophyletic groups one would need to erect at least four tribes with only one genus each (*Eburopone*, *Simopone*, *Tanipone*, and *Vicinopone*) and then either erect a single tribe for the New World clade (Figure 1) or further split that group into multiple monogeneric tribes to preserve the distinct ‘Ecitonini’. Here I chose an alternative approach and abandon tribal classification altogether, instead referring to clades grouping multiple genera with informal names such as ‘Old World army ants’ or ‘*Eciton* genus-group’.

It is worth stating here that the division of these taxa into multiple genera is not motivated solely by the need for monophyletic groupings in modern classifications, but also by the fact that the species hitherto classified under *Cerapachys* exhibit unusual and confusing variation in morphological characters that have traditionally been used to delimit genera in other ant groups. These characters include the number of palpal segments, number and development of tibial spurs, the development of abdominal segment III (postpetiole), as well as other features. I believe that the revised classification not only better reflects the phylogeny, but also targets for assignment of names those clades that are most morphologically distinct. Reorganization of the ‘*Cerapachys*’ diversity into more manageable taxa will hopefully stimulate future species-level revisions and permit easy integration of newly discovered forms into this new framework.

### Classification of the Dorylinae proposed here

The classification proposed here is outlined below. The general distribution and the number of valid described species (excluding subspecies) are given in parentheses. Biogeographic divisions follow those outlined by Cox (2001). Indomalayan region comprises Indian subcontinent and Southeast Asia west of Wallace’s line and the Australasian region includes Australia and Pacific islands east of Wallace’s line.

**Dorylinae Leach, 1815** (worldwide, 27 extant genera and 1 extinct genus, 685 described extant and 8 extinct species)

= Acanthostichini Emery, 1901a

= Aenictinae Emery, 1901a

= Aenictogitoninae Ashmead, 1905

= Cerapachyinae Forel, 1893a

= Cheliomyrmecini Wheeler, W. M., 1921

= Cylindromyrmecini Emery, 1901a

= Ecitoninae Forel, 1893a

= Eusphinctinae Clark, 1951

= Leptanilloidinae Baroni Urbani, Bolton & Ward, 1992

= Lioponerini Ashmead, 1905

***Acanthostichus* Mayr, 1887** (Nearctic, Neotropical, and Dominican amber, 23 extant and1 fossil species)

= *Ctenopyga* Ashmead, 1906

***Aenictogiton* Emery, 1901b** (Afrotropical, 7 extant species)

***Aenictus* Shuckard, 1840b** (Palearctic, Afrotropical, Indomalayan, and Australasian, 184 extant species)

= *Paraenictus* Wheeler, W. M., 1929

= *Typhlatta* Smith, 1857

***Cerapachys* Smith, F., 1857** (Indomalayan, 5 extant species)

= *Ceratopachys* Schulz, 1906

***Cheliomyrmex* Mayr, 1870** (Neotropical, 4 extant species)

***Chrysapace* Crawley, 1924a, gen. rev.** (Malagasy, Indomalayan, and Baltic amber, 3 extant and 1 undescribed fossil species)

***Cylindromyrmex* Mayr, 1870** (Neotropical and Dominican amber, 10 extant and 3 fossil species)

= *Holcoponera* Cameron, 1891

= *Hypocylindromyrmex* Wheeler, W. M., 1924a

= *Metacylindromyrmex* Wheeler, W. M., 1924a

***Dorylus* Fabricius, 1793** (Palearctic, Afrotropical, and Indomalayan, 60 extant species)

= *Alaopone* Emery, 1881, **syn. n.**

= *Anomma* Shuckard, 1840c, **syn. n.**

= *Cosmaecetes* Spinola, 1851

= *Dichthadia* Gerstäcker, 1863, **syn. n.**

= *Rhogmus* Shuckard, 1840c, **syn. n.**

= *Shuckardia* Emery, 1895b

= *Sphecomyrmex* Schulz, 1906

= *Sphegomyrmex* Imhoff, 1852

= *Typhlopone* Westwood, 1839, **syn. n.**

***Eburopone* Borowiec, gen. n.** (Afrotropical and Malagasy, 1 extant species)

***Eciton* Latreille, 1804** (Neotropical, 12 extant species)

= *Camptognatha* Grey, 1832

= *Holopone* Santschi, 1925

= *Mayromyrmex* Ashmead, 1905

***Eusphinctus* Emery, 1893a, gen. rev.** (Indomalayan, 2 extant species)

***Labidus* Jurine, 1807** (Nearctic and Neotropical, 7 extant species)

= *Nycteresia* Roger, 1861

= *Pseudodichthadia* André, 1885

***Leptanilloides* Mann, 1923** (Nearctic and Neotropical, 19 extant species)

= *Amyrmex* Kusnezov, 1953, **syn. n.**

= *Asphinctanilloides* Brandão, Diniz, Agosti & Delabie, 1999, **syn. n.**

***Lioponera* Mayr, 1879, gen. rev.** (Palearctic, Afrotropical, Malagasy, Indomalayan, and Australasian, 73 extant species)

= *Neophyracaces* Clark, 1941, **syn. n.**

= *Phyracaces* Emery, 1902, **syn. n.**

***Lividopone* Fisher and Bolton, 2016** (Malagasy, 1 extant species)

***Neivamyrmex* Borgmeier, 1940** (Nearctic, Neotropical, and Dominican amber, 127 extant and 1 fossil species)

= *Acamatus* Emery, 1894

= *Woitkowskia* Enzmann, 1952

***Neocerapachys* Borowiec, gen. n.** (Neotropical, 2 extant species)

***Nomamyrmex* Borgmeier, 1936** (Nearctic and Neotropical, 2 extant species)

***Ooceraea* Roger, 1862, gen. rev.** (Pantropical; native in Indomalayan and Australasian, 11 extant species)

= *Cysias* Emery, 1902, **syn. n.**

***Parasyscia* Emery, 1882, gen. rev.** (Palearctic, Afrotropical, Malagasy, Indomalayan, and Australasian, 50 extant species)

†***Procerapachys* Wheeler, W. M., 1915b** (Baltic amber, 3 fossil species)

***Simopone* Forel, 1891** (Afrotropical, Malagasy, Indomalayan, and Australasian, 39 extant species)

***Sphinctomyrmex* Mayr, 1866b** (Neotropical, 3 extant species)

***Syscia* Roger, 1861, gen. rev.** (Nearctic, Neotropical, and Indomalayan, 5 extant species)

***Tanipone* Bolton & Fisher, 2012** (Malagasy, 10 extant species)

***Vicinopone* Bolton and Fisher, 2012** (Afrotropical, 1 extant species)

***Yunodorylus* Xu, 2000b, gen. rev.** (Indomalayan, 4 extant species)

***Zasphinctus* Wheeler, W. M., 1918, gen. rev.** (Afrotropical and Australasian, 20 extant species)

= *Aethiopopone* Santschi, 1930, **syn. n.**

= *Nothosphinctus* Wheeler, W. M., 1918, **syn. n.**

### Morphology

The Dorylinae possess a number of characteristic morphological traits, which are discussed in detail below. A Hymenoptera Anatomy Ontology (HAO) table of annotated terms used here, along with their approximately corresponding HAO definitions, is provided in the Appendix (Yoder et al. 2010).

**Figure 2.**
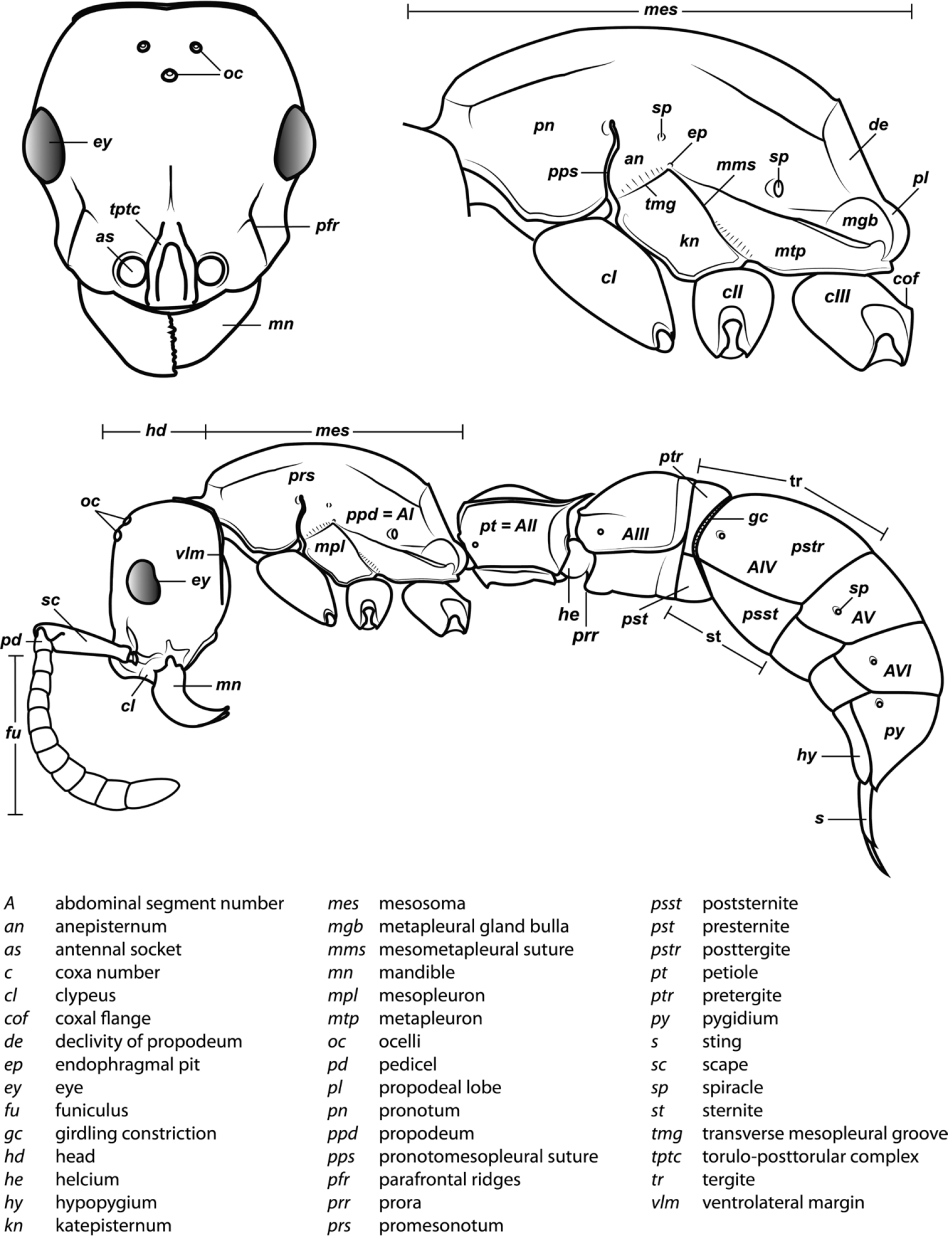
Dorylinae worker morphology based on *Lioponera clarus*.

**Figure 3.**
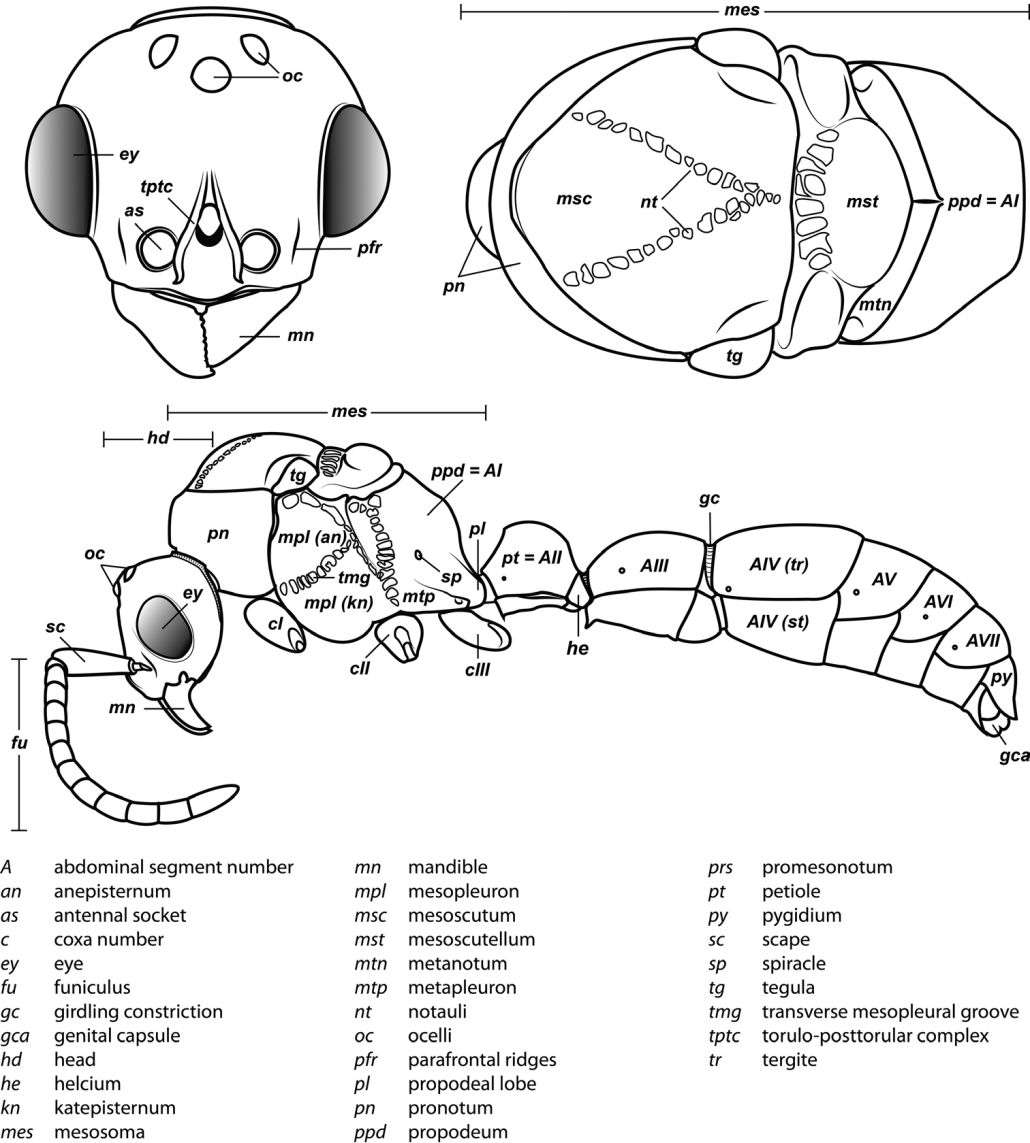
Dorylinae male morphology based on Lioponera cf. mayri.

**Figure 4.**
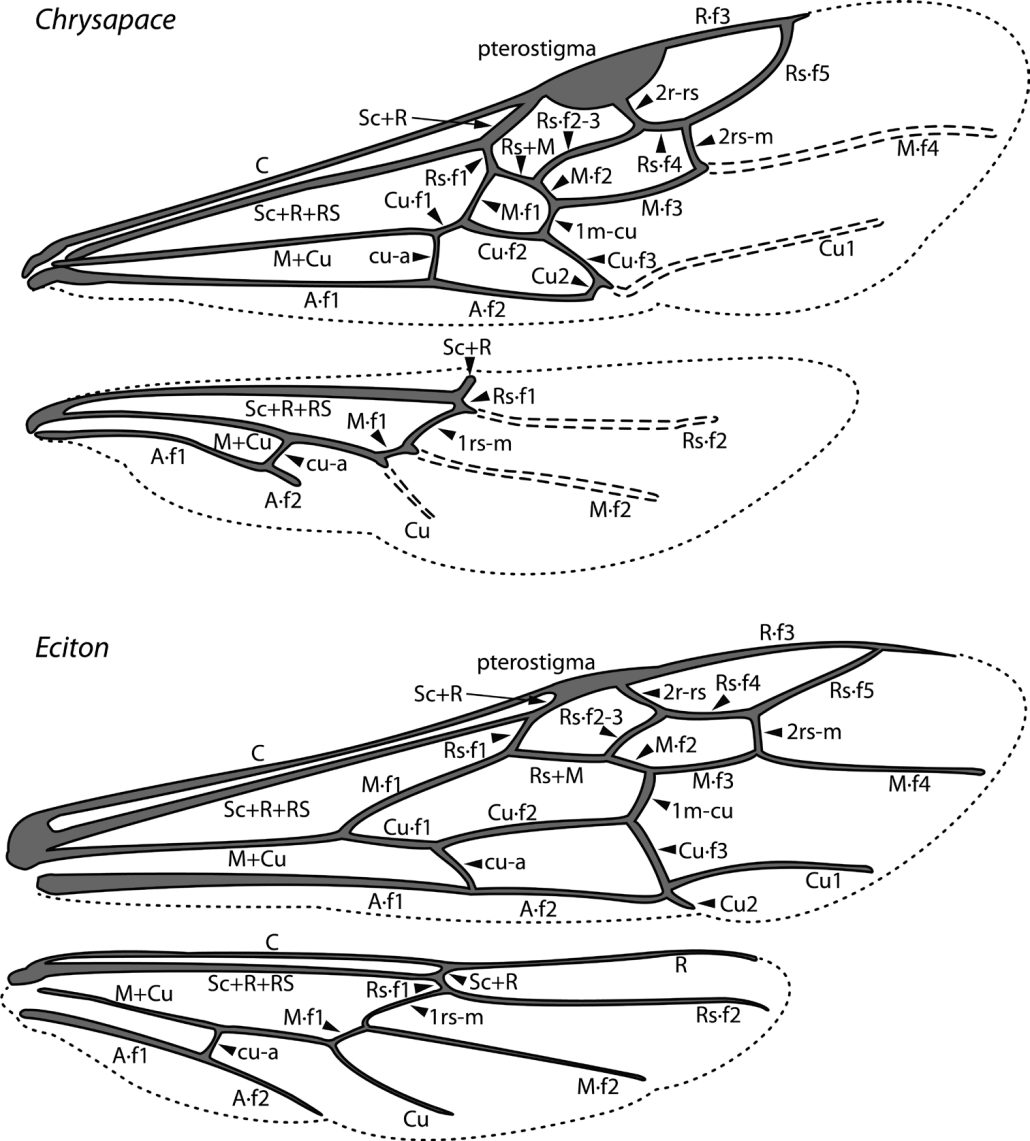
Dorylinae wing venation based on *Chrysapace* sp. and *Eciton* sp.

An illustration of morphological characters used in this revision is presented in Figures 2–4. Below I discuss these characters in hope that this will help to clarify the terminology and facilitate identification.

Doryline ants are characterized by their predation on other social insects, a condition that appears to be apomorphic for the group, but there is also a plethora of morphological characters that distinguish them from other ant lineages. Extensive work grounded in examination of morphology has been done to infer ant phylogeny (Gotwald 1969, Ward 1990, Baroni Urbani et al. 1992, Bolton 2003, Keller 2011), and the evolution of the doryline clade in particular (Bolton 1990a, 1990b, Brady and Ward 2005, Barry Bolton’s unpublished work, this study). As a result, multiple morphological characters have been identified as diagnostic or possibly synapomorphic for the Dorylinae. The list and discussions below are adapted and updated from the works cited above. Likely synapomorphies are in italics.

### Diagnostic characters of the worker

1. *Lateral area of clypeus very narrow in full face view; distance from anterior clypeal margin to paraoculoclypeal sulcus greater than distance from anterior clypeal margin to frontoclypeal sulcus where antennae insert.*

The clypeal area of the head capsule (Figure 2) is medially delimited by the so-called frontoclypeal sulcus that laterally extends from the midline of the head to the front of the antennal socket area. Lateral to the antennae, the boundary delimiting the clypeus from the rest of the head capsule has been referred to as the paraoculoclypeal sulcus (Keller 2011). In most ants, the distance from the anterior margin of the head capsule (including clypeus) to the frontoclypeal sulcus in the area where the antennae attach is greater than the distance from the margin to the paraoculoclypeal sulcus. In the dorylines the clypeus is very narrow and this condition is reversed. Certain *Cylindromyrmex* and *Acanthostichus* species have the clypeal sulci poorly visible and the medial area of clypeus is protruding forward over the mandibles, thus creating a considerable distance from the antennal socket area to the anterior margin of head capsule. The distance from the anterior margin of the torulo-posttorular complex (see below) to the anterior margin of head, however, is always short and thus the clypeus can be still considered laterally narrow in these species.

A relatively narrow clypeus is present in several ant subfamilies, but the condition described above appears to be restricted to the Dorylinae, *Martialis*, Leptanillinae, Amblyoponinae, and Proceratiinae. I suspect that a thorough study of the clypeal area in the dorylines may reveal new characters of diagnostic or phylogenetic utility within the subfamily.

2. *Parafrontal ridges present, i.e. genae carinate laterally of antennal sockets*.

**Figure 5.**
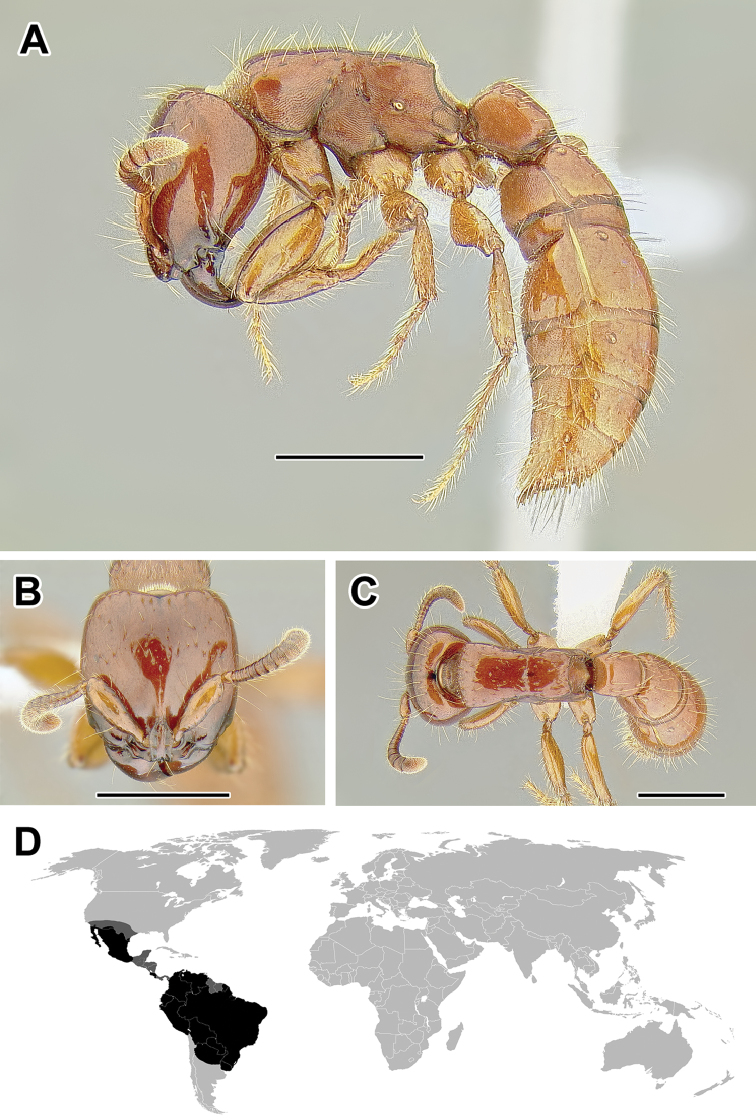
**A–C** Worker of Acanthostichus cf. serratulus (CASENT0732109) **A** Body in lateral view **B** Head in full-face view **C** Body in dorsal view **D** World distribution of *Acanthostichus* (black: present, dark grey: likely present). Scale bar equals 1.0 mm.

Another feature characteristic and likely synapomorphic for the Dorylinae is the presence of often prominent ridges extending some distance back from the paraoculoclypeal sulcus (when visible), laterally to the antennal socket (Figure 2). This feature is present in most doryline species, although it has been reduced several times, for example in *Acanthostichus* (Figure 5) and *Cylindromyrmex*, *Dorylus*, and in some species of *Aenictus*, *Simopone* and *Zasphinctus*. This character was considered a synapomorphy of the Cerapachyinae (Brown 1975, Bolton 2003), even though it is well developed in many New World true army ants.

3. *Torulo-posttorular complex present, often vertical, occasionally horizontal; antennal sockets exposed or (more rarely) partially concealed by torulo-posttorular complex in full-face view*.

Keller (2011) provided much needed clarification of the nomenclature relating to the structures in the antennal socket area in ants. He differentiated frontal carinae from the medial arch of torulus (both structures that arise medially from the antennal socket) and recognized that the relative development and configuration of these structures vary considerably among ants. In the dorylines, the frontal carinae *sensu* Keller have been interpreted to be present only in their posterior part (called posttorular flanges) and fused to the torular lobe (an expansion of the above-mentioned medial arch of the torulus). This whole structure was named torulo-posttorular complex (Keller 2011). This complex in the dorylines can be vertical and thus forming lobes that in full-face view project towards the observer without obscuring the antennal socket area (Figure 2) or, more rarely (*Acanthostichus*, *Cylindromyrmex*, *Simopone*), horizontal or expanded laterally to conceal the antennal sockets. The distinction among the two conditions is not entirely clear-cut however, as multiple lineages or species of the dorylines possess poorly developed lateral expansions of the torulo-posttorular complex that may conceal parts of the medial area of antennal sockets.

The vertical configuration of the torulo-posttorular complex is characteristic and probably synapomorphic to the Dorylinae. A similar morphology is present in some proceratiines, in particular in *Probolomyrmex*. Similar configuration exists in the Leptanillinae and in *Apomyrma* (Amblyoponinae). A horizontally expanded torulo-posttorular complex is also found in the Amblyoponinae (*Myopopone*).

4. *Stipes of maxilla sharply divided into proximal and distal faces, with proximal face extending beyond inner margin of stipes; prementum concealed when mouthparts closed*.

The major sclerite of the maxilla, the stipes, is in dorylines sharply divided into a raised proximal face and sunken distal face on its outer surface (Gotwald 1969). The distal face of the stipes accommodates the labrum when the mouthparts are closed and the proximal face is medially expanded beyond the inner margin of the sclerite. When the mouthparts are closed, this condition causes the prementum (the major sclerite of the labium) to be concealed behind the labrum and the medial extension of the proximal face of the stipes. In most worker ants the stipes is not divided into two distinct faces and prementum is visible when mouthparts are closed, although a carina dividing the stipes is present in numerous taxa. *Aenictus* is an exception among dorylines, as the maxilla in this genus is not divided into two faces and the prementum is visible with mouthparts fully closed. The prementum can also be seen in *Cheliomyrmex*, where the division of the stipital surface is weakly marked. The division of maxilla into two faces is a likely synapomorphy of the Dorylinae, secondarily lost in *Aenictus*.

A similar condition has apparently independently evolved in some Amblyoponinae where the prementum is also concealed (Keller 2011).

5. *Eyes frequently reduced or absent.*

Eyes are poorly developed in most species of the doryline workers, although large and multifaceted eyes are present in a number of lineages. A few speciose lineages lack the eyes completely and without exception in any of the species (e.g. *Aenictus*, *Dorylus*), while many other genera are either blind in most species or with very small eyes present (e.g. *Acanthostichus*, *Eciton*, *Neivamyrmex*, *Syscia*, *Ooceraea*). Large worker eyes can be present in some or all species of *Cerapachys*, *Chrysapace*, *Cylindromyrmex*, *Lioponera*, *Lividopone*, *Simopone*, *Tanipone*, and *Vicinopone*. Many of the species with large eyes are arboreal, but others are surface-foragers or their natural history is unknown.

6. *Mesosoma internally with fused meso- and metafurcal arms, externally corresponding to an endophragmal pit.*

The mesosoma of the Dorylinae worker ants possess a pit in the cuticle, located anteriorly to the propodeal spiracle and near mesometapleural Pronotomesopleural suture (where present; Figures 2 and 14A). This pit, known as the mesosomal endophragmal pit, interiorly corresponds to a junction between cuticular projections that serve as muscle attachments (apodemes) and the lateral wall of the mesosoma. The endophragmal pit may not be discernable with light microscopy in species where it is not surrounded by a cuticular concavity, especially in smaller species. In most ants, the apodemes of the second and third thoracic sternites are separate and bifurcated, and called mesofurca and metafurca, respectively. The mesofurca and metafurca thus form two U- or V-shaped structures inside the mesosoma, with their bases directed posteriorly, and the opening of the ‘V’ directed anteriorly. Some distance from their base, the arms of the mesofurca are additionally connected by a transverse bar of cuticle, the mesofurcal bridge. A variation of this scheme occurs when metafurcal arms first diverge and then fuse together; in some ants the mesofurcal arms point sideways and the mesofurcal bridge forms a triangle but mesofurca and metafurca still separate. In the dorylines, this condition appears to be much modified, with the arms of the mesofurca directed laterally instead of anteriorly and fused to the anteriorly-pointing arms of the metafurca. The laterally projecting fused arms of mesofurca and metafurca then attach to the lateral wall of the mesosoma. In larger species this manifests itself as the mesosomal endophragmal pit. As a result of this modification, in the dorylines the ordinarily transverse mesofurcal bridge points backwards to reach the transverse mesofurcal arms. The character system of mesosomal apodemes has not been methodically investigated in ants but Vilhelmsen et al. (2010) studied these structures in other aculeates.

7. *Metapleural gland orifice concealed beneath a ventrally directed cuticular flap or flange*.

The metapleural gland is a feature found exclusively in ants and is believed to aid in colony sanitation (Yek and Mueller 2011). The gland is internally located in a cuticular chamber at the junction of the metathorax and the propodeum and it opens externally on either side of the mesosoma at a variable distances from the propodeal declivity and above the hind coxae. This metapleural gland orifice and cuticle surrounding it exhibit various modifications in the Formicidae. In the dorylines, the orifice is not visible in dorsal or lateral view because it is overhung by a dorsal flap of cuticle (Figure 2). A similar flap has been reported in *Simopelta* in the Ponerinae (Keller 2011). A dorsal flange also occurs in the Leptanillinae, but the orifice can still be seen in lateral view.

I suspect that a careful study focused on the structures surrounding metapleural gland orifice would reveal additional genus-level diagnostic characters in the Dorylinae.

8. *Helcium sternite bulging ventrally and articulated on the inner wall of the tergite*.

The helcium is a term used for the presclerites of abdominal segment III. The relative development and place of articulation of the helcial sternite varies in ants. The dorylines exhibit a rare condition where the sternite is well developed and bulging ventrally, not obscured by the tergite in lateral view. The sternite is also articulated to the tergite some distance up on the inner wall of the latter, so that the tergite overlaps the sternite. A ventrally bulging helcial sternite appears to occur only in some Proceratiinae, *Tatuidris*, and in the Myrmicinae. In the myrmicine ants, however, the articulation of the sternite and the tergite is along the lateral margins, and thus the sclerites are not overlapping. In all other ants the helcial sternite is flat or only slightly convex, not readily visible in lateral view.

9. *Abdominal segment III with complete tergosternal fusion.*

The degree of fusion of sternites to the tergites of abdominal segments varies in ants (Kusnezov 1955). Bolton (1990b) examined numerous representatives of the Dorylinae (‘the doryline section’) and concluded that in general, the tergite of the abdominal segment III is fused to the sternite in workers and gynes of these ants, although the condition varies in males. Keller (2011) refined this character system by distinguishing whether it is presclerites (helcium) or postsclerites, or both, that are fused. The complete fusion in dorylines is also present in most poneroid ants (most Amblyoponinae, Ponerinae, Proceratiinae) and possibly in *Martialis*. In the formicoid clade, the fusion occurs in Ectatomminae and in Heteroponerinae. In other subfamilies, either the postsclerites or both pre- and postsclerites of abdominal segment III are unfused.

10. *Abdominal segment IV without tergosternal fusion*.

Complete fusion of tergites and sternites of abdominal segment IV is rare in ants and apparently restricted to Agroecomyrmecinae, Ponerinae, and Proceratiinae. In other subfamilies the sclerites are unfused or only presclerites exhibit fusion (Bolton 1990b, 2003).

11. *Spiracles of abdominal segments V–VII shifted posteriorly on each segment, not concealed by the posterior margin of the preceding tergite and visible without distension or dissection.*

This is a character that is a likely synapomorphy of the subfamily and does not appear to occur in any other ants. In most ants the spiracles of abdominal segments I (the propodeum) through IV are visible, but those of abdominal segments V, VI, and VII are ordinarily concealed by the posttergites of their respective preceding segments. These spiracles cannot be thus seen without distension or dissection of the gaster. In the dorylines, however, the spiracles are shifted posteriorly on the posttergites and visible in specimens without any manipulation (Figures 2, 5A). The exposed spiracles are obvious even in species where the size of the distal abdominal segments has been substantially reduced, such as in *Ooceraea*.

12. *Pygidium modified*: either large and with dorsum flattened and armed with teeth or spines, or reduced to a narrow V-shaped sclerite.

In general, the pygidium (last visible abdominal tergite) is derived in the dorylines, departing from a condition of a large, evenly rounded sclerite that is presumed to be plesiomorphic for ants. The degree and nature of the modification varies, however. In many genera previously classified under the Cerapachyinae the pygidium has a flattened medial area and is armed with thick, specialized setae that are thought to have sensory function (Hölldobler 1982). Occasionally the tip of pygidium is also forked, with a prong of variable length along each side of the sting. In *Dorylus* the pygidium also has a flattened disc but it is never armed with numerous modified setae, instead possessing two to four cuticular spines that are tipped with one thick seta each. In *Aenictus*, *Eciton* and other New World army ants, as well as in *Leptanilloides*, the pygidium is modified into a very narrow transverse sclerite that is not armed with any modified spines or setae. A pair or two of thick setae can be, however, present in some New World army ants. Worker *Aenictogiton* is an exception among the Dorylinae, as the pygidium in this genus is large and simple, evenly rounded and without armament of cuticular projections or spine-like setae.

13. *Sting apparatus with furcula fused to base of sting or absent in most species.*

The furcula is a Y- or wishbone-shaped sclerite flexibly attached at the base of the sting (Snodgrass 1984), present in most ants but apparently fused to the sting base in all Dorylinae examined thus far except *Leptanilloides* (Brandão et al. 1999a). In the Aneuretinae, Dolichoderinae, and Formicinae the furcula is also reduced or fused and in *Simopelta*, a ponerine genus with army ant-like habits, this sclerite is also fused to the sting base. The furcula serves as the attachment for muscles responsible for protraction of the sting (Hermann and Chao 1983). These muscles connect directly to the anterior region of the sting bulb in the dorylines where furcula was observed to be fused with the sting (Hermann and Blum 1967, Hermann 1969). It is unclear whether the fusion of furcula and sting is a synapomorphy of the Dorylinae that has reverted to the unfused state in *Leptanilloides* or whether the fusion evolved more than once in the subfamily. A comprehensive and comparative study of the doryline sting is lacking. The sting apparatus appears generally functional and capable of piercing human skin, as in *Eciton*, but more rarely it can be non-functional as a weapon, as in *Dorylus* (Hermann 1969, Bolton 1990b). The sting been described in varying detail in the army ant genera *Aenictus*, *Cheliomyrmex*, *Dorylus*, *Eciton*, *Labidus*, *Neivamyrmex*, and *Nomamyrmex* (Hermann and Blum 1967, Hermann 1969) and in a few non-army ant species including *Leptanilloides* (Brandão et al. 1999a), *Acanthostichus*, *Lioponera*, *Syscia*, and *Zasphinctus* (Hermann 1969).

14. *Metacoxal cavities fully closed, without a Pronotomesopleural suture in the broad annulus*.

The morphology of the cuticle surrounding the sockets where hind coxae articulate (the coxal cavities) varies among ants. The primitive condition is presumably one where the cavities are not fully surrounded by the cuticle (the annulus) and the cavities are connected to the petiolar foramen. This condition is present in Myrmeciinae, Aneuretinae, *Platythyrea* in the Ponerinae, and Ectatomminae. A modification of this state occurs where the annulus surrounds the cavities, so that the cuticle is continuous around the openings, although a Pronotomesopleural suture can be discerned where the outgrowths of the cuticle closing the foramen meet. This state is present in some Amblyoponinae, most Ponerinae, some Heteroponerinae, *Paraponera*, and some Proceratiinae. Finally, the cuticle can be entirely fused and no Pronotomesopleural suture is visible in the cuticle surrounding coxal cavities. This is the condition observed in the Dorylinae. This character state is common among the subfamilies of the formicoid clade, including Dolichoderinae, Formicinae, Myrmicinae, Pseudomyrmecinae, and some Heteroponerinae (Bolton 2003). The cavities are also fully closed and without a Pronotomesopleural suture in some taxa outside the formicoids, namely in the Leptanillinae, *Martialis*, some Amblyoponinae, Agroecomyrmecinae, few Ponerinae, and some Proceratiinae.

15. *Metatibial gland present, located distally on the ventral surface of hind tibia*.

Multiple ant lineages have been found to possess a glandular structure on the ventral (flexor) surface of their hind tibiae (Hölldobler et al. 1996). Given our current understanding of ant phylogeny (e.g. Brady et al. 2006), it is likely that these metatibial glands have evolved more than once. Bolton (1990b) recognized the presence of this gland as characteristic of most dorylines and described the variations in its external manifestation within the subfamily. In some taxa the presence of metatibial gland can be detected only with histological examination, and externally its visibility in very small-bodied species can sometimes be confirmed only through scanning electron microscopy (Borowiec and Longino 2011). The gland is externally visible in most doryline genera and is conspicuous in most true army ants except *Nomamyrmex*, where it is entirely absent, at least externally. Other genera apparently lacking obvious metatibial glands are *Chrysapace*, *Eusphinctus*, *Lividopone*, *Simopone*, *Sphinctomyrmex*, *Tanipone*, and *Vicinopone*. The metatibial gland is also apparently absent in a few species of *Acanthostichus*, *Lioponera*, *Neivamyrmex*, and *Zasphinctus*. Most *Simopone* possess a well-developed gland on the inner surface of hind basitarsus but no gland on the tibia. *Syscia* possess both the metatibial and metabasitarsal glands, the latter of different appearance and apparently independent origin from the one found in *Simopone*.

Glands on the hind tibiae also occur in the Ponerinae (Schmidt and Shattuck 2014), as well as in a few Amblyoponinae and Myrmicinae, although in all these cases the glandular surfaces are positioned differently than in the Dorylinae, suggesting convergence.

As mentioned above, several Dorylinae genera are lacking externally visible metatibial glands and there has been no comprehensive histological study of all the lineages. Given this, coupled with unresolved relationships among most lineages of the subfamily, it is somewhat uncertain whether this character is a synapomorphy of the whole clade or if it is primitively absent from early-branching lineages.

Despite its presence in some of the better-studied species (true army ants, *Ooceraea biroi*), the function of the gland in the Dorylinae is unknown (Billen 2009).

The worker Dorylinae can be thus easily recognized through a combination of metapleural gland orifice concealed by a dorsal cuticular flap, large and convex sternite of the helcium, and exposed abdominal spiracles of segments V–VII.

### Diagnostic characters of the male

1. *Abdominal sternite IX (hypopygium) modified, bidentate to biaculeate*.

The appearance of the abdominal sternite IX of the male is distinctive in the Dorylinae. A simple sclerite with convex or medially tapered posterior margin appears to be the plesiomorphic condition, present in most ants. In the dorylines the hypopygium often has a convex posterior margin and is laterally drawn into two processes, ranging from blunt triangular denticles to long, parallel prongs. Occasionally further modifications, including folds, excisions, and additional teeth are also present on the hypopygium. In some lineages the male hypopygium is useful for species identification. *Leptanilloides* is again the exception, and the sclerite in this lineage is relatively simple, sometimes medially convex or concave. Outside of the Dorylinae, a biaculeate male hypopygium is present in at least two genera, *Paraponera* and *Nothomyrmecia*.

2. *Cerci absent from male genitalia*.

The cerci (also called pygostyles) are paired sensory structures articulating with the last abdominal tergite. Most male ants have cerci but their loss has occurred several times independently, in Leptanillinae, *Martialis*, some Amblyoponinae, and some Proceratiinae (Boudinot 2015). The Dorylinae is the largest clade of ants where male cerci appear to be absent from all species. This character was important for the early recognition of a close relationship of *Acanthostichus* and other ‘cerapachyines’ with the true army ants (Emery 1895).

3. *Genitalia completely retractile*.

The doryline males are able to retract their genital capsule into the abdomen. In other ant taxa, even those that also lack cerci, the genitalia cannot be completely retracted. The genitalia of true army ants are always well-concealed in dead specimens and not visible without dissection. The genitalia of other, particularly smaller dorylines, however, can be partially visible in dead males. The small-sized males of *Leptanilloides* may be an exception among the dorylines, as the known specimens have exerted genitalia, with most of the genital capsule visible without artificial distension or dissection of the abdomen. The abdomen of some *Leptanilloides* species also appears too small to allow for full retraction of the genital capsule.

4. *Jugal lobe absent from hindwing*.

The jugal lobe is a basally located projection of the wing membrane. Bolton (2003) recognized that its presence varies in the hind wing of alate ants. He cites this character as highly polymorphic in the poneromorph subfamilies, absent in the Leptanillinae and the formicoid subfamilies.

The male Dorylinae can be thus recognized by the lack cerci, almost universally bispinose hypopygium, and retractable genital capsule. The last two characters do not apply to *Leptanilloides*, but the cerci are lacking in this genus, too. *Leptanilloides* also has extremely reduced tegulae or is lacking them entirely, a loss perhaps unique among male ants.

### Diagnostic characters of the gyne

Doryline gynes share many worker characteristics and can be recognized by the same putative synapomorphies as the worker, except perhaps for the highly specialized ‘dichthadiigyne’ queens of the true army ants. The latter may be difficult to distinguish from convergently evolved specialized queens of *Leptanilla* (Leptanillinae; Baroni Urbani 1977), *Onychomyrmex* (Amblyoponinae; Wheeler 1916) or *Simopelta* (Ponerinae; Gotwald and Brown 1967). Perhaps the best single character to identify dichthadiigynes as Dorylinae is the *presence of posteriorly shifted abdominal spiracles V-VII, visible on the gaster without distension or dissection of abdominal sclerites*. For further discussion of gyne morphology see ‘Characters used to describe gyne morphology’ below.

### Characters used to describe worker morphology

*Number of antennal segments*. The ant antenna includes only three ‘true’ segments (scape, pedicel, and funiculus), that is metameric structures connected to other segments via muscles, with funiculus further subdivided into secondary structures. Together, the scape, pedicel, and funicular segments are thus sometimes referred to as antennomeres. In the taxonomic literature concerning ants, however, use of ‘antennal segments’ instead of ‘antennomeres’ is widespread and I follow this convention here. In the Dorylinae, the plesiomorphic condition is 12-segmented antennae (Figure 12A) but a number of lineages underwent reduction, either to 11 segments (*Eusphinctus*, many *Ooceraea*, few *Parasyscia*, *Simopone*, many *Syscia*, some *Yunodorylus*, some *Zasphinctus*), 10 segments (most *Aenictus*, at least one *Ooceraea*; Figure 9A), or 8–9 segments (few *Aenictus*, some *Ooceraea*, some *Syscia*). In *Dorylus* the number of antennal segments may vary among individuals from the same colony (Eguchi et al. 2014).

*Relative size of the apical antennal segment.* The size of the last (apical or terminal) antennal segment varies widely within the Dorylinae, both in length and width relative to other segments. The apical segment ranges from small in *Simopone* (Figure 49A) to swollen, much wider than the penultimate segment and longer than several preceding segments together in some *Parasyscia* (Figure 48). This character may potentially aid in genus-level identification but high intrageneric variation and continuous nature of this trait make it less suitable for a dichotomous key.

*Cuticular apron of the clypeus*. Many species in various genera possess a semi-translucent to opaque cuticular projection, or lamella, that arises from the clypeus and closes the gap between mandibles and the head capsule. This trait varies among and within genera.

*Lateroclypeal teeth*. Many dorylines possess cuticular projections that are arising from lateral portions of the clypeus and overhang mandibles (Figure 26B). These projections can be of various sizes and shapes, most commonly finger-like or triangular. This trait varies among and within genera.

*Parafrontal ridges*. As discussed above under worker diagnostic character 2, this is one of distinguishing characters of the Dorylinae worker. A few lineages such as *Acanthostichus* and *Dorylus* seem to lack this trait completely, although reductions of various degree occurred in several genera (see also above under Diagnostic characters of the worker).

*Torulo-posttorular complex.* Another defining feature of the Dorylinae, this character is discussed above under worker diagnostic character 3.

*Antennal scrobes*. Depressions of the cuticle that receive retracted antennal scapes are uncommon in the Dorylinae, well developed only in *Cylindromyrmex* and some species of *Simopone*. Although not considered scrobes here, feebly marked depressions that apparently receive antennal scape can be seen in certain species of *Parasyscia* and *Lividopone*.

*Labrum shape*. Most dorylines have a labrum that is notched medially on its distal (non-articulated) margin, although in a few (*Aenictogiton*, some *Dorylus*, *Leptanilloides*) the margin is evenly rounded across.

*Proximal face of stipes*. The stipites concealing the prementum are another trait of the subfamily that is discussed above under worker diagnostic character 4.

*Number of maxillary palp segments*. This character varies from the plesiomorphic number of six segments in *Tanipone* and most *Simopone* to only one segment in *Aenictogiton*. Although the palpal segment count is apparently constant throughout some genera, its reduction appears to often correlate with small size. As such, and because this character is often impossible to see without dissection, it is of limited utility for genus-level identification.

*Number of labial palp segments*. The discussion regarding maxillary palp segmentation applies to labial palps as well. The labial palps are never composed of more than four segments and as a rule have fewer segments than maxillary palps of the same individual. Exceptions to this rule are the New World army ants and *Acanthostichus*, where the labial palps are longer than maxillary palps, with three and two segments, respectively. In most *Dorylus* both maxillary palps are 2- or 1-segmented and labial palps are 2-segmented but the labial palps are more slender and longer than the maxillary palps. Taken together, the number of maxillary and labial palp segments is sometimes expressed as ‘palp formula’ which simply gives the two numbers separated by a comma. For example, palp formula 4,3 means the maxillary palps are 4-segmented and labial palps are 3-segmented.

*Mandible shape and dentition*. Shape of the mandibles varies across the Dorylinae, with many species retaining plesiomorphic triangular mandibles with well-differentiated basal and masticatory margins and numerous denticles of uniform shape on the latter (Figure 18B). Some dorylines, however, possess falcate mandibles where the distinction between basal and masticatory margins is blurred and a few large, one, or no teeth are present in addition to a pointed mandibular apex (Figure 14B). Derived mandibles are characteristic of lineages with pronounced worker size polymorphism, being especially conspicuous in army ant genera such as *Dorylus* or *Eciton*.

*Eye size*. In general, eyes are small or absent in dorylines, although exceptions do occur, as discussed under worker diagnostic character 5 above.

*Presence of ocelli*. The ocelli are rare in worker dorylines, although *Chrysapace*, *Simopone* (Figure 49B), and *Tanipone* species always possess them. Ocelli also occur in workers of some species of *Cylindromyrmex* and *Lioponera* (Figure 2). Because ergatoid (wingless and worker-like) queens and intercastes are common in the Dorylinae, presence of ocelli can be mistakenly inferred for the worker caste when examining a very worker-like gyne.

*Head capsule above occipital foramen*. Dorylines vary in the degree of differentiation between dorsal (or frontal) and posterior (or occipital) faces of the head. Most species have a distinct posterior surface of the head just anterior to the attachment with the mesosoma. In *Simopone* and *Vicinopone* (Figure 57A) there is no posterior surface and the head is seemingly ‘hanging’ by the dorsal face immediately anterior to occipital carina (see below). Some *Aenictus* and *Neivamyrmex* army ants have a very gradual transition to the posterior face, thus achieving similar appearance (Figures 11, 39).

*Carina on ventrolateral surface of head*. Ventrally on the head of most dorylines, a carina surrounding occipital foramen can be found (see below). Additionally, in some species there may be additional ridges that originate at the lateral corners of that ventral occipital carina and run partways or down the length of ventral head surface towards mandibular insertions (Figure 2). These ridges do not appear to be universally present in any genus except perhaps *Neocerapachys* but can be found in many *Lioponera*, *Lividopone*, and *Parasyscia*.

*Carina surrounding occipital foramen*. Many dorylines possess a carina around the occipital foramen. In some species, this carina joins ventrally to separate the area immediately anterior to the occipital foramen from the rest of the head capsule. This character is variable within and among genera.

*Pronotal flange delimited by a ridge*. The sloping surface of the mesosoma immediately behind the occipital articulation is known as the pronotal flange. This area can be evenly rounding into the pronotal dorsum, also called the pronotal neck, or separated from it by a variously developed cuticular margin. This feature can be consistently present within certain genera like in *Cerapachys* or *Eburopone* (Figure 22A), while in others, such as *Ooceraea* or *Parasyscia*, it occurs only in some species. In general, this trait tends to be reduced in small species.

*Promesonotal connection*. The connection between the first mesosomal notum, the pronotum, and the rest of the mesosoma is variously developed in ants. The pronotum is dorsally adjacent to the mesonotum and laterally to the mesopleuron. The connection can be fully articulated as in most Formicinae where a well-developed Pronotomesopleural suture is present, or rigidly fused as in the Myrmicinae where the entire mesosoma forms a single rigid block and usually there is no trace of Pronotomesopleural suture dorsally. Among the dorylines both of the above mentioned conditions can be found, along with intergradations. A completely unfused and mobile connection is present only in certain *Leptanilloides*, while in *Dorylus*, for example, a conspicuous Pronotomesopleural suture is present but the connection is not mobile. In others, only lateral portions of the Pronotomesopleural suture are unfused (see next character below) or, as is the case in *Parasyscia*, the connection is fully fused with no trace of Pronotomesopleural suture.

*Pronotomesopleural suture*. In the Dorylinae, the promesonotum and mesopleuron are often linked by a Pronotomesopleural suture (sometimes termed the 'promesopleural' Pronotomesopleural suture). This character is linked to the preceding one but it is treated separately because in many genera the Pronotomesopleural suture may be completely fused dorsally, at the same time being unfused laterally on the mesosoma (Figure 12A). A completely fused pronotomesopleural Pronotomesopleural suture is characteristic of *Lividopone*, *Parasyscia*, and *Zasphinctus*. In the army ants the lateral wall of the mesosoma may be dorsoventrally compressed around this area. As a result, the Pronotomesopleural suture is generally short, directed more posteriorly rather than dorsally as in other dorylines, or entirely fused.

*Mesometapleural groove*. Directly posterior to the pronotomesopleural Pronotomesopleural suture (or the area where the Pronotomesopleural suture would be found) is the mesopleuron. This area can be delimited posteriorly from the succeeding sclerite, the metapleuron, by a groove (Figure 12A) or it can be elevated relative to the metapleuron so that a cuticular ridge is formed at the boundary. This character is somewhat difficult to quantify but most dorylines show a division between the meso- and metapleura. The separation tends to be least pronounced in *Aenictus* and most New World army ants.

*Transverse groove dividing mesopleuron*. The mesopleuron can be undivided or separated into two parts by a Pronotomesopleural suture or a cuticular ridge (Figure 2). If divided, the upper part is usually referred to as anepisternum and the lower as the katepisternum. This is another character that is rather variable in the dorylines, often within a genus. This division is generally absent from *Aenictus* and New World army ants.

*Concavity surrounding pleural endophragmal pit*. As explained in the discussion of the internal mesosomal structure above, an endophragmal pit is an impression in the cuticle that corresponds to invaginations of the cuticle. An examination of the pit itself often requires scanning electron microscope but the pit can be surrounded by a variously pronounced concavity of the cuticle, making it more easily discernable. When visible, the concavity is usually placed some distance anterior to and/or below the propodeal spiracle.

*Margination of various body segments*. This category encompasses characters that include dorsolateral margination of the mesosoma, petiole, or other segments. Several genera have some form of margination at the junction of the lateral and dorsal faces of the mesosoma or above the petiolar spiracle. The most characteristic margination of dorsolateral corners of the body is present in *Lioponera*, where it can range from being confined to the anterior half of abdominal segment II (petiole) to well-defined margins present across the posterior half of the head, most of mesosoma, posteriorly to abdominal segment IV (Figure 2).

*Metanotal depression or groove on mesosoma*. Dorsally the mesosoma may possess a groove or depression marking the division between the thorax and the propodeum (which is anatomically the first abdominal segment). Most dorylines have no such groove, but in *Aenictus* and New World army ants this distinction is usually pronounced (Figures 11, 14A).

*Propodeal spiracle position*. The position of propodeal spiracle on the lateral wall of the mesosoma can serve as a good character distinguishing army ants from other dorylines. When inspected in lateral view, the spiracle opening is almost always at or below the midheight of the mesosoma in the genera that are not considered army ants (Figure 12A) and well above in the army ants (i.e. in *Aenictogiton*, *Aenictus*, *Cheliomyrmex*, *Dorylus*, *Eciton*, *Labidus*, *Neivamyrmex*, or *Nomamyrmex*; Figure 20A). Rarely the spiracle is positioned slightly above the midheight in non-army ant dorylines but then the pygidium is usually armed with numerous peg-like setae (see below).

*Shape and margination of propodeal declivity*. The propodeum has a sloping or vertical posterior face that can be variously shaped and dorsally immarginate or with a distinct margin, bound by a carina. The most common shape of the doryline propodeum when viewed from behind is approximately rectangular. However, in *Aenictus* a more triangular shape is common. The dorsal margination appears to be more common in certain genera then in others, although there is often variability within genus.

*Metapleural gland bulla*. The metapleural gland is positioned at the posterior end of the metapleuron, on the lateral mesosoma directly above where the hind coxa articulates. The gland opens through the cuticle and has a chamber, or bulla, situated below the opening. The metapleural gland orifice is further discussed above under diagnostic characteristics. In the descriptions I indicate whether the gland bulla is visible through (Figure 5A) or obscured by the cuticle (Figure 16A).

*Propodeal lobes*. Propodeal lobes are projections of the cuticle on the propodeum, arising immediately lateral to the propodeal foramen, the opening in the mesosoma where abdominal segment II (petiole) articulates. These lobes are variously developed in the Dorylinae. They are completely absent in *Aenictogiton*, *Dorylus*, and *Leptanilloides* and absent or short in the *Eciton* genus-group. In other genera the lobes are well-developed and visible laterally as semicircular extensions of the propodeum projecting past the metapleural gland bulla (Figure 2).

*Position of helcium*. Two major portions can be distinguished in each abdominal segment starting with segment II (the petiole; the propodeum corresponds to segment I): presclerites and postsclerites. Presclerites form the portion that articulates with the preceding segment. Postsclerites constitute the part of the segment that is always exposed without dissection or extension of gastral sclerites. The helcium comprises the presclerites of abdominal segment III, that is its anterior portion that articulates with the petiole. The helcium can be positioned at or above the tergosternal Pronotomesopleural suture of segment III. The position of helcium can also be described relative to the midheight of postsclerites of abdominal segment III in lateral view. Axial helcium means positioned at the midheight, infraaxial below, and supraaxial above. The helcium can thus be positioned at the tergosternal Pronotomesopleural suture and at the same time be considered infraaxial if the Pronotomesopleural suture occurs below the midheight of the segment. In most dorylines the helcium is placed at the Pronotomesopleural suture and more or less axially. The most obvious departure from this state is when the helcium is in a supraaxial position and above the Pronotomesopleural suture (Figure 35A). This means that there is a narrow posterior face of the petiolar and narrow anterior face of the abdominal posttergite III. A supraaxial helcium also tends to have larger circumference than an axial helcium. This condition can be seen in *Acanthostichus*, *Cerapachys*, *Lividopone*, and at least one *Yunodorylus*. In some *Leptanilloides* the helcium is positioned supraaxially because abdominal sternite III is very large but in this case there is still a defined posterior face to the petiole and anterior face of the sternite III. For brevity, a helcium with small circumference is referred to as ‘narrow’ and a helcium with large circumference is referred to as ‘broad’ in the diagnoses.

*Prora*. Prora is used to describe a protrusion on the anterior face of abdominal poststernite III, below the helcium. In the dorylines this feature is variously developed, as a simple angle not delimited by carinae (Figure 7A) through a flat surface bounded by a U-shaped carina, to a broad lip-like structure (Figure 35A). This character tends to vary within genera.

**Figure 6.**
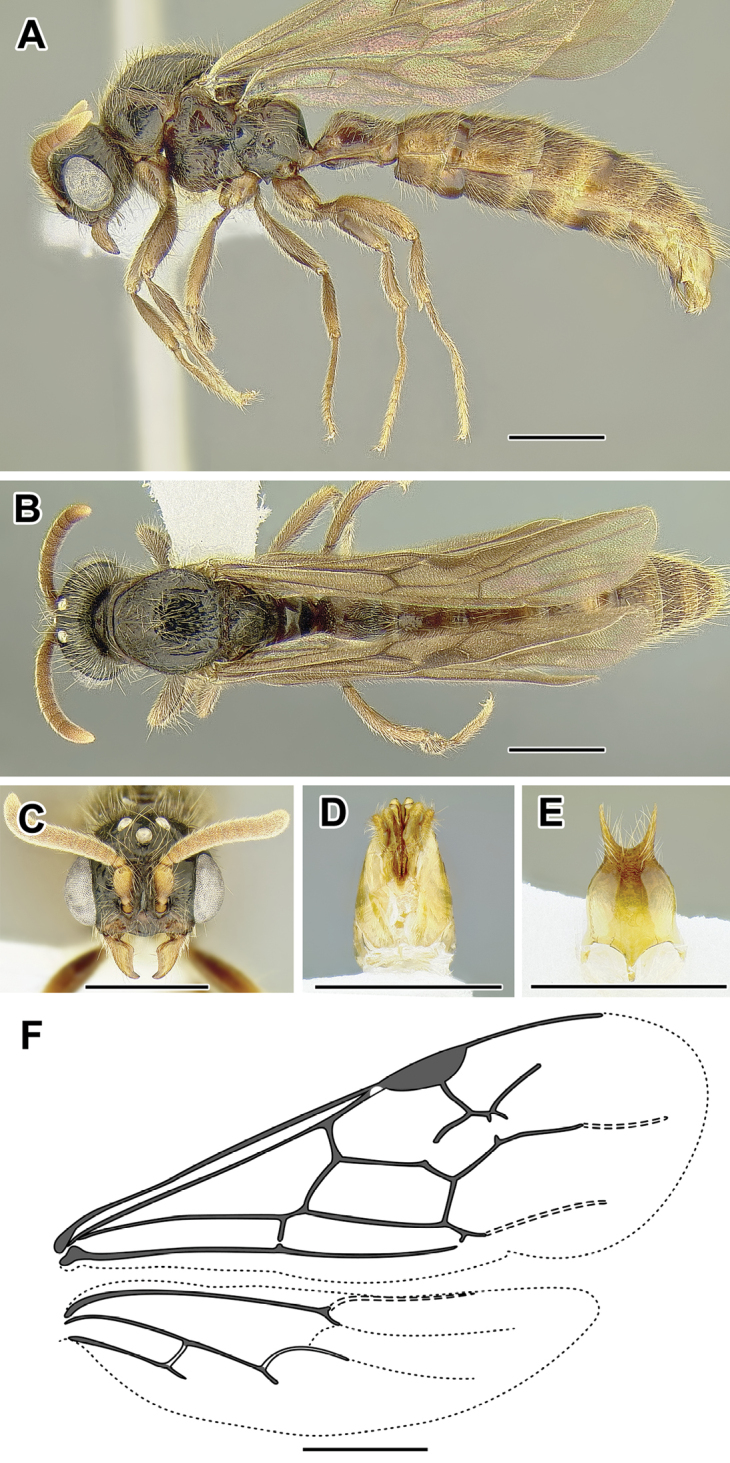
**A–F** Male of *Acanthostichus* sp. (CASENT0731087) **A** Body in lateral view **B** Body in dorsal view **C** Head in full-face view **D** Genital capsule in ventral view **E** Abdominal segment IX (subgenital plate) **F** Wing venation. Scale bar equals 1.0 mm.

**Figure 7.**
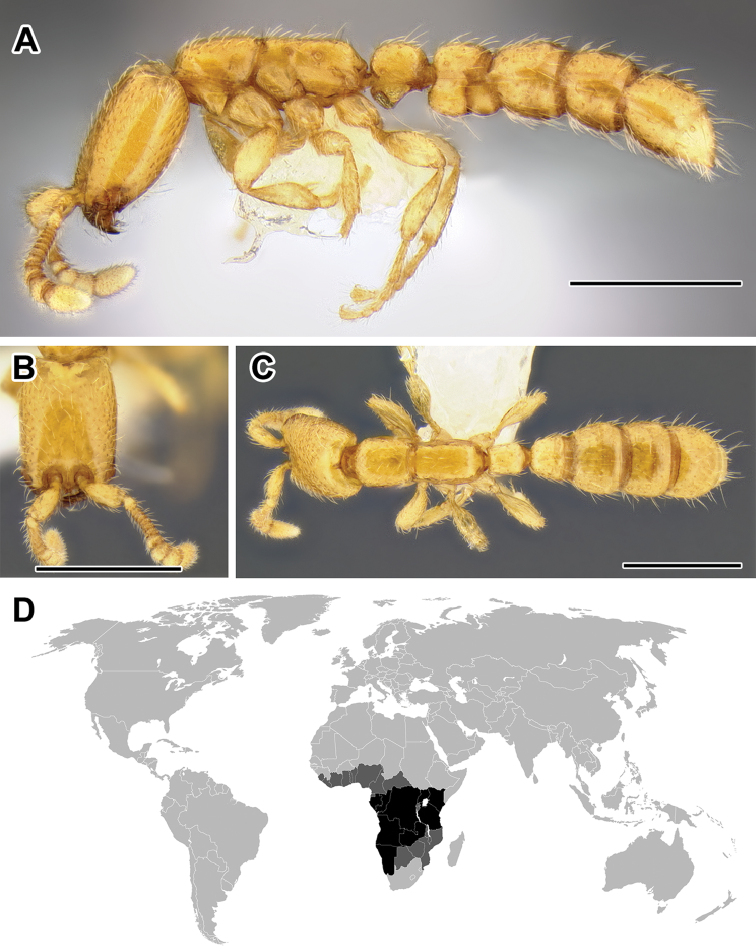
**A–C** Worker of *Aenictogiton* sp. (CASENT0317577) **A** Body in lateral view **B** Head in full-face view **C** Body in dorsal view **D** World distribution of *Aenictogiton* (black: present, dark grey: likely present). Scale bar equals 0.5 mm

*Spiracle openings of abdominal segments IV–VI*. Spiracles are visible on the gaster in the Dorylinae without dissection and their orifices can be variously shaped, from round to narrow and slit-shaped. Slit-shaped openings are rare and are found only in *Eciton*, *Nomamyrmex*, and some *Neivamyrmex*. Sometimes the spiracle opening on segment IV is more round than those of succeeding segments.

*Girdling constriction of segment IV*. The boundary between pre- and postsclerites of segment IV can be inconspicuous (Figure 58A) or marked with a constriction, also known as a cinctus (Figure 2). A simple transition is present only in some *Yunodorylus* while in all other genera there is a constriction. Because in specimens where the gastral sclerites are not expanded the constriction is near the articulation with the preceding segment it appears as if there is a constriction between the segments themselves.

*Sculpturing of cinctus of abdominal segment IV*. When the girdling constriction between pre- and postsclerites is present, it can take different forms. It can be a simple dip or a defined trench or gutter-like concavity. The constriction can also be smooth or sculptured, most often cross-ribbed with short lines (Figure 2). This character is sometimes variable within a genus, but in general the cross-ribbed cinctus is common among non-army ant dorylines and does not occur in true army ants.

*Relative size of abdominal segment IV*. This abdominal segment can form the bulk of the metasoma in certain species (Figure 9A) or, alternatively, be similar in size to segments III or V (Figure 7A). A large segment IV is associated with species where the waist is two segmented, or in other words where the segment III is well-differentiated from the rest of the metasoma. This condition is thus observed in all *Aenictus*, *Eciton*, *Labidus*, *Neivamyrmex*, *Nomamyrmex*, and *Ooceraea*. Other doryline genera have a generally smaller abdominal segment IV. *Eburopone* and *Syscia* present an intermediate condition where this abdominal segment is the largest but not always conspicuously so.

**Figure 8.**
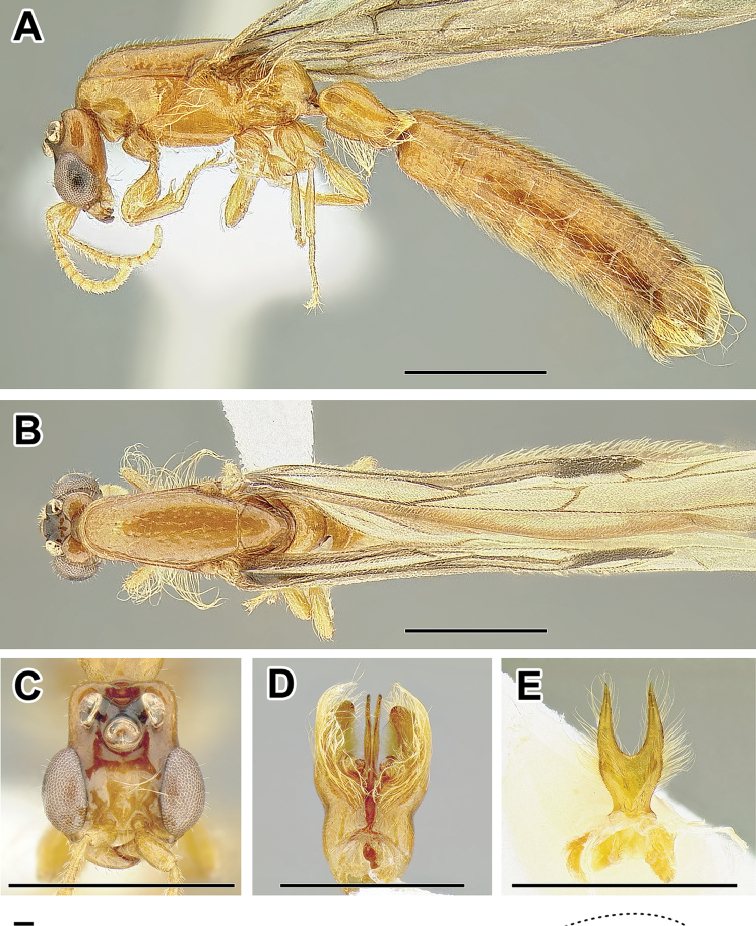
**A–F** Male of *Aenictogiton* sp. (CASENT0731199) **A** Body in lateral view **B** Body in dorsal view **C** Head in full-face view **D** Genital capsule in ventral view **E** Abdominal segment IX (subgenital plate) **F** Wing venation. Scale bar equals 1.0 mm.

**Figure 9.**
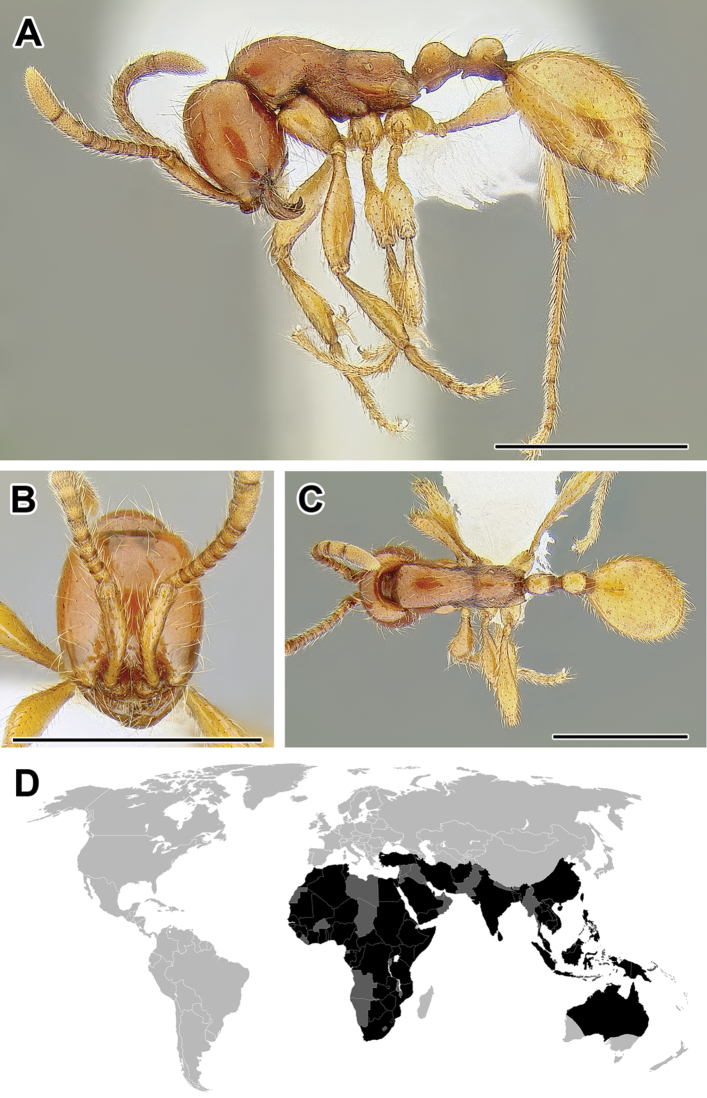
**A–C** Worker of *Aenictus* sp. (CASENT0249272) **A** Body in lateral view **B** Head in full-face view **C** Body in dorsal view **D** World distribution of *Aenictus* (black: present, dark grey: likely present). Scale bar equals 1.0 mm.

*Anterior folding of abdominal tergite IV*. In most dorylines the tergosternal Pronotomesopleural suture runs across the midheight of the segment in lateral view such that both poststernite and posttergite are visible across the entire length of the segment. In *Syscia*, however, the anterior portion of the Pronotomesopleural suture drops down in lateral view resulting in only the tergite being visible posterior to the cinctus (Figure 53A). This means that in ventral view the tergite can also be seen towards the anterior end of the postsclerite.

*Girdling constrictions on tergites or sternites V and VI*. Although most dorylines possess some form of constriction on abdominal segment IV, such constrictions can be also present posterior to that segment (Figure 51A). These can be conspicuous on both tergal and sternal portions and result in the characteristic metasoma of *Aenictogiton*, *Eusphinctus*, *Sphinctomyrmex*, and *Zasphinctus*. In other genera, the constrictions are less conspicuous but nevertheless present, as in *Dorylus* (Figure 20A) and certain *Leptanilloides*. The constrictions can also be present only on the sternites, as in some *Acanthostichus* or *Cylindromyrmex*.

*Size and shape of pygidium*. A modified pygidium, or tergite of abdominal segment VII, is likely a synapomorphy of the Dorylinae. See diagnostic characters above for a discussion.

*Hypopygium*. The hypopygium is the sternal portion of abdominal segment VII of workers. Occasionally, as in some species of *Syscia* and *Ooceraea*, the hypopygium can be lined with specialized thick setae similar to those on the pygidium.

*Number and shape of tibial spurs*. One or two multicellular articulated projections, known as spurs, may occur at the apex of tibiae. The configuration of spurs on the middle and hind tibiae can be useful in identification of doryline genera. There can be two spurs on both middle and hind tibiae, which is the condition seen in *Aenictus*, *Chrysapace*, *Cylindromyrmex*, and *Yunodorylus*, as well as at least one *Leptanilloides* species. More commonly, there is one spur on both middle and hind tibiae. In a few genera there are no spurs on middle tibiae but one spur is present on the apex of hind tibia. These include *Simopone*, *Tanipone*, and *Vicinopone*. The shape of the spurs may also vary, from spurs that have a well-defined comb-like or pectinate margin, through barbulate surface, to simple seta- or spike-like spurs.

*Form of hind basitarsus*. In most dorylines the first segment of hind tarsus, the basitarsus, is circular in cross-section and as wide basally as distally. *Syscia* is an exception where the basitarsus is oval in cross-section, gradually widening towards the apex (Figure 53A).

*Posterior flange of hind coxa*. The joint between the coxa and femur is marked by a pronounced concavity in the former. Just posterior of that concavity, a thin lamella can be found in *Lioponera* (Figure 2). The lamella is usually broadest distally, toward the apex of coxa. This character is variously developed but will serve to distinguish most *Lioponera* species from other dorylines.

*Metatibial gland*. See the discussion under diagnostic character 15 above.

*Metabasitarsal gland*. See discussion under diagnostic character 15 above.

*Hind pretarsal claws*. The pretarsal claws can be simple or armed with a tooth in certain lineages. This feature is generally consistent within a genus and thus a reliable character for identification. Pretarsal claws are armed with a tooth at least on the hind leg in some *Cerapachys*, all *Chrysapace*, *Simopone*, *Tanipone*, *Vicinopone*, and the *Eciton* genus-group species except *Neivamyrmex*.

### Characters used to describe male morphology

Because several features of male morphology are similar to those of the worker, I focus on the characters and character systems unique to the male, including flight sclerites, genitalia, and wing venation.

*Number of antennal segments*. This character varies as in the worker caste, but the plesiomorphic condition is 13 segments (Figure 52A) and a reduction to 12 is less common, occurring only in *Eusphinctus* (Figure 27A), *Ooceraea*, *Simopone*, and *Syscia*. Many *Ooceraea* have 11-segmented antennae. Some *Acanthostichus* and *Zasphinctus* also show a reduction in the number of antennal segments to 12.

*Notauli*. The notauli are grooves on the mesoscutum, or the anterior plate of the male mesonotum. When present, they are usually well-developed as V- or Y-shaped grooves converging towards the posterior (Figures 3, 27A). The notauli appear to be consistently absent from all army ant genera (Figure 8A) as well as *Tanipone* and *Yunodorylus*. Genera that are polymorphic with regard to the presence or absence of notauli include *Acanthostichus*, *Cylindromyrmex*, *Eburopone*, *Lioponera*, *Parasyscia*, and *Zasphinctus*.

*Metapleural gland opening*. Unlike doryline workers, where the orifice of the metapleural gland is always present, many males have lost this feature. Even in the case of males possessing a concavity or orifice in the cuticle where the gland would be located, it is not clear whether this structure is connected to functioning glandular tissue. Because this character appears to be of some diagnostic value, however, I coded its presence and absence in the doryline males without any assumptions on gland activity.

*Propodeal lobes*. See discussion of this character under worker morphology above. The lobes are well-developed in males of non-army ant dorylines, where they seem to nearly always project beyond the dorsal margin of propodeal foramen. They are somewhat better developed in many *Eciton* genus-group males relative to the worker but in these genera the dorsal margin of propodeal foramen projects about as far posteriorly as the propodeal lobes.

*Shape of abdominal sternite VII*. The abdominal sternite VII is often a simple sclerite with a flat surface and no protrusions. In most *Ooceraea*, however, this sternite is modified to be notched, often with extensions on either side of the sclerite supporting thick setation, sometimes forming a brush.

*Shape of abdominal sternite IX*. See discussion under male diagnostic character 1.

*Male genitalia*. In the descriptions I provide a very general account of the genital morphology. The terminology I use here follows Boudinot (2015).

*Cupula*. The basalmost sclerites form the cupula, also known as the basal ring. In most general terms, the doryline cupula can be short or long relative to the length of the genital capsule. The cupula is best developed in the *Eciton* genus-group, where it is characteristically nearing or exceeding the length of the rest of the genital capsule. In most non-army ant dorylines the cupula is shorter than half the length of the rest of genital capsule but nevertheless conspicuous. A few genera have a cupula that is very short, a narrow ring of cuticle at the base of genital capsule. These include *Aenictus*, *Dorylus*, *Leptanilloides*, and *Yunodorylus*. Apparent cupula length can also vary depending on whether viewed from above or ventrally.

**Figure 10.**
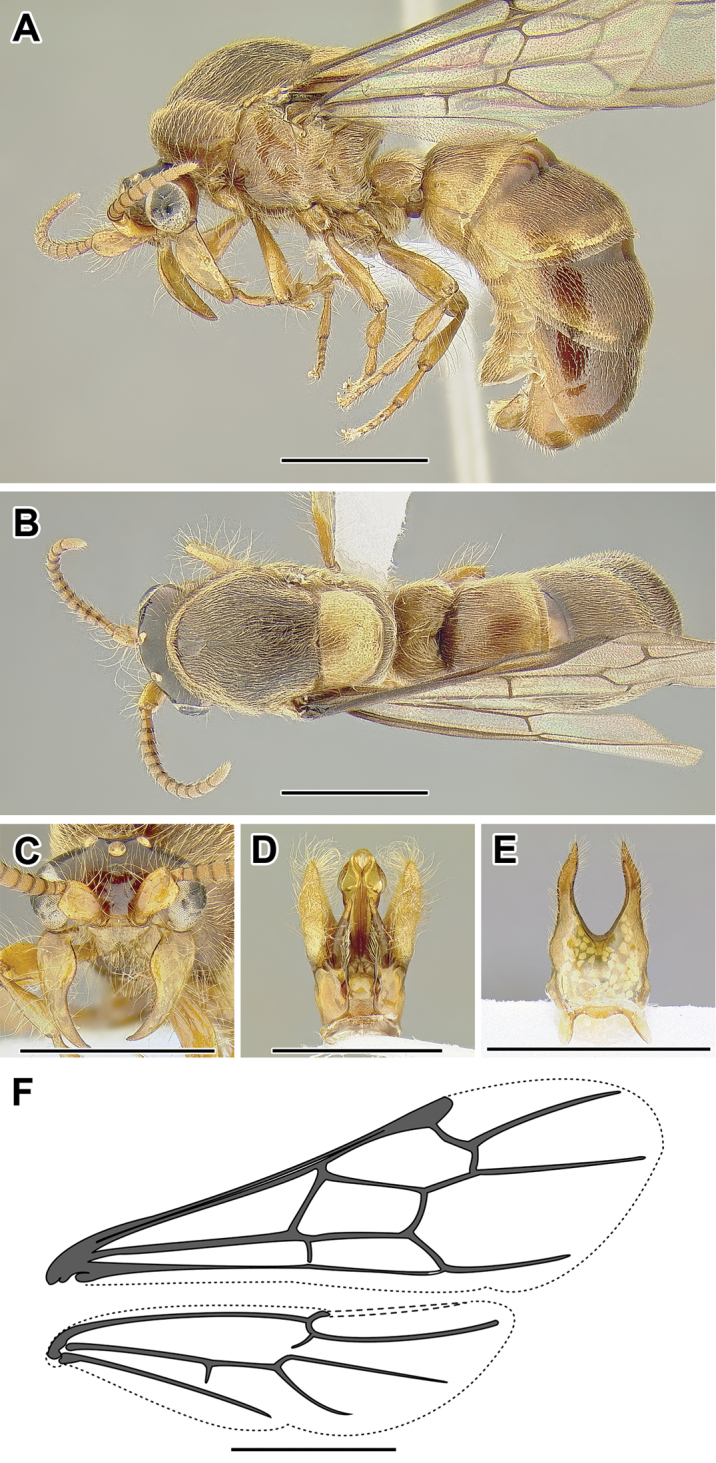
**A–F** Male of *Aenictus* sp. (CASENT0731090) **A** Body in lateral view **B** Body in dorsal view **C** Head in full-face view **D** Genital capsule in ventral view **E** Abdominal segment IX (subgenital plate) **F** Wing venation. Scale bar equals 2.0 mm.

*Basimere and telomere*. The outermost valve of the genital capsule is the paramere. The paramere can be divided into the basal portion called the basimere and the distal portion the telomere. In most dorylines these two portions are broadly connected but the New World army ants are an exception where the telomeres are very narrowly connected to dome-like basimeres (Figure 15D). In other dorylines that connection is broad and either marked by a sulcus or not. Furthermore, the left and right basimere arms are most often abutting ventrally although occasionally they can be separated, most conspicuously in some *Aenictus* (Figure 10D). The distal portion of the basimere, the telomere, can be variously developed, straight or hooked, gradually tapering to a point at the apex or broad distally.

*Volsella*. The second outermost valve is the volsella. Similarly to the telomere, volsella can be of variable shape.

*Penisvalvae*. The innermost valves, the penisvalvae, aedeagal valves, or collectively the aedeagus, are similarly variously shaped and can be apically rounded, grossly expanded, and straight or hooked. The setation of the apex of penisvalvae is a reliable character distinguishing otherwise similar males of New World army ant genera *Cheliomyrmex*, *Labidus*, and *Nomamyrmex*, which possess hairs, from *Eciton* and *Neivamyrmex* with hairless penisvalvae. Given much intrageneric variability of the genital capsule, and especially its inner valves, it is likely that this revision does not provide a complete description of its morphological diversity. Statements about male genitalia will certainly be revised and refined when additional males are examined.

*Tegula*. The tegula is a small, dome- or strap-shaped sclerite that covers the base of the fore wing. In the Dorylinae the tegula varies in shape from broadly oval to thin and strap-shaped but it is generally conspicuous (Figure 19A–B) except for *Leptanilloides* in which it is extremely reduced or completely absent (Figure 31B), i.e. not easily discernable except under high magnification in the largest of species.

*Fore wing venation*. Wing venation often varies considerably within a genus. However, because it is obvious and because a certain pattern can be characteristic for the vast majority of species within a given genus despite occasional reductions, this is a character system valuable for identification (Figure 4). The fore wing venation of a doryline ant can include one to eight closed cells. When referring to presence or absence of veins in the descriptions, a vein is considered present regardless of whether it is tubular, nebulous, or spectral (Mason 1986).

*Costal vein (C)*. The vein on the leading margin of the fore wing anterior to the pterostigma is called the costal vein. This vein is an important character for genus identification although its presence may be challenging to ascertain when the leading wing margin is folded onto itself. The costal vein is often present (Figure 6F) but conspicuously absent from *Lioponera* (Figure 33A), *Lividopone*, *Parasyscia*, *Syscia*, and *Zasphinctus*. *Ooceraea* appears to be exceptional in being polymorphic for the presence of the costal vein. The pterostigma is a pigmented area that in the Dorylinae can be either a conspicuous oval at the leading edge of the fore wing or only a narrow extension in line with the longitudinal veins running along that edge. Most dorylines possess the former state where pterostigma is well-differentiated from surrounding venation. *Dorylus* and *Eciton* genus-group are exceptions as these genera have a more or less narrow pterostigma.

*Radial vein (R)*. In ants, this vein is considered to be fused with subcostal vein and radial sector (Sc+R+Rs) proximally, bifurcating into Sc+R and radial sector (R) before reaching pterostigma. The free abscissae R·f1–2 are fused with the pterostigma and R·f3 projects distally of the pterostigma on the leading edge of the fore wing. In the Dorylinae, R·f3 is generally associated with overall well-developed venation and is found in all New World army ant genera. Among non-army dorylines, it can be found in *Acanthostichus*, *Cerapachys*, *Chrysapace*, *Cylindromyrmex*, most *Eburopone*, *Neocerapachys*, *Procerapachys*, and *Yunodorylus*.

*Radial sector (Rs)*. Past the separation from Sc+R, the radial sector continues posterior to the pterostigma, first as a usually short free abscissa Rs·f1, then merging with median vein (M) and continuing fused (Rs+M) for some time, followed by the free abscissae Rs·f2–5 that, when present, constitute the next longitudinal vein system posterior to the pterostigma. Rs·f5 may distally connect with R·f3 to close a marginal cell. When Rs is present distally to Rs+M, it connects to the pterostigma via the second radial-radial sector cross-vein (2r-rs). Various levels of reduction of the radial sector are found within the Dorylinae. The most complete development is found in *Eciton* genus-group, *Cerapachys*, *Chrysapace*, *Cylindromyrmex*, *Procerapachys*, *Sphinctomyrmex*, and some *Neocerapachys* and *Yunodorylus*. In these ants the free abscissae of the radial sector continue to close the marginal vein by joining R·f3 at the wing margin, in some species interrupted only at the connection with Rs+M. In other genera such as *Parasyscia*, *Lividopone*, or *Zasphinctus* radial sector abscissae Rs·f2–3 connect to Rs+M but radial abscissa R·f3 is absent and Rs·f4–5 do not close the marginal cell. In *Aenictogiton* and *Simopone* the radial sector is further reduced, interrupted near Rs+M junction and not reaching wing margin. In *Lioponera*, *Eburopone*, and some *Ooceraea* and *Syscia* the abscissae Rs·f2–3 are absent and Rs·f4–5 together with 2r-rs form a ‘free stigmal vein’ that does not reach wing margin. More reduction is found in various genera where smaller species sometime lost all radial sector veins past Rs+M.

*Median vein (M)*. Further away from the leading wing margin is the median vein, proximally fused with cubital vein (M+Cu), following separation continuing as a free abscissa M·f1 before joining with radial sector to form Rs+M. In the Dorylinae the next free abscissa (M·f2) may be separated from Rs+M if Rs·f2–3 or continuous with Rs+M in the absence of radial sector. If median vein is present past the junction with the radial sector, further free abscissae M·f3 and M·f4 can be differentiated in the presence of second radial sector-median cross-vein (2rs-m) and M·f4 may extend all the way to the distal wing margin. Various reductions are possible from this basic pattern. The median vein is highly variable, from the best developed in the *Eciton* genus-group, where it almost always reaches wing margin as a tubular vein, through a state where nebulous or spectral free abscissae M·f3–4 are disconnected from other veins, to entirely absent past Rs+M as in certain *Leptanilloides*.

*Cubital vein (Cu)*. Proximally the cubital vein is fused with median vein (M+Cu) and can have up to three free abscissae, Cu·f1 through Cu·f3. The first median-cubital cross-vein (1m-cu) may connect the cubital vein to the median vein between Cu·f2 and Cu·f3. Cu·f3 can further distally branch into as many as three branches, Cu1–3. Cu1 is often present, even in species with venation otherwise reduced in the radial sector and the median vein. Cu1 is the long branch running towards the distal wing margin. Cu2 is short and sometimes connects to the anal vein (A). When present, Cu3 is always a short stub directed towards the posterior wing margin. In dorylines it is found only in the largest of males, in the New World army ants and some *Dorylus*.

*Anal vein (A)*. The anal vein is the longitudinal vein running near the posterior wing margin. In dorylines it consists of at least one free abscissa fused to or terminating near cubital-anal cross-vein (cu-a), a connection to the cubital vein, and often two abscissae are present (A·f1–2) if continuing past cu-a. The position of cu-a relative to the branching of M·f1 can help distinguish a male of the *Eciton* genus-group from an Old World army ant: in the former M·f1 arises much closer to the base of the wing than cu-a while in *Aenictogiton*, *Aenictus*, or *Dorylus* M·f1 arises either distally to cu-a, directly below it, or only slightly proximally.

*Hind wing venation*. Veins in the hind wing follow a similar pattern to that found in the fore wing but there is no pterostigma and the venation is simplified (Figure 4). In the dorylinae the hind wing venation can from zero up to three closed cells. At least vein Sc+R+Rs appears to be present in all species.

### Characters used to describe gyne morphology

Because the morphology of the majority of doryline gynes is much like the worker, this revision does not describe gynes in detail. Instead, a general morphology is indicated, including how gynes differ from the worker, whether the known forms are alate, ergatoid (wingless and worker-like), or dichthadiigyne (see below), and references to more detailed descriptions are provided where available. The gyne morphology can be variable within a genus or even within species where intercastes, or individuals with morphology intermediate between workers and gynes, are known in addition to fully-developed gynes.

As mentioned above, the doryline gynes can be classified as alate, worker-like, or ‘dichthadiigyne’ or ‘subdichthadiigyne’, although a gradation of intermediates between all these morphologies is also observed among the doryline species. Fully alate gynes possess the usual complement of flight-associated sclerites, relatively large eyes and ocelli, and are apparently capable of flight. Alate gyne material available is often scarce and inference about the presence of wings from dealated gynes is difficult because obvious mesosomal sutures and wing scar-like structures are not always indicative of presence of fully developed wings in the virgin gynes. At least one species is known to have brachypterous (short-winged) gynes, bridging the gap between fully alate and worker-like morphologies. Wingless, ergatoid gynes are very common in dorylines. They exhibit variation in how different they are from the workers, ranging from gynes essentially indistinguishable from the worker to ones that have enlarged gasters, large eyes and ocelli, and wing scar-like structures on the mesosoma. The term ‘dichthadiigyne’ (Wheeler 1908) has been used to describe the highly specialized gyne morphology characteristic of the ‘true army ants’. Dichthadiigynes are wingless but, unlike ergatoids, much different from the worker caste. They usually have falcate mandibles, are often completely blind or possess only small eyes, have enlarged gasters capable of significant distension, and possess one-segmented waist, even if the corresponding worker caste has a well-differentiated abdominal segment III (postpetiole). Gynes intermediate between ‘simple’ ergatoids and highly derived dichthadiigynes are also known and these have been sometimes termed ‘subdichthadiigynes’.

Alate or apparently alate (known only from dealated specimens) gynes are so far known in *Acanthostichus*, *Cerapachys*, *Chrysapace*, *Cylindromyrmex*, *Eburopone*, *Lioponera*, *Lividopone*, *Neocerapachys*, *Parasyscia*, *Simopone*, *Syscia*, *Vicinopone*, and *Zasphinctus*. Ergatoid gynes are found in *Cerapachys*, *Eburopone*, *Eusphinctus*, *Lioponera*, *Ooceraea*, *Parasyscia*, *Simopone*, *Sphinctomyrmex*, *Tanipone*, and *Zasphinctus*. Subdichthadiigynes or dichthadiigynes are found in *Acanthostichus*, *Leptanilloides*, *Ooceraea*, *Zasphinctus*, and ‘true army ants’ in the genera *Aenictus*, *Dorylus*, *Eciton*, *Labidus*, *Neivamyrmex*, and *Nomamyrmex*. A description of a *Yunodorylus* subdichthadiigyne is currently awaiting publication (Eguchi et al. in press). No gynes are known so far for *Aenictogiton* and *Cheliomyrmex*. Because of the apparent repeated evolution of derived wingless gynes, the dorylines could become a good system for studying gyne evolution once a more complete picture of the diversity of gyne morphologies is available.

**Figure 11.**
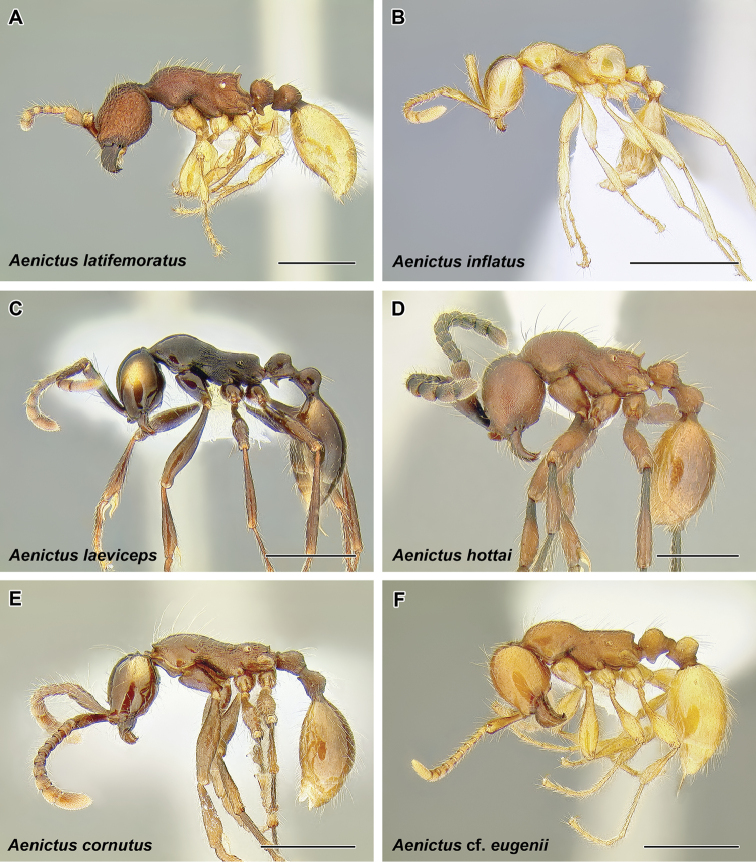
**A–F** Morphological diversity of *Aenictus*. **A** *Aenictus latifemoratus* (CASENT0249279) **B** *Aenictus inflatus* (CASENT0732111) **C** *Aenictus laeviceps* (CASENT0732112) **D** *Aenictus hottai* (CASENT0249278) **E** *Aenictus cornutus* (CASENT0249267) **F** Aenictus cf. eugenii (CASENT0249274). Scale bar equals 1.0 mm.

**Figure 12.**
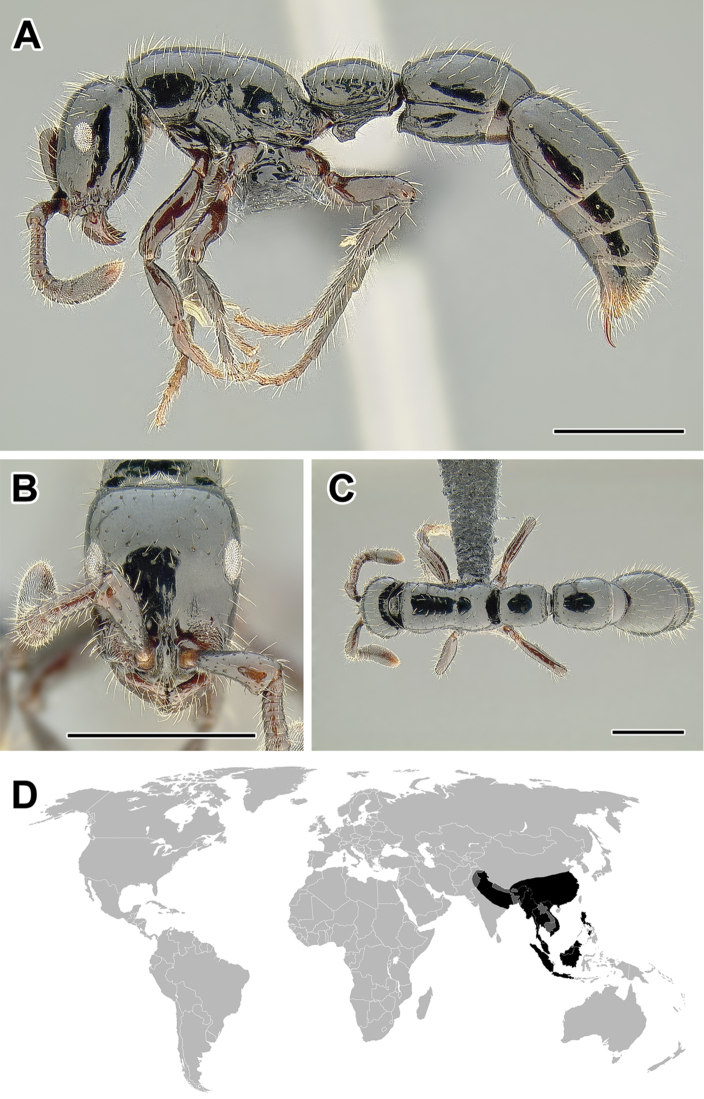
**A–C** Worker of *Cerapachys* sp. (CASENT0162338) **A** Body in lateral view **B** Head in full-face view **C** Body in dorsal view **D** World distribution of *Cerapachys* (black: present, dark grey: likely present). Scale bar equals 1.0 mm.

**Figure 13.**
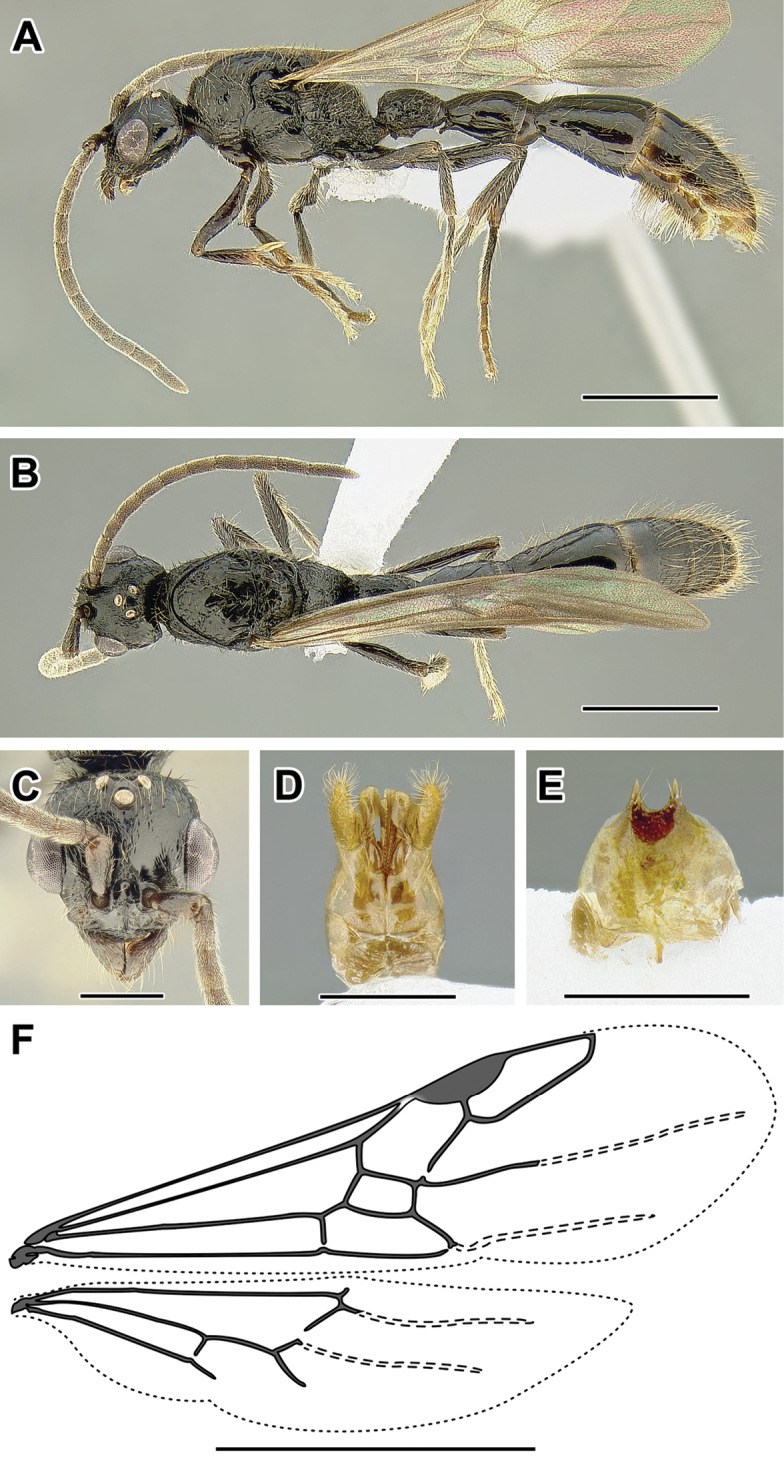
**A–F** Male of *Cerapachys antennatus* (CASENT0731091) **A** Body in lateral view **B** Body in dorsal view **C** Head in full-face view **D** Genital capsule in ventral view **E** Abdominal segment IX (subgenital plate) **F** Wing venation. Scale bar equals 2.0 mm in **A–C** and **F**, 0.5 mm in **D** and **E**.

**Figure 14.**
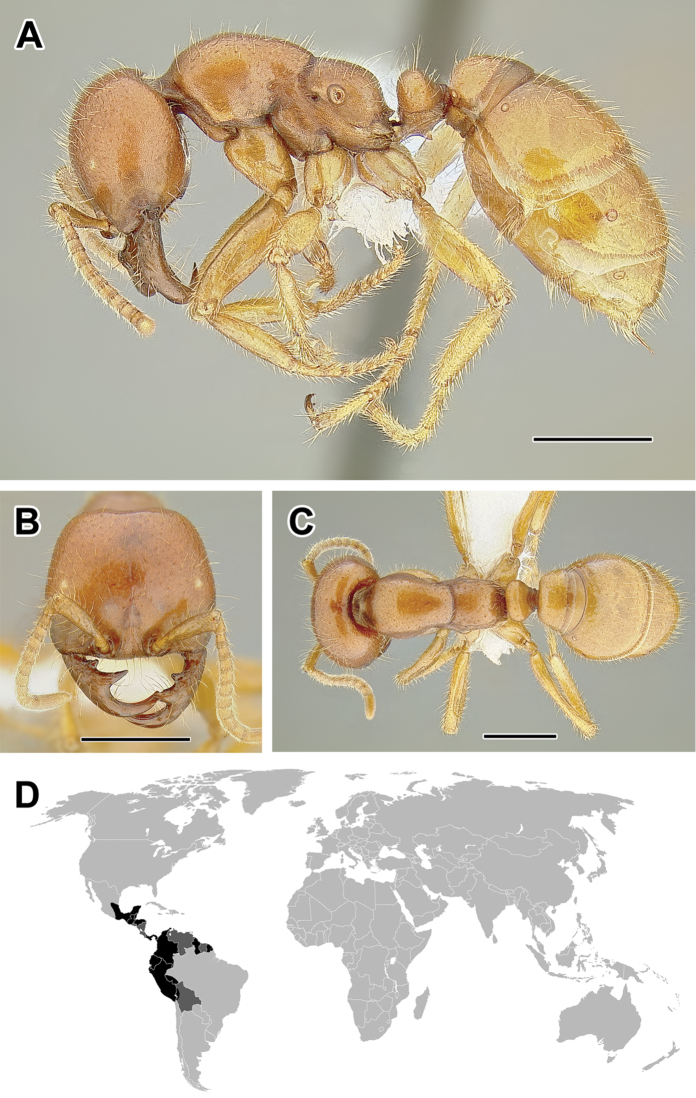
**A–C** Worker of *Cheliomyrmex morosus* (CASENT0731129) **A** Body in lateral view **B** Head in full-face view **C** Body in dorsal view **D** World distribution of *Cheliomyrmex* (black: present, dark grey: likely present). Scale bar equals 1.0 mm.

**Figure 15.**
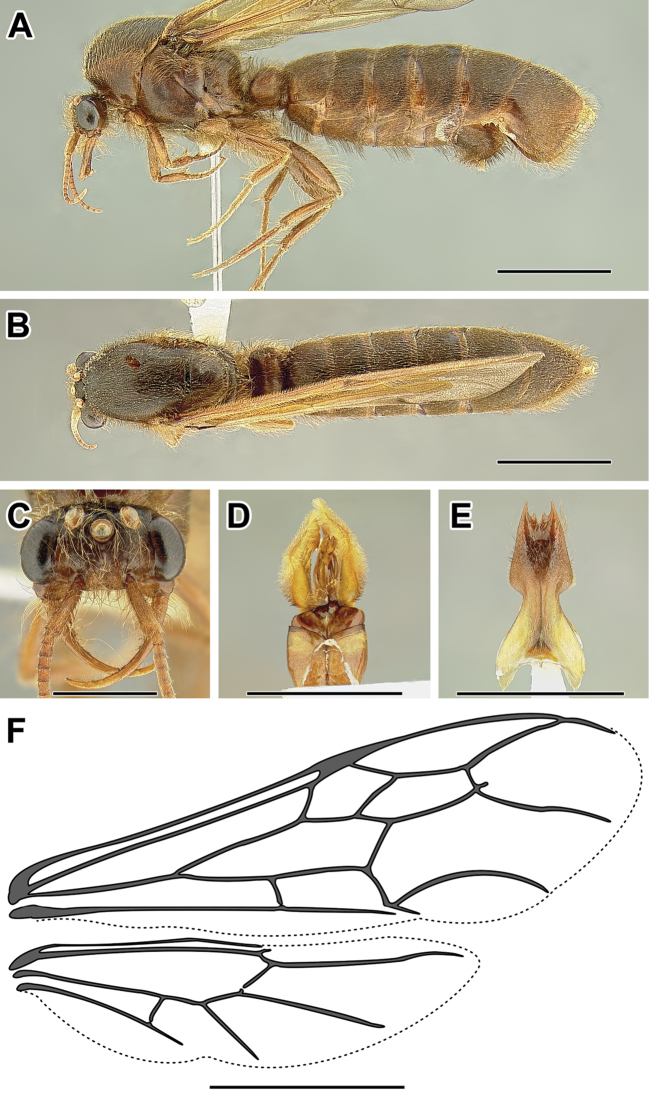
**A–F** Male of *Cheliomyrmex morosus* (CASENT0731092) **A** Body in lateral view **B** Body in dorsal view **C** Head in full-face view **D** Genital capsule in ventral view **E** Abdominal segment IX (subgenital plate) **F** Wing venation. Scale bar equals 5.0 mm in **A** and **B, D–F**, 2.0 mm in **C**.

**Figure 16.**
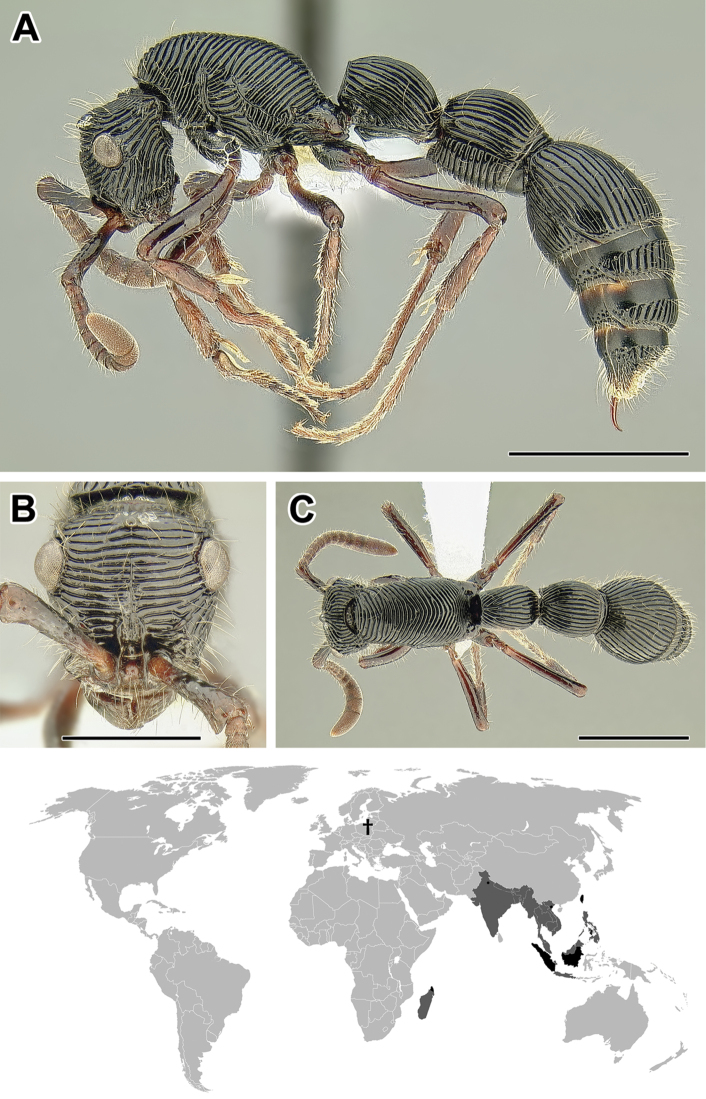
**A–C** Worker of *Chrysapace* sp. (CASENT0731133) **A** Body in lateral view **B** Head in full-face view **C** Body in dorsal view **D** World distribution of *Chrysapace* (black: present, dark grey: likely present). Scale bar equals 2.0 mm in **A** and **C**, 1.0 mm in **B**.

**Figure 17.**
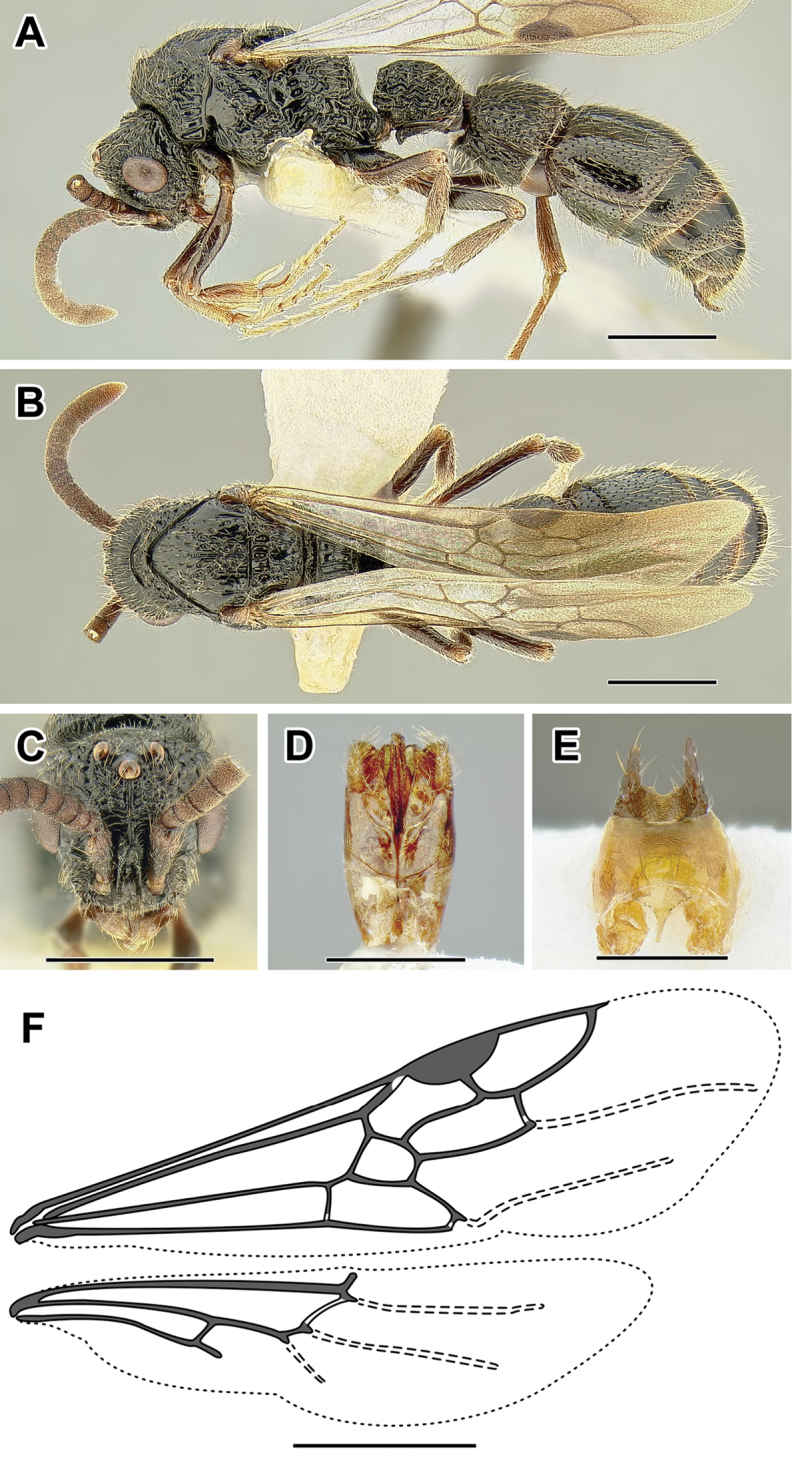
**A–F** Male of *Chrysapace* sp. (CASENT0731113) **A** Body in lateral view **B** Body in dorsal view **C** Head in full-face view **D** Genital capsule in ventral view **E** Abdominal segment IX (subgenital plate) **F** Wing venation. Scale bar equals 1.0 mm in **A–C** and **F**, 0.5 mm in **D** and **E**.

**Figure 18.**
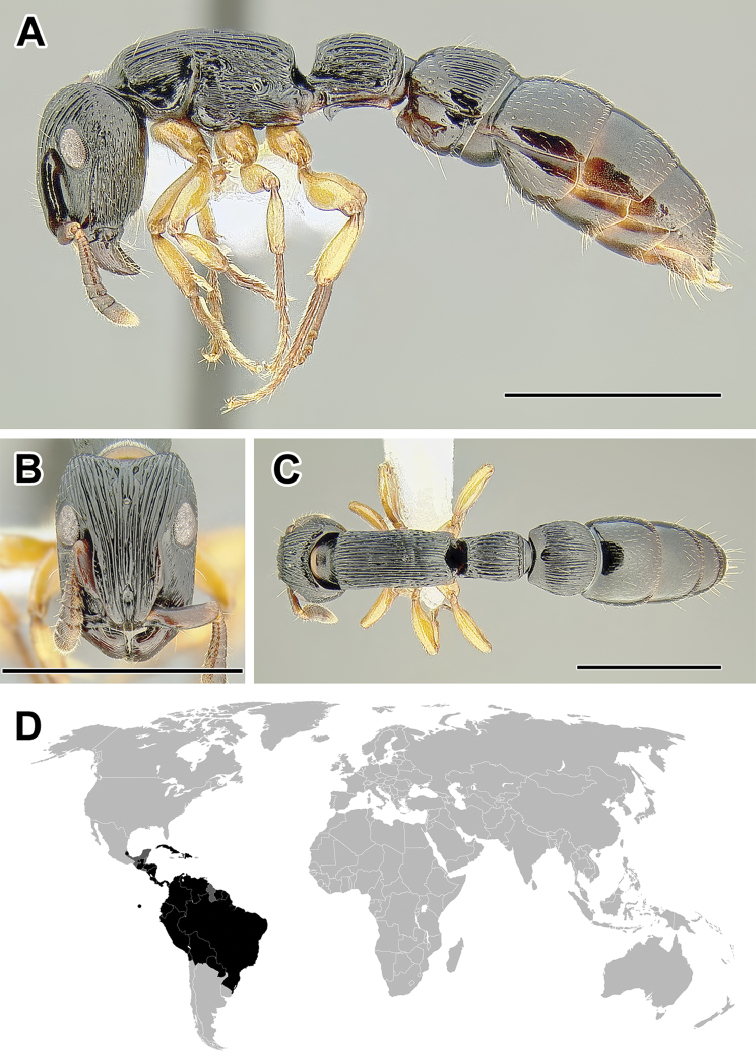
**A–C** Worker of *Cylindromyrmex brasiliensis* (CASENT0731132) **A** Body in lateral view **B** Head in full-face view **C** Body in dorsal view **D** World distribution of *Cylindromyrmex* (black: present, dark grey: likely present). Scale bar equals 2.0 mm.

**Figure 19.**
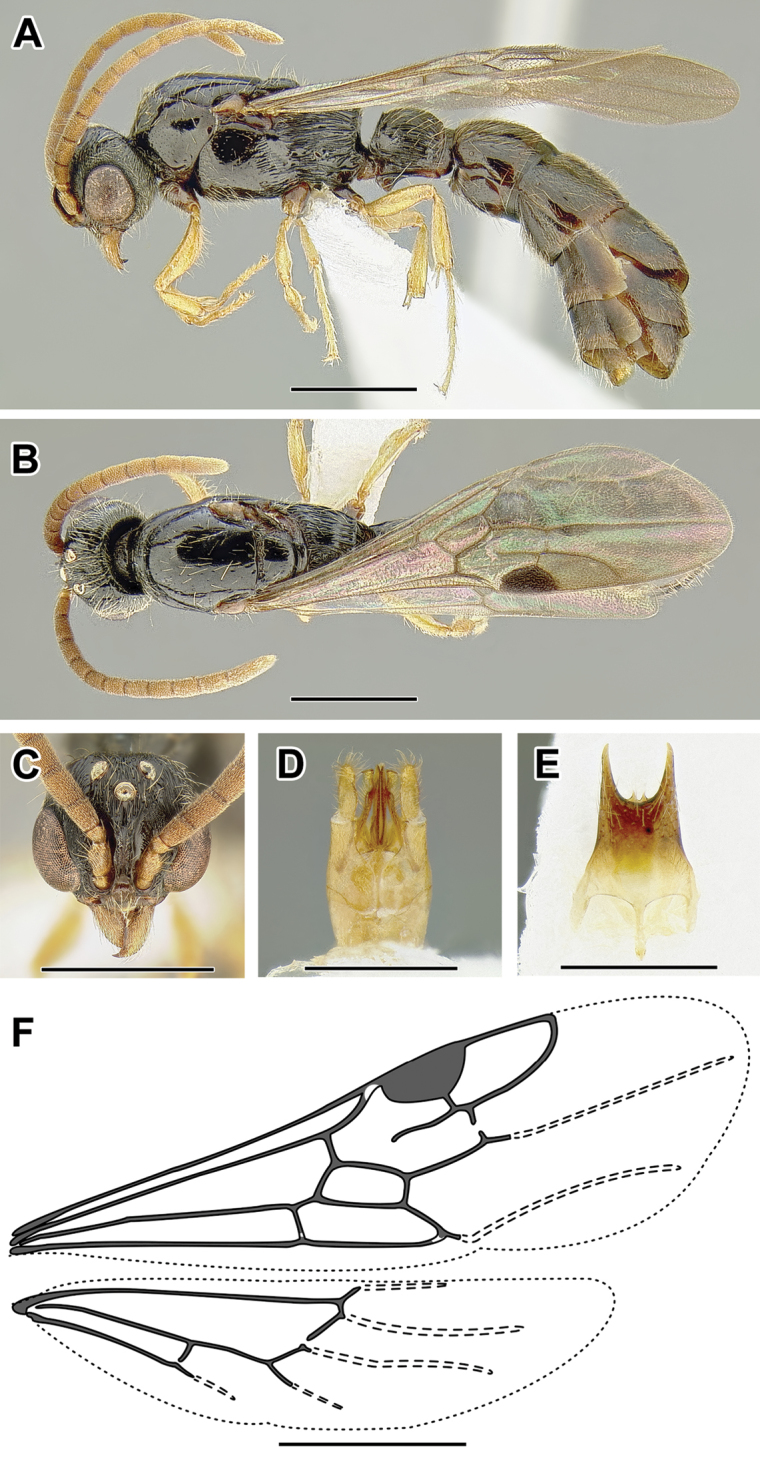
**A–F** Male of *Cylindromyrmex brevitarsus* (CASENT0731094) **A** Body in lateral view **B** Body in dorsal view **C** Head in full-face view **D** Genital capsule in ventral view **E** Abdominal segment IX (subgenital plate) **F** Wing venation. Scale bar equals 1.0 mm in **A–C** and **F**, 0.5 mm in **D** and **E**.

**Figure 20.**
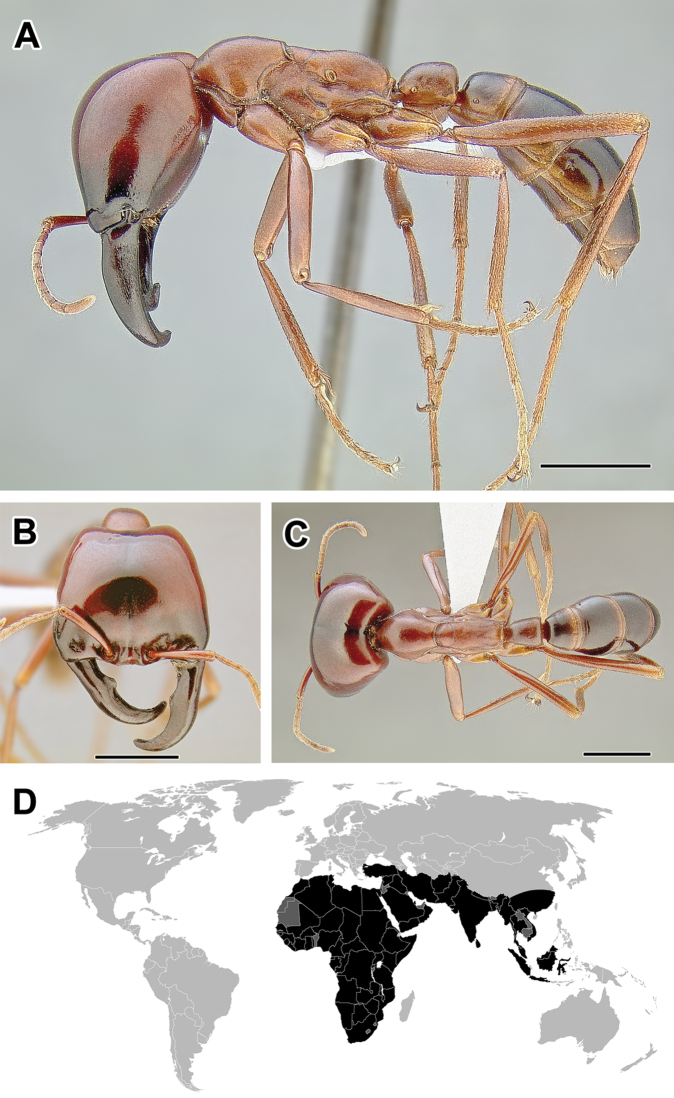
**A–C** Worker of *Dorylus nigricans terrificus* (CASENT0731192) **A** Body in lateral view **B** Head in full-face view **C** Body in dorsal view **D** World distribution of *Dorylus* (black: present, dark grey: likely present). Scale bar equals 2.0 mm.

**Figure 21.**
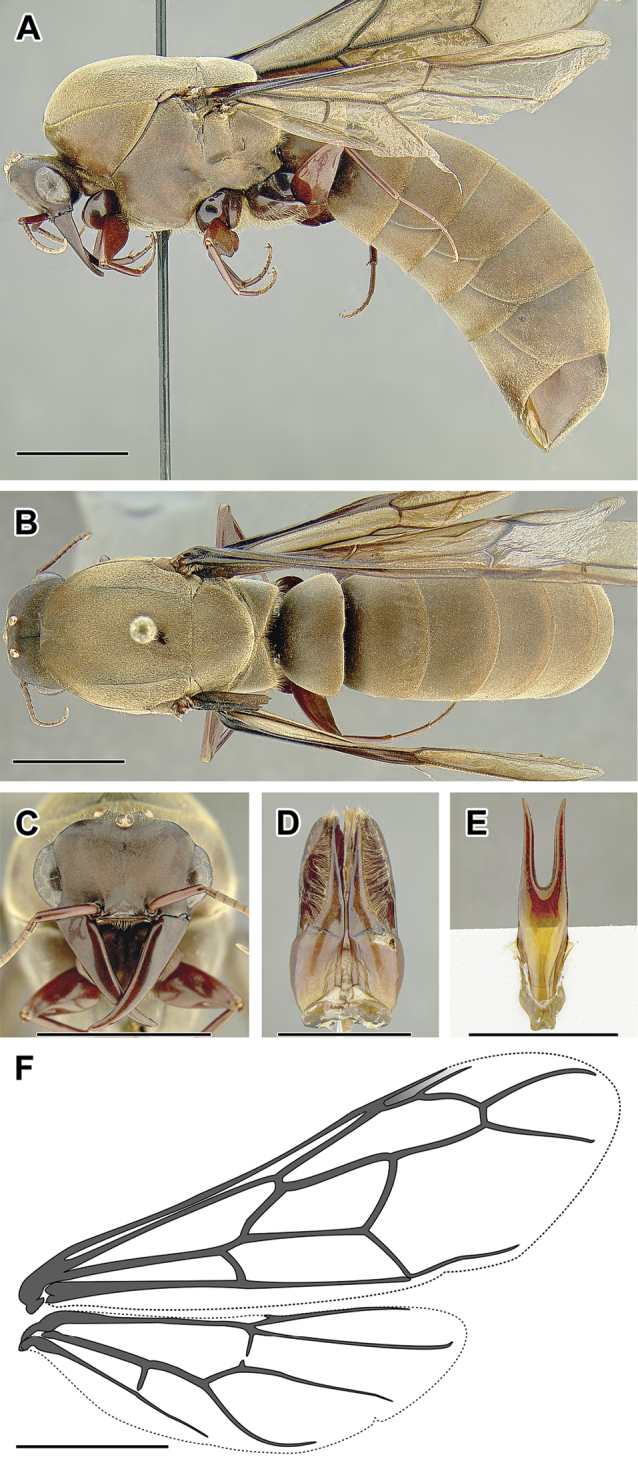
**A–F** Male of *Dorylus nigricans terrificus* (CASENT0731198). **A** Body in lateral view **B** Body in dorsal view **C** Head in full-face view **D** Genital capsule in ventral view **E** Abdominal segment IX (subgenital plate) **F** Wing venation. Scale bar equals 5.0 mm.

**Figure 22.**
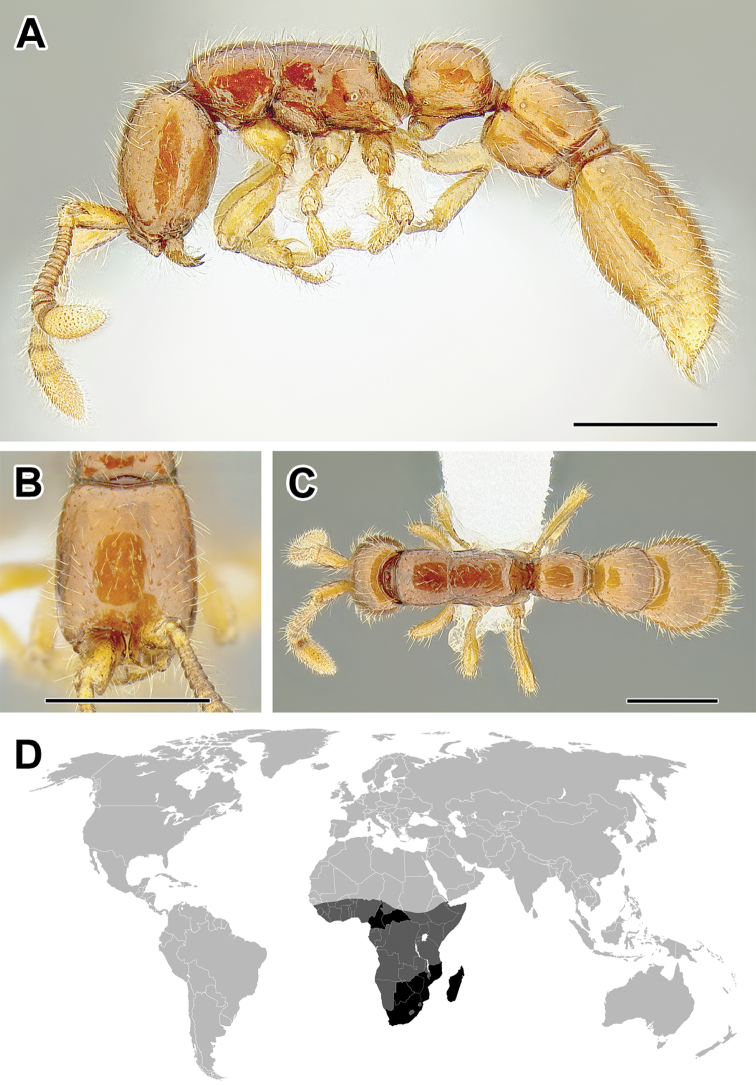
**A–C** Worker of *Eburopone* sp. (CASENT073120) **A** Body in lateral view **B** Head in full-face view **C** Body in dorsal view **D** World distribution of *Eburopone* (black: present, dark grey: likely present). Scale bar equals 0.5 mm.

**Figure 23.**
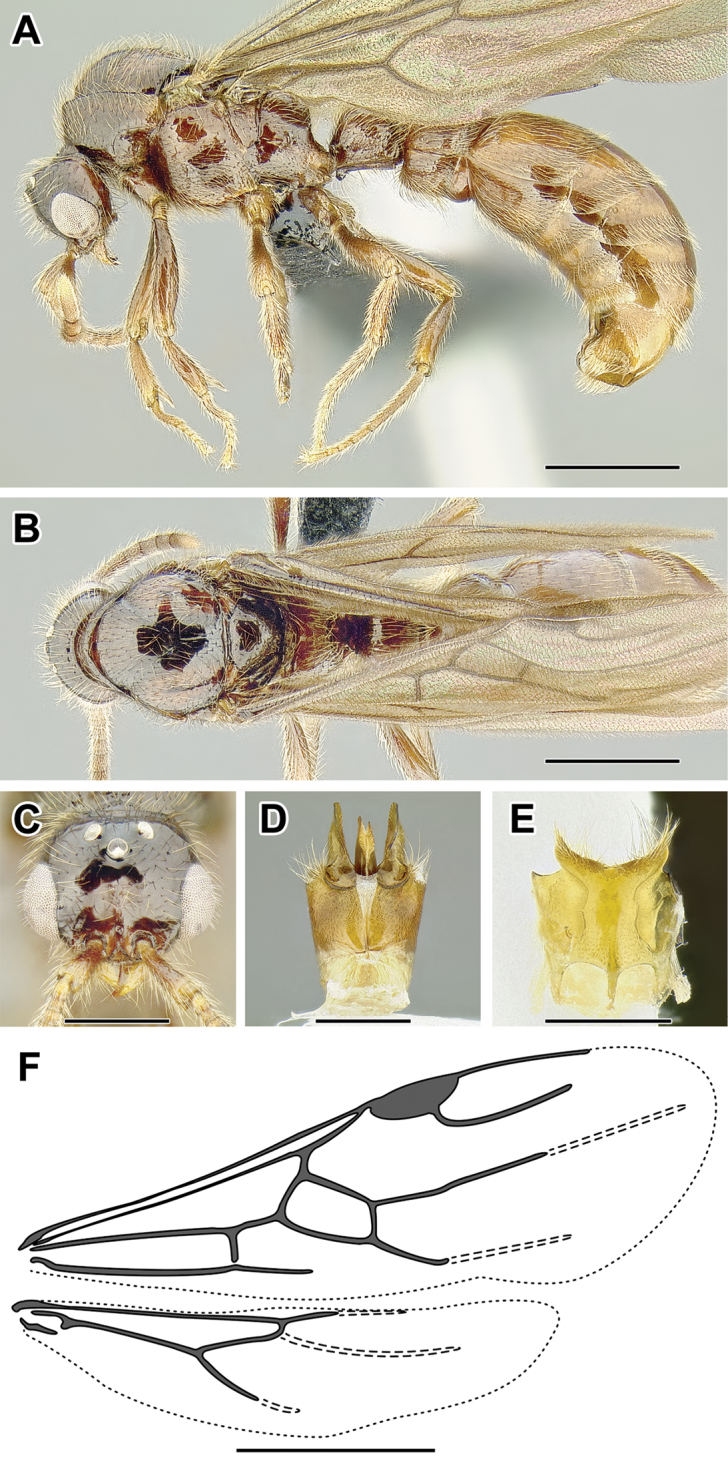
**A–F** Male of *Eburopone* sp. (**A–C** CASENT0731095 **D–F** CASEN0113882) **A** Body in lateral view **B** Body in dorsal view **C** Head in full-face view **D** Genital capsule in ventral view **E** Abdominal segment IX (subgenital plate) **F** Wing venation. Scale bar equals 1.0 mm in **A, B**, and **F**, 0.5 mm in **C–E**.

**Figure 24.**
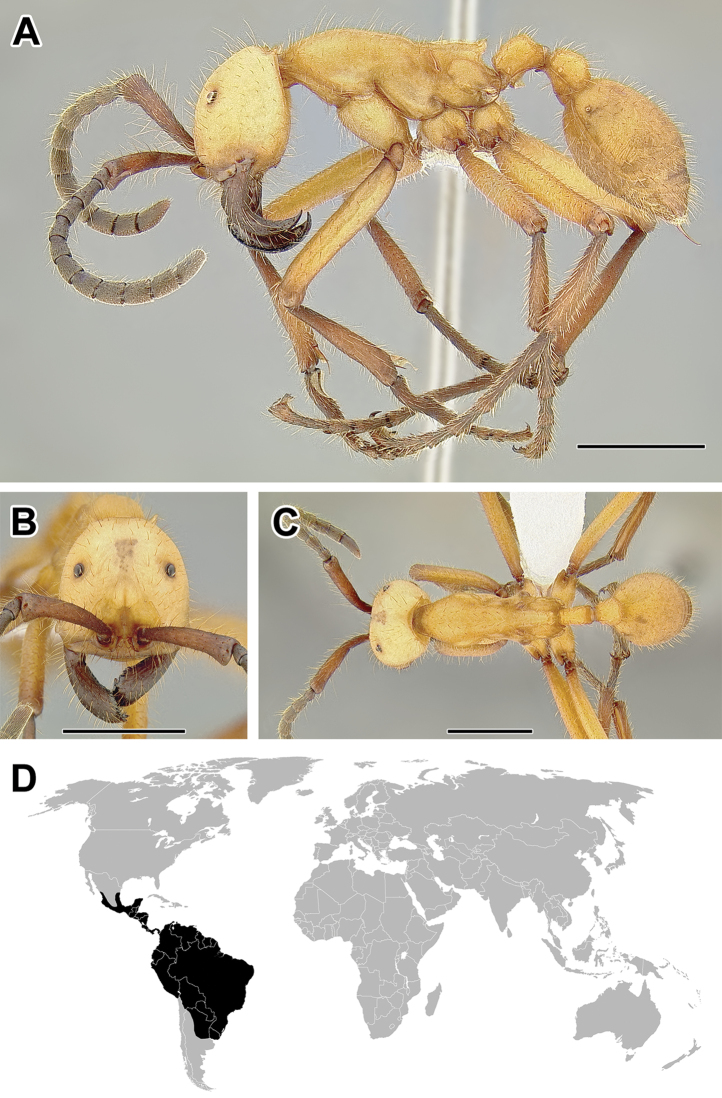
**A–C** Worker of *Eciton hamatum* (CASENT0731194) **A** Body in lateral view **B** Head in full-face view **C** Body in dorsal view **D** World distribution of *Eciton* (black: present, dark grey: likely present). Scale bar equals 2.0 mm.

**Figure 25.**
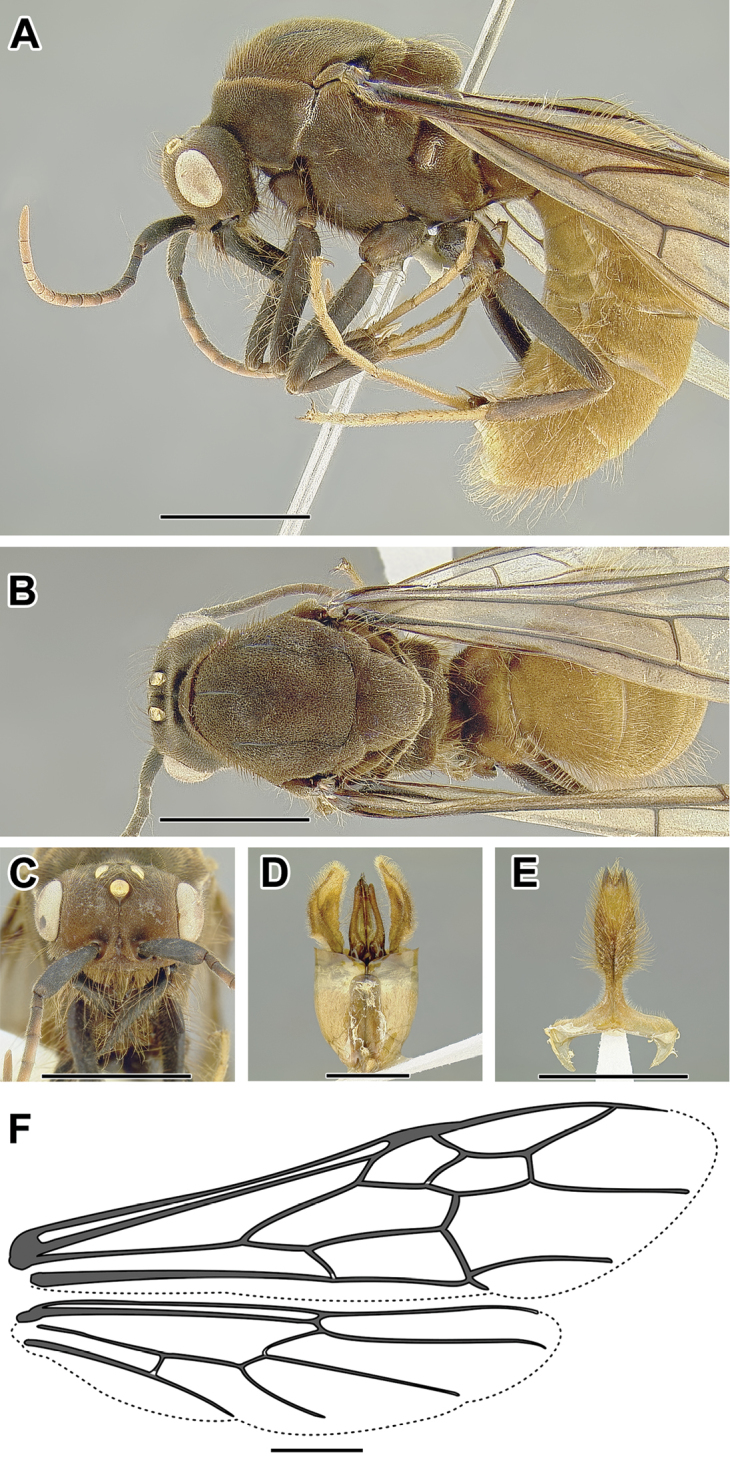
**A–F** Male of *Eciton burchelli* (CASENT0731197) **A** Body in lateral view **B** Body in dorsal view **C** Head in full-face view **D** Genital capsule in ventral view **E** Abdominal segment IX (subgenital plate) **F** Wing venation. Scale bar equals 2.0 mm.

**Figure 26.**
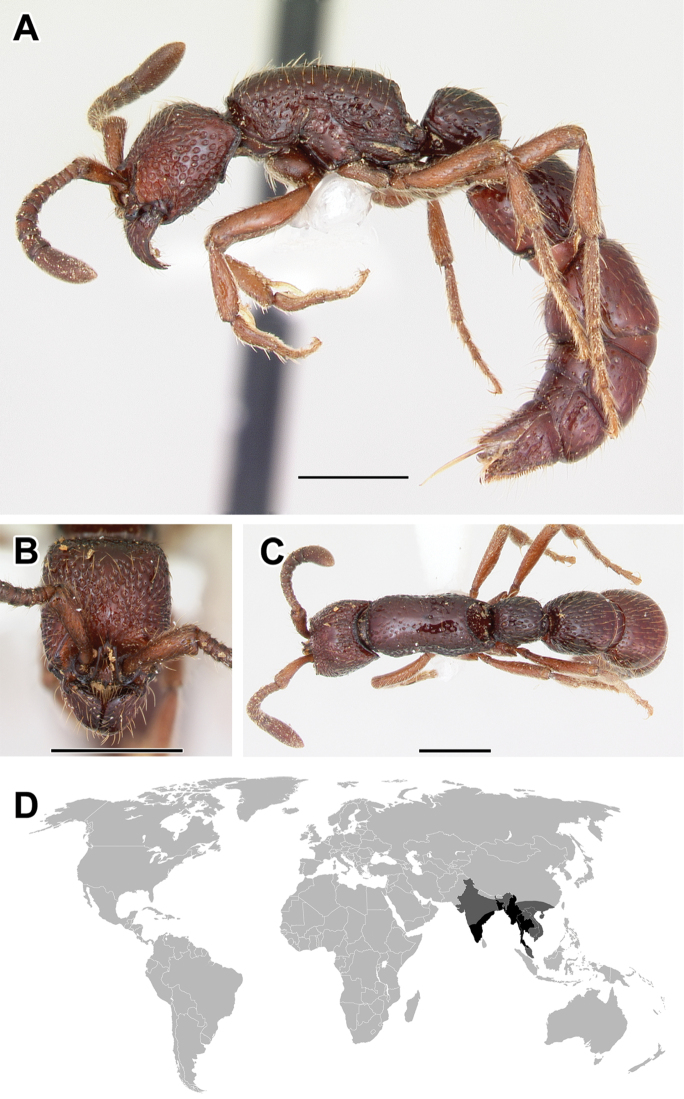
**A–C** Worker of *Eusphinctus furcatus* (CASENT0173056) **A** Body in lateral view **B** Head in full-face view **C** Body in dorsal view **D** World distribution of *Eusphinctus* (black: present, dark grey: likely present). Scale bar equals 1.0 mm. Photographs courtesy of www.antweb.org (April Nobile).

**Figure 27.**
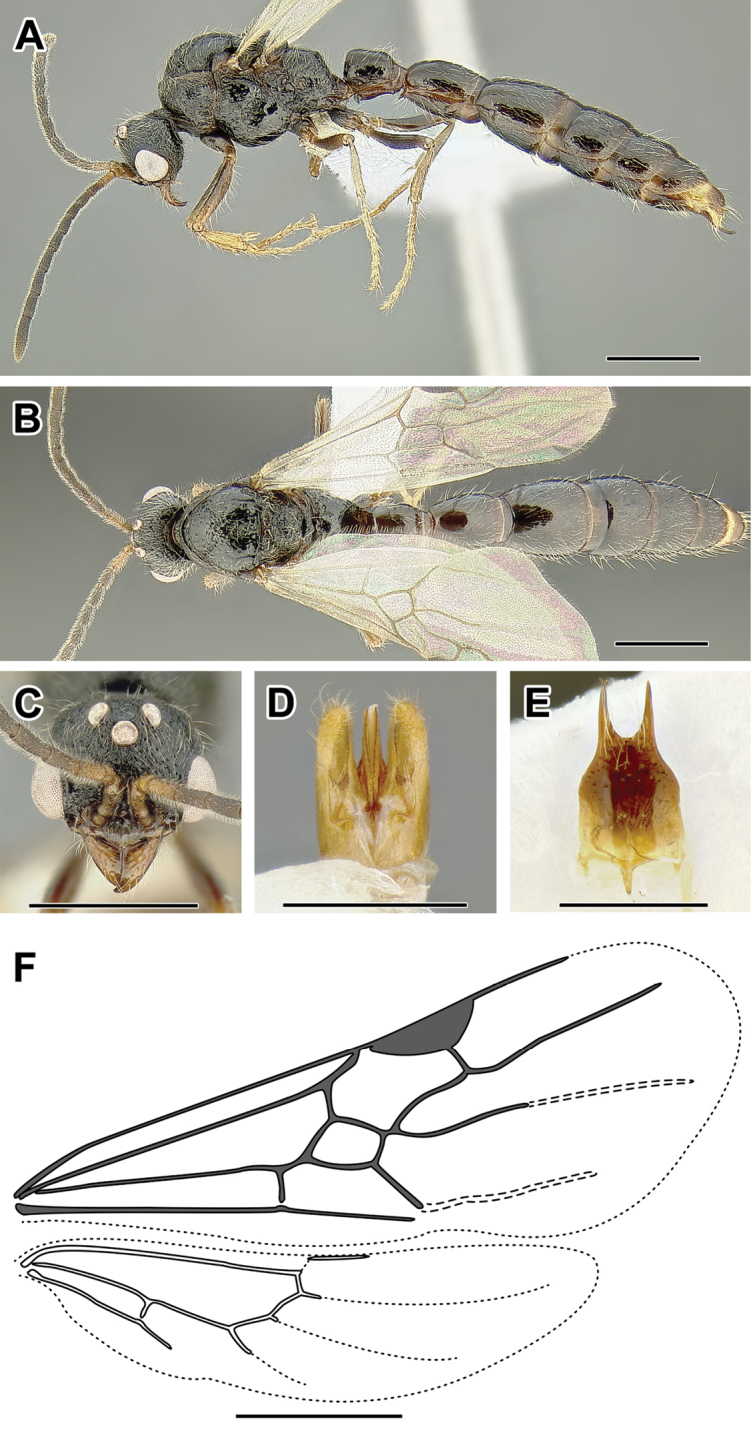
**A–F** Male of *Eusphinctus* sp. (A–C: CASENT0278069, D–F: CASENT0131978) **A** Body in lateral view **B** Body in dorsal view **C** Head in full-face view **D** Genital capsule in ventral view **E** Abdominal segment IX (subgenital plate) **F** Wing venation. Scale bar equals 1.0 mm in **A–C** and **F**, 0.5 mm in **D** and **E**.

**Figure 28.**
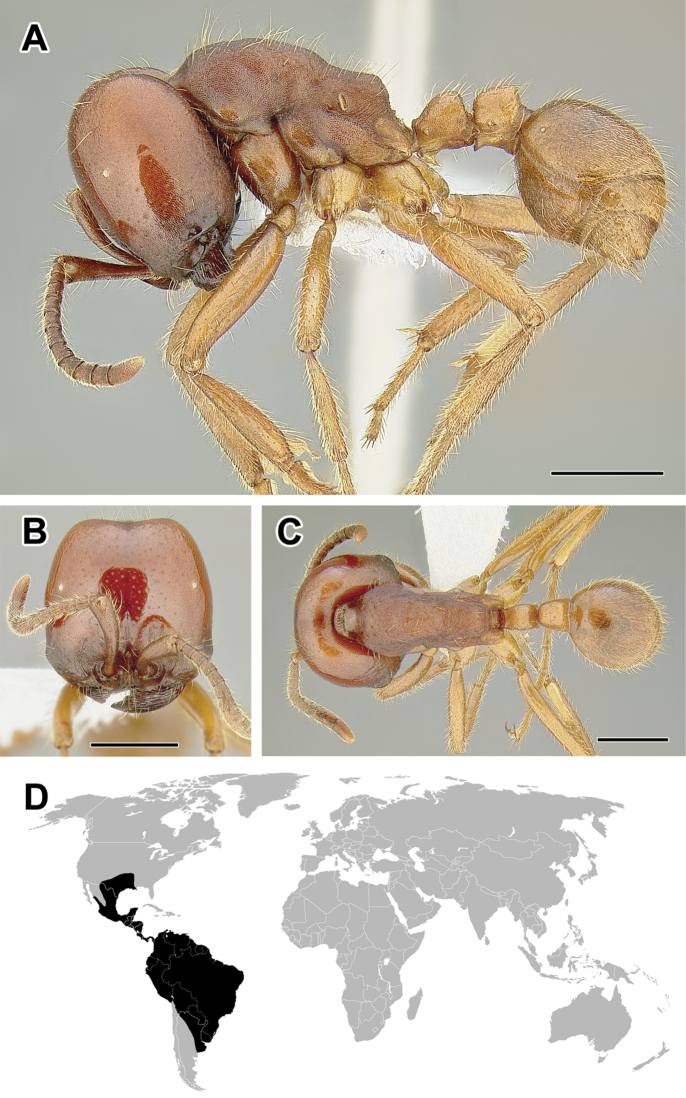
**A–C** Worker of *Labidus coecus* (CASENT0731195) **A** Body in lateral view **B** Head in full-face view **C** Body in dorsal view **D** World distribution of *Labidus* (black: present, dark grey: likely present). Scale bar equals 1.0 mm.

**Figure 29.**
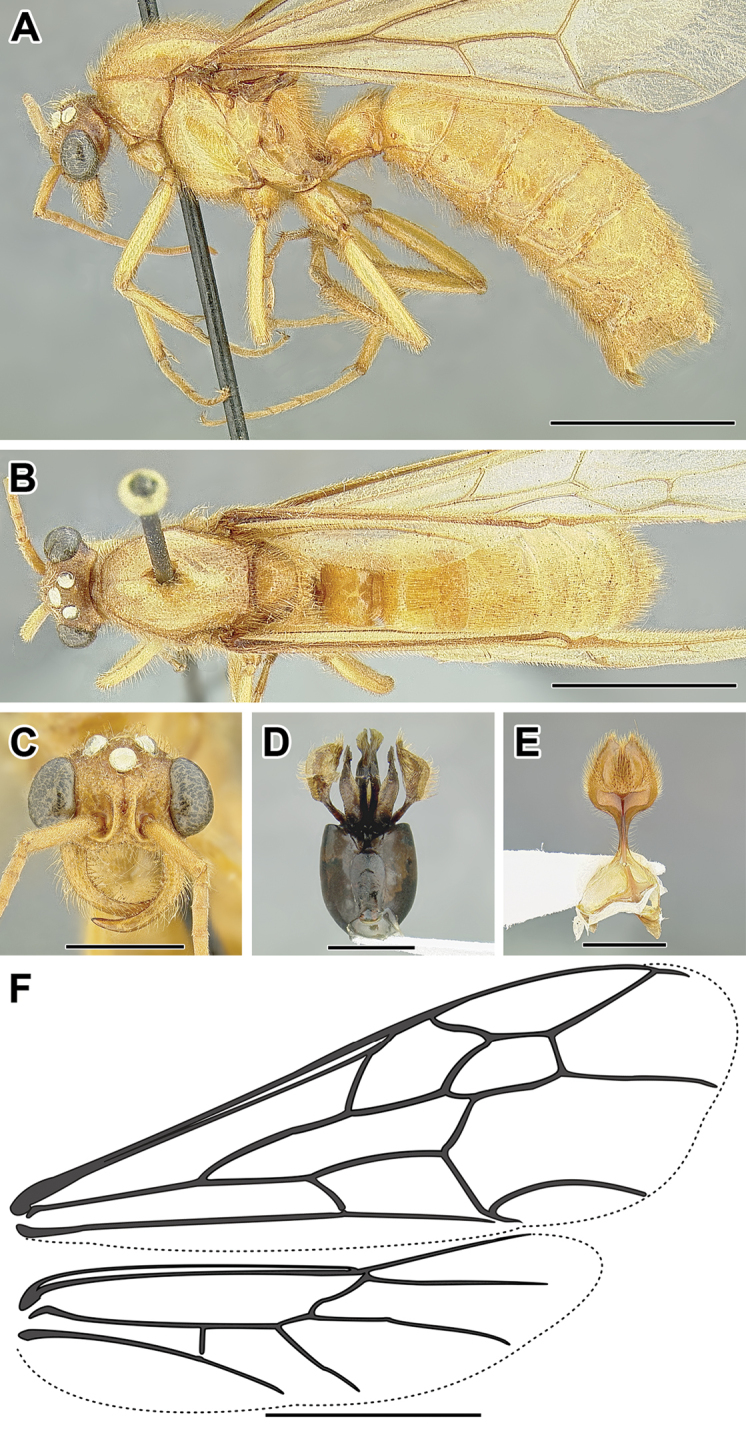
**A–F** Male of *Labidus coecus* (A–C: CASENT0731124, D–F: CASENT0731218) **A** Body in lateral view **B** Body in dorsal view **C** Head in full-face view **D** Genital capsule in ventral view **E** Abdominal segment IX (subgenital plate) **F** Wing venation. Scale bar equals 5.0 mm in **A, B**, and **F**, 2.0 mm in **C–E**.

**Figure 30.**
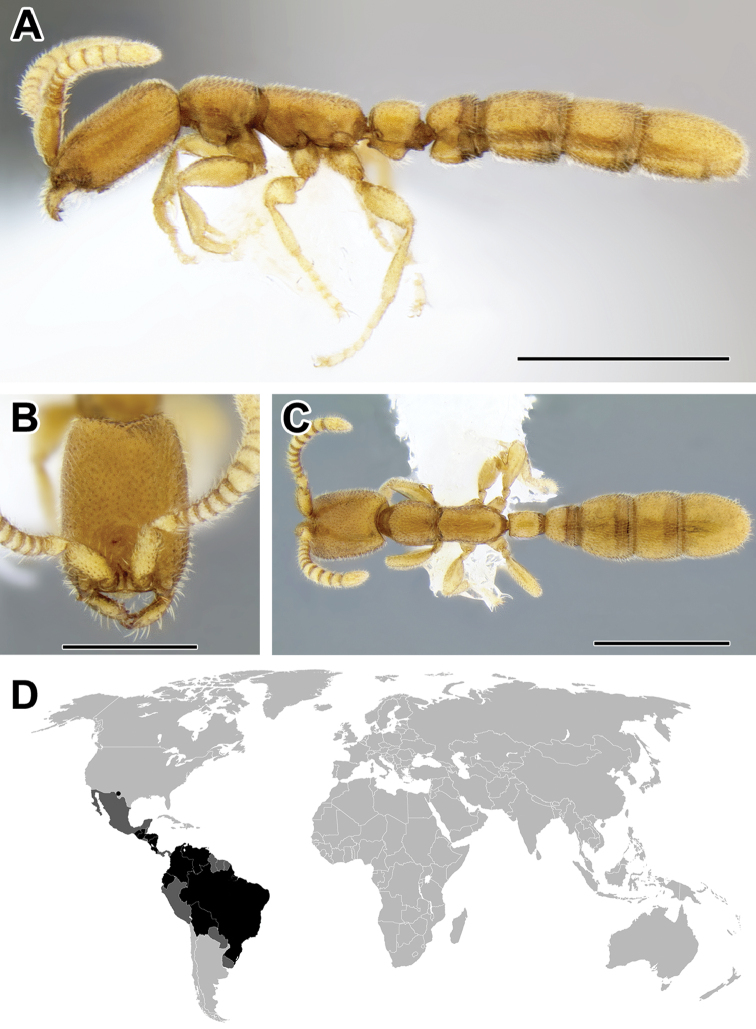
**A–C** Worker of *Leptanilloides gracilis* (CASENT0234574) **A** Body in lateral view **B** Head in full-face view **C** Body in dorsal view **D** World distribution of *Leptanilloides* (black: present, dark grey: likely present). Scale bar equals 0.5 mm in **A** and **C**, 0.25 mm in **B**.

**Figure 31.**
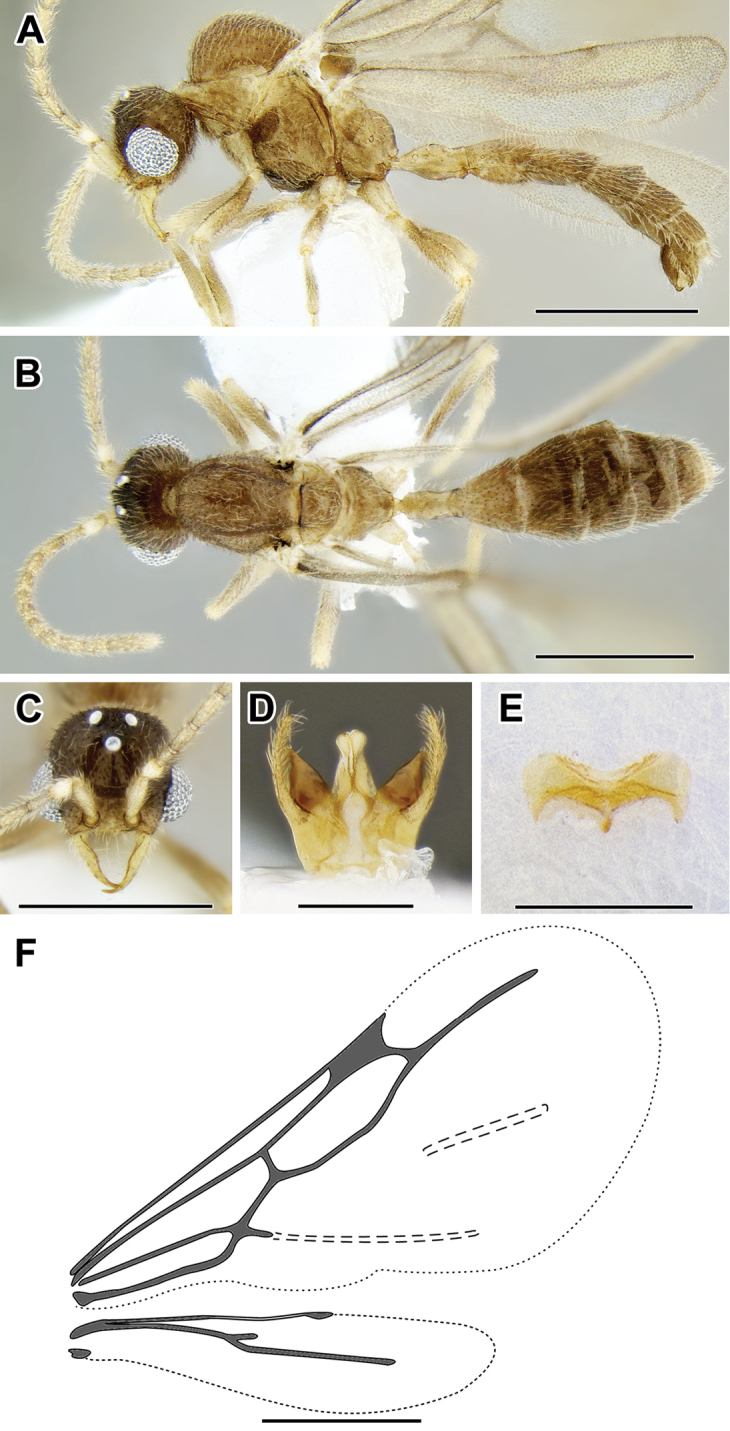
**A–F** Male of *Leptanilloides* sp. (A–C, F: CASENT0234556, D and E: CASENT0731110) **A** Body in lateral view **B** Body in dorsal view **C** Head in full-face view **D** Genital capsule in ventral view **E** Abdominal segment IX (subgenital plate) **F** Wing venation. Scale bar equals 0.5 mm in **A–C** and **F**, 0.25 mm in **D** and **E**.

**Figure 32.**
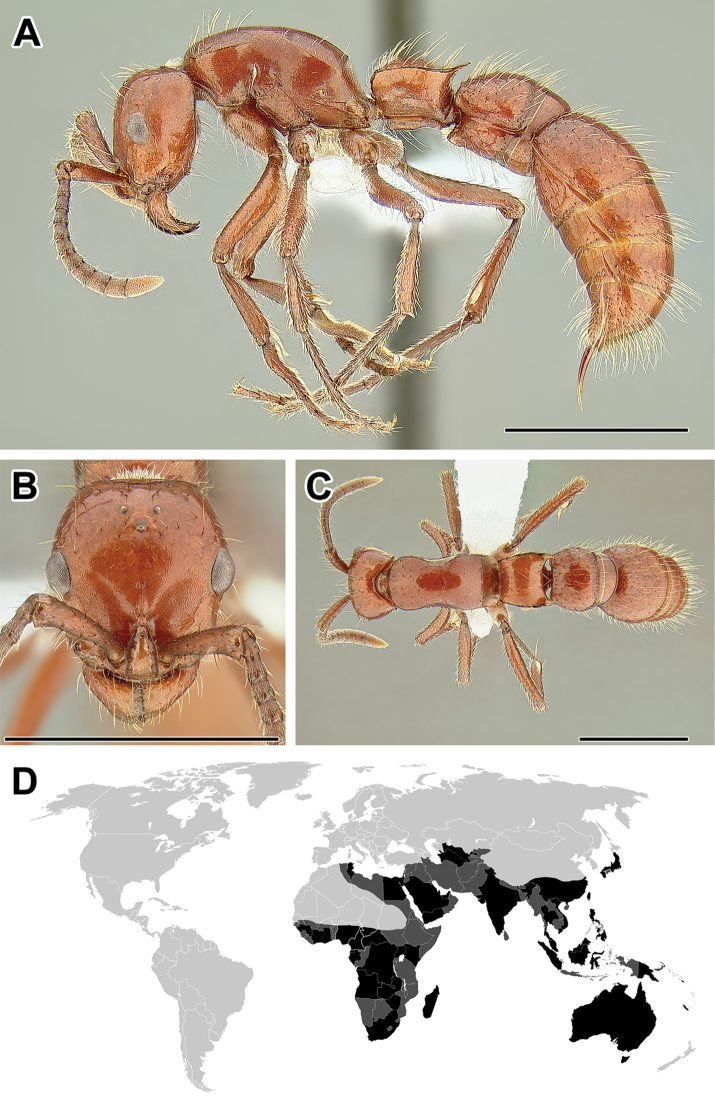
**A–C** Worker of *Lioponera clarus* (CASENT0731128) **A** Body in lateral view **B** Head in full-face view **C** Body in dorsal view **D** World distribution of *Lioponera* (black: present, dark grey: likely present). Scale bar equals 2.0 mm.

**Figure 33.**
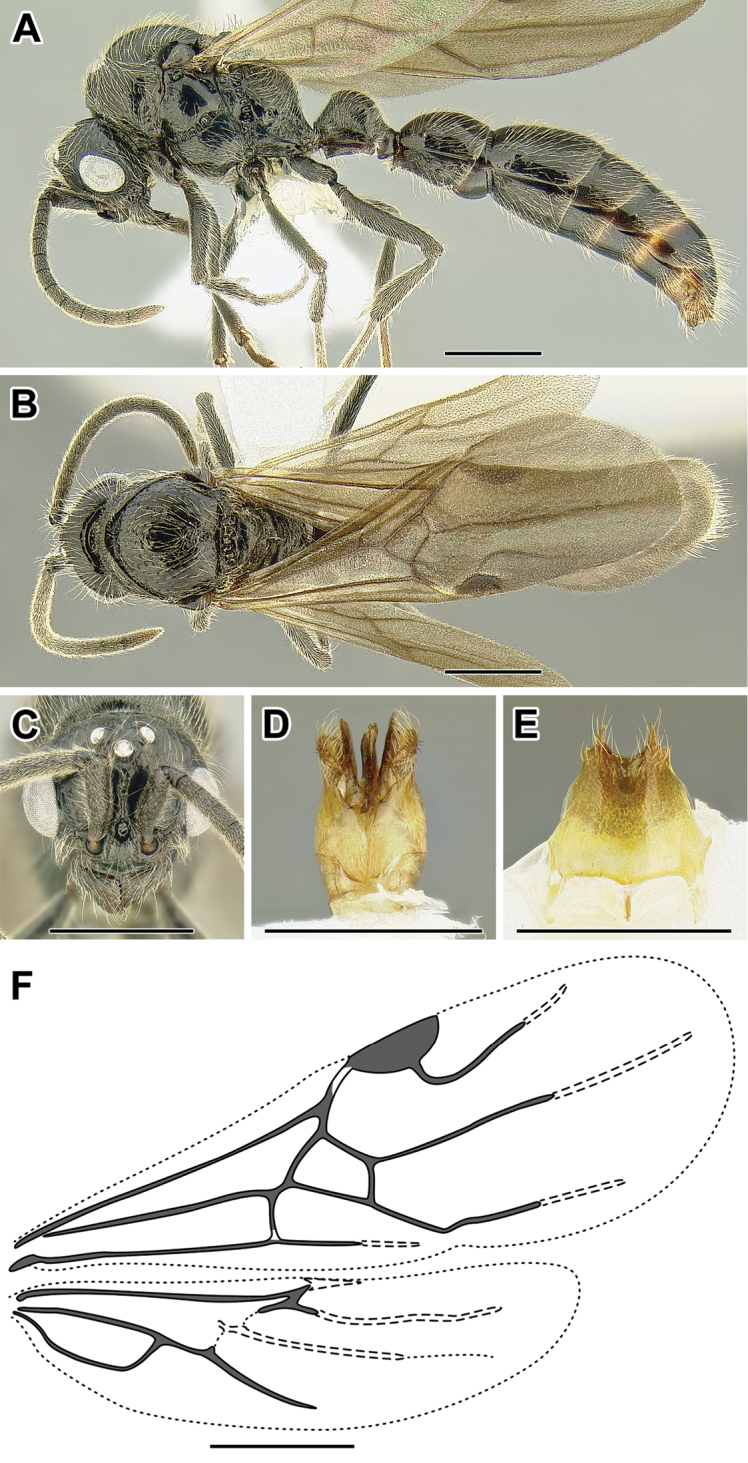
**A–F** Male of Lioponera cf. mayri (**A–C** CASENT0731200 **D–F** CASENT0234856) **A** Body in lateral view **B** Body in dorsal view **C** Head in full-face view **D** Genital capsule in ventral view **E** Abdominal segment IX (subgenital plate) **F** Wing venation. Scale bar equals 1.0 mm.

**Figure 34.**
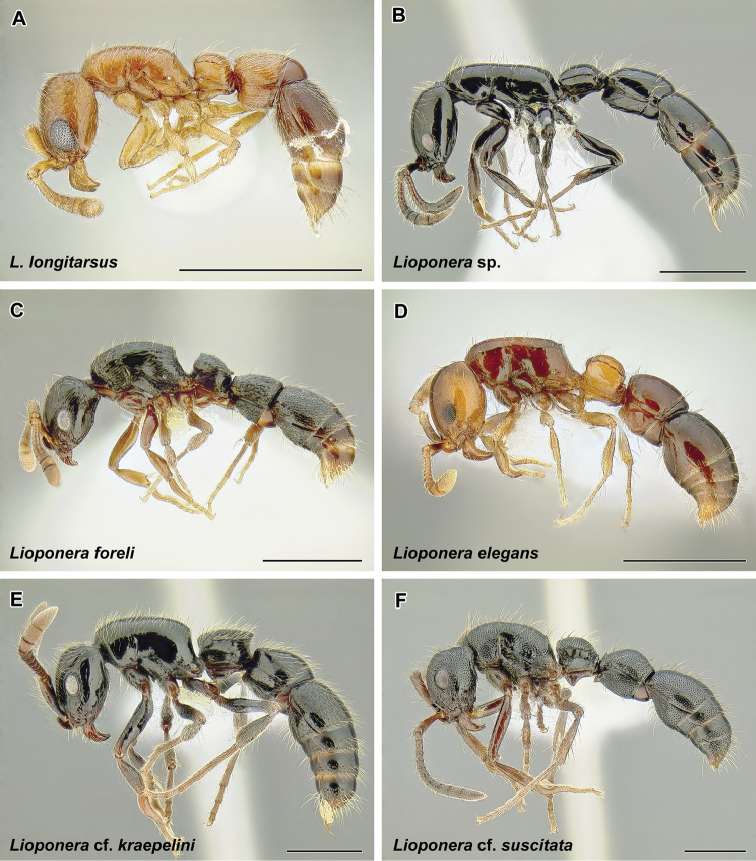
**A–F** Morphological diversity of *Lioponera*. **A** *Lioponera longitarsus* (CASENT0731207) **B** *Lioponera* sp. (CASENT0215877) **C** *Lioponera foreli* (CASENT0731206) **D** *Lioponera elegans* (CASENT0249293) **E** Lioponera cf. kraepelini (CASENT0731204) **F** Lioponera cf. suscitata (CASENT0731205). Scale bar equals 1.0 mm.

**Figure 35.**
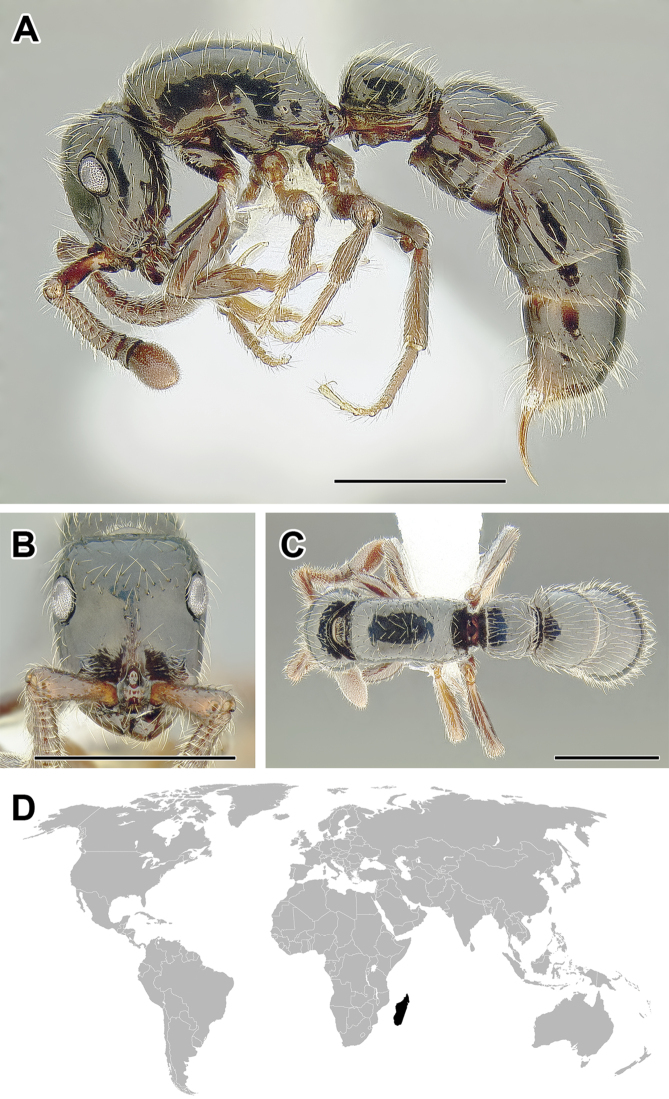
**A–C** Worker of *Lividopone livida* (CASENT0731209) **A** Body in lateral view **B** Head in full-face view **C** Body in dorsal view **D** World distribution of *Lividopone* (black: present, dark grey: likely present). Scale bar equals 1.0 mm.

**Figure 36.**
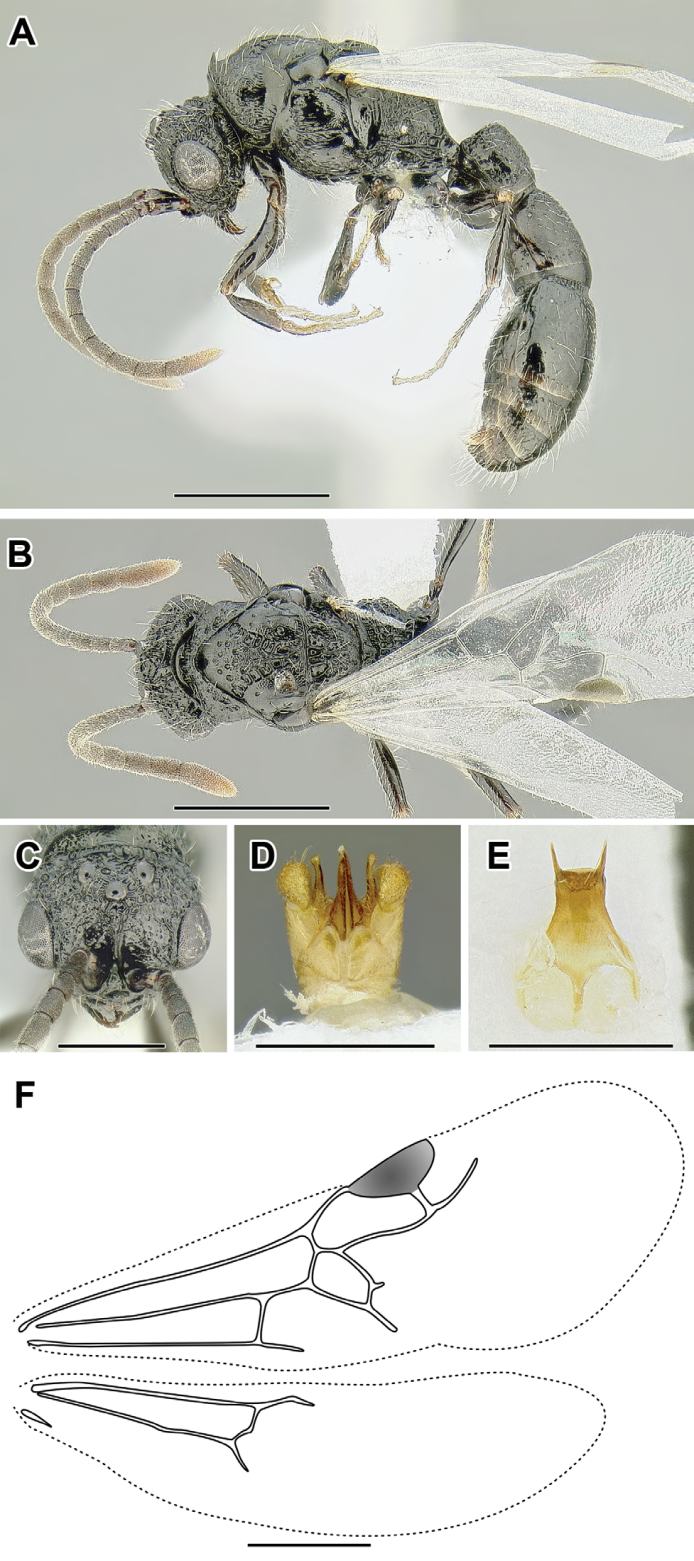
**A–F** Male of *Lividopone* sp. (CASENT0234857) **A** Body in lateral view **B** Body in dorsal view **C** Head in full-face view **D** Genital capsule in ventral view **E** Abdominal segment IX (subgenital plate) **F** Wing venation. Scale bar equals 1.0 mm in **A** and **B**, 0.5 mm in **C–F**.

**Figure 37.**
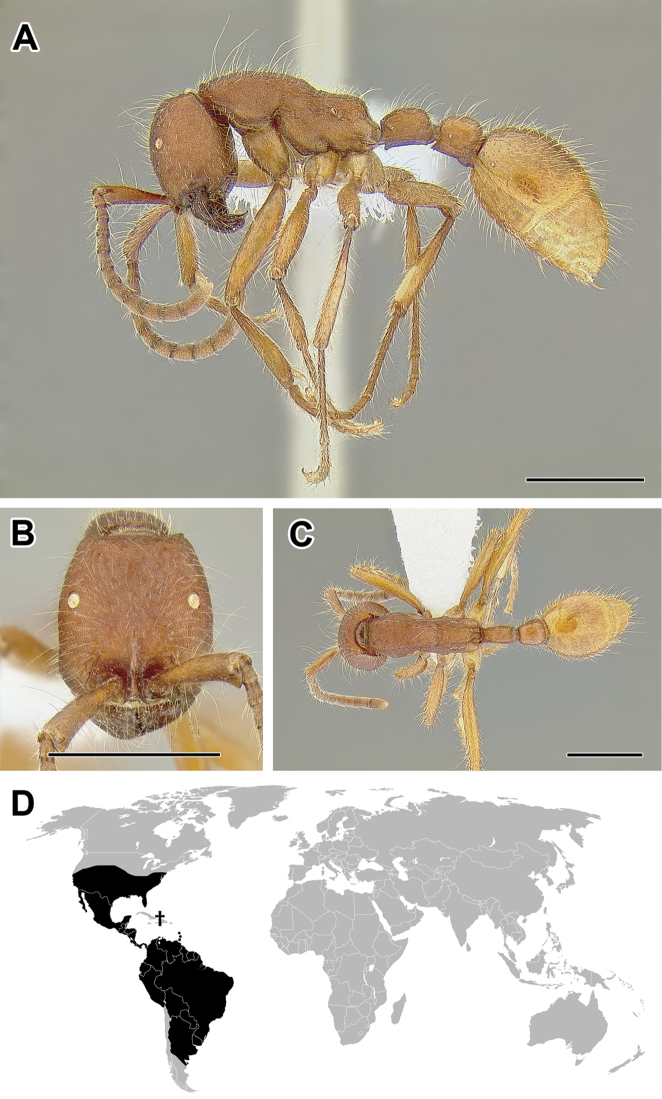
**A–C** Worker of *Neivamyrmex nigrescens* (CASENT0249493) **A** Body in lateral view **B** Head in full-face view **C** Body in dorsal view **D** World distribution of *Neivamyrmex* (black: present, dark grey: likely present). Scale bar equals 1.0 mm.

**Figure 38.**
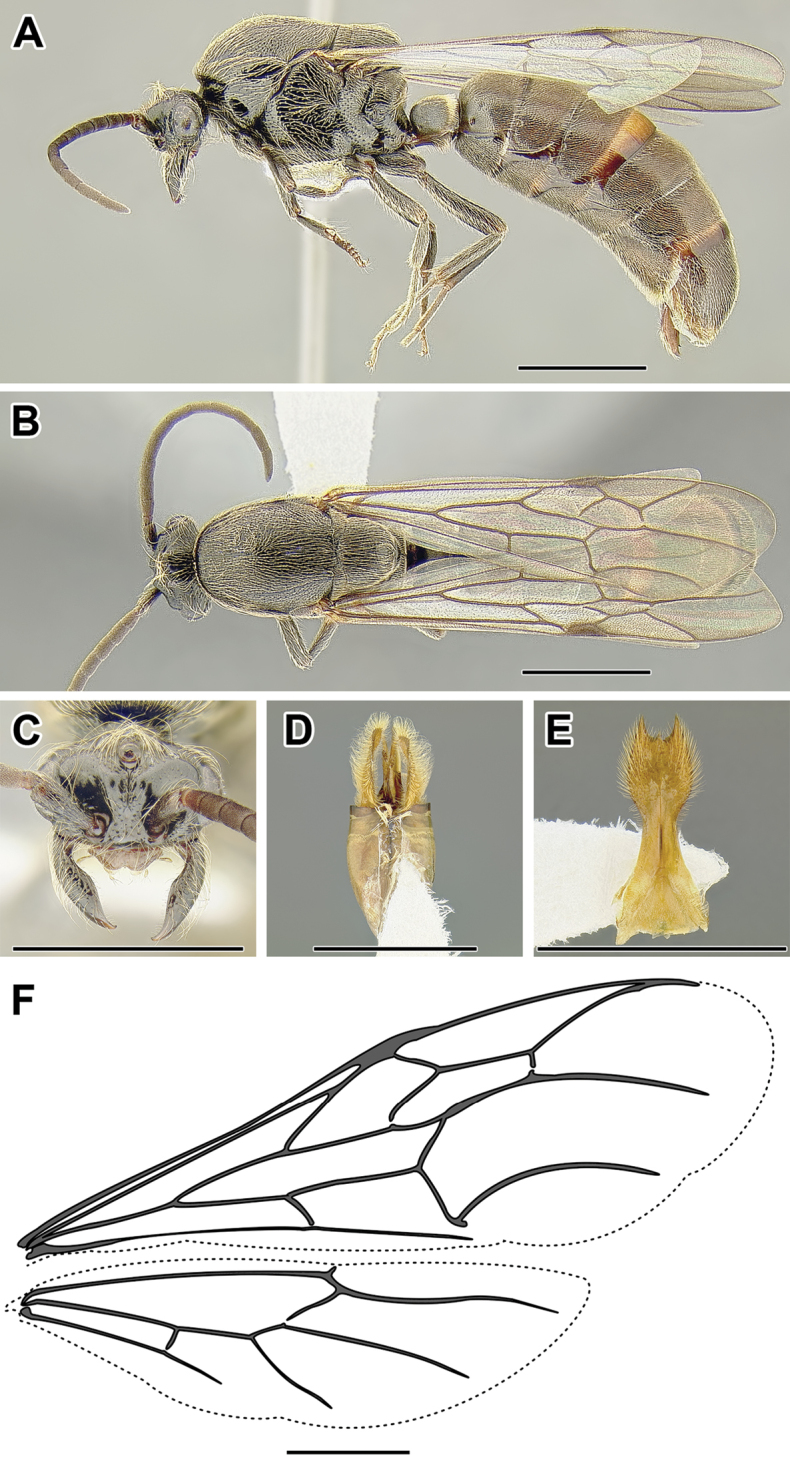
**A–F** Male of *Neivamyrmex nigrescens* (CASENT0732110) **A** Body in lateral view **B** Body in dorsal view **C** Head in full-face view **D** Genital capsule in ventral view **E** Abdominal segment IX (subgenital plate) **F** Wing venation. Scale bar equals 2.0 mm.

**Figure 39.**
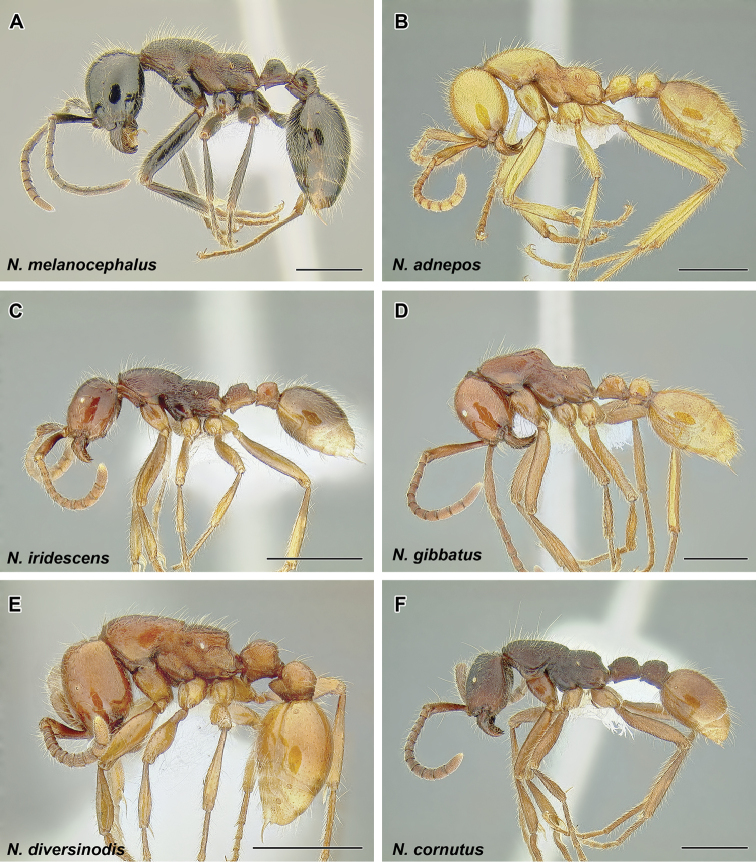
**A–F** Morphological diversity of *Neivamyrmex*. **A** *Neivamyrmex melanocephalus* (CASENT0731183) **B** *Neivamyrmex adnepos* (CASENT0249470) **C** *Neivamyrmex iridescens* (CASENT0249488) **D** *Neivamyrmex gibbatus* (CASENT0731189) **E** *Neivamyrmex diversinodis* (CASENT0249480) **F** *Neivamyrmex cornutus* (CASENT0249478). Scale bar equals 1.0 mm.

**Figure 40.**
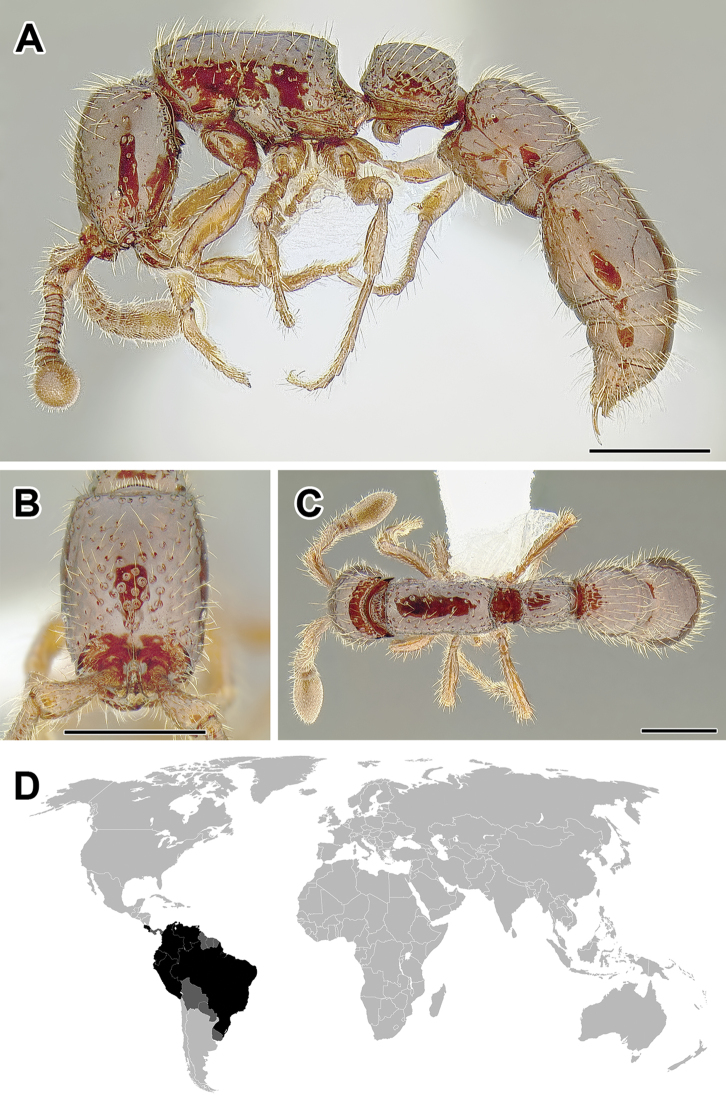
**A–C** Worker of *Neocerapachys neotropicus*. **A** Body in lateral view **B** Head in full-face view **C** Body in dorsal view **D** World distribution of *Neocerapachys* (black: present, dark grey: likely present). Scale bar equals 0.5 mm.

**Figure 41.**
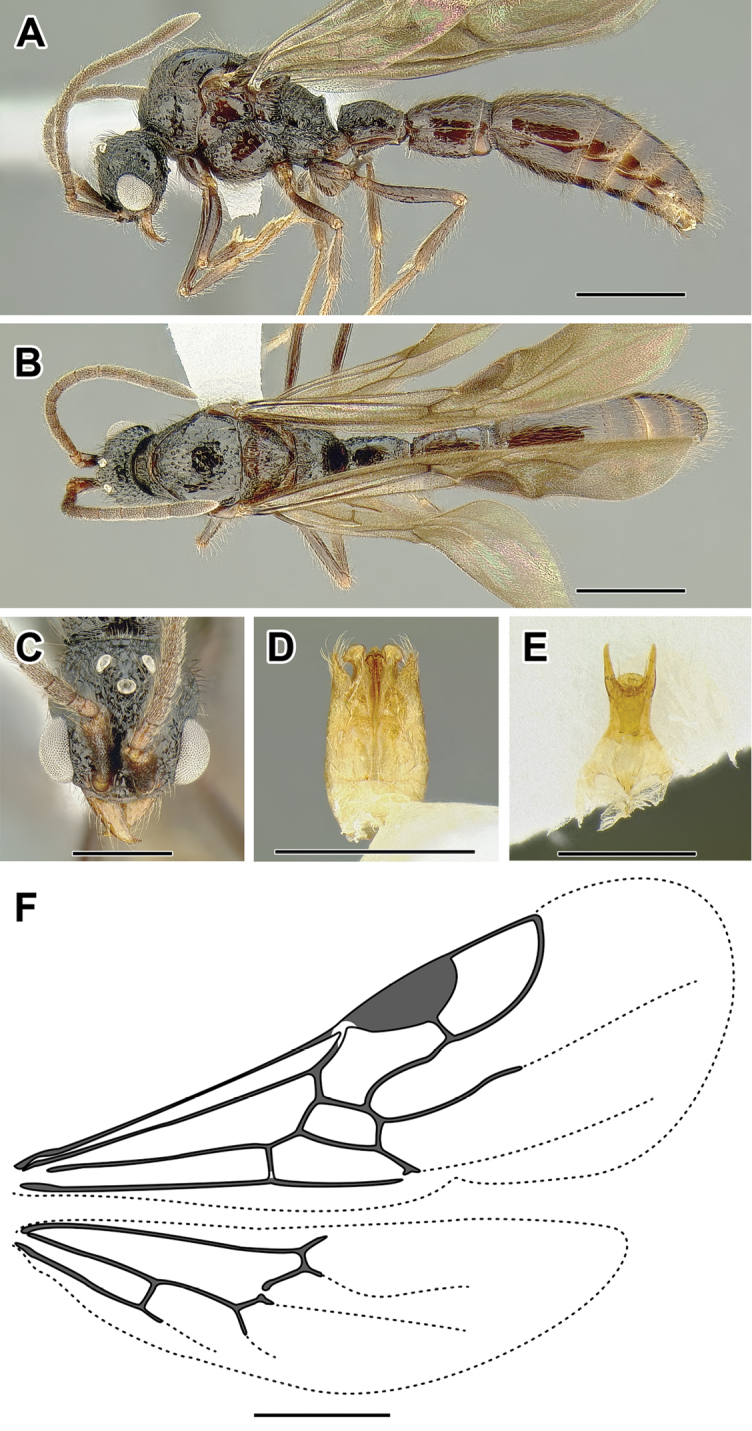
**A–F** Male of *Neocerapachys* sp. (A–C: CASENT0731109, D–F: CASENT0731210) **A** Body in lateral view **B** Body in dorsal view **C** Head in full-face view **D** Genital capsule in ventral view **E** Abdominal segment IX (subgenital plate) **F** Wing venation. Scale bar equals 1.0 mm in **A, B**, and **F**, 0.5 mm in **C–E**.

**Figure 42.**
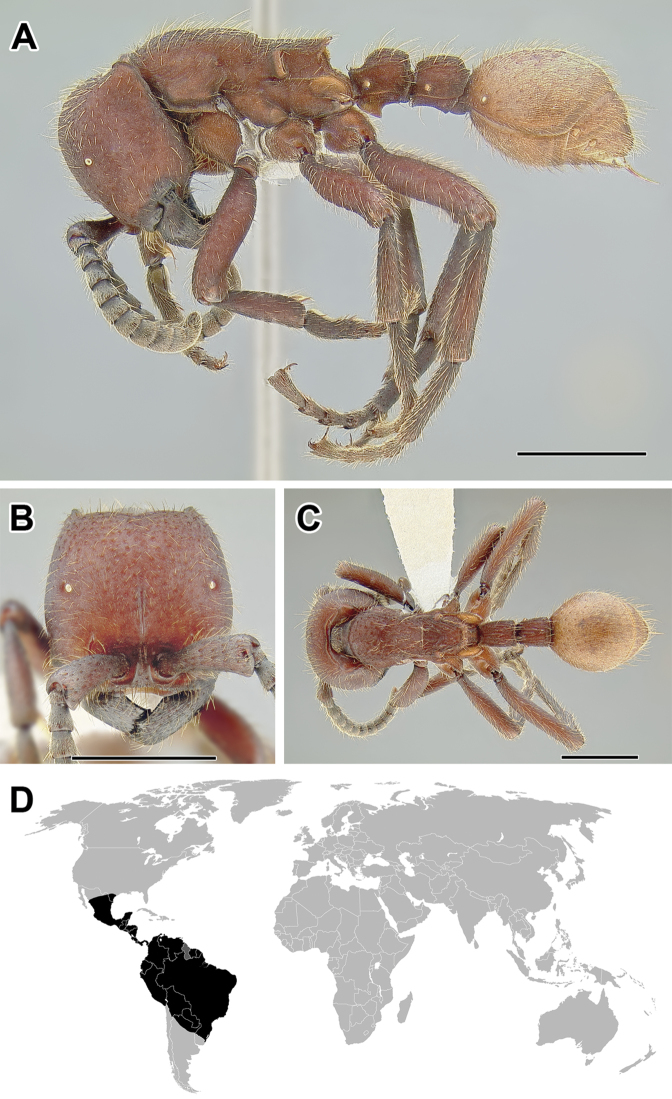
**A–C** Worker of *Nomamyrmex esenbeckii* (CASENT0731191) **A** Body in lateral view **B** Head in full-face view **C** Body in dorsal view **D** World distribution of *Nomamyrmex* (black: present, dark grey: likely present). Scale bar equals 2.0 mm.

**Figure 43.**
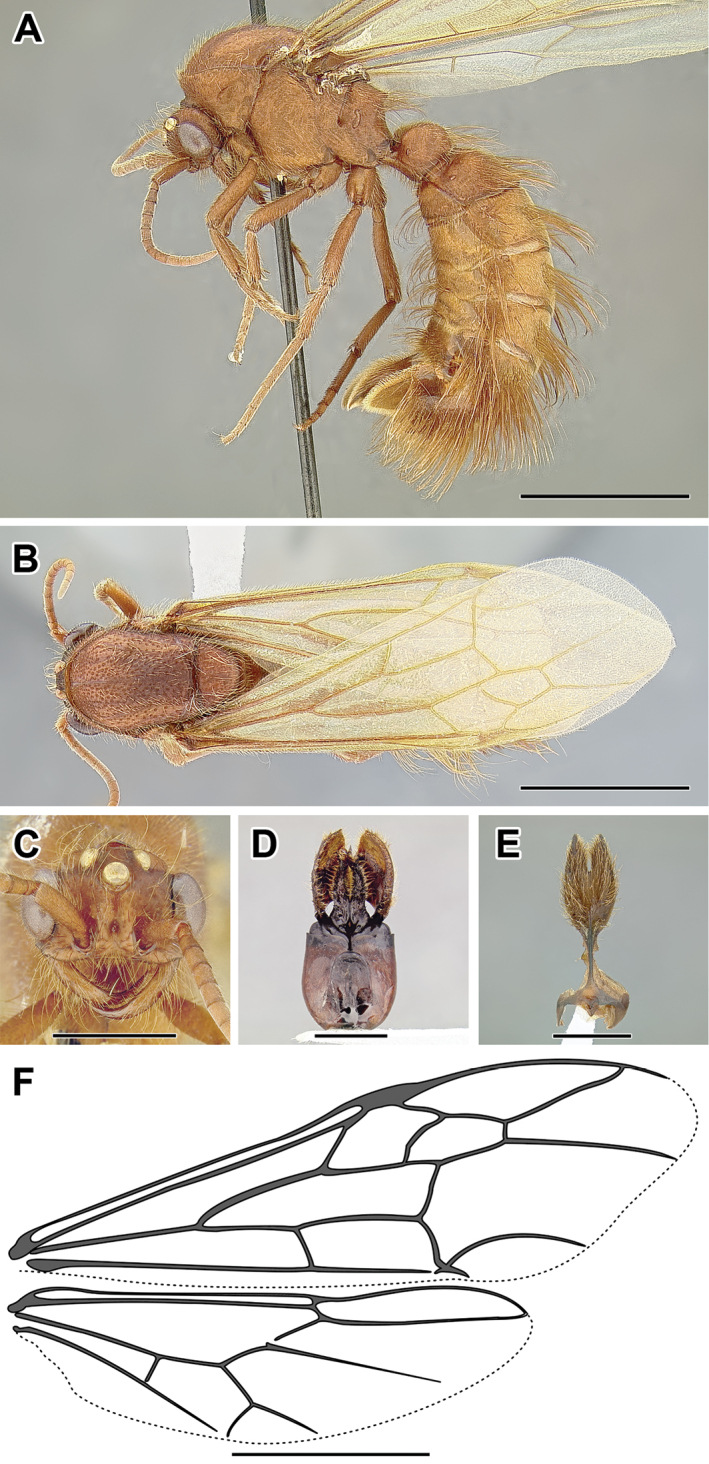
**A–F** Male of *Nomamyrmex esenbeckii* (CASENT0731217) **A** Body in lateral view **B** Body in dorsal view **C** Head in full-face view **D** Genital capsule in ventral view **E** Abdominal segment IX (subgenital plate) **F** Wing venation. Scale bar equals 5.0 mm in **A, B**, and **F**, 2.0 mm in **C–E**.

**Figure 44.**
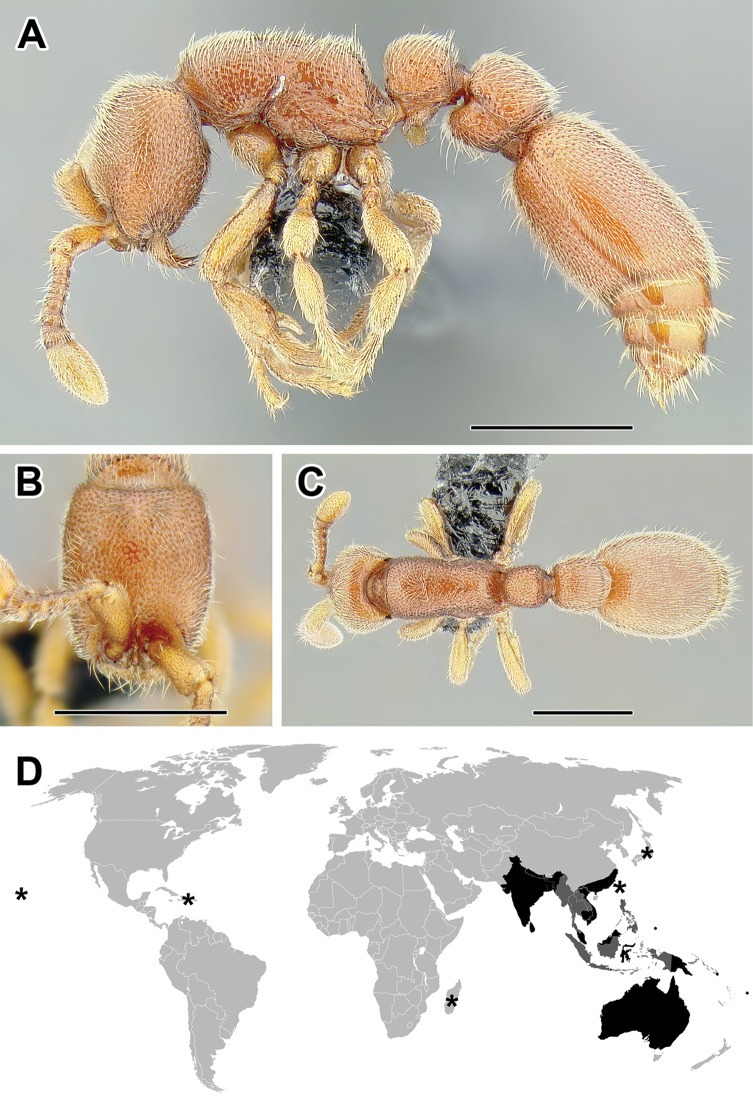
**A–C** Worker of *Ooceraea biroi* (CASENT0731215) **A** Body in lateral view **B** Head in full-face view **C** Body in dorsal view **D** World distribution of *Ooceraea* (black: present, dark grey: likely present, asterisk: introduced). Scale bar equals 0.5 mm.

**Figure 45.**
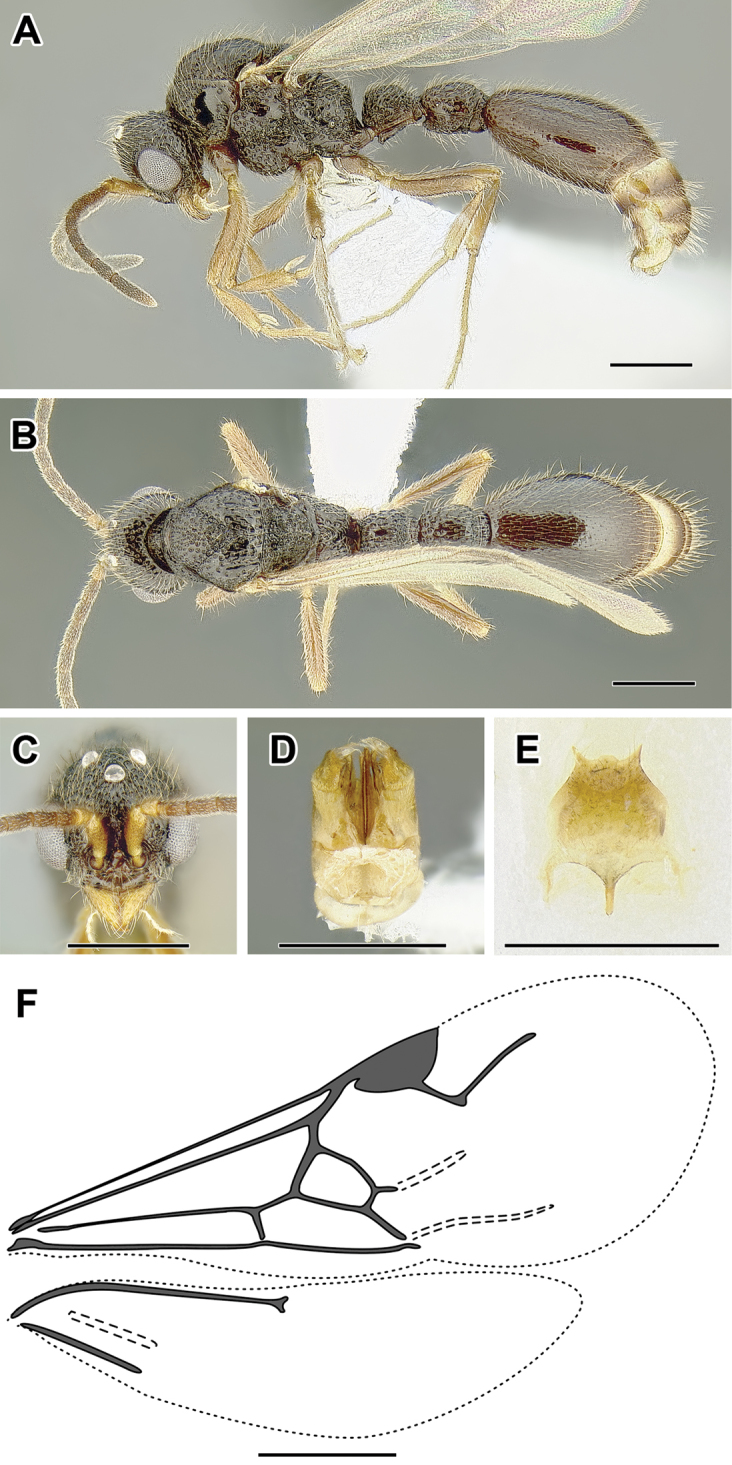
**A–F** Male of *Ooceraea* sp. (**A–C, F** CASENT0731100 **D** and **E** CASENT0731098) **A** Body in lateral view **B** Body in dorsal view **C** Head in full-face view **D** Genital capsule in ventral view **E** Abdominal segment IX (subgenital plate) **F** Wing venation. Scale bar equals 0.5 mm.

**Figure 46.**
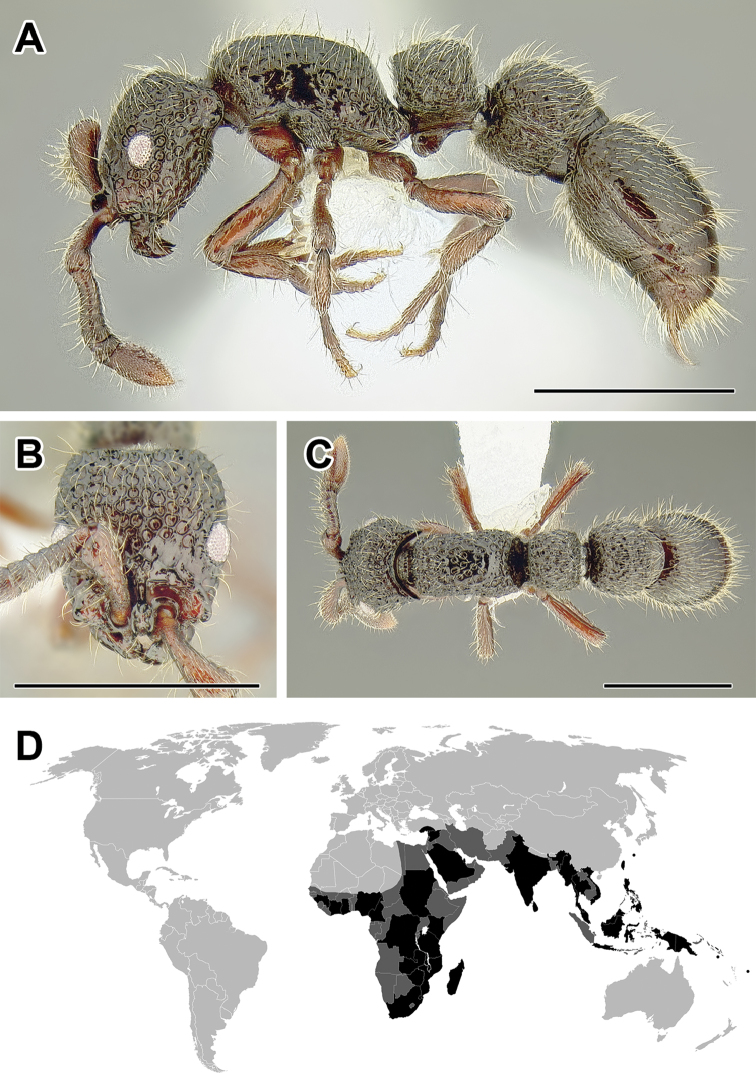
**A–C** Worker of *Parasyscia kodecorum* (CASENT0731152) **A** Body in lateral view **B** Head in full-face view **C** Body in dorsal view **D** World distribution of *Parasyscia* (black: present, dark grey: likely present). Scale bar equals 1.0 mm.

**Figure 47.**
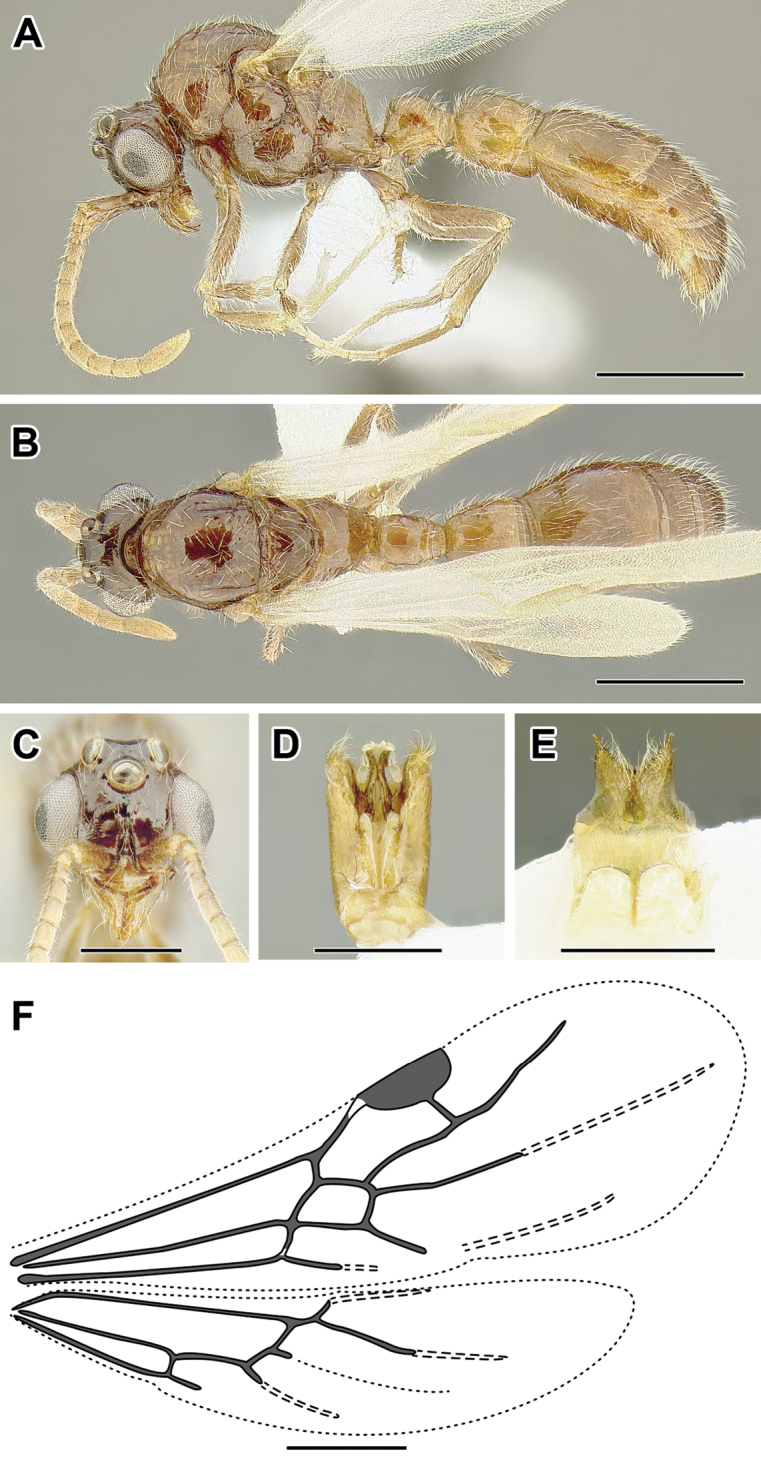
**A–F** Male of *Parasyscia* sp. (**A–C** CASENT0731116 **D–F** CASENT0731101) **A** Body in lateral view **B** Body in dorsal view **C** Head in full-face view **D** Genital capsule in ventral view **E** Abdominal segment IX (subgenital plate) **F** Wing venation. Scale bar equals 1.0 in **A** and **B**, 0.5 mm in **C–F**.

**Figure 48.**
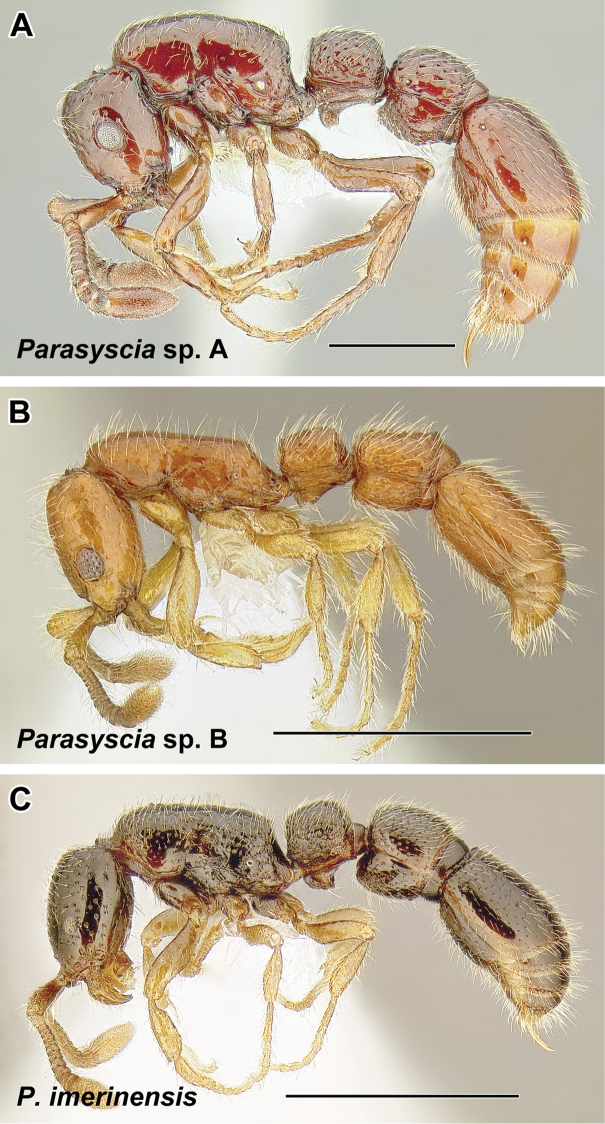
**A–C** Morphological diversity of *Parasyscia*. **A** *Parasyscia* sp. A (CASENT0731212) **B** *Parasyscia* sp. B (CASENT0216859) **C** *Parasyscia imerinensis* (CASENT0731170). Scale bar equals 1.0 mm.

**Figure 49.**
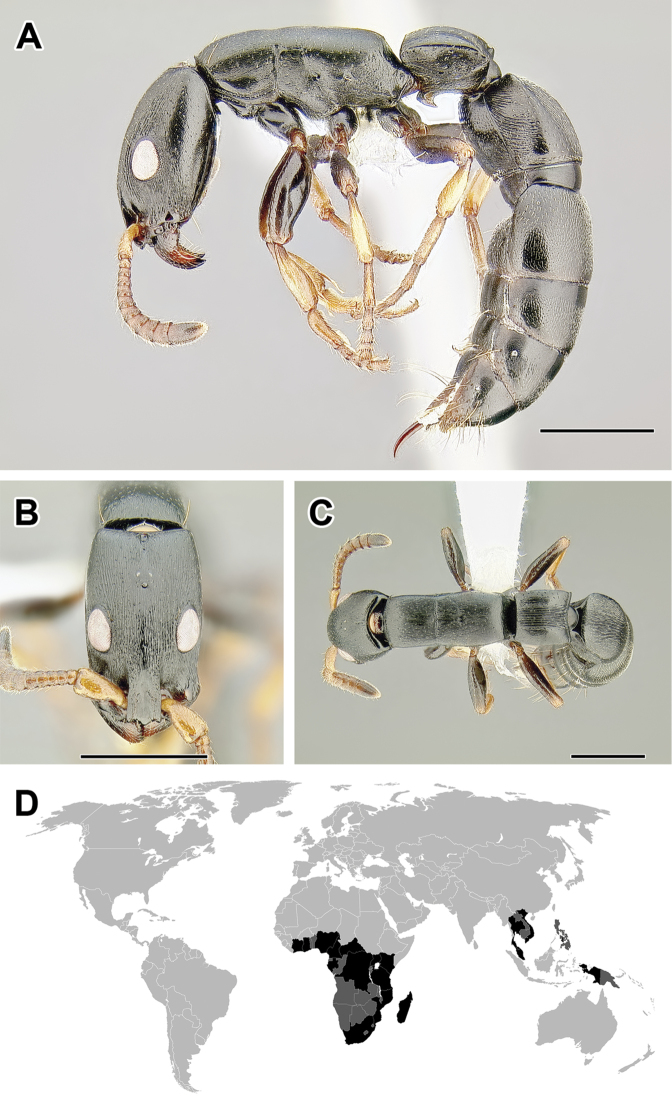
**A–C** Worker of *Simopone conradti* (CASENT0731157) **A** Body in lateral view **B** Head in full-face view **C** Body in dorsal view **D** World distribution of *Simopone* (black: present, dark grey: likely present). Scale bar equals 1.0 mm.

**Figure 50.**
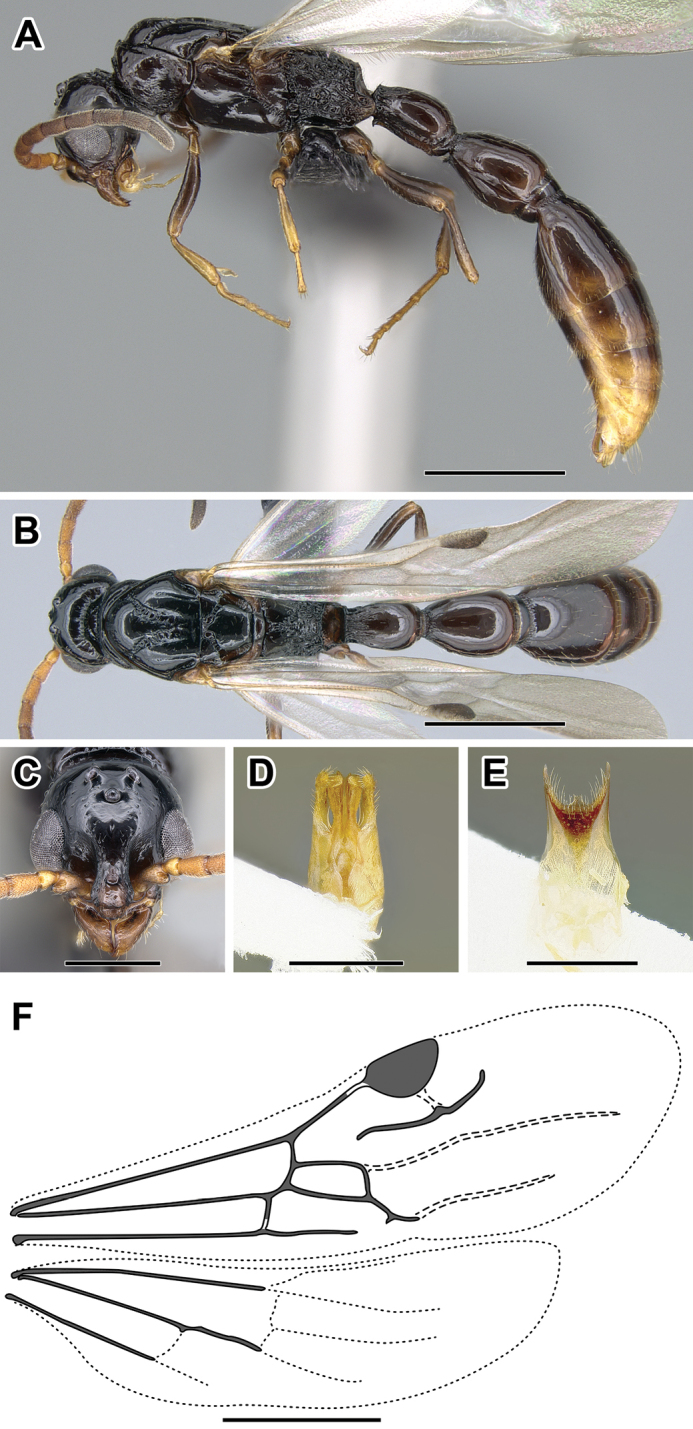
**A–F** Male of *Simopone grandidieri* (**A–C** CASENT0148973), *Simopone marleyi* (**D–F** CASENT0731102). **A** Body in lateral view **B** Body in dorsal view **C** Head in full-face view **D** Genital capsule in ventral view **E** Abdominal segment IX (subgenital plate) **F** Wing venation. Scale bar equals 1.0 mm in **A, B**, and **F**, 0.5 mm in **C–E**. Photographs **A–C** courtesy of www.antweb.org (Michele Esposito).

**Figure 51.**
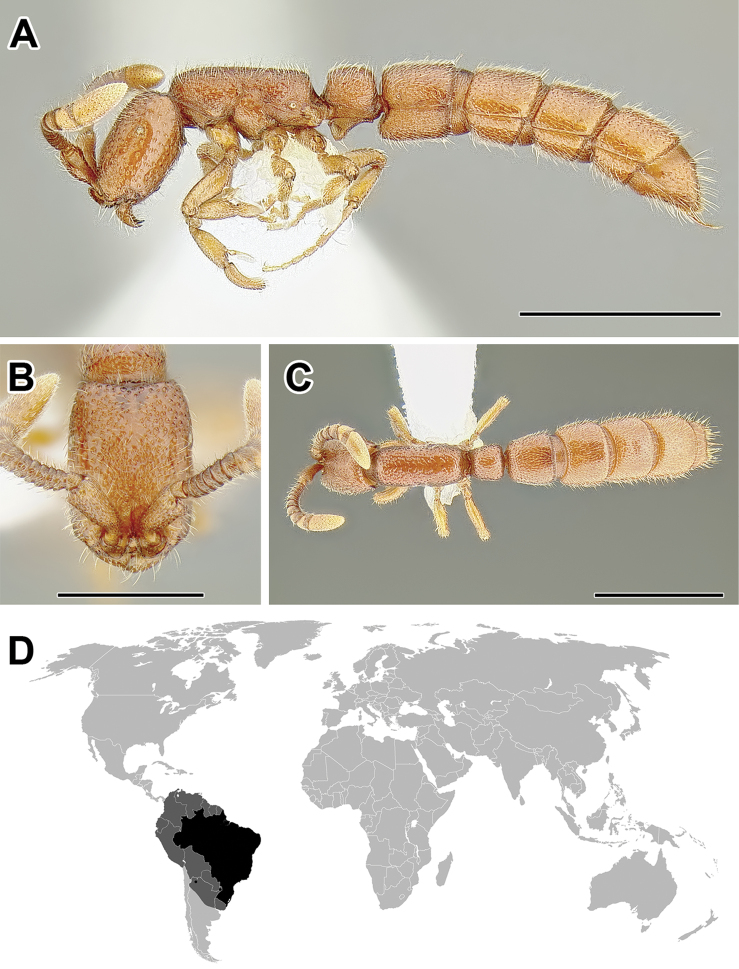
**A–C** Worker of Sphinctomyrmex cf. marcoyi (CASENT0731146) **A** Body in lateral view **B** Head in full-face view **C** Body in dorsal view **D** World distribution of *Sphinctomyrmex* (black: present, dark grey: likely present). Scale bar equals 1.0 mm **A** and **C**, 0.5 mm in **B**.

**Figure 52.**
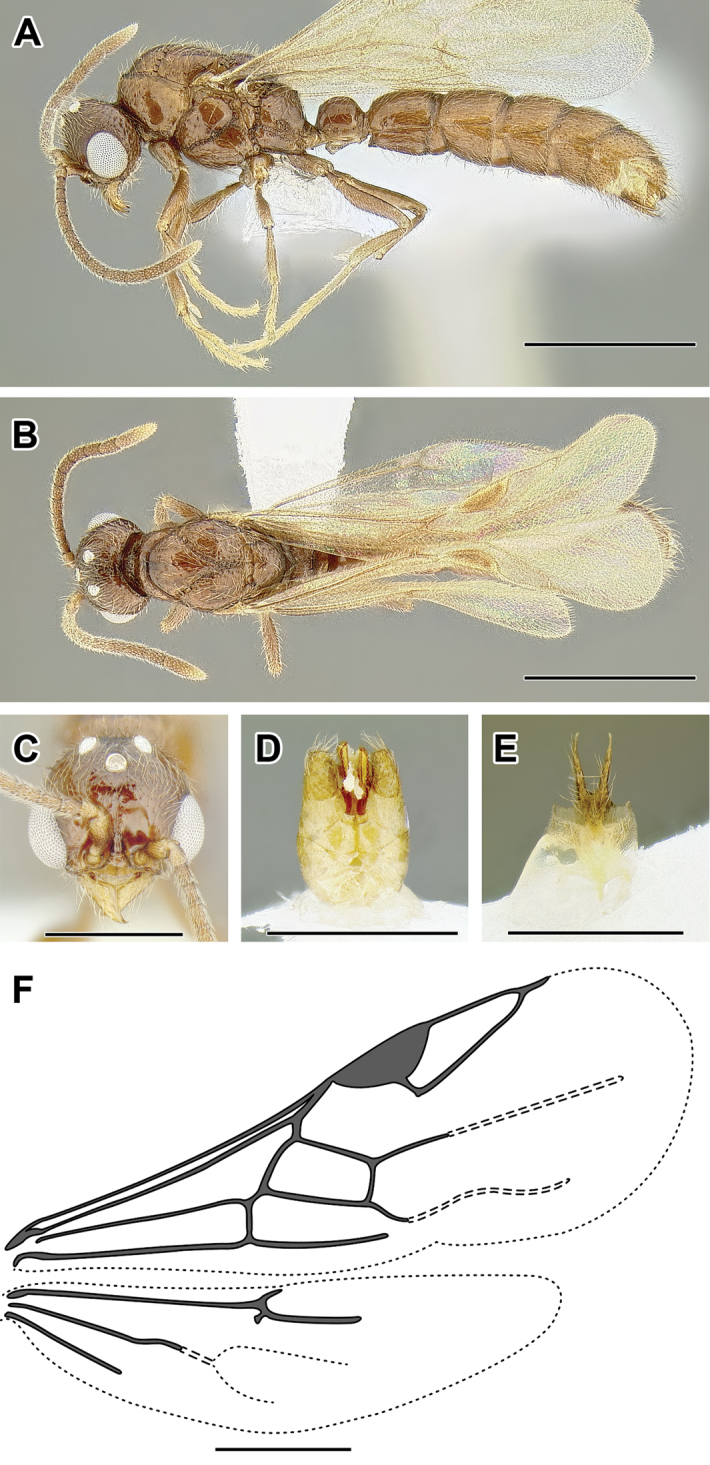
**A–F** Male of *Sphinctomyrmex* sp. (CASENT0731118) **A** Body in lateral view **B** Body in dorsal view **C** Head in full-face view **D** Genital capsule in ventral view **E** Abdominal segment IX (subgenital plate) **F** Wing venation. Scale bar equals 1.0 mm in **A** and **B**, 0.5 mm in **C–F**.

**Figure 53.**
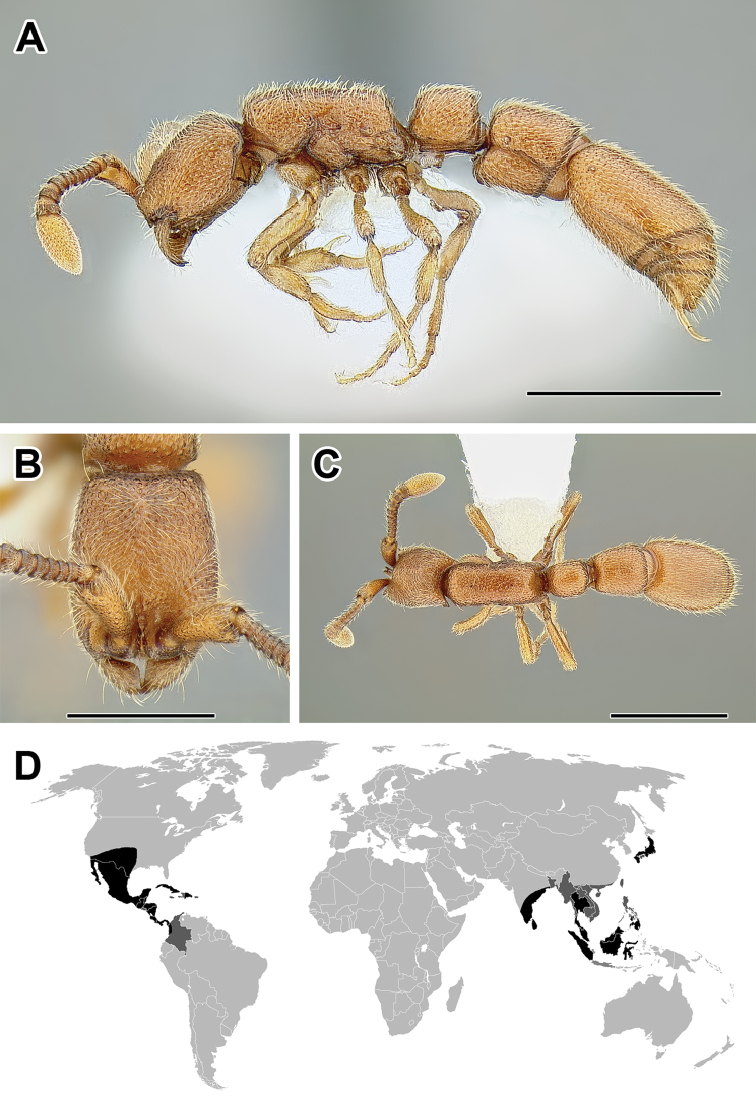
**A–C** Worker of *Syscia augustae* (CASENT0731214) **A** Body in lateral view **B** Head in full-face view **C** Body in dorsal view **D** World distribution of *Syscia* (black: present, dark grey: likely present). Scale bar equals 1.0 mm in **A** and **C**, 0.5 mm in **B**.

**Figure 54.**
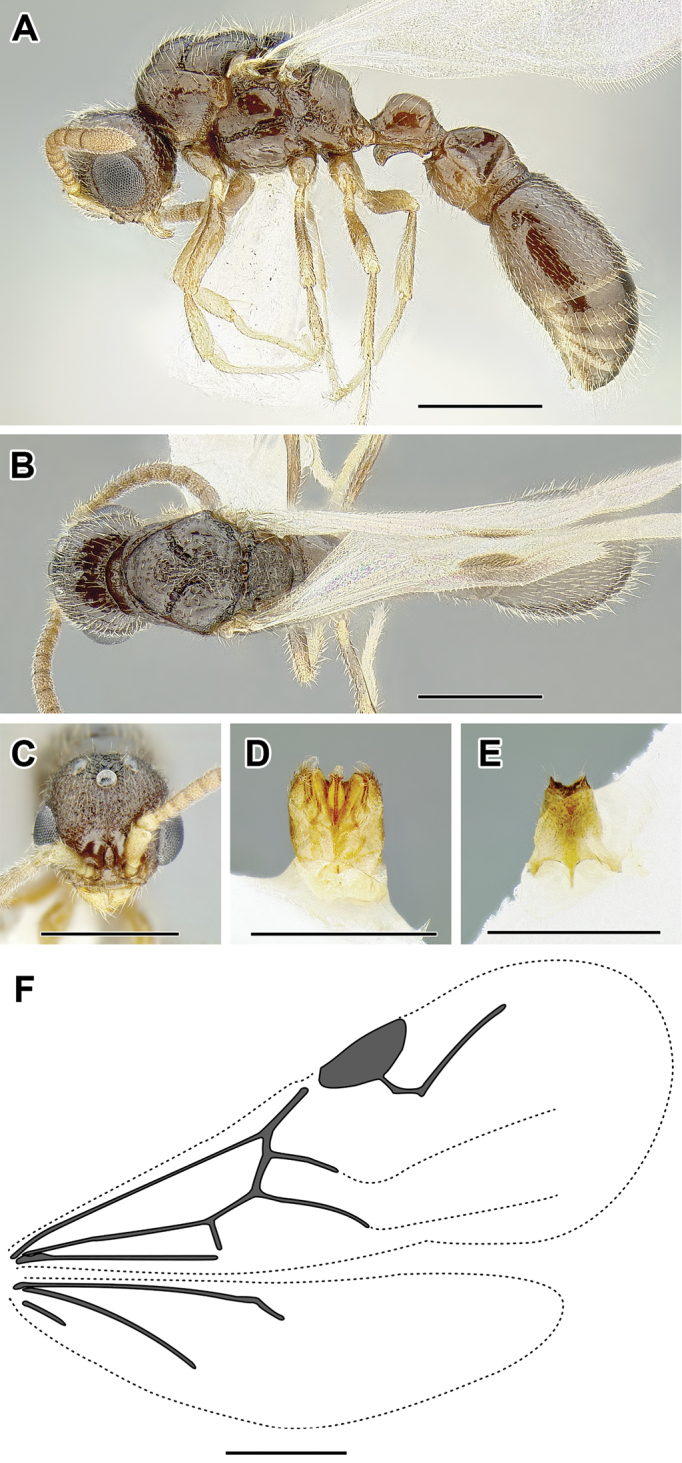
**A–F** Male of *Syscia humicola* (**A–C** CASENT0731213), *Syscia* sp. (**D–F** CASENT0731104) **A** Body in lateral view **B** Body in dorsal view **C** Head in full-face view **D** Genital capsule in ventral view **E** Abdominal segment IX (subgenital plate) **F** Wing venation. Scale bar equals 0.5 mm.

**Figure 55.**
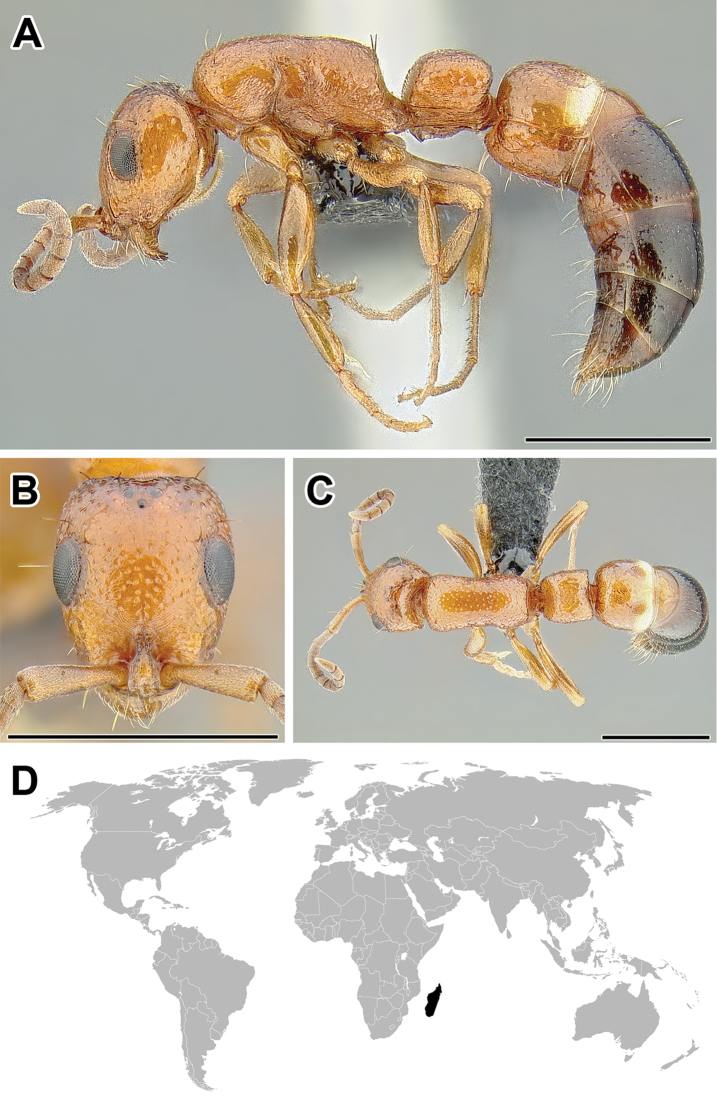
**A–C** Worker of *Tanipone aversa* (CASENT0207895) **A** Body in lateral view **B** Head in full-face view **C** Body in dorsal view **D** World distribution of *Tanipone* (black: present, dark grey: likely present). Scale bar equals 1.0 mm.

**Figure 56.**
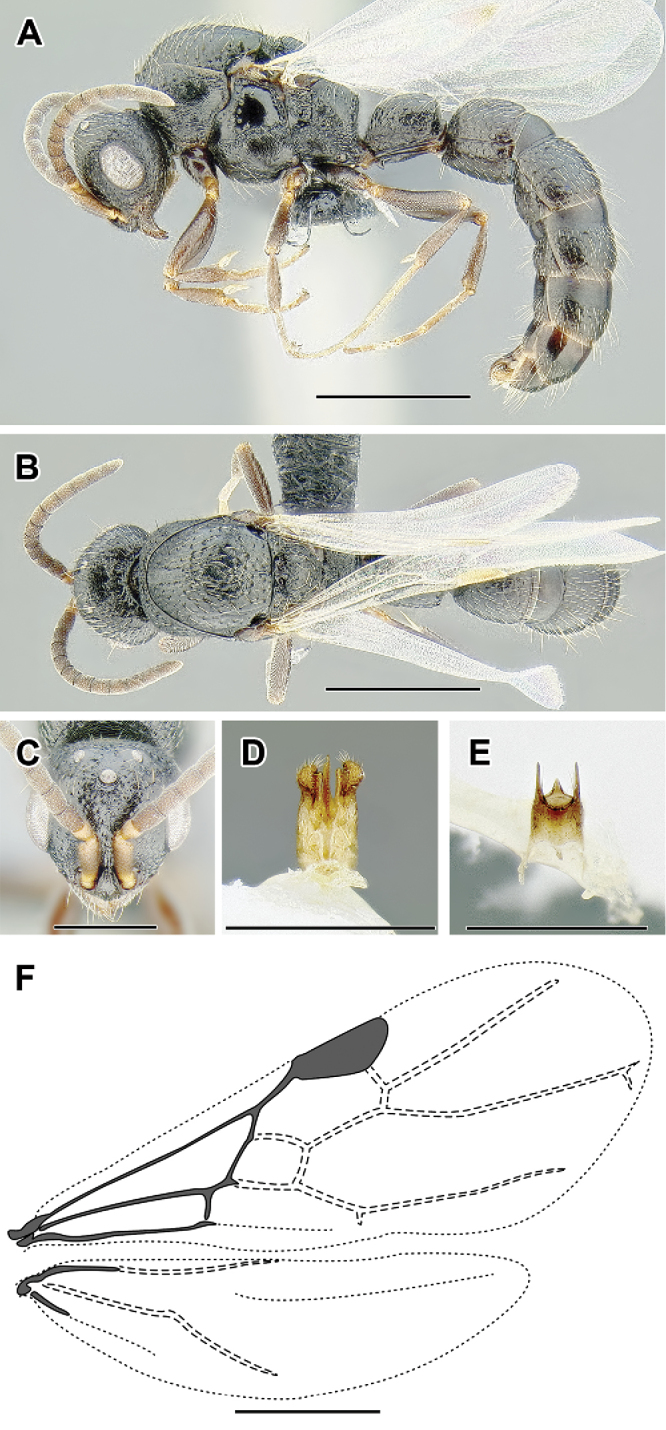
**A–F** Male of *Tanipone* sp. (**A–C** CASENT0154714 **D** and **E** CASENT0731105 **F** CASENT0217353) **A** Body in lateral view **B** Body in dorsal view **C** Head in full-face view **D** Genital capsule in ventral view **E** Abdominal segment IX (subgenital plate) **F** Wing venation. Scale bar equals 1.0 mm in **A, B**, and **F**, 0.5 mm in **C–E**.

**Figure 57.**
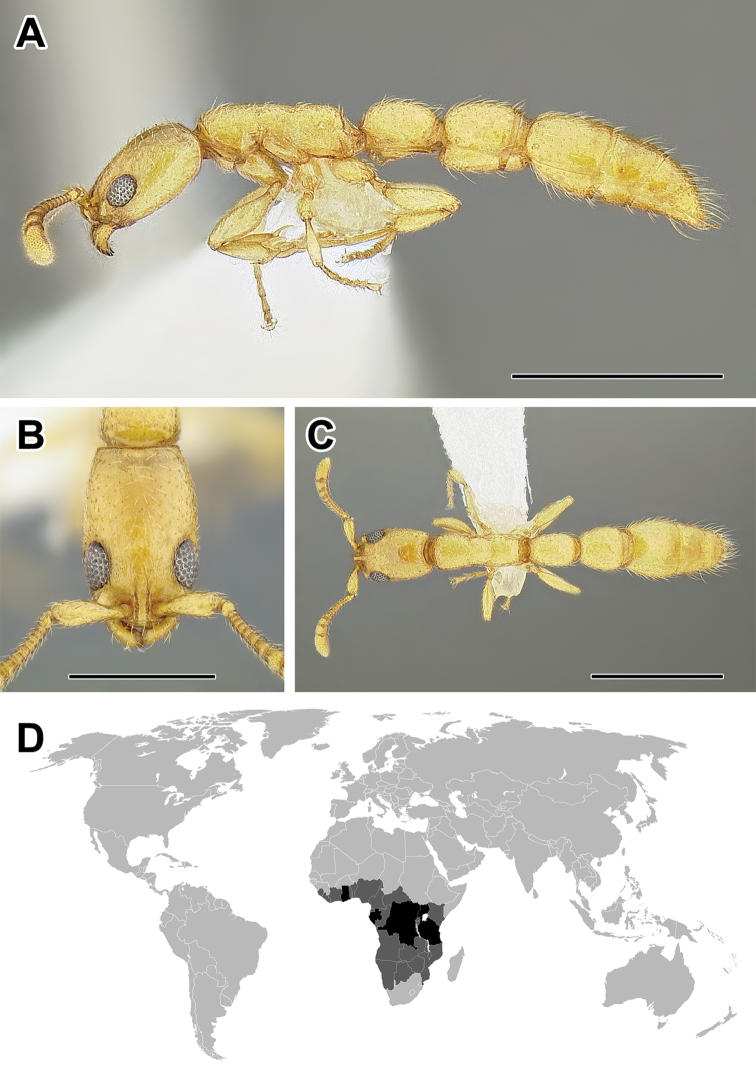
**A–C** Worker of *Vicinopone conciliatrix* (CASENT0731137) **A** Body in lateral view **B** Head in full-face view **C** Body in dorsal view **D** World distribution of *Vicinopone* (black: present, dark grey: likely present). Scale bar equals 1.0 mm.

**Figure 58.**
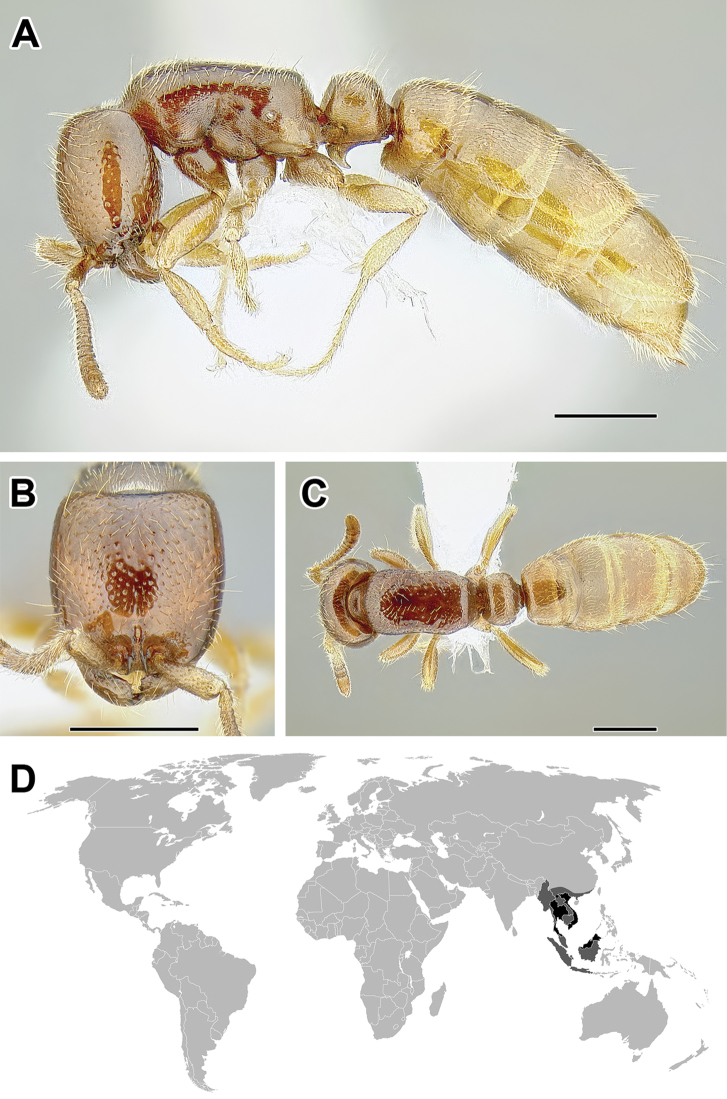
**A–C** Worker of *Yunodorylus eguchii* (CASENT0731166) **A** Body in lateral view **B** Head in full-face view **C** Body in dorsal view **D** World distribution of *Yunodorylus* (black: present, dark grey: likely present). Scale bar equals 0.5 mm.

**Figure 59.**
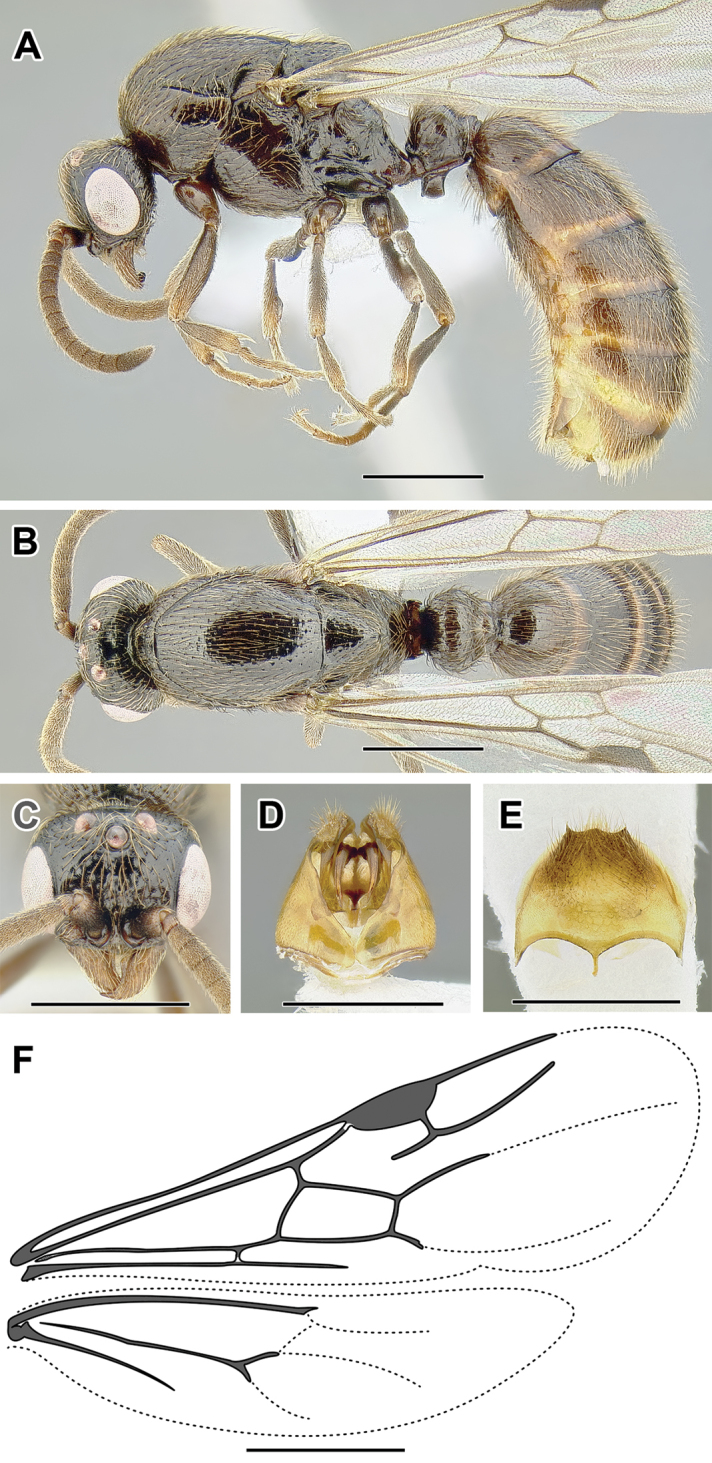
**A–F** Male of *Yunodorylus* sp. (CASENT0278751) **A** Body in lateral view **B** Body in dorsal view **C** Head in full-face view **D** Genital capsule in ventral view **E** Abdominal segment IX (subgenital plate) **F** Wing venation. Scale bar equals 1.0 mm.

**Figure 60.**
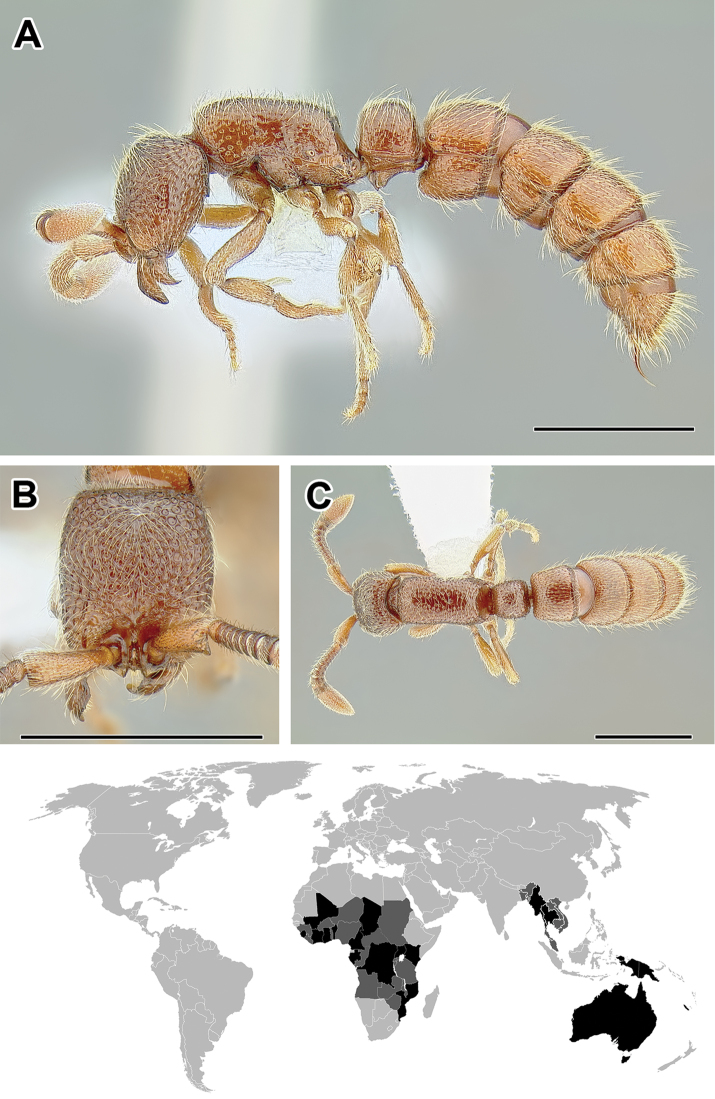
**A–C** Worker of *Zasphinctus trux* (CASENT0731216) **A** Body in lateral view **B** Head in full-face view **C** Body in dorsal view **D** World distribution of *Zasphinctus* (black: present, dark grey: likely present). Scale bar equals 1.0 mm.

**Figure 61.**
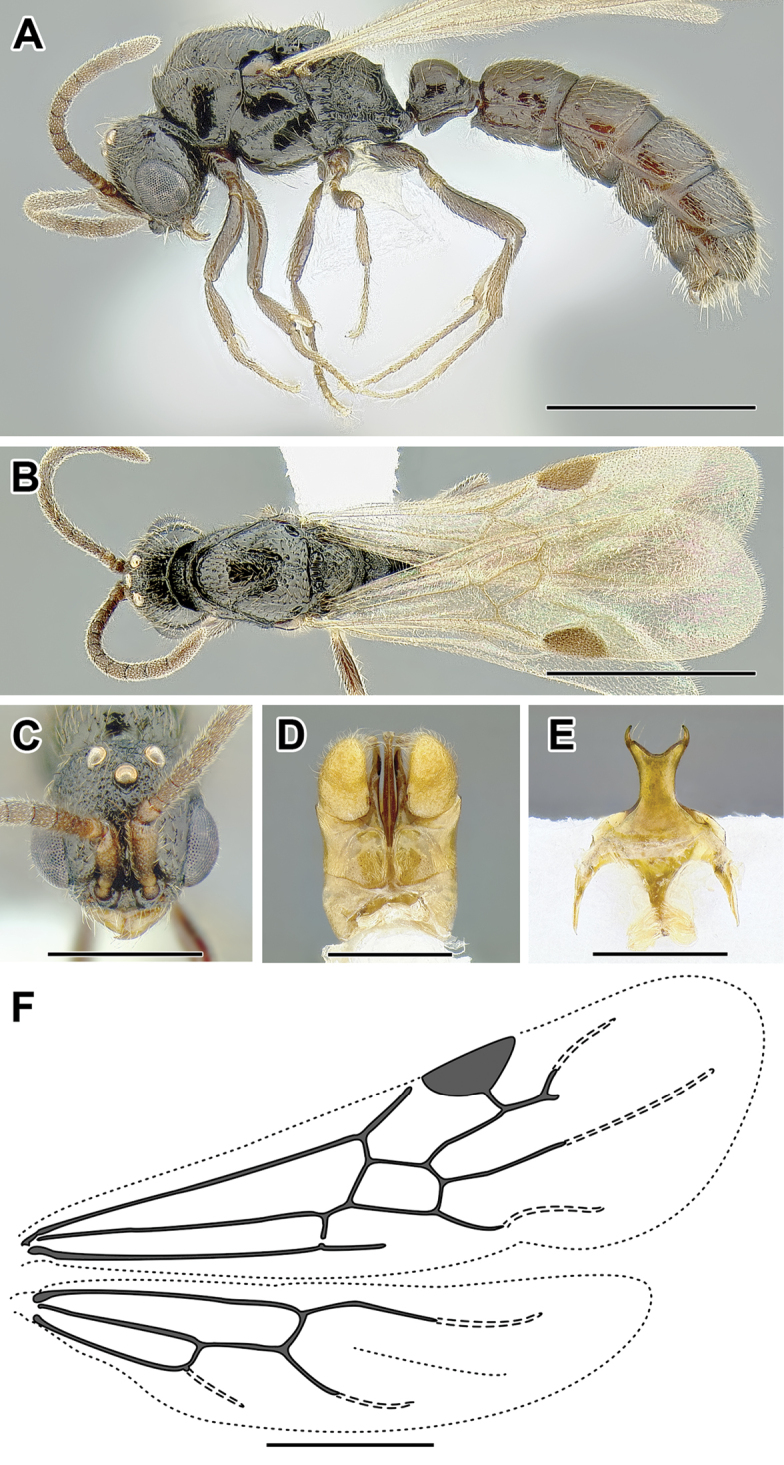
A–F Male of *Zasphinctus* sp. (A–C: CASENT0731115, D–F: CASENT0731106) **A** Body in lateral view **B** Body in dorsal view **C** Head in full-face view **D** Genital capsule in ventral view **E** Abdominal segment IX (subgenital plate) **F** Wing venation. Scale bar equals 1.0 mm in **A, B**, and **F**, 0.5 mm in **C–E**.

### Key to the genera of doryline ants based on workers

Certain couplets build upon keys in Gotwald (1982), Bolton (1994), and Bolton and Fisher (2012). Figure pointers refer to plate following couplet.

**Table:** 

1	Last visible abdominal tergite, the pygidium, not armed with numerous modified setae, at most with only one or two pairs of thick setae or cuticular projections (Figures A, B). Propodeal lobes short or absent	**2**
–	Pygidium armed with numerous specialized, peg-like or spiniform setae much thicker than surrounding fine hairs (Figure C); setae more than four in number, often more numerous. If pygidium small or with few specialized setae, then propodeal lobes conspicuous	**11**
	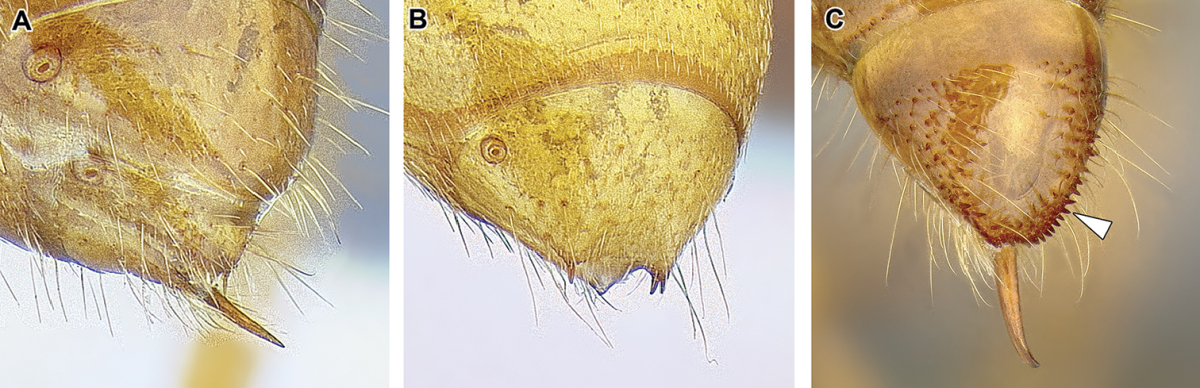	
2 (1)	Propodeal spiracles positioned low on propodeum, at or below mid-height of the sclerite (Figure A)	**3**
–	Propodeal spiracles positioned high on propodeum, above mid-height of the sclerite (Figures B, C)	**4**
	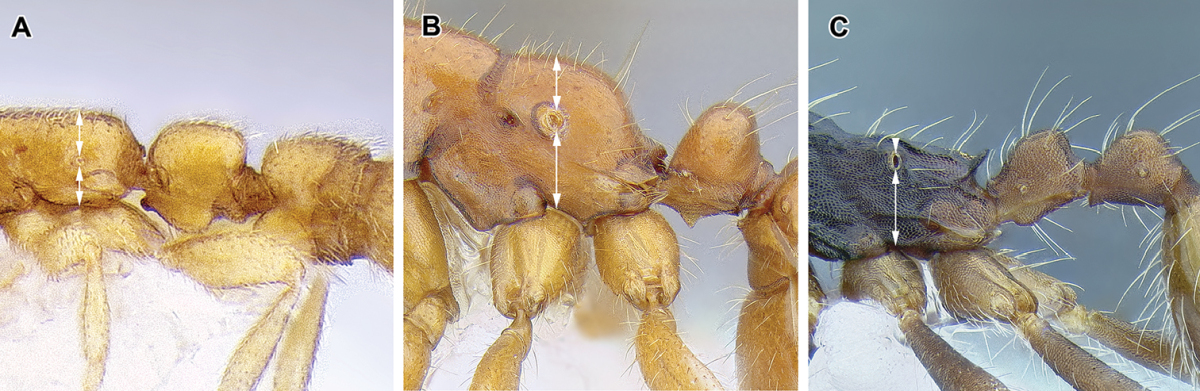	
3 (2)	Pygidium large. Propodeal lobes present (Baltic amber)	***Procerapachys***
–	Pygidium small. Propodeal lobes absent (Nearctic, Neotropical)	***Leptanilloides***
4 (2)	Abdominal segment II (petiole) and segment III differentiated and both segments much smaller than the succeeding segment IV. Abdominal segment IV always conspicuously the largest segment (Figure A).	**5**
–	Only abdominal segment II (petiole) differentiated and smaller than succeeding segments III and IV (Figure B). If abdominal segment III attached to segment IV through a strong constriction and somewhat differentiated, then abdominal segment IV not conspicuously the largest segment.	**9**
	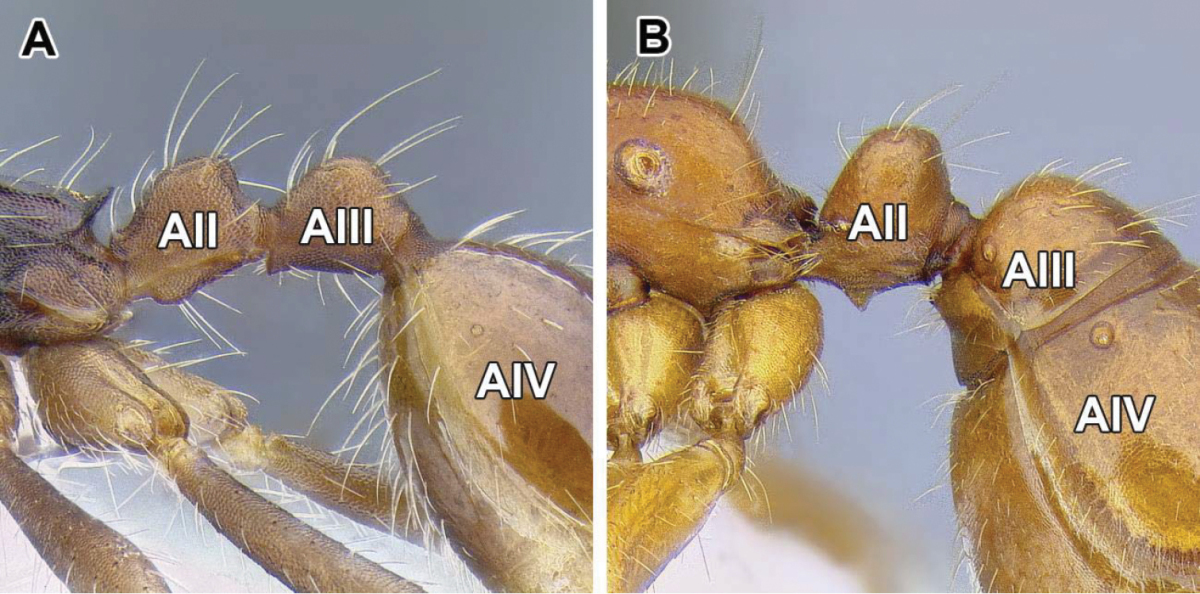	
5 (4)	Antennae with 8–10 segments. Old World species (Figure A) (Palearctic, Afrotropical, Indomalayan, Australasian)	***Aenictus***
–	Antennae with 12 segments (Figure B). New World species	**6**
	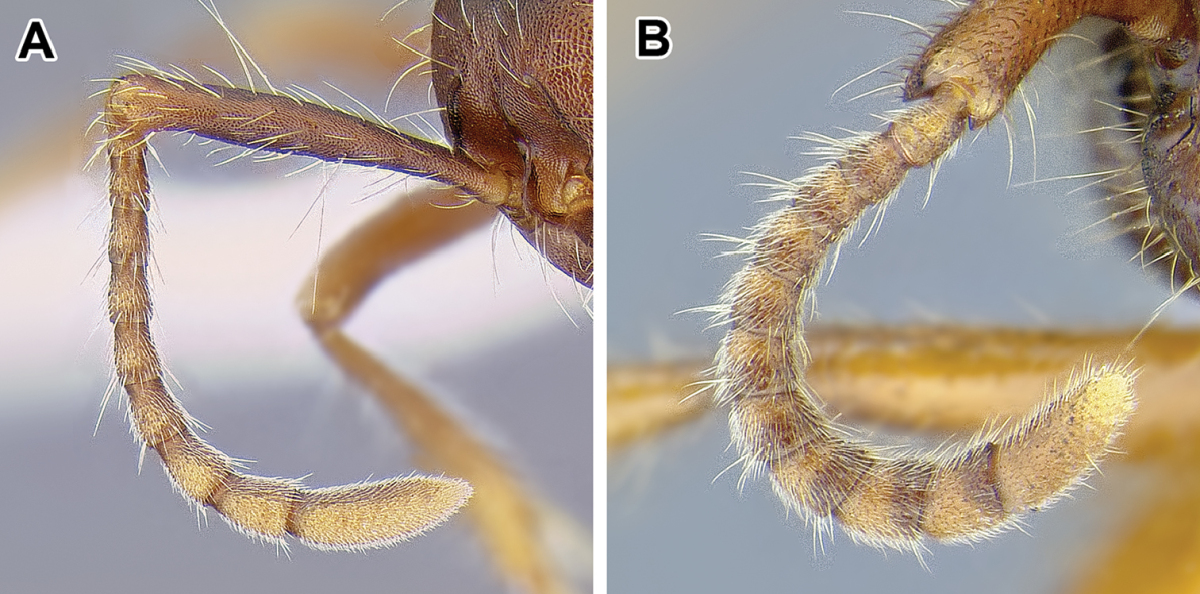	
6 (5)	Tarsal claws simple, without teeth (Figure A) (Nearctic, Neotropical, Dominican amber)	***Neivamyrmex***
–	Tarsal claws armed with teeth (Figure B)	**7**
	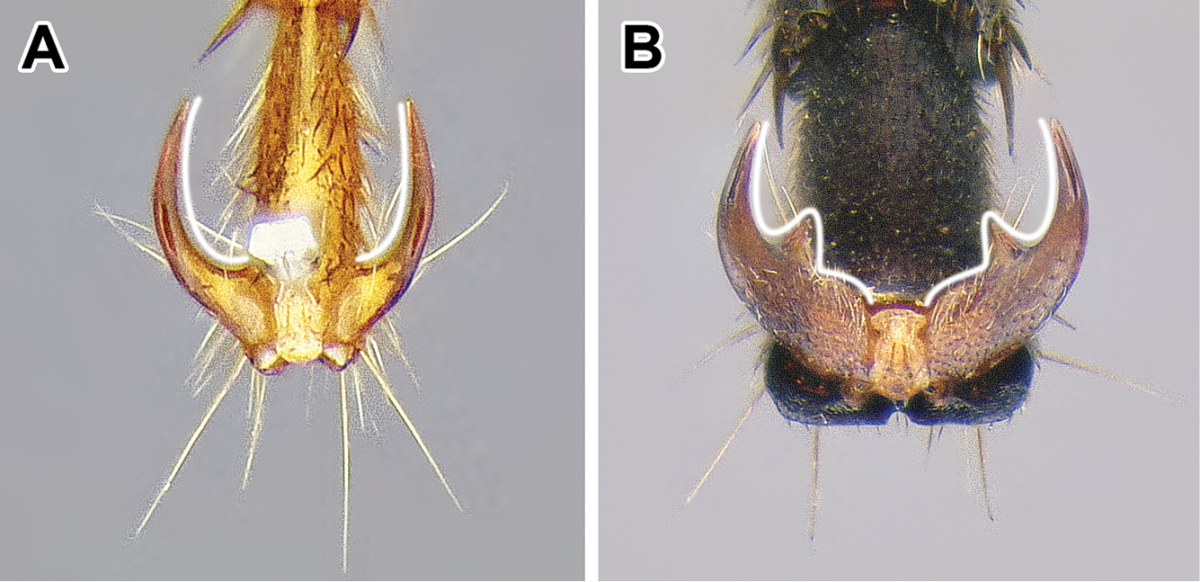	
7 (6)	Inner (flexor) surface of hind tibiae without any sign of differentiated pale cuticle (Figure A) (Nearctic, Neotropical)	***Nomamyrmex***
–	Inner surface of hind tibiae with differentiated surface of pale cuticle (metatibial gland), from elongately oval patch near tibial spur to a narrow stripe spanning much of the length of tibia (Figure B)	**8**
	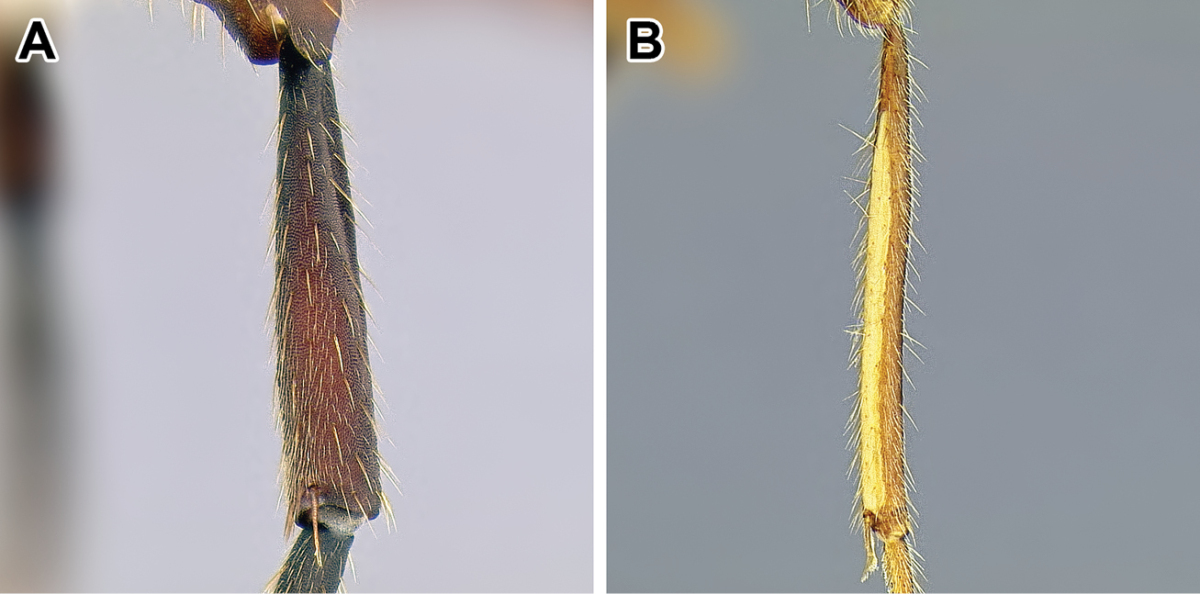	
8 (7)	Propodeum armed with cuticular lamellae or spines (Figure A) (Neotropical)	***Eciton***
–	Propodeum unarmed, dorsal propodeal surface rounding into propodeal declivity (Figure B) (Nearctic, Neotropical)	***Labidus***
	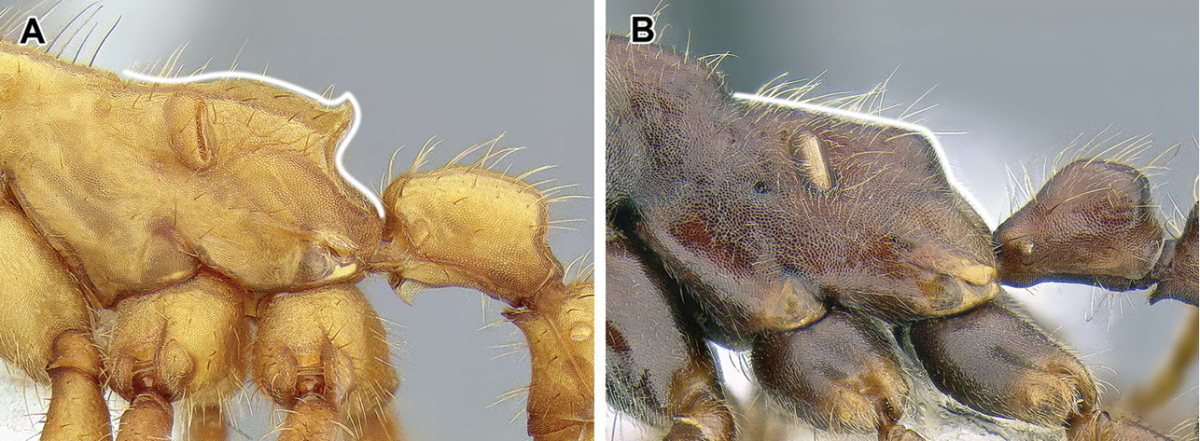	
9 (4)	Constrictions present at anterior end of abdominal segments V and VI (Figure A) (Afrotropical)	***Aenictogiton***
–	Constrictions absent from anterior end of abdominal segments V and VI (Figure B)	**10**
	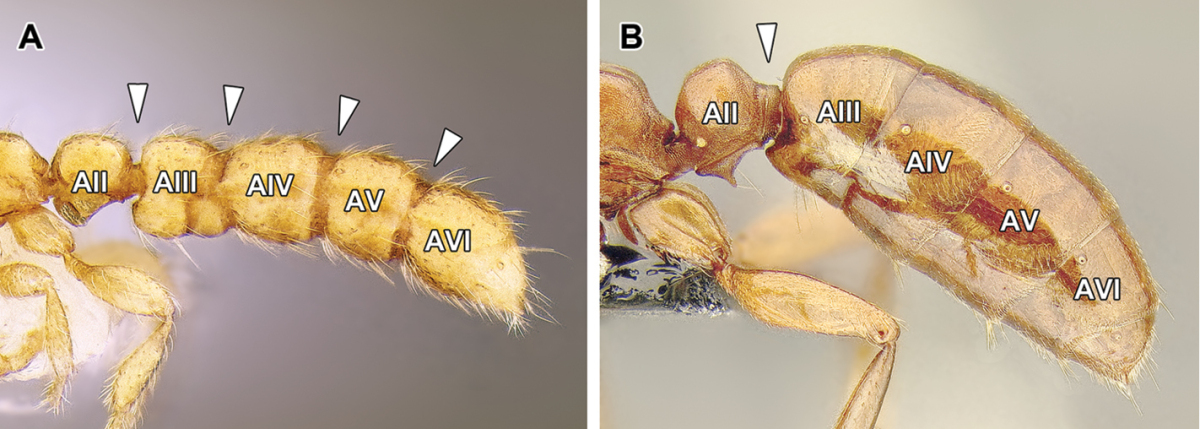	
10 (9)	Promesonotal Pronotomesopleural suture conspicuous (Figure A). Pygidium large and impressed at apex, armed with one or two cuticular teeth or spines on each side (Figure C). Pretarsal claws unarmed (Palearctic, Afrotropical, Indomalayan)	***Dorylus***
–	Promesonotal Pronotomesopleural suture absent (Figure B). Pygidium small and convex at apex, unarmed or with one or two peg-like setae on each side (Figure D). Pretarsal claws armed with teeth (Neotropical)	***Cheliomyrmex***
	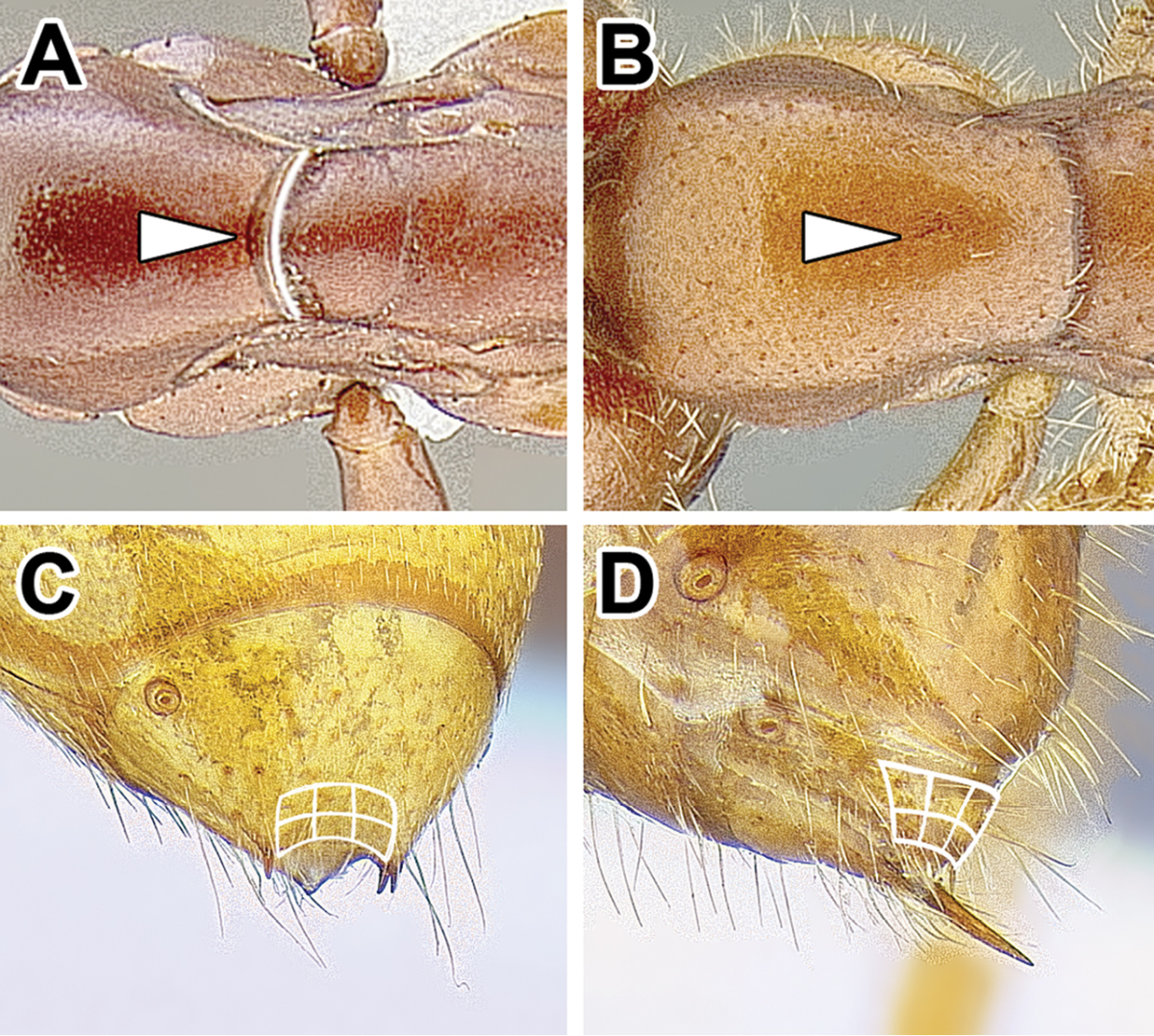	
11 (1)	Waist consisting only of abdominal segment II (petiole) and abdominal segment III broadly attached to segment IV, without conspicuous constrictions between pre- and postsclerites of abdominal segment IV (Figure A) (Indomalayan)	***Yunodorylus***
–	Waist with abdominal segment III at least weakly differentiated from segment IV; the latter with a constriction between its pre- and postsclerites (Figure B)	**12**
	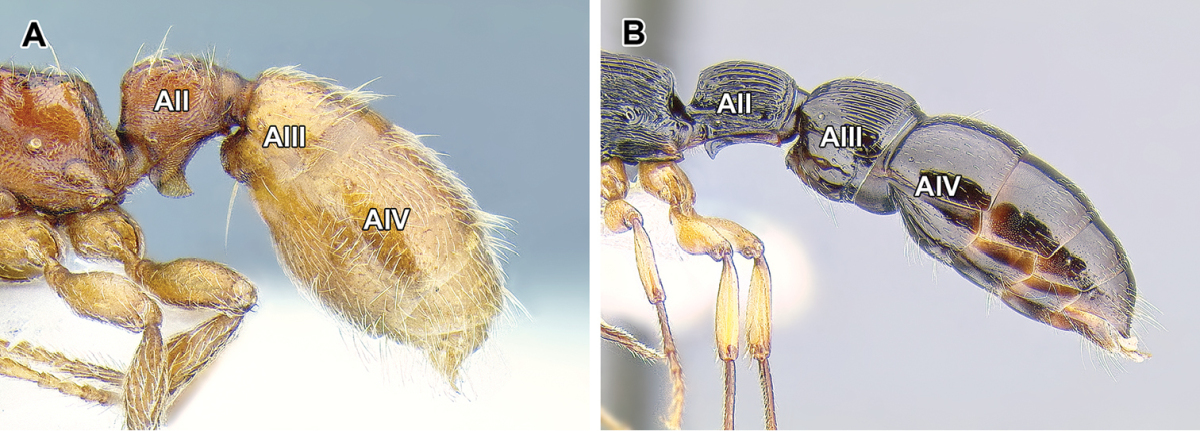	
12 (11)	Mid and hind tibiae each with two spurs (Figure A)	**13**
–	Middle tibiae with a single spur (Figure B) or without spurs (Figure C) and hind tibiae always with a single spur	**14**
	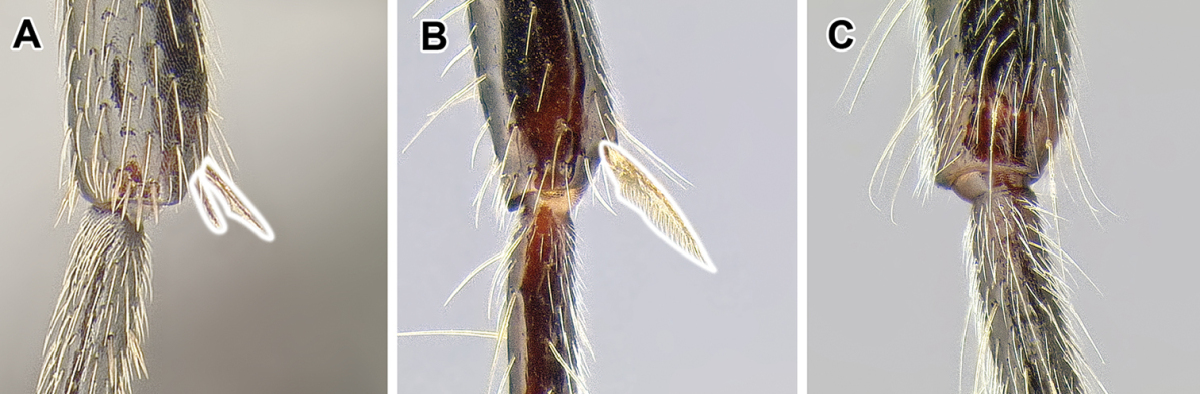	
13 (12)	Antennal sockets at least partly concealed in full face view (Figure A). Pretarsal claws simple (Figure C). Maxillary palps 2-segmented, labial palps 3-segmented (Neotropical, Dominican amber)	***Cylindromyrmex***
–	Antennal sockets exposed in full face view (Figure B). Pretarsal claws armed with a tooth (Figure D). Maxillary palps 5-segmented, labial palps 3-segmented (Malagasy, Indomalayan, Baltic amber)	***Chrysapace***
	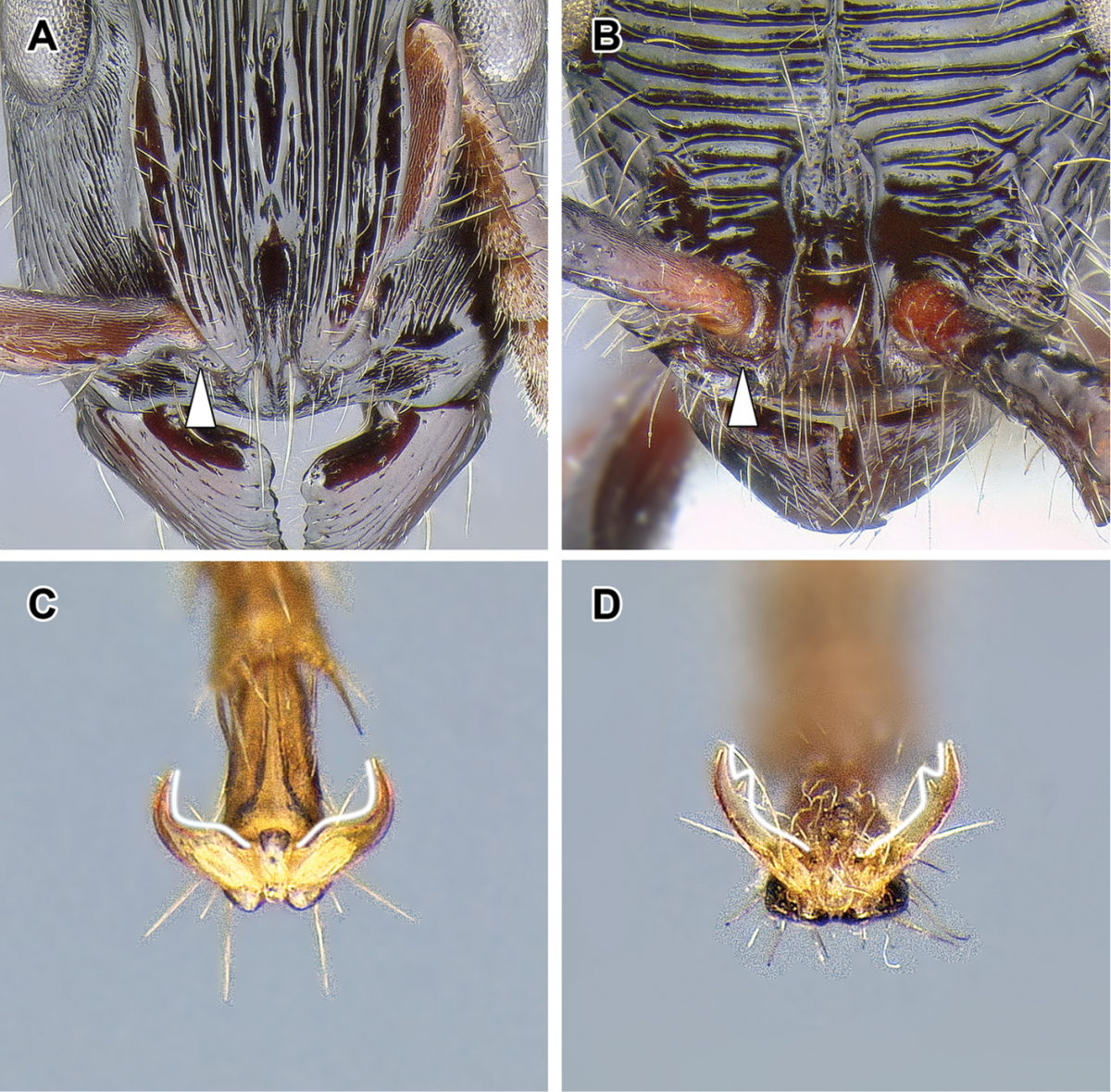	
14 (12)	Pretarsal claws of hind leg armed ventrally with a tooth or at least a small denticle (Figure A); teeth can be difficult to discern below 50× magnification	**15**
–	Pretarsal claws of hind leg simple (Figure B)	**18**
	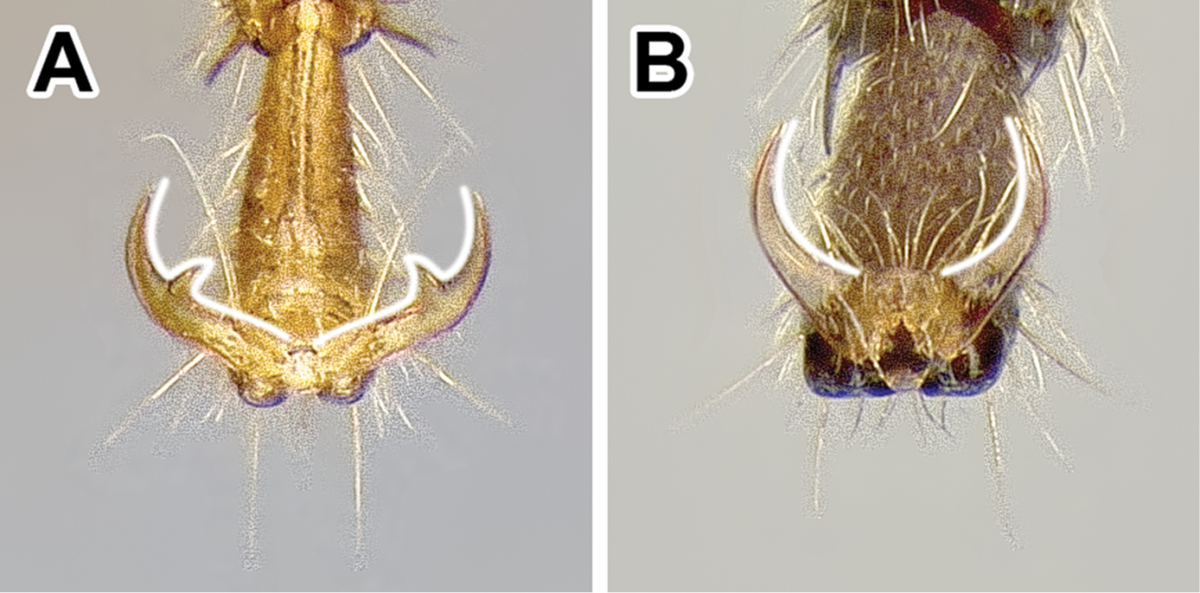	
15 (14)	Middle tibiae always with a pectinate spur. Hind tibiae with a patch light cuticle near distal end (Figure A; metatibial gland) (Indomalayan)	***Cerapachys*** (part)
–	Middle tibiae without spurs. Hind tibiae without a patch differentiated cuticle, but a conspicuous sulcus or groove on hind basitarsus may be present (Figure B; metabasitarsal gland)	**16**
	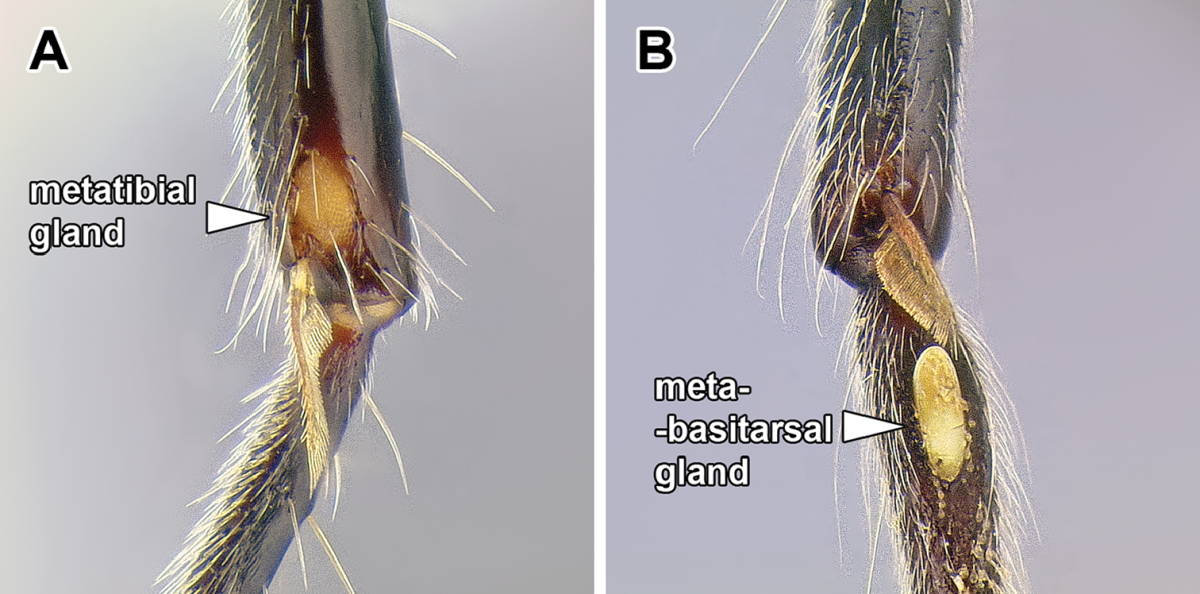	
16 (15)	Antennae with 11 segments (Figure A). Longitudinal glandular groove (metabasitarsal gland) present on basal half of ventral surface of hind basitarsi (Afrotropical, Malagasy, Indomalayan, Australasian)	***Simopone***
–	Antennae with 12 segments (Figures B, C). Longitudinal glandular groove (metabasitarsal gland) absent from basal half of ventral surface of hind basitarsi.	**17**
	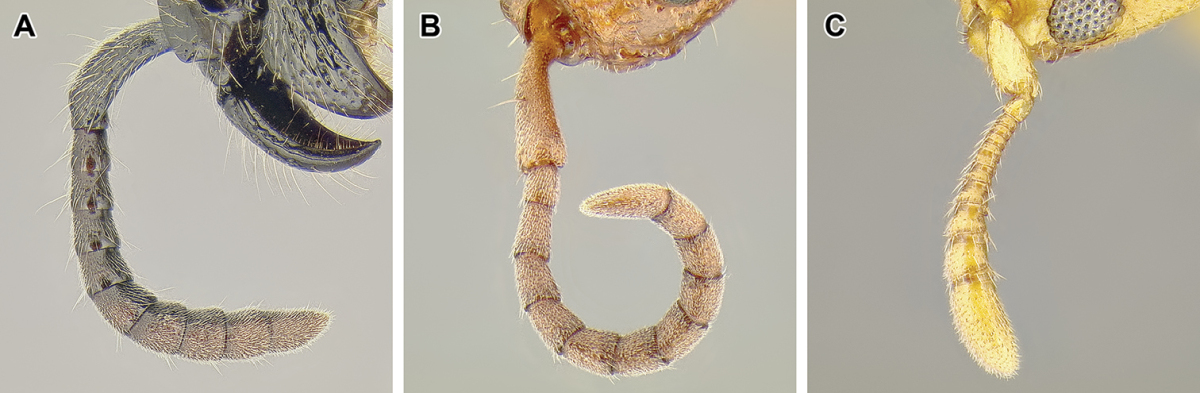	
17 (16)	Ocelli absent. Posterior margin of eyes anterior to midlength of head capsule (Figure A). Maxillary palps 3-segmented and labial palps 2-segments. Maxillary palps short and often not exposed in pinned specimens. When palps extended, the maxillary palp terminates well before occipital foramen (Figure C) (Afrotropical)	***Vicinopone***
–	Ocelli present. Posterior margin of eyes behind midlength of head capsule (Figure B). Maxillary palps 6-segmented and labial palps 4-segmented. Maxillary palps very long, when extended almost reaching occipital foramen (Figure D) (Malagasy)	***Tanipone***
	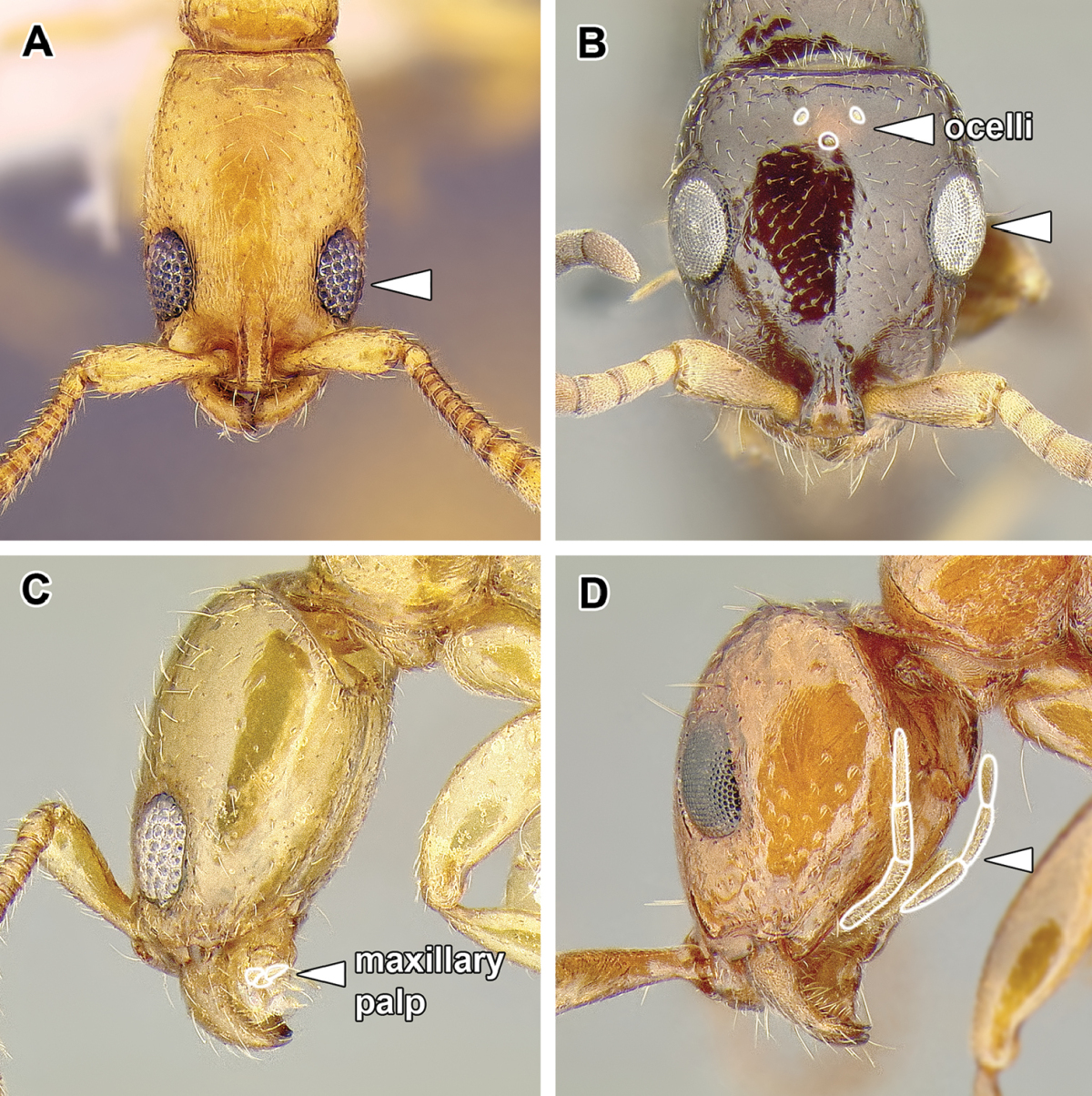	
18 (14)	At least anterior dorsolateral portions of abdominal segment II (petiole) marginate (Figure B) and often entire length of petiolar tergite with pronounced margins (Figure A). Hind coxa usually with posterior flange drawn into a vertical, opaque or semi-translucent lamella (Figure D). Metatibial gland pore plate usually in a depression or invagination of the cuticle, appearing as a slit (Figure F) or, more rarely, a circular opening (Figure G) or inconspicuous (Palearctic, Afrotropical, Malagasy, Indomalayan, Australasian)	***Lioponera***
–	No segment of body conspicuously dorsolaterally marginate although lateral crest immediately above abdominal segment II (petiolar) spiracle may be present (Figure C). If abdominal segment II appearing marginate, hind coxae without posterior vertical lamella (Figure E). Metatibial gland pore plate not in a depression, either an oval whitish patch (Figure H), or not discernable (Figure I)	**19**
	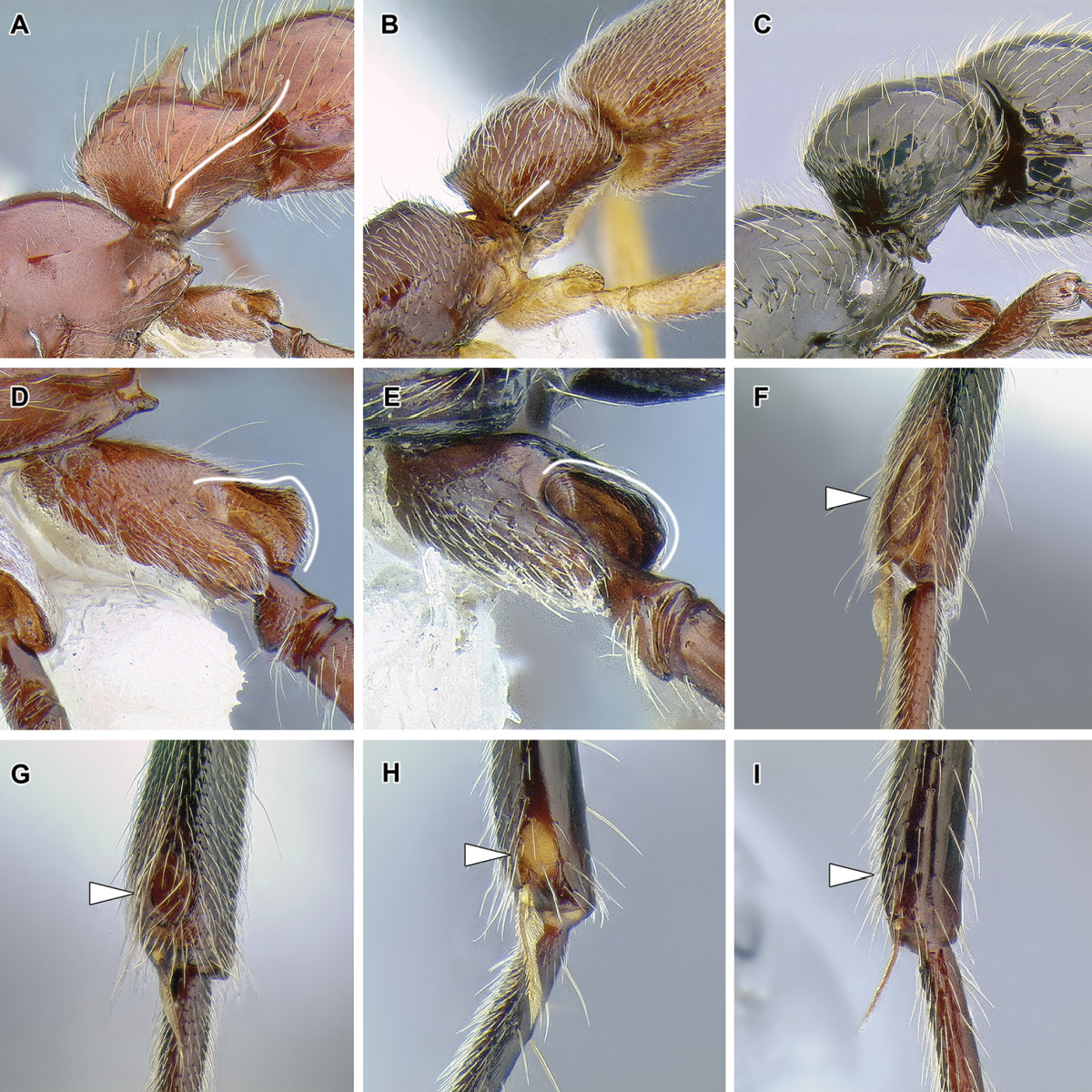	
19 (18)	In lateral view pronotomesopleural Pronotomesopleural suture either completely or partially fused, never a curved cut in cuticular surface approaching dorsolateral margins of promesonotum (Figures A, B). Sometimes in place of Pronotomesopleural suture a groove (especially *Neocerapachys* and *Sphinctomyrmex*) or a row of punctures present, or the Pronotomesopleural suture short; there is never a lining of short pubescence	**20**
–	In lateral view pronotomesopleural Pronotomesopleural suture present as a deep cut in the cuticle, often curved below dorsolateral margins of mesosoma and with inside lined with short pubescence (Figures C, D)	**24**
	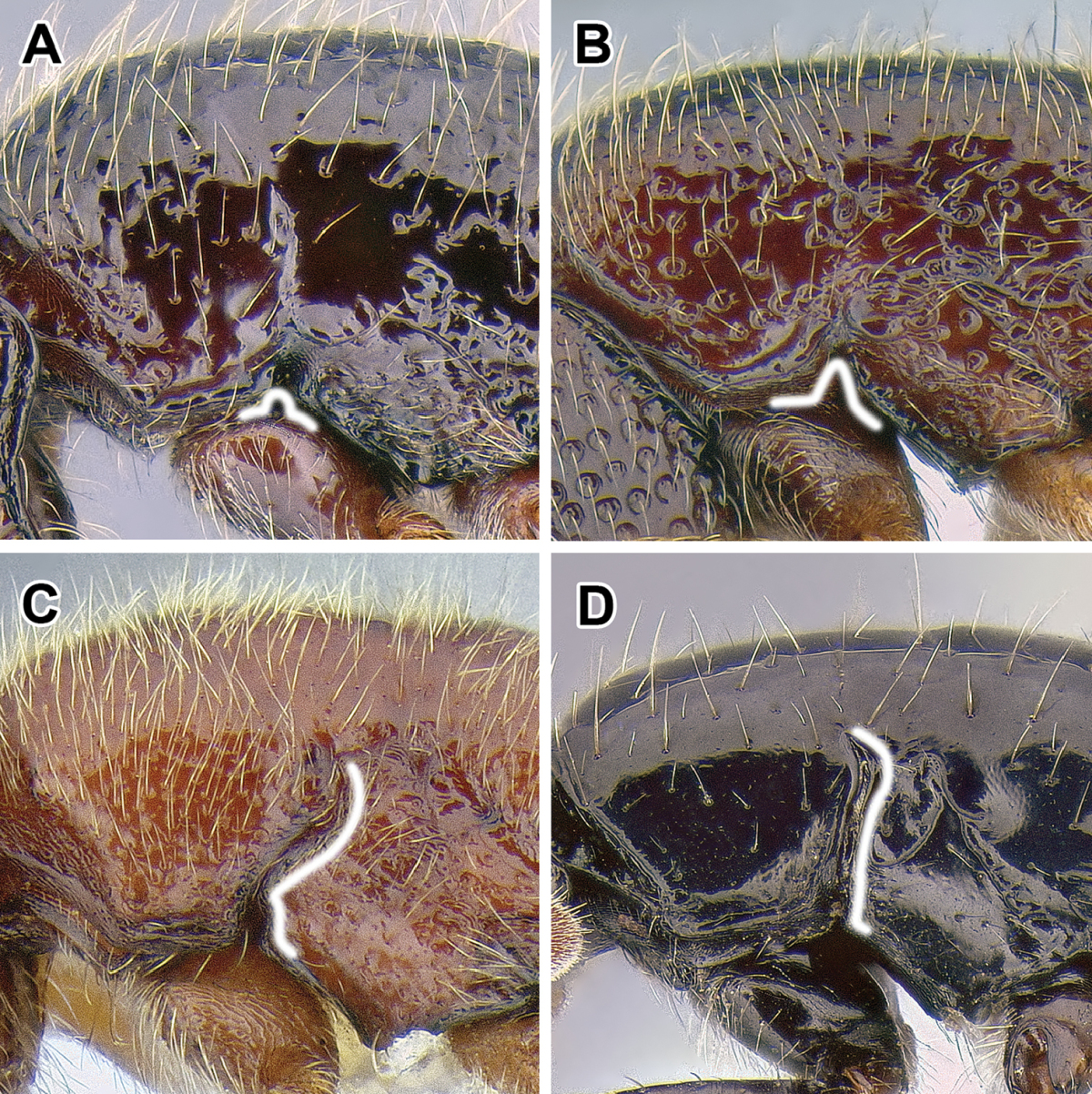	
20 (19)	Helcium circumference large relative to abdominal segment II (petiole) and placed above midheight of the segment, resulting in low, undifferentiated posterior face of abdominal segment II and low anterior face of abdominal segment III (Figure A) (Malagasy)	***Lividopone***
–	Helcium circumference small relative to abdominal segment II (petiole) and placed at about midheight of segment, resulting in pronounced posterior face to abdominal segment II and conspicuous anterior face of abdominal segment III (Figure B)	**21**
	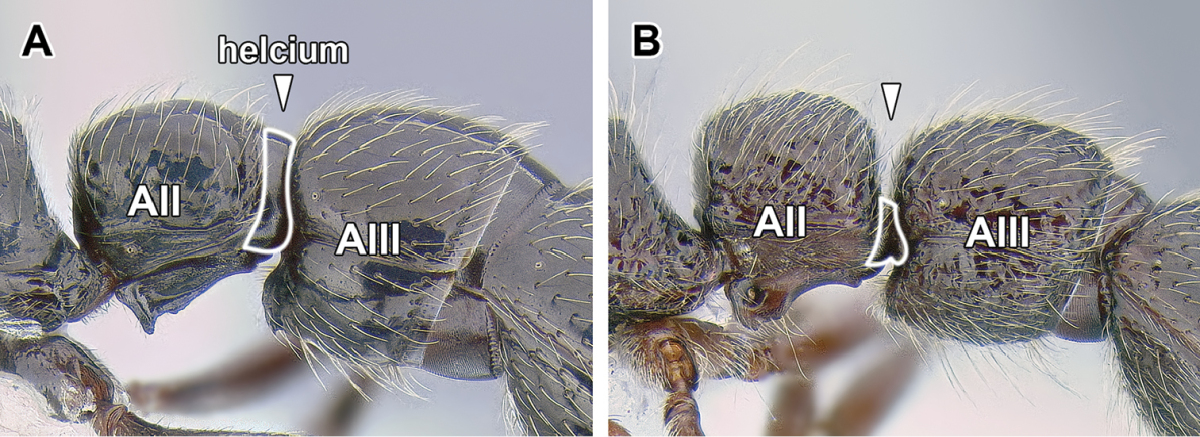	
21 (20)	Metapleural gland trench an inconspicuous narrow slit, with posterior opening smaller than the diameter of propodeal spiracle (Figure A) (Neotropical)	**22**
–	Metapleural gland trench conspicuous, throughout its length broader than the diameter of propodeal spiracle opening (Figure B). If the posterior opening of the trench narrow (rarely), it is through a constriction made by an elevated ventral flange of the trench, the latter being broad and deep anteriorly to the constriction (Old World)	**23**
	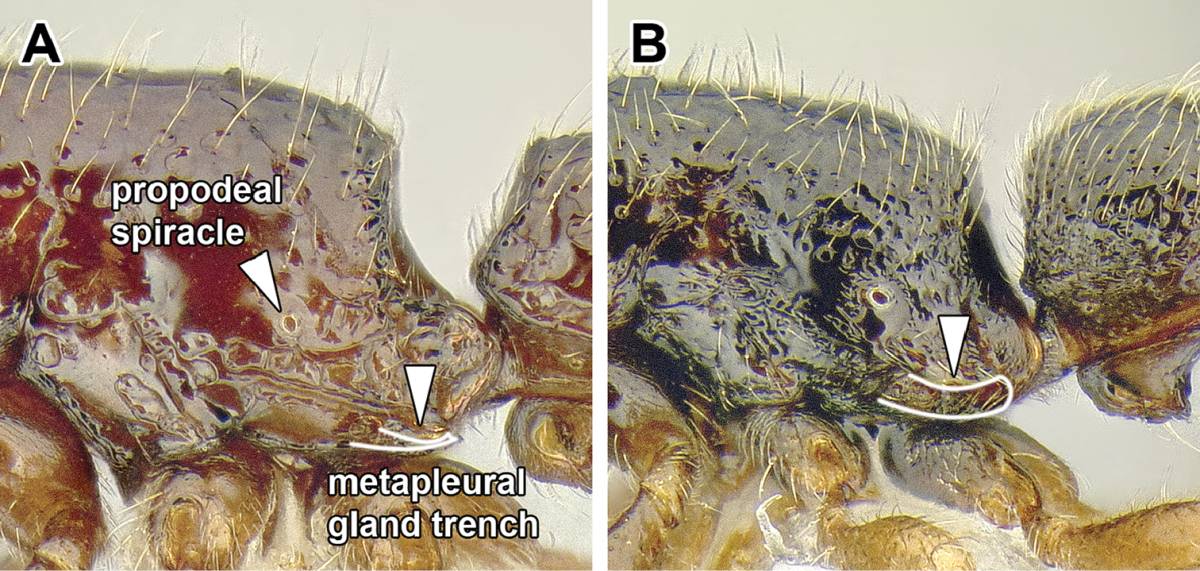	
22 (21)	Constrictions present at anterior end of abdominal segments V and VI (Figure A). No patches of differentiated cuticle on abdominal tergite IV (Figure C) (Neotropical)	***Sphinctomyrmex***
–	Constrictions absent from anterior end of abdominal segments V and VI (Figure B). Circular porous and pubescent patches differentiated from surrounding cuticle (occasionally indistinct) present laterally on abdominal tergite IV, just medial to the spiracles (Figure D) (Neotropical)	***Neocerapachys***
	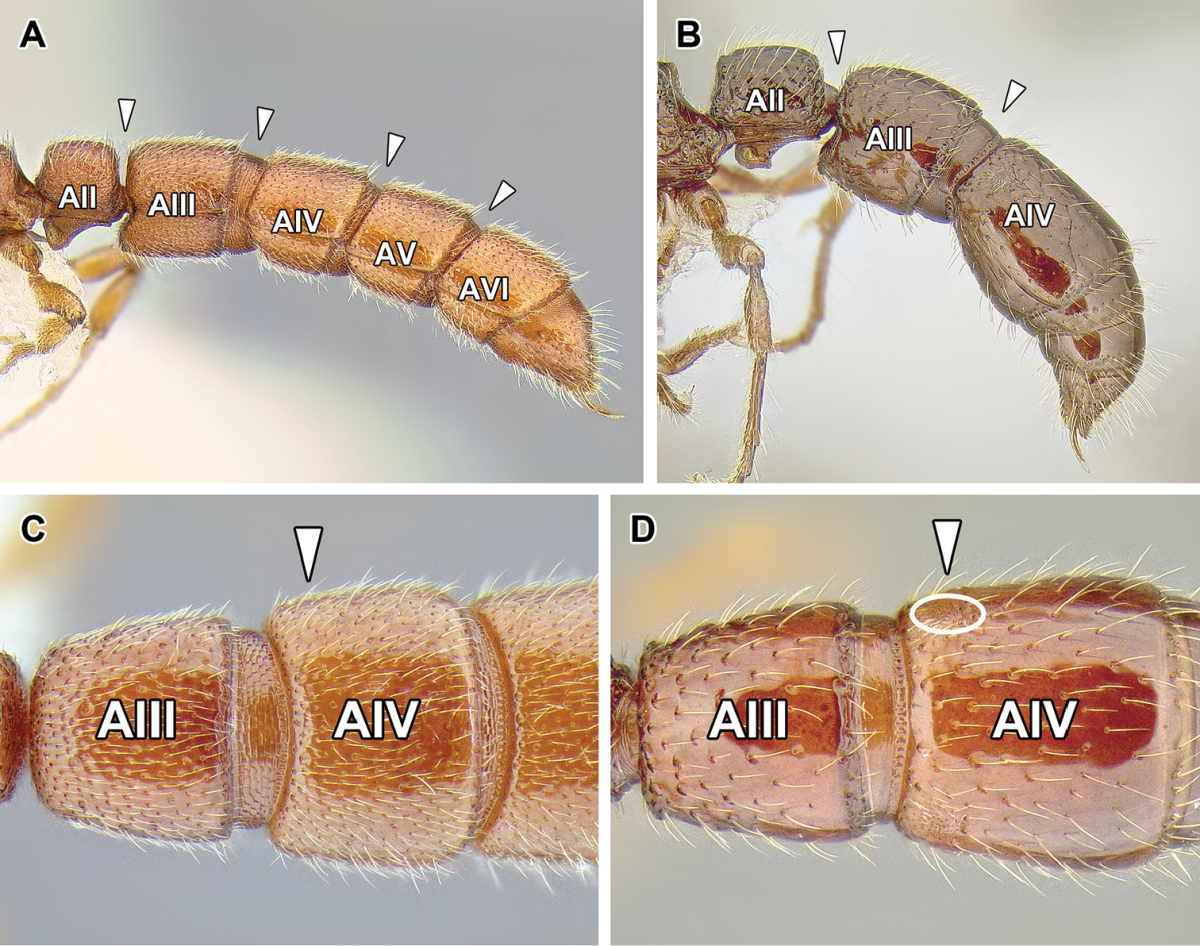	
23 (21)	Constrictions present at anterior end of abdominal segments V and VI (Figure A) (Afrotropical, Australasian)	***Zasphinctus***
–	Constrictions absent from anterior end of abdominal segments V and VI (Figure B) (Palearctic, Afrotropical, Malagasy, Indomalayan, Australasian)	***Parasyscia***
	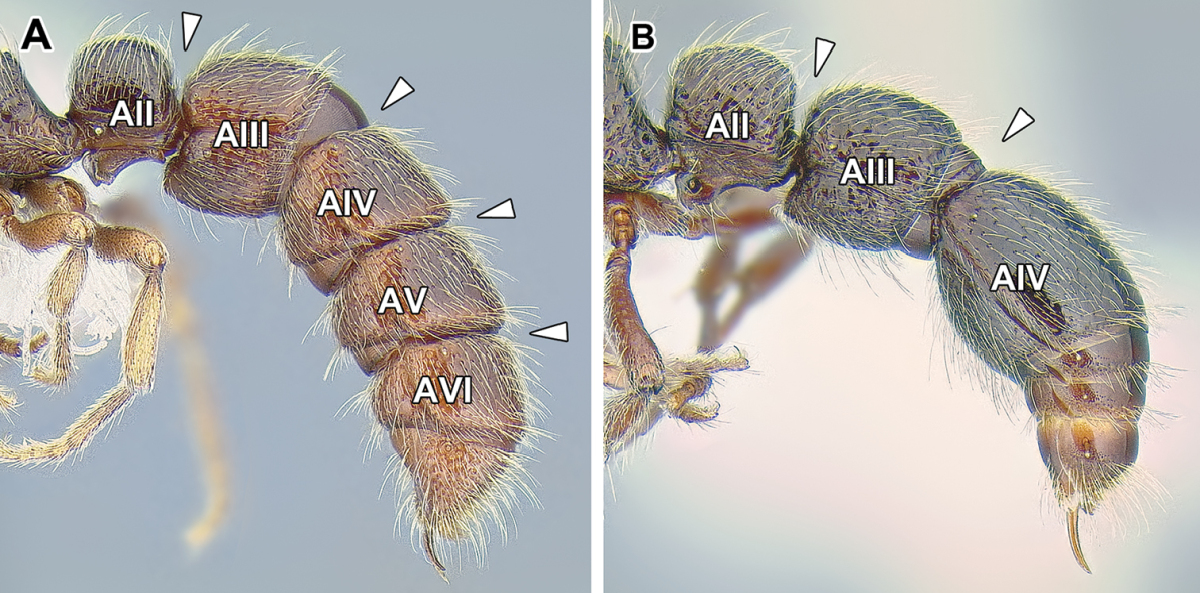	
24 (19)	Helcium circumference large relative to abdominal segment II (petiole) and placed above midheight of the segment, resulting in very low, undifferentiated posterior face of petiole and low anterior face of abdominal segment III (Figure A)	**25**
–	Helcium circumference small relative to abdominal segment II (petiole) placed at about midheight of segment, usually resulting in differentiated posterior face to abdominal segment II and conspicuous anterior face of abdominal segment III (Figure B)	**26**
	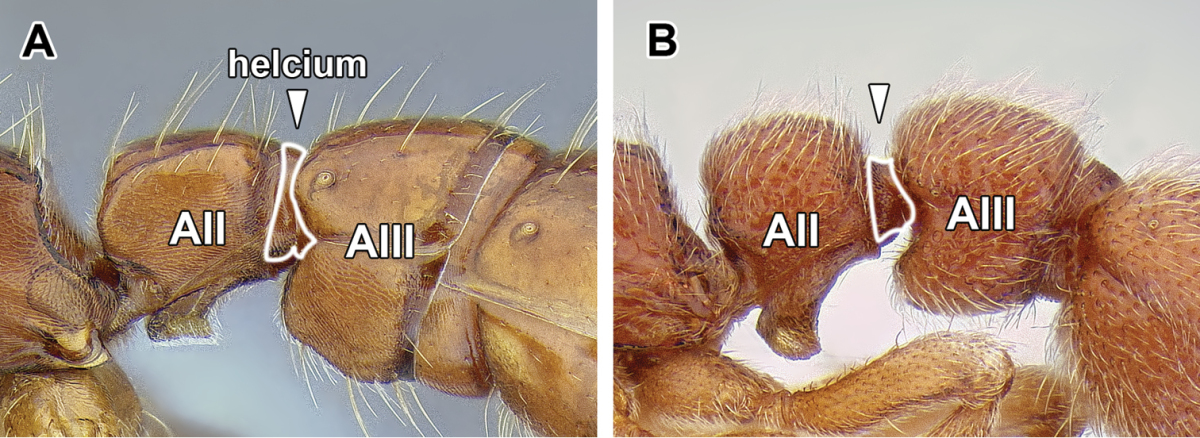	
25 (24)	Pronotal flange not separated from collar by distinct ridge (Figure A). Eyes small, composed of few weakly differentiated ommatidia (Nearctic, Neotropical, Dominican amber)	***Acanthostichus***
–	Pronotal flange separated from collar by distinct ridge (Figure B). Eyes composed of more than 20 well-defined ommatidia (Indomalayan)	***Cerapachys*** (part)
	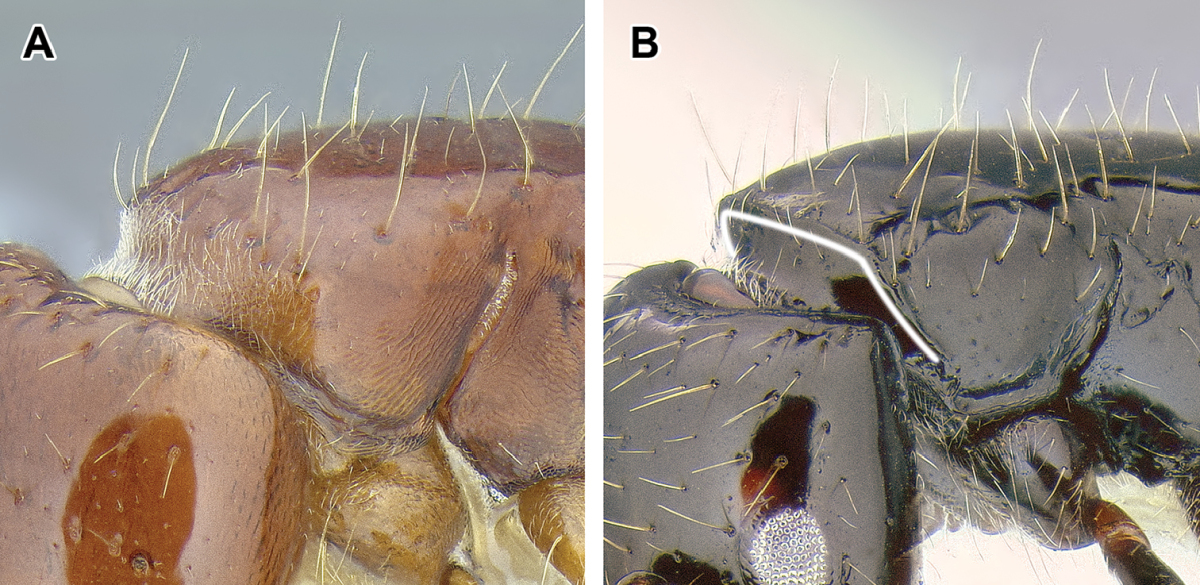	
26 (24)	Constrictions present at anterior end of abdominal segment V and abdominal segment VI (similar to couplet 23, Figure A) (Indomalayan)	***Eusphinctus***
–	Constrictions absent from anterior end of abdominal segment V and abdominal segment VI (similar to couplet 23, Figure B)	**27**
27 (26)	Antennae with 12 segments (Figure A). A pale oval or finger-like patch of cuticle often conspicuous in the middle at posterior margin of abdominal sternite IV (Figure D) (Afrotropical, Malagasy)	***Eburopone***
–	Antennae with 9 to 11 segments (Figures B, C). No visible glandular patch in the middle at posterior margin of abdominal sternite IV (Figure E)	**28**
	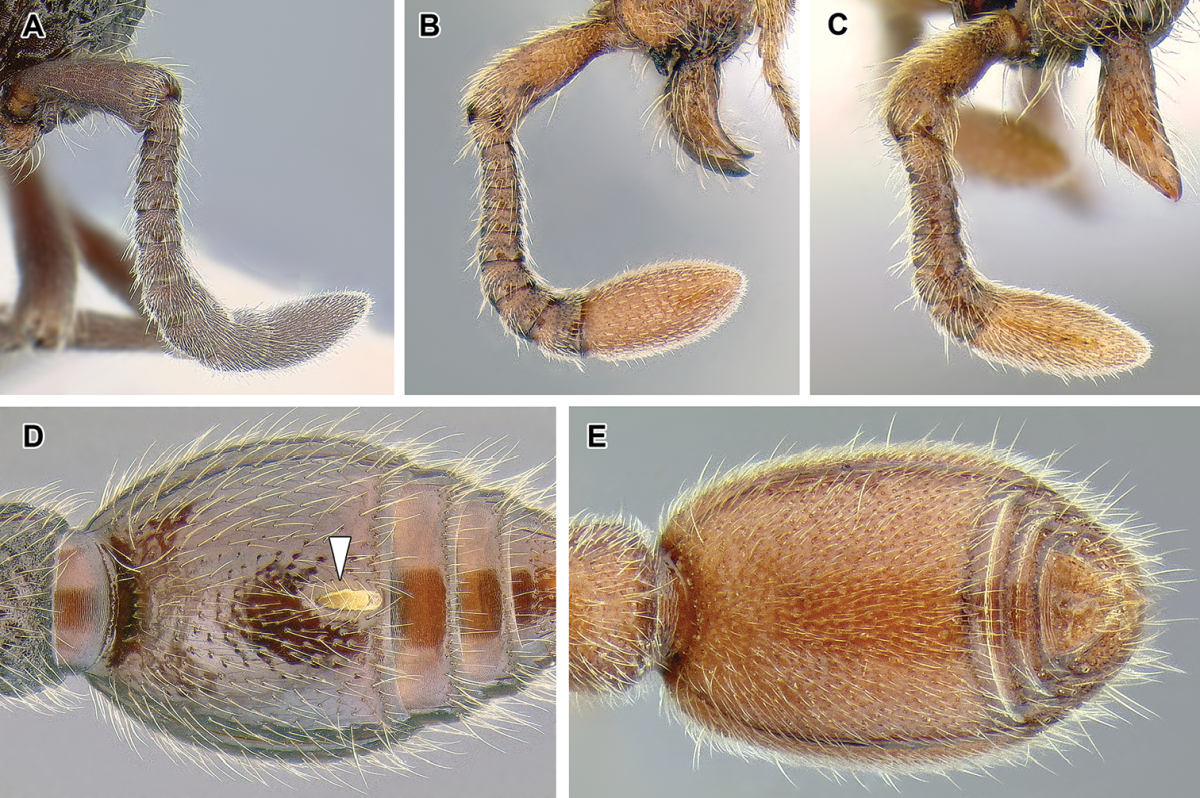	
28 (27)	Abdominal segment III relatively narrow in dorsal view and similar in size to the preceding abdominal segment II segment (petiole). In lateral view, abdominal tergite IV not folding over sternite and the anterior portion of the sternite visible (Figure A). Hind basitarsi not dilating distally, circular in cross-section (Figure C). Metabasitarsal glands absent (Indomalayan, Australasian, *Ooceraea biroi* is a pantropical tramp species)	***Ooceraea***
–	Abdominal segment III relatively wide in dorsal view and larger than the preceding abdominal segment II segment (petiole). In lateral view, abdominal tergite IV folding over sternite and the anterior portion of sternite at least partly obscured (Figure B). Hind basitarsi swollen at about two thirds of their length, oval in cross-section (Figure D). Metabasitarsal glands present in addition to metatibial glands, although difficult to discern under magnification lower than 100× (Nearctic, Neotropical, Palearctic, Indomalayan)	***Syscia***
	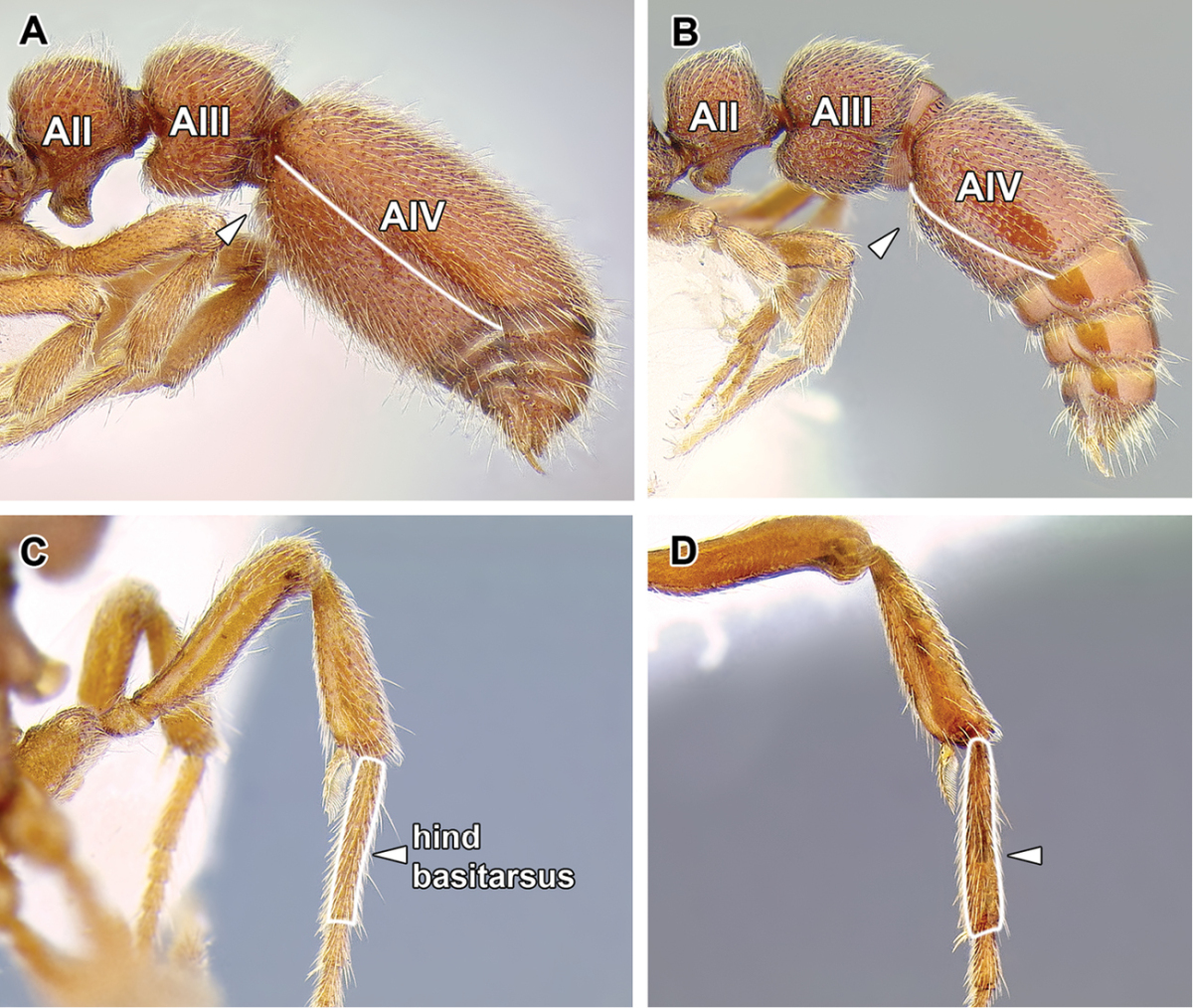	

### Provisional key to the genera of doryline ants based on males

Keys to the true army ants are modified from Gotwald (1982). Males of *Vicinopone* are unknown. This key is preliminary and should be used in conjunction with generic diagnoses and descriptions. Figure pointers refer to plate following couplet.

**Table:** 

1	Tegula inconspicuous or absent, not covering the base of the wing (Figure A). Discal cell (DC) open (Nearctic, Neotropical)	***Leptanilloides***
–	Tegula present, broad or narrow but always covering the base of the wing and easily discernible (Figure B). Discal cell (DC) open or closed	**2**
	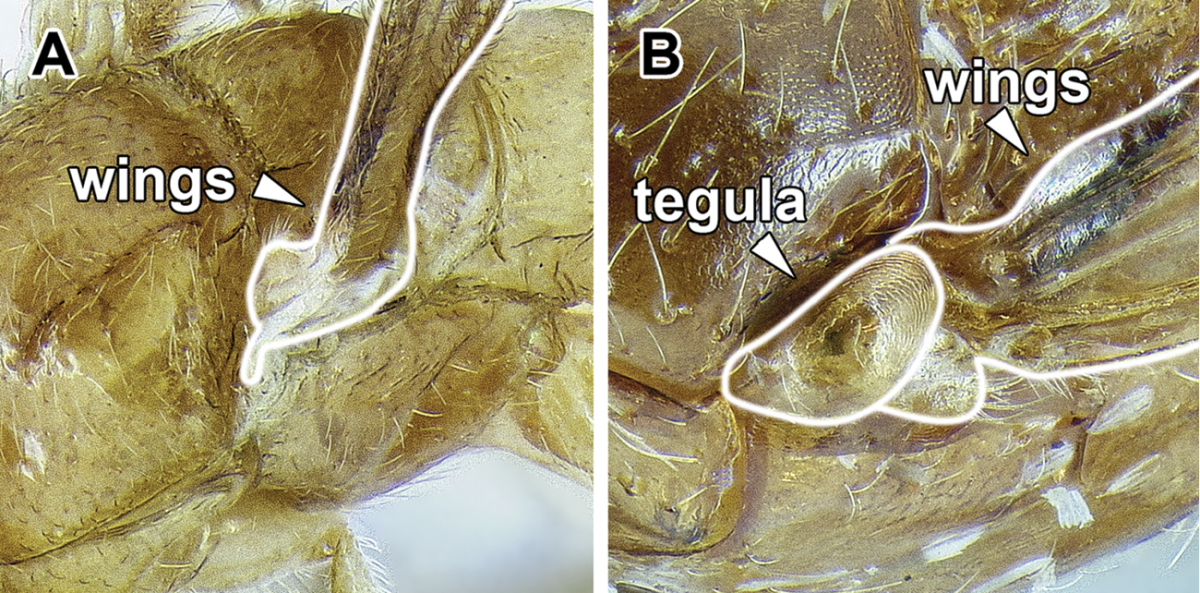	
2 (1)	Propodeal lobes inconspicuous or absent. If present, then not projecting beyond dorsal margin of propodeal foramen (Figure A). Pronotum usually with dorsal and postero-ventral margins meeting at a sharp angle anterior of tegula (Figure C). Head relatively small compared to the mesosoma. Notauli always absent (‘the true army ants’)	**3**
–	Propodeal lobes present, occasionally inconspicuous, projecting beyond dorsal margin of propodeal foramen (Figure B). Pronotum usually with a defined posterior margin in front of tegula, meeting the dorsal margin at approximately right angle (Figure D). Head relatively large compared to the mesosoma. Notauli present or absent (non-army ant dorylines)	**10**
	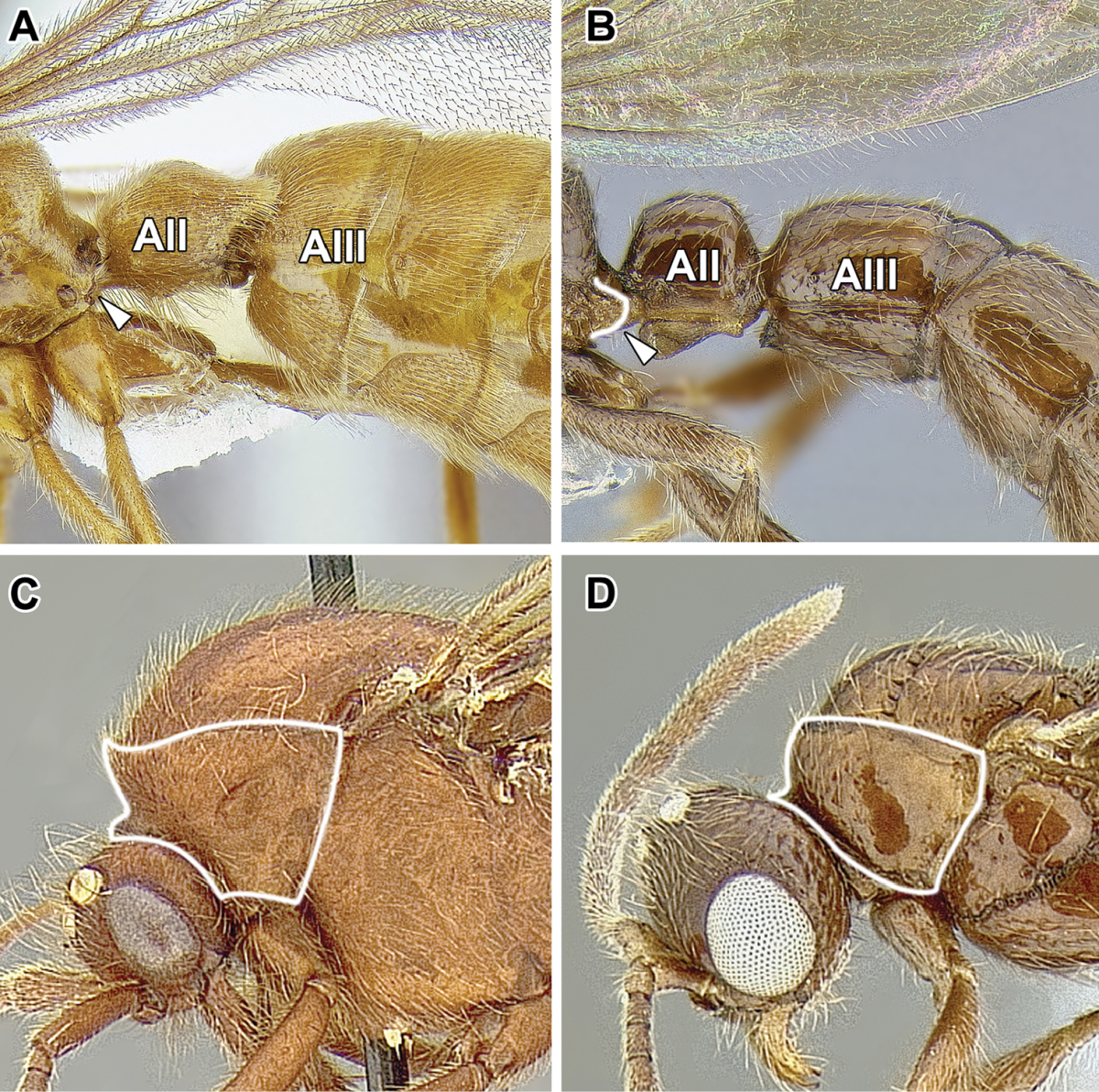	
3 (2)	M·f1 vein of fore wing arising from M+Cu at angle lower than 45° and conspicuously proximal relative to cu-a. Two submarginal cells present (SMC), Rs·f2–3 connecting to M·f1 and marginal cell closed (MC; Figure A)	**4**
–	M·f1 vein of fore wing arises from M+Cu at angle close to or higher than 45° and near cu-a, distal or, less commonly, slightly proximal. Usually one submarginal cell present (SMC; Figures B, C). If Rs·f2–3 dividing the submarginal cell, the marginal cell open (Figure D)	**8**
	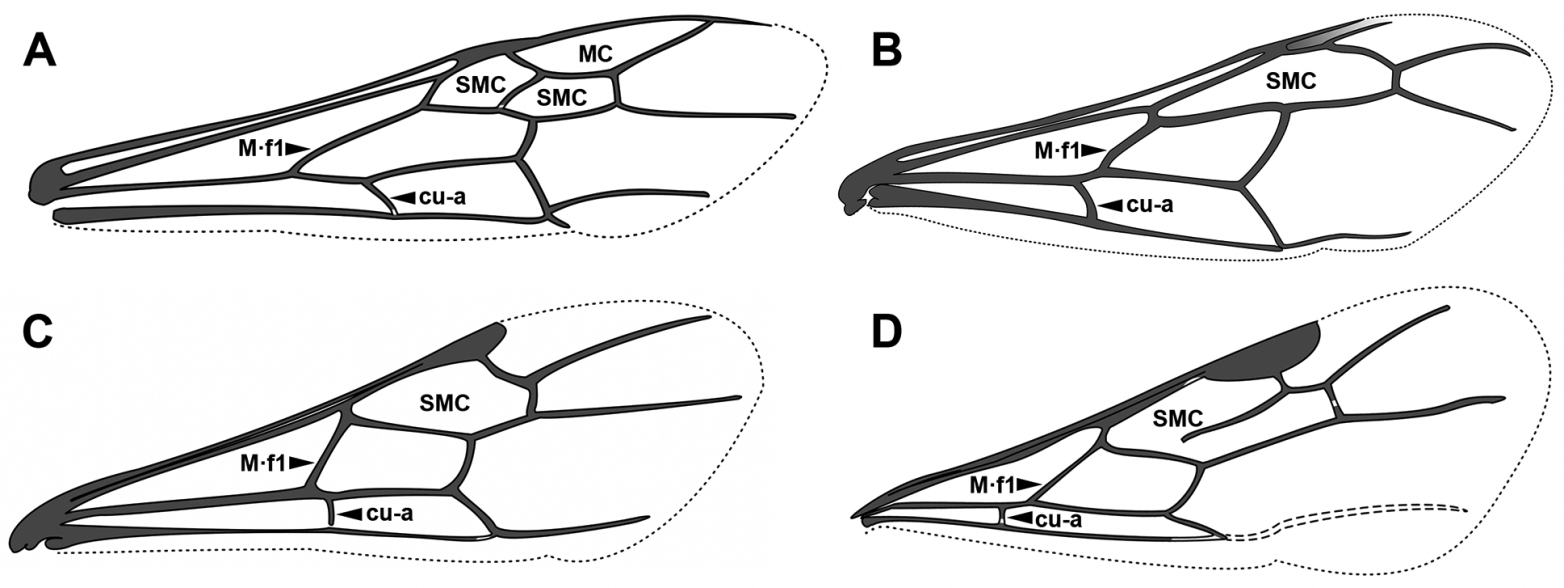	
4 (3)	Abdominal segments III–VII with dense tufts of long setae, distributed throughout the center of tergites; longest setae as long or longer than fore femur (Figure A). Apex of penisvalvae with setae (Nearctic, Neotropical)	***Nomamyrmex***
–	Abdominal segments III–VII without dense tufts of setae. If long setae present, then either confined to posterior half of dorsum of abdominal terga IV–VII or conspicuously shorter than fore femur (Figures B, C). Apex of penisvalvae with or without setae	**5**
	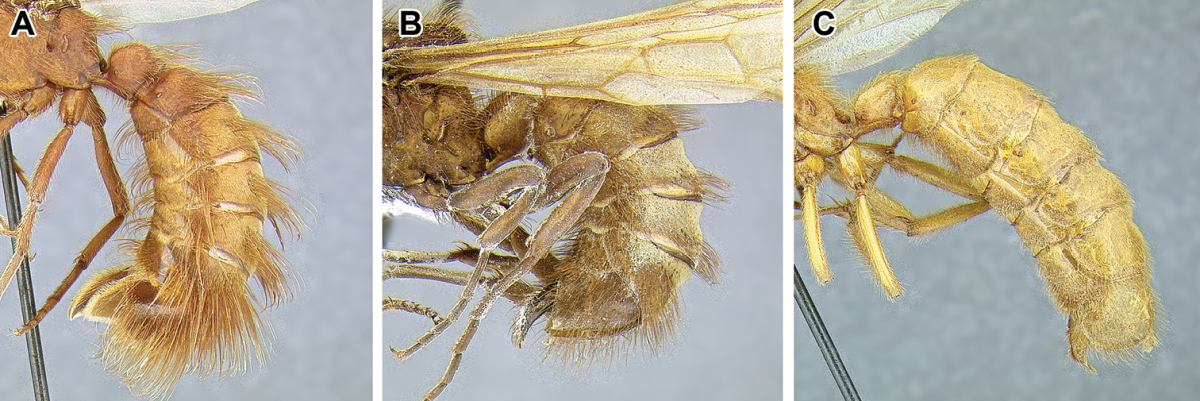	
5 (4)	Apex of penisvalvae without setae (Figure A)	**6**
–	Apex of penisvalvae with setae (Figure B)	**7**
	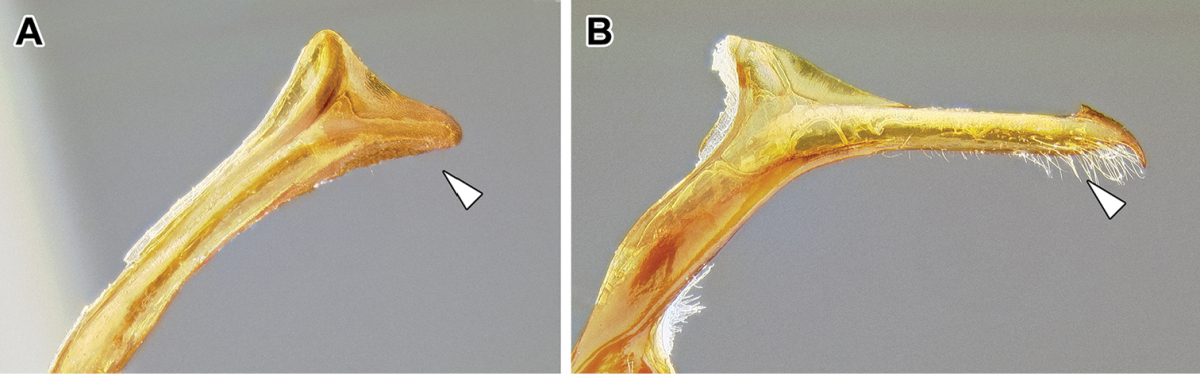	
6 (5)	Abdominal segment II (petiole) dorsum convex, flat or slightly depressed but not deeply excavated (Figure A). Volsella sharply pointed apically, often forked or curving downwards (Figure C). Legs relatively short, in mounted specimens hind femur not reaching past posterior margin of abdominal sternite IV (Nearctic, Neotropical, Dominican amber)	***Neivamyrmex***
–	Abdominal segment II (petiole) dorsum strongly concave (Figure B). Volsella gradually tapering to a blunt apex (Figure D). Legs longer, in mounted specimens hind femur reaching past posterior margin of abdominal sternite IV (Neotropical)	***Eciton***
	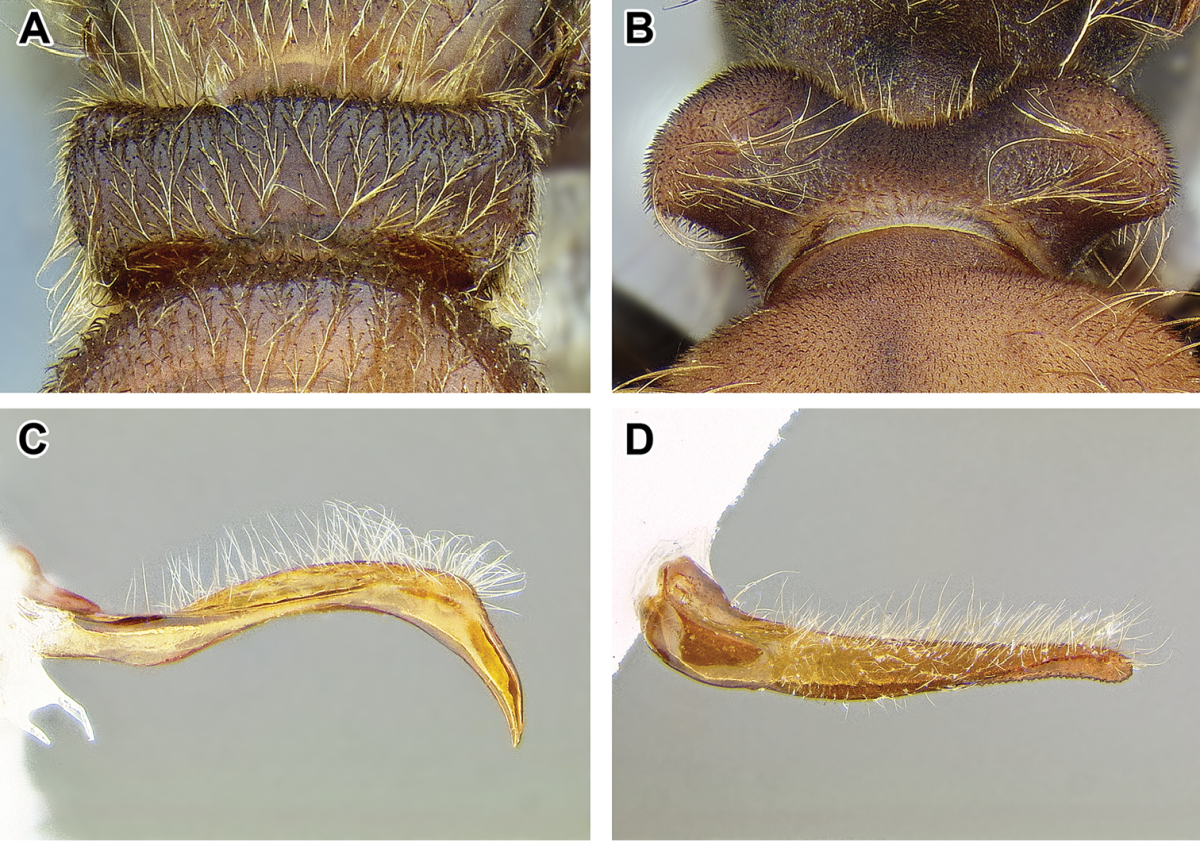	
7 (5)	Abdominal sternite IX (subgenital plate) with four teeth (Figure A). Basal tarsal segment of hind leg flattened, without grooves (Figure C) (Neotropical)	***Cheliomyrmex***
–	Abdominal sternite IX with two teeth (Figure B). Basal tarsal segment of hind leg complex, with oblique groove accommodating tibial spur (Figure D) (Nearctic, Neotropical)	***Labidus***
	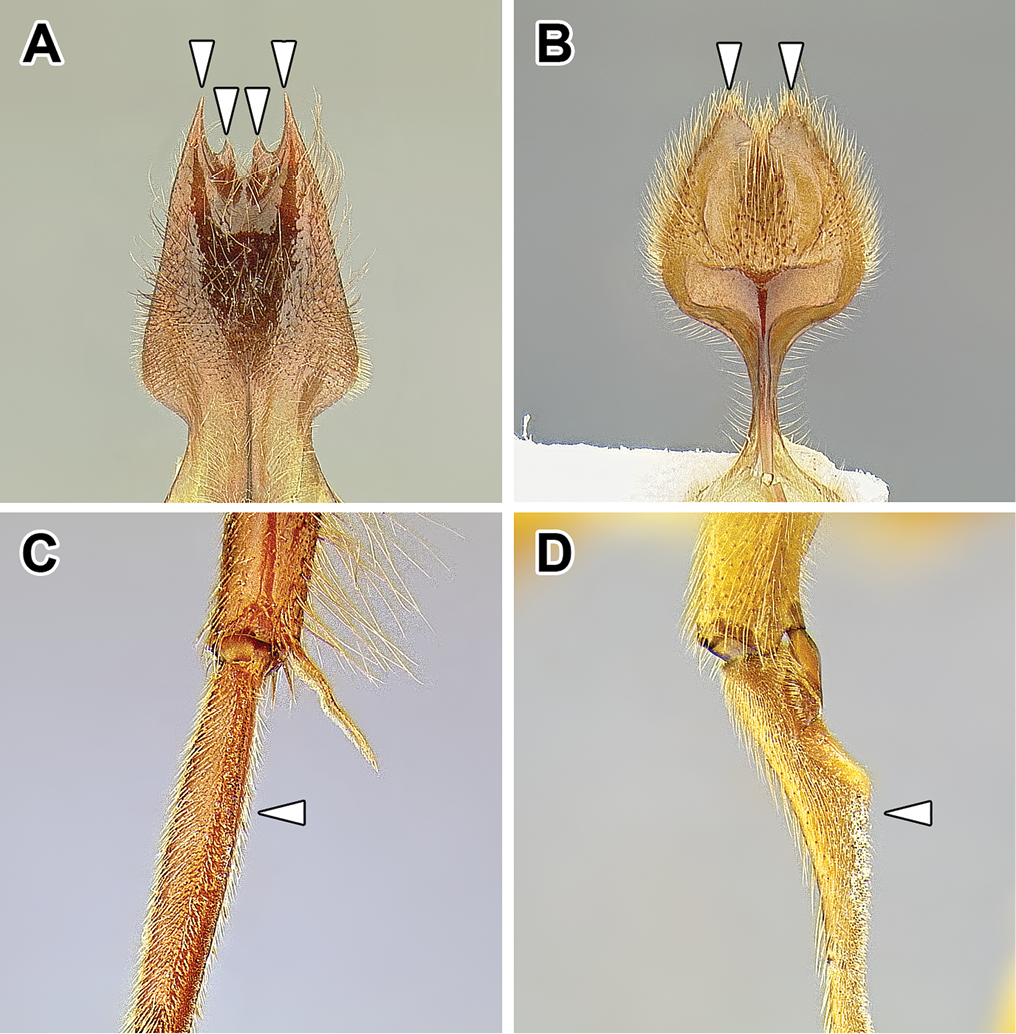	
8 (3)	Submarginal cell (SMC) in fore wing partly or entirely divided by Rs·f2–3 vein (Figure A). In full face view head capsule excluding eyes and mandibles longer than wide (Figure C) (Afrotropical)	***Aenictogiton***
–	Submarginal cell (SMC) in fore wing not divided (Figure B). In full face view head capsule excluding eyes and mandibles wider than long (Figure D)	**9**
	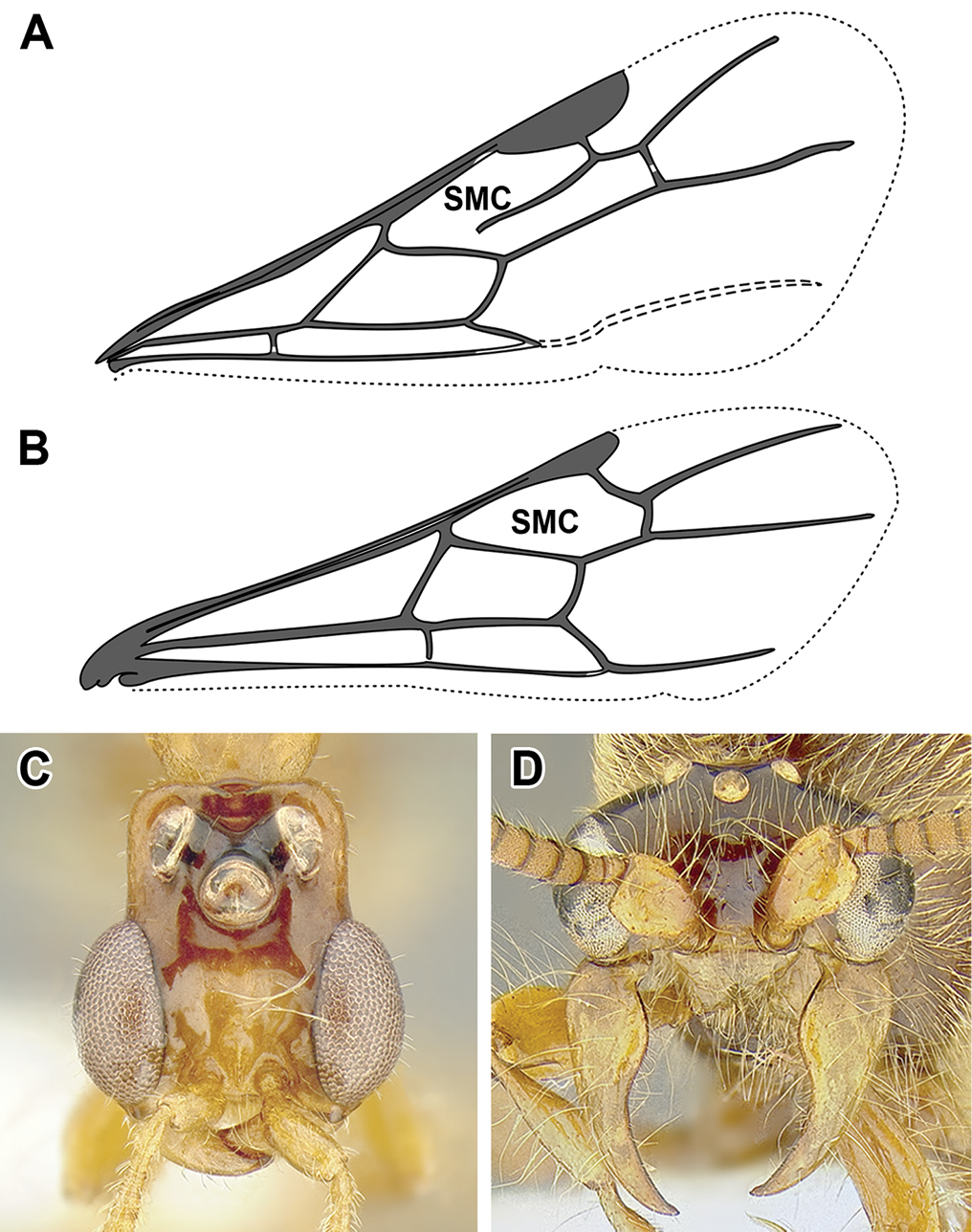	
9 (8)	Pterostigma narrow or inconspicuous and anterior wing margin pigmented (Figure A) Trochanters and femora compressed, broad relative to cylindrical tibiae (Figure C) (Palearctic, Afrotropical, Indomalayan)	***Dorylus***
–	Pterostigma broad, often with convex posterior edge, wing margin not pigmented past pterostigma (Figure B). Trochanters and femora never compressed. If femora flattened and broad, trochanter cylindrical and tibia not conspicuously more narrow than femora (Figure D) (Afrotropical, Palearctic, Indomalayan, Australasian)	***Aenictus***
	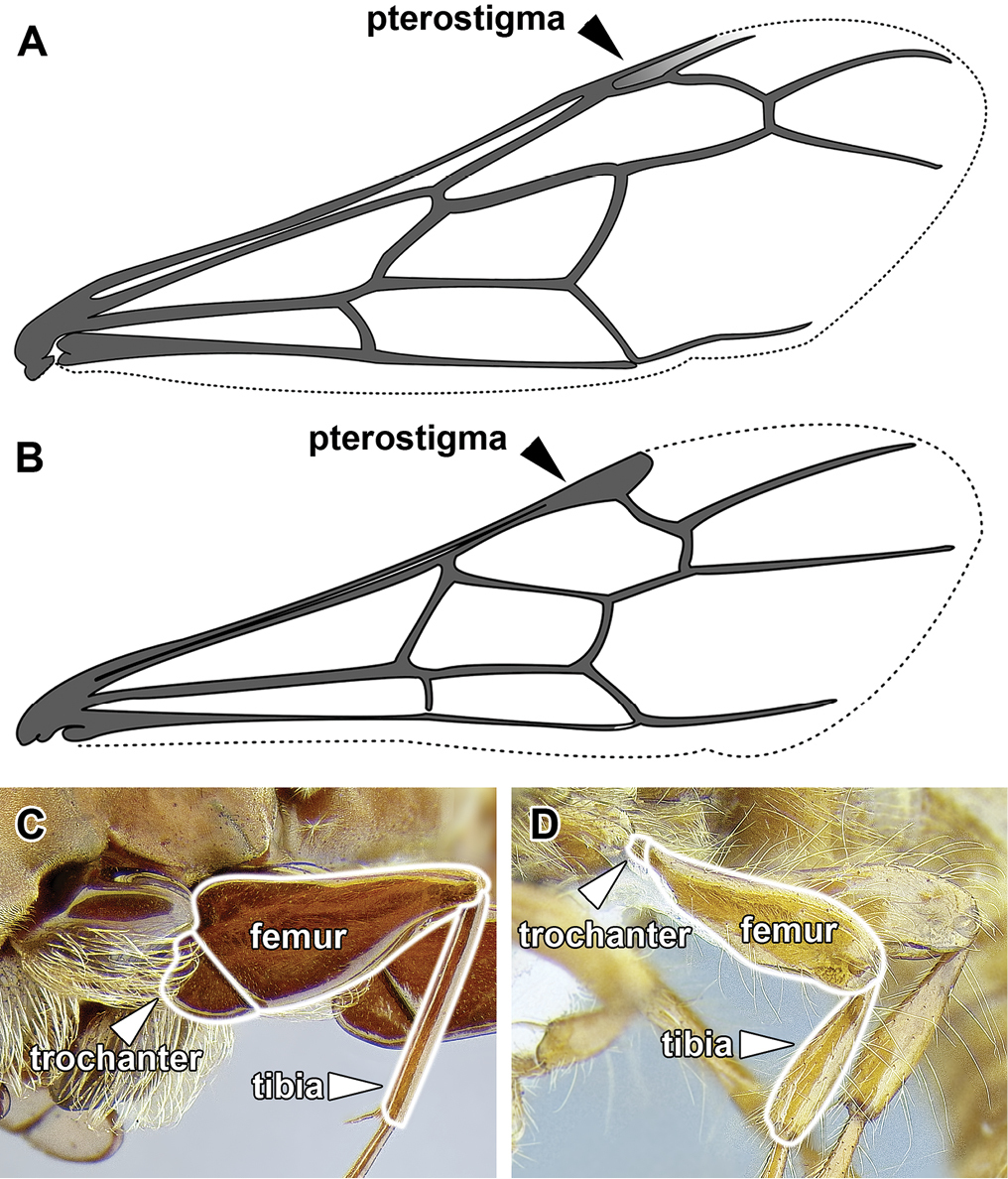	
10 (2)	Maxillary palps very long and reaching occipital foramen, 6-segmented and visible in mounted specimens (Figure A) (Malagasy)	***Tanipone***
–	Maxillary palps never reaching occipital foramen, usually not visible without dissection and often with fewer than six segments (Figure B)	**11**
	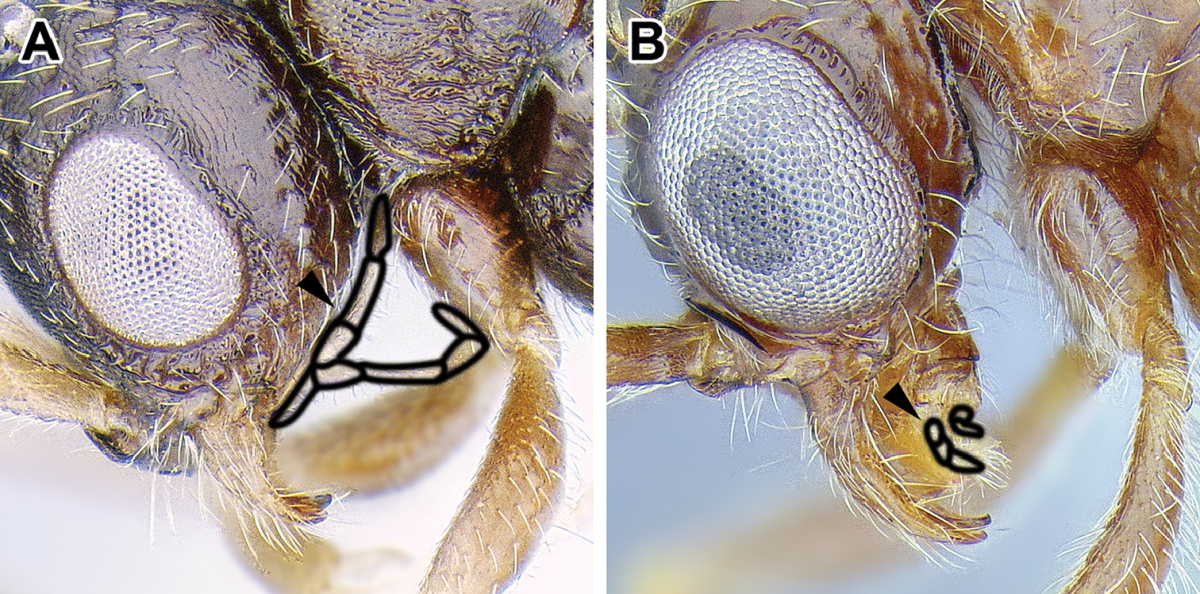	
11 (10)	Constriction present between pre- and postsclerites of abdominal segment V, both dorsally and ventrally **and** helcium circumference small with helcium positioned at about the midheight of segment III (Figures A, B)	**12**
–	No constriction between pre- and postsclerites of abdominal segment V (Figure C) **or** helcium circumference large and helcium positioned above midheight of segment III. Rarely, pre- and posttergites may be separated by a gutter-like cinctus but in lateral view there is no constriction, the surface of pre- and postsclerites is contiguous and there is no cinctus on the sternite	**13**
	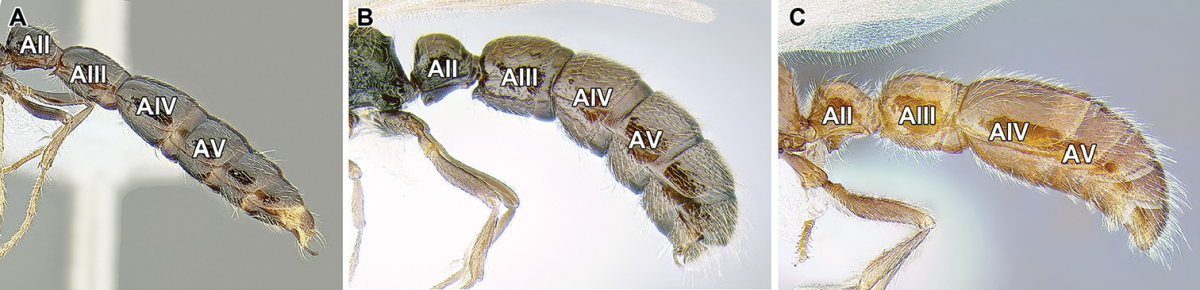	
12 (11)	Antennae with 12 segments (Indomalayan)	***Eusphinctus***
–	Antennae with 13 segments (Neotropical)	***Sphinctomyrmex***
13 (11)	Veins C and R·f3 absent from the fore wing (Figure A). Sternite of abdominal segment IX (subgenital plate) usually visible without dissection as two thin spines, in lateral view curved upwards. Posttergite of abdominal segment VIII (pygidium) often flat or impressed and delimited by a carina (Afrotropical, Indomalayan, Australasian)	***Zasphinctus***
–	Veins C and R·f3 present in the fore wing (Figure B). Sternite of abdominal segment IX (subgenital plate) visible without dissection as two thin spines, in lateral view more or less straight or slightly upcurved. Posttergite of abdominal segment VIII (pygidium) not delimited by a carina	**14**
	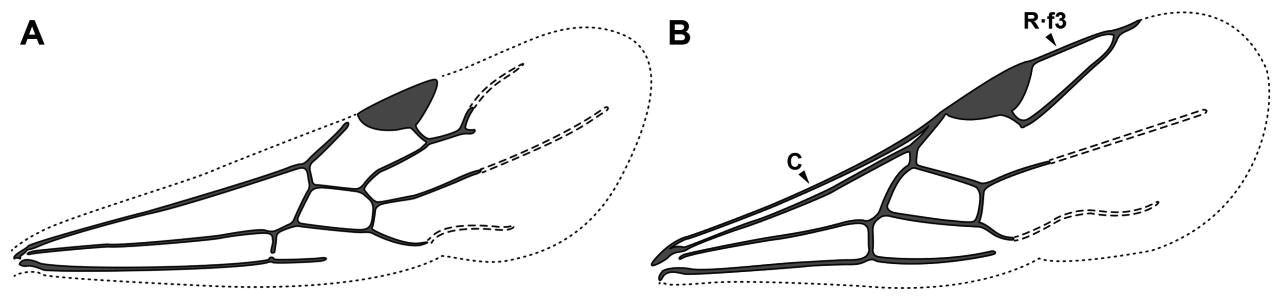	
14 (13)	Submarginal cell (SMC) in fore wing closed by Rs·f2–3 (a fenestra may be present at junction of Rs+M and Rs·f2–3) (Figure A) **or** SMC open but Rs·f2–3 present and 2rs-m also developed, closing SMC or not (Figure B)	**15**
–	SMC not closed by Rs·f2–3, either open (Figure C) **or** Rs·f2–3 completely absent and SMC closed by vein 2rs-m (Figure D)	**23**
	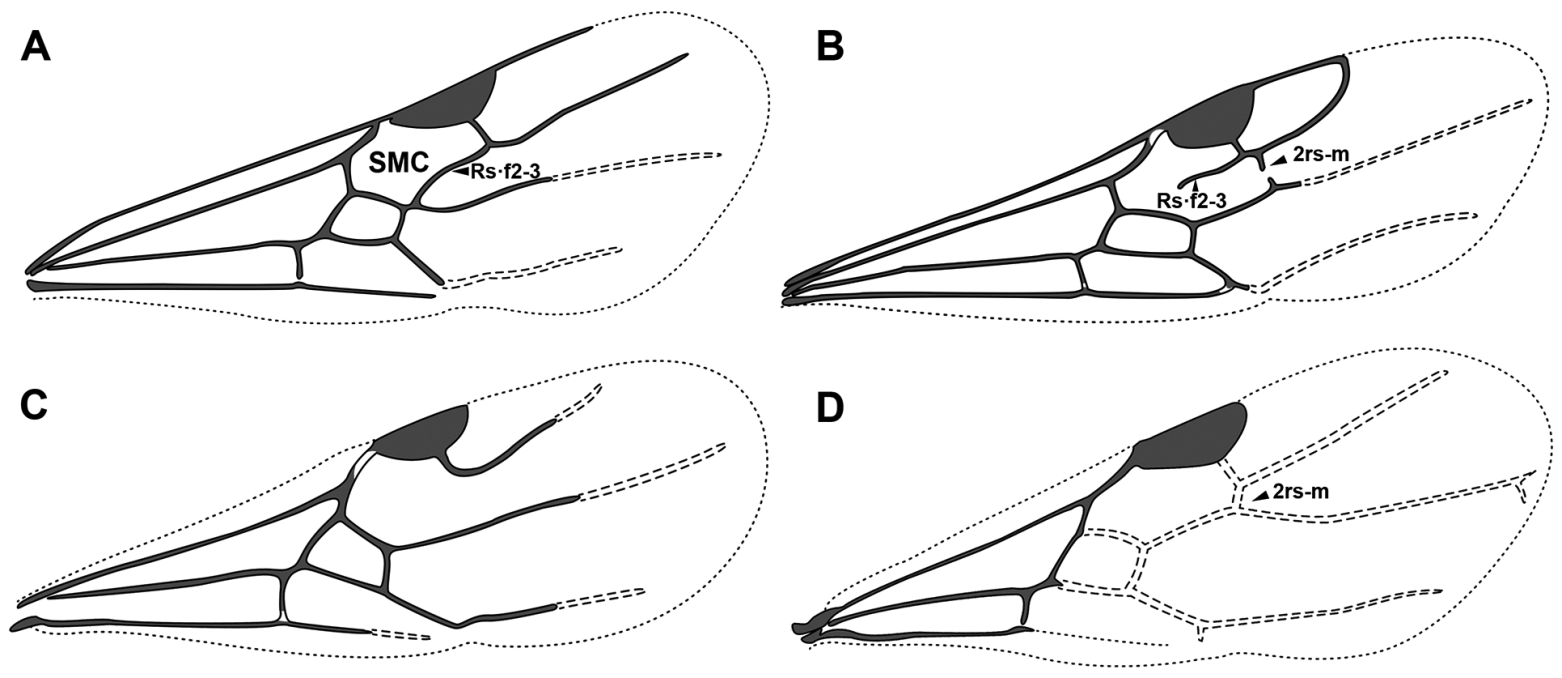	
15 (14)	Vein 2rs-m present, partial or complete in fore wing (Figure A)	**16**
–	Vein 2rs-m absent or at most stub-like in fore wing (Figure B)	**19**
	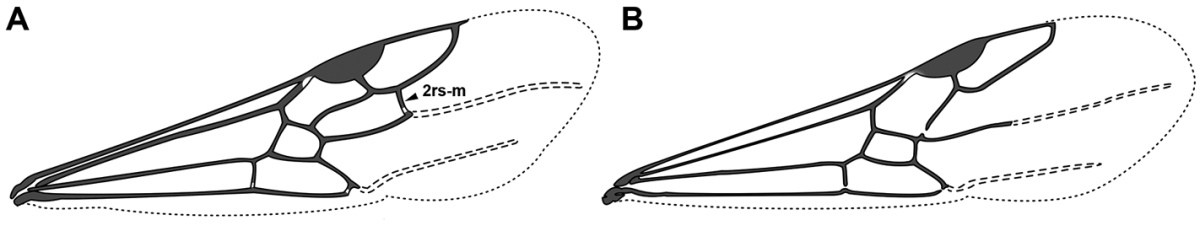	
16 (15)	Hind tibiae with one spur (Figure A)	**17**
–	Hind tibiae with two spurs (Figure B)	**18**
	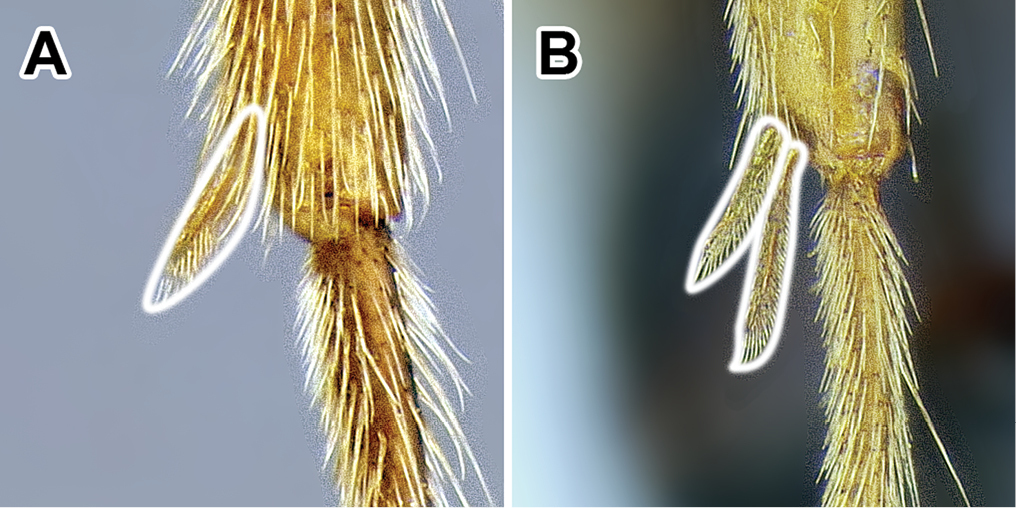	
17 (16)	Marginal cell closed (Baltic amber)	***Procerapachys***
–	Marginal cell open (Nearctic, Neotropical, Dominican amber)	***Acanthostichus*** (part)
18 (16)	Mesopleuron divided by oblique groove, irregularly sculptured (Figure A) (Malagasy, Indomalayan, Baltic amber)	***Chrysapace***
–	Mesopleuron not divided by a groove, mostly smooth with longitudinal rugae (Figure B) (Neotropical, Dominican amber)	***Cylindromyrmex***
	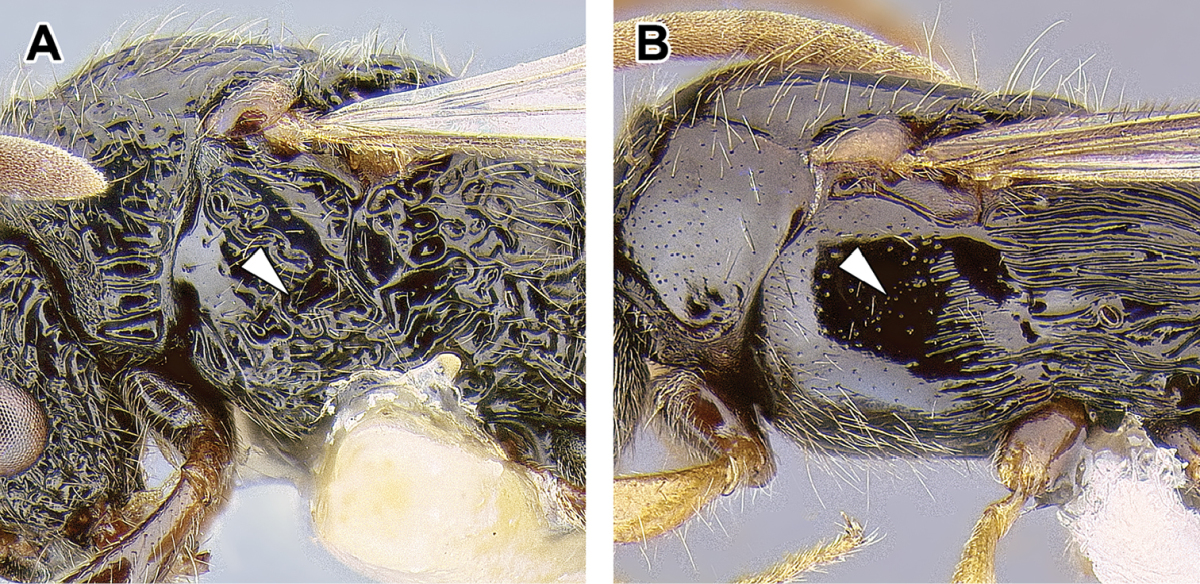	
19 (15)	Costal vein (C) absent in fore wing, R·f3 absent or at most a stub past pterostigma (Figure A)	**20**
–	Costal vein (C) present in fore wing, R·f3 present past pterostigma (Figure B)	**21**
	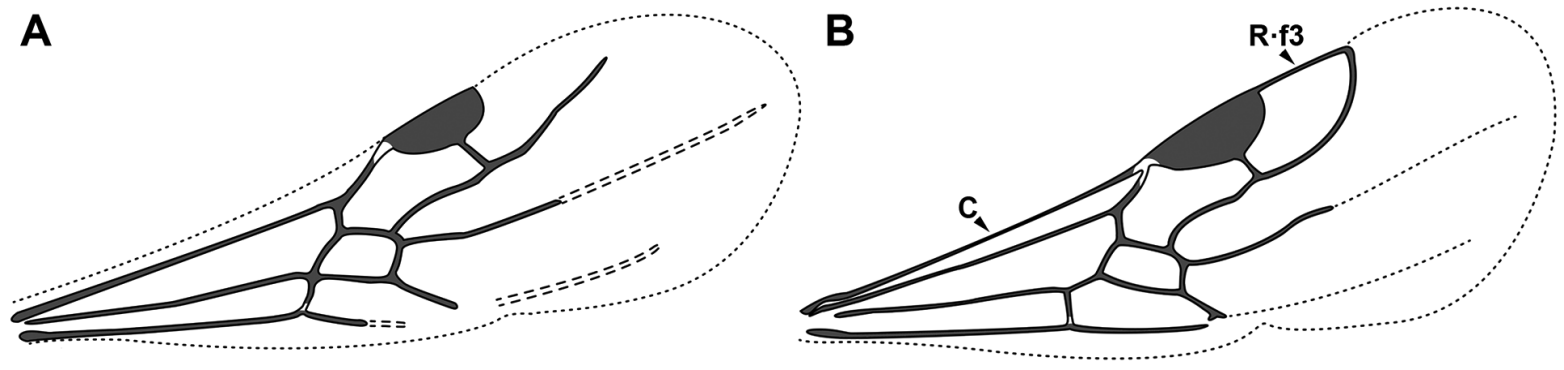	
20 (19)	Helcium circumference large and helcium positioned supraaxially; posterior face of abdominal tergite II (petiolar node) and anterior face of abdominal tergite III poorly developed (Figure A) (Malagasy)	***Lividopone*** (part)
–	Helcium circumference small and helcium positioned axially; posterior face of abdominal tergite II (petiolar node) and anterior face of abdominal tergite III well developed (Figure B) (Palearctic, Afrotropical, Malagasy, Indomalayan, Australasian)	***Parasyscia*** (part)
	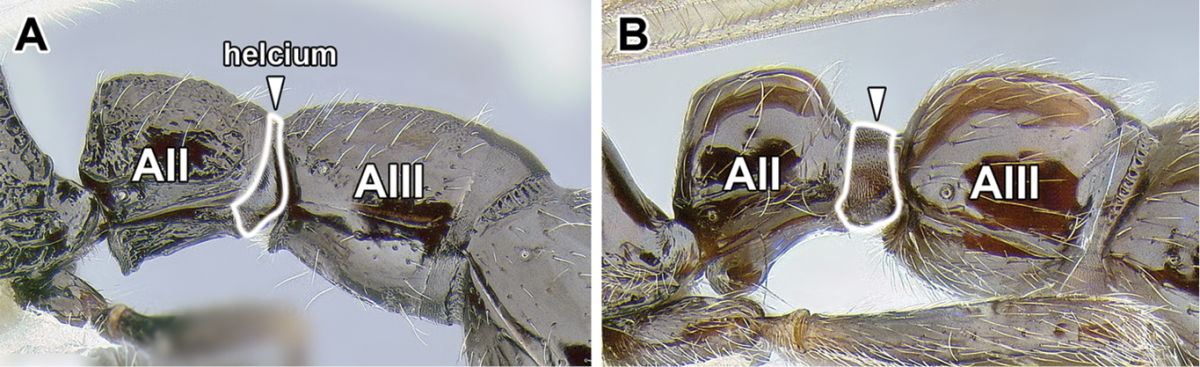	
21 (19)	Abdominal segment III very broadly attached to segment IV such that waist appears composed of one segment. Gaster usually widest posterior to abdominal segment IV (Figure A) (Indomalayan)	***Yunodorylus*** (part)
–	Abdominal segment III narrowly attached to segment IV such that second segment of the waist (postpetiole) somewhat differentiated from rest of gaster. Gaster widest at abdominal segment IV (Figure B)	**22**
	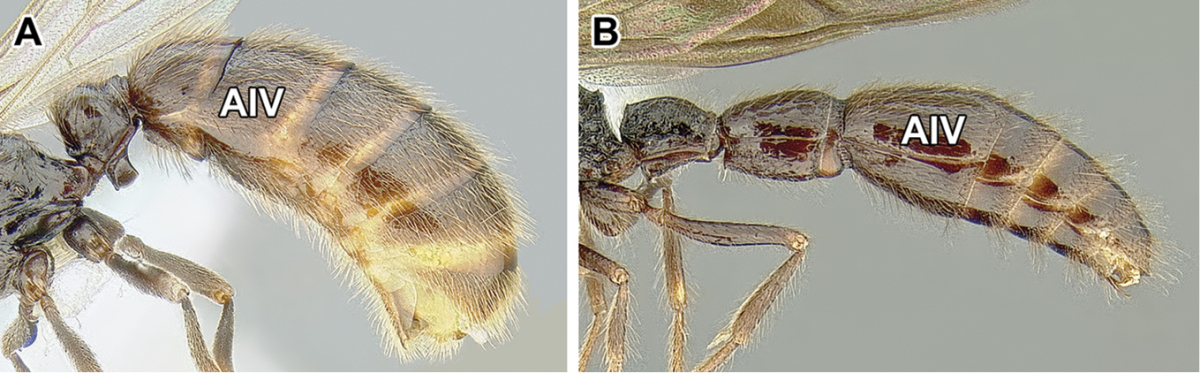	
22 (21)	Antennal segment III the shortest segment (Figure A). In lateral view, anterior margin of eye situated very close to mandibular insertion, separated by less than maximum scape diameter. Maxillary palps with 4 segments, labial palps with 3 segments (Neotropical)	***Neocerapachys*** (part)
–	Antennal segment II is the shortest segment (Figure B). In lateral view, anterior margin of eye is situated relatively far from mandibular insertion, separated by more than maximum scape diameter. Maxillary palp with 5 segments, labial palps with 3 segments (Indomalayan)	***Cerapachys***
	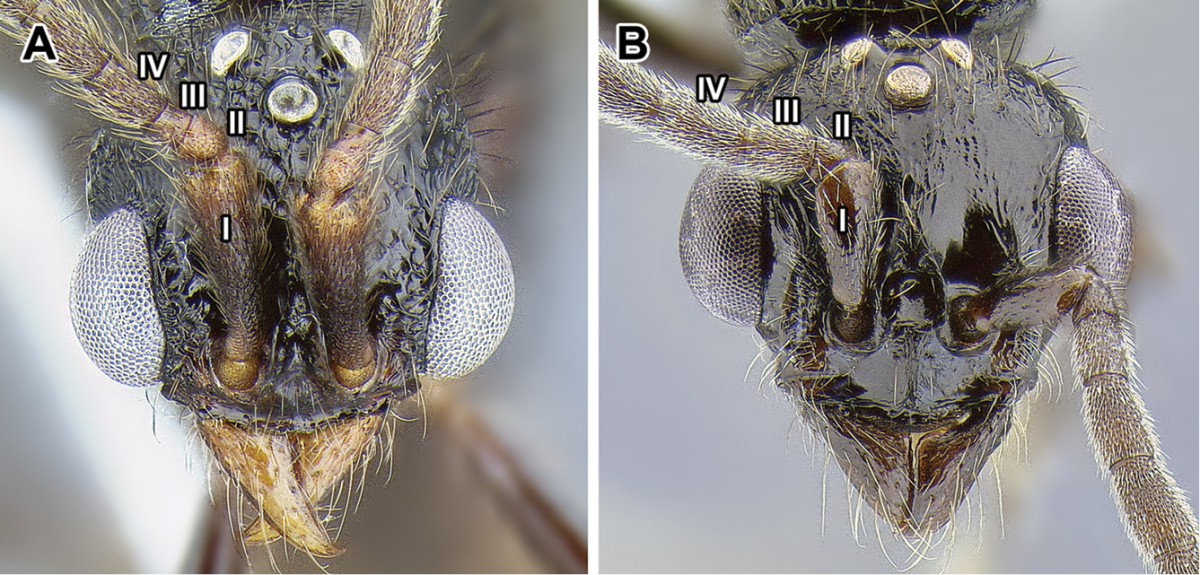	
23 (14)	Notauli absent (Figure A)	**24**
–	Notauli present, at least anteriorly (Figure B)	**27**
	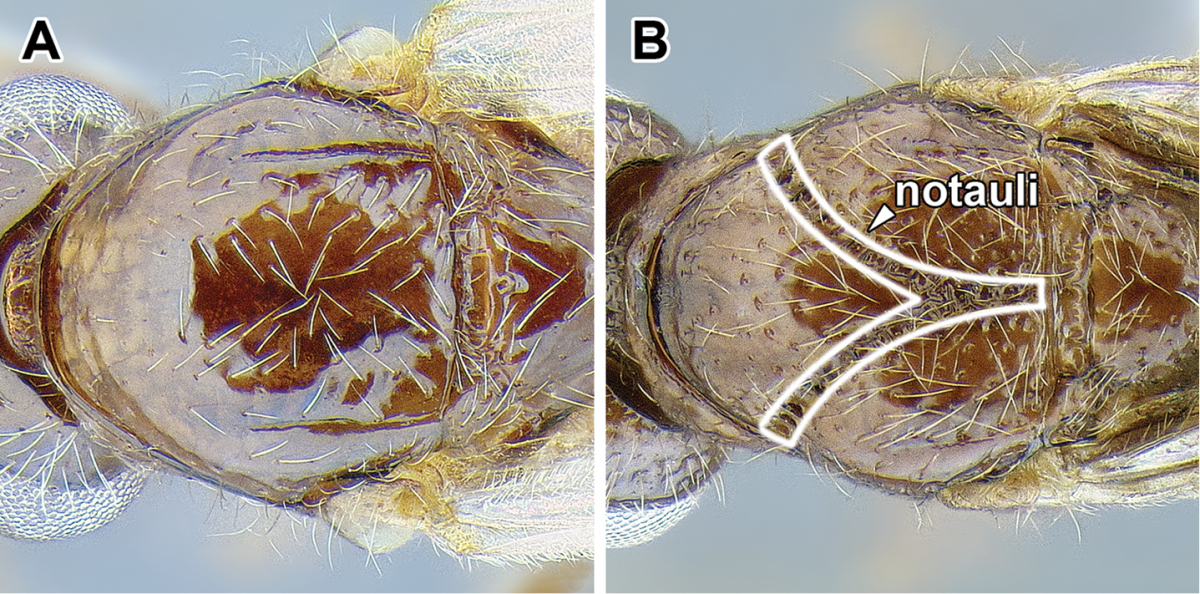	
24 (23)	Helcium circumference large and helcium positioned supraaxially; posterior face of abdominal tergite III and anterior face of abdominal tergite IV poorly developed (Figure A). Maxillary palps 2-segmented, labial palps 3-segmented (Nearctic, Neotropical, Dominican amber)	***Acanthostichus*** (part)
–	Helcium circumference small and helcium positioned axially; posterior face of abdominal tergite III and anterior face of abdominal tergite IV developed (Figure B). Maxillary palps not 2-segmented in combination with 3-segmented labial palps	**25**
	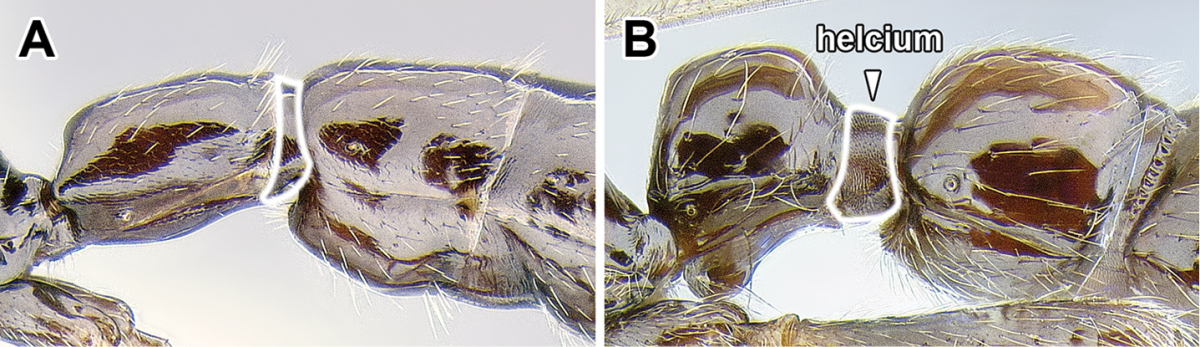	
25 (24)	R·f3 vein present in fore wing (Figure A), long and conspicuous, sometimes joining Rs·f4–5 to form a closed marginal vein (Indomalayan)	***Yunodorylus*** (part)
–	R·f3 vein absent in fore wing, at most a stub (Figure B)	**26**
	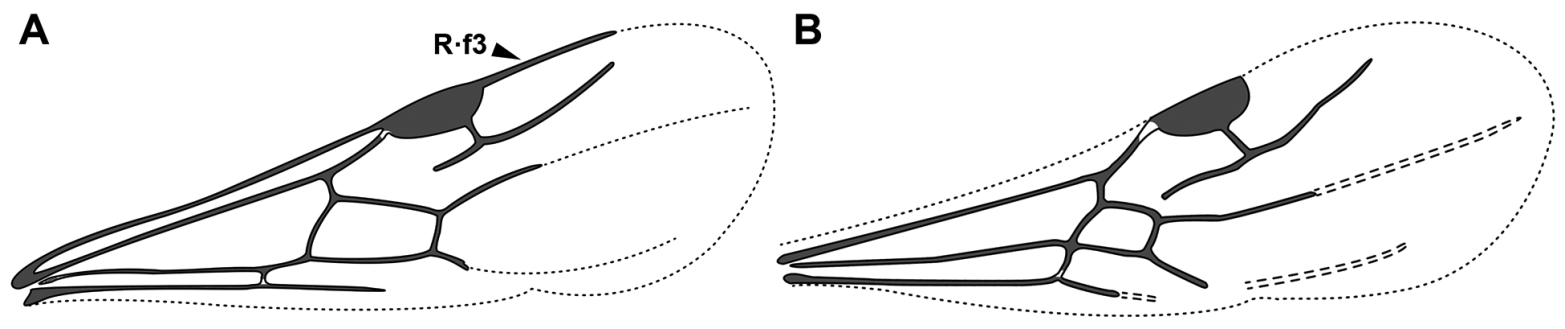	
26 (25)	Rs·f2–3 vein absent in fore wing. Pterostigma gives origin to a ‘free stigmal vein’ composed of 2r-rs&Rs·f4–5 (Figure A) or Rs connected to M through 2rs-m or, in smaller species, the free stigmal vein entirely absent or only a stub of 2r-rs present (Palearctic, Afrotropical, Malagasy, Indomalayan, Australasian)	***Lioponera*** (part)
–	Rs·f2–3 vein present in fore wing, long or a stub (Figure B) (Palearctic, Afrotropical, Malagasy, Indomalayan, Australasian)	***Parasyscia*** (part)
	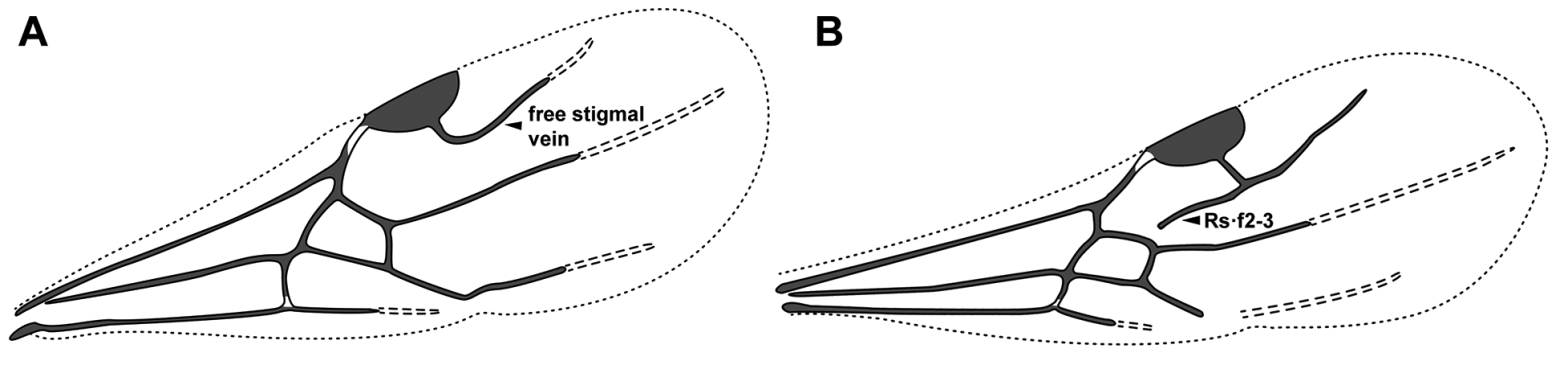	
27 (23)	Inner margins of antennal sockets concealed by ‘frontal carinae’ (torulo-posttorular complex) in full-face view (Figure A). Middle tibiae without spurs (Figure C) (Afrotropical, Malagasy, Indomalayan, Australasian)	***Simopone***
–	Antennal sockets completely exposed in full-face view (Figure B). Middle tibiae with a single spur, which may be simple and inconspicuous (Figure D)	**28**
	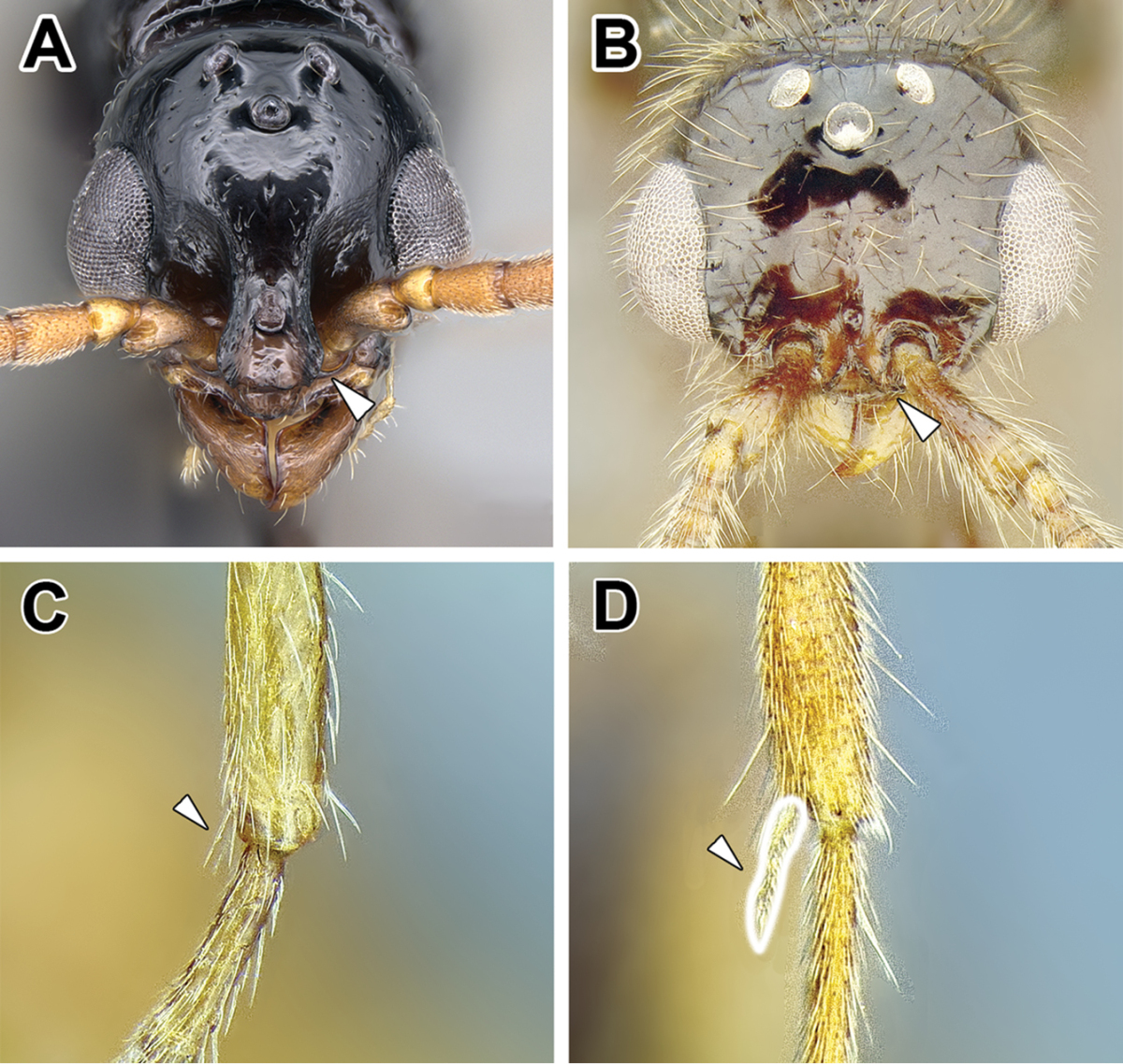	
28 (27)	Helcium circumference large and helcium positioned supraaxially; posterior face of abdominal tergite II (petiolar node) and anterior face of abdominal tergite III poorly developed (similar to couplet 12 Figure A) (Malagasy)	***Lividopone*** (part)
–	Helcium circumference small and helcium positioned axially; posterior face of abdominal tergite II (petiolar node) and anterior face of abdominal tergite III well developed (similar to couplet 12 Figure B)	**29**
29 (28)	Antennae with 11 or 12 segments	**30**
–	Antennae with 13 segments	**31**
30 (29)	Discal cell (DC) often closed (Figure A). Abdominal segment III distinctly smaller than the succeeding segment IV, i.e. postpetiole well differentiated and often similar in size to abdominal segment II (petiole). Abdominal sternite VII almost always modified, notched, equipped with tufts of setae, with palpiform or flat projections, or otherwise hypertrophied (Figure C). Most species with 11-segmented antennae, some with 12-segmented (Indomalayan, Australasian, *Ooceraea biroi* is a pantropical tramp species)	***Ooceraea***
–	Discal cell (DC) open (Figure B). Abdominal segment III may be smaller than the succeeding segment IV but usually larger than abdominal segment II (petiole). Abdominal sternite VII simple, never modified (Figure D). Antennae 12-segmented (Palearctic, Nearctic, Neotropical, Indomalayan)	***Syscia***
	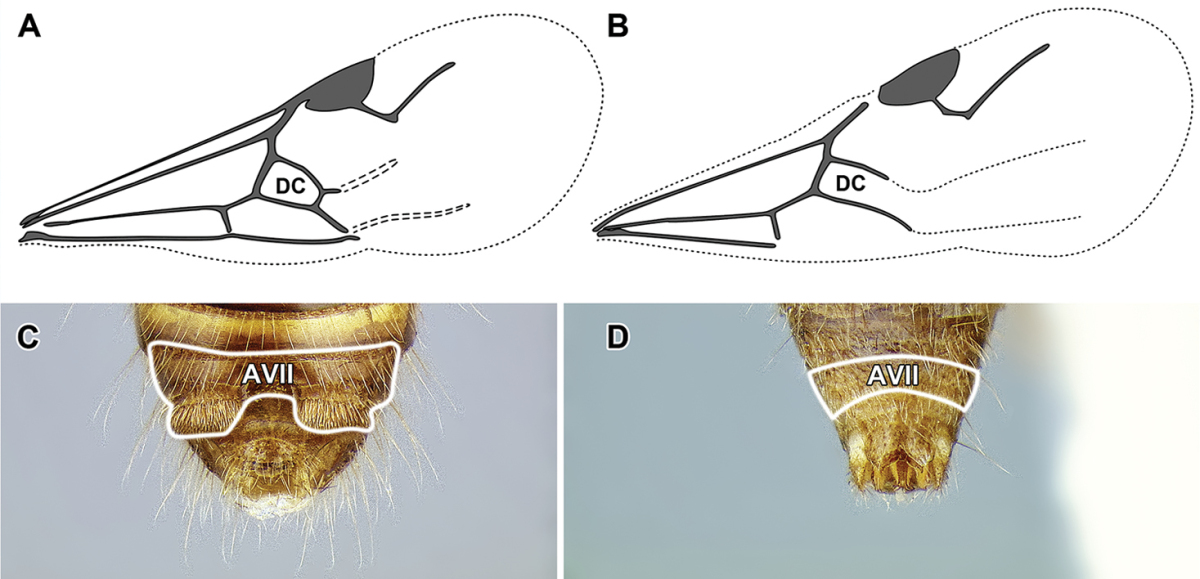	
31 (29)	Costal (C) and R·f3 veins absent from fore wing (Figure A) (Palearctic, Afrotropical, Malagasy, Indomalayan, Australasian)	***Lioponera*** (part)
–	Costal (C) vein always present in fore wing, R·f3 often present (Figure B)	**32**
	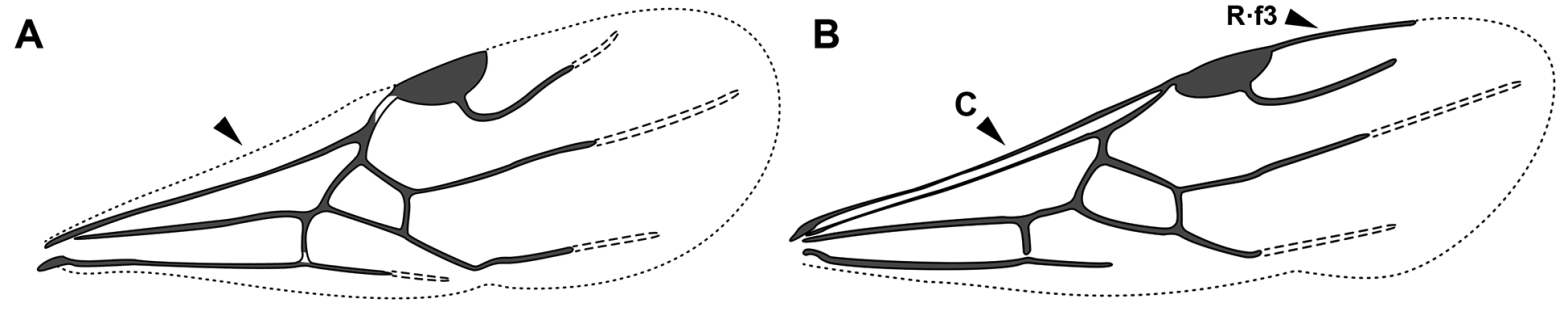	
32 (31)	Rs·f2–3 vein present in fore wing (Figure A). Propodeal declivity and anterior face of abdominal segment II (petiole) surrounded by a conspicuous carina (Neotropical)	***Neocerapachys*** (part)
–	R·f2–3 vein absent in fore wing (Figure B). Propodeal declivity and anterior face of abdominal segment II usually not surrounded by a carina (Afrotropical, Malagasy)	***Eburopone***
	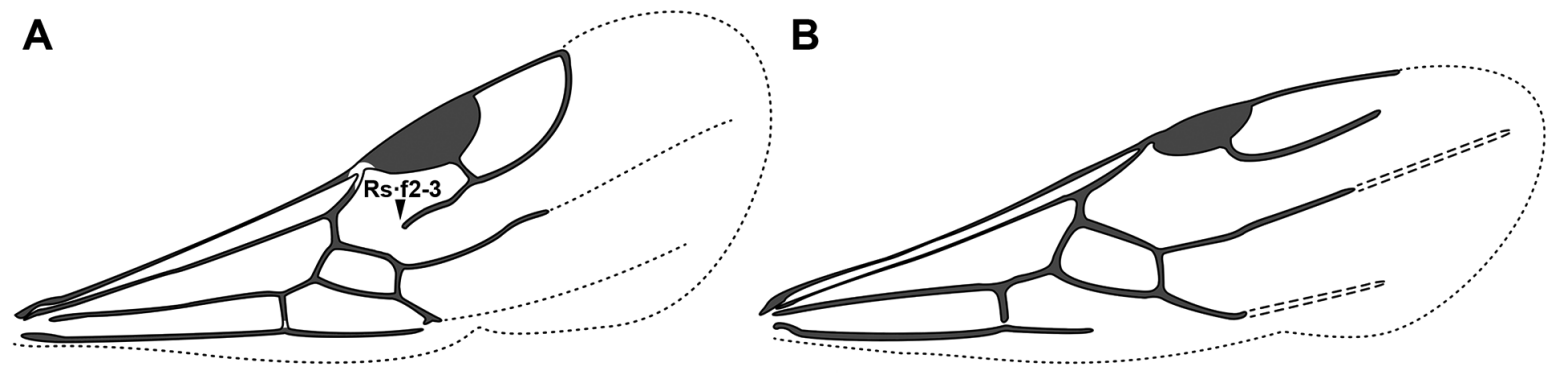	

## Taxonomic treatment of the genera of Dorylinae

### Acanthostichus

Taxon classificationAnimaliaHymenopteraFormicidae

Mayr, 1887

= Ctenopyga Ashmead, 1906

#### Type-species.

*Typhlopone serratula*, by monotypy.

*Acanthostichus* is a New World genus of termite-hunting dorylines most closely related to *Cylindromyrmex*.

#### Diagnosis.

***Worker.*** The workers of this distinctive lineage can be recognized by a combination of 12-segmented antennae, absence of ridge on pronotal collar, unfused pronotomesopleural Pronotomesopleural suture, highly positioned helcium, a single pectinate spur on mid and hind tibiae, propodeal spiracle usually positioned below the midheight of the sclerite, and large pygidium armed with modified finger-like setae. Restricted to the New World, the species of *Acanthostichus* are medium-sized ants that are often brown or yellowish in coloration and lack distinctive sulcate or striate sculpturing characteristic of its close relative *Cylindromyrmex* or very conspicuous constrictions between gastral segments of *Sphinctomyrmex*. Other New World dorylines (army ants related to *Eciton*, species of *Leptanilloides*) all have simple, small pygidium, at most armed with several thick setae. Workers of *Syscia* and the introduced *Ooceraea biroi*, also found in the New World, can be distinguished by antennal segment count reduced to 11 or 9, respectively.

***Male.*** The male of *Acanthostichus* can be separated from all other dorylines by a combination of propodeal lobes conspicuous, supraaxial helcium, single spur on each mid and hind tibiae, costal vein (C) present in fore wing, and R·f3 present past pterostigma but not enclosing a cell with Rs·f5. Most species appear to have 12-segmented antennae but at least *Acanthostichus texanus* and *Acanthostichus davisi* are known to have 13 antennal segments. Among New World dorylines the habitus of males is similar to that of *Cylindromyrmex*, *Neocerapachys*, *Syscia*, and *Sphinctomyrmex*. *Sphinctomyrmex* has conspicuous constrictions between abdominal segments IV, V, and VI in combination with narrow axial helcium, *Cylindromyrmex* has two tibial spurs, and *Syscia* lacks the costal vein in the fore wing. *Neocerapachys* has either a closed marginal cell or lacks cross-vein 2rs-m. Furthermore, *Acanthostichus* males that lack 2rs-m have a broader helcium and more poorly developed posterior face of the petiole than is characteristic of *Neocerapachys*. Other dorylines found in the New World include the army ants, and males in these genera always have marginal cell closed by R·f3 and Rs·f5, only one well-differentiated waist segment, and no constriction between abdominal segments III and IV. The remaining neotropical genus, *Leptanilloides*, has no conspicuous tegula and venation reduced, without R·f3 or discal cell.

#### Description.

***Worker.*** *Head*: Antennae with 12 segments. Apical antennal segment moderately enlarged, broader than and about equal in length to two preceding segments combined. Clypeus without cuticular apron. Lateroclypeal teeth absent. Parafrontal ridges absent. Torulo-posttorular complex horizontal. Antennal scrobes absent. Labrum with median notch or concavity. Proximal face of stipes projecting beyond inner margin of sclerite, concealing prementum when mouthparts fully closed. Maxillary palps 2-segmented. Labial palps 3-segmented. Mandibles triangular, with median tooth or triangular, edentate. Eyes present, composed of 1–20 ommatidia. Ocelli absent. Head capsule with differentiated vertical posterior surface above occipital foramen. Ventrolateral margins of head without lamella or ridge extending towards mandibles and beyond carina surrounding occipital foramen. Posterior head corners dorsolaterally immarginate. Carina surrounding occipital foramen absent or present. *Mesosoma*: Pronotal flange not separated from collar by distinct ridge. Promesonotal connection with Pronotomesopleural suture completely fused. Pronotomesopleural suture visible, unfused partway to notal surface. Mesometapleural groove replaced by cuticular ridge. Transverse groove dividing mesopleuron absent. Pleural endophragmal pit concavity present. Mesosoma dorsolaterally weakly to conspicuously marginate. Metanotal depression or groove on mesosoma absent. Propodeal spiracle situated low or high on sclerite. Propodeal declivity with distinct dorsal edge or margin and rectangular in posterior view. Metapleural gland with bulla visible through cuticle. Propodeal lobes present, well developed. *Metasoma*: Petiole anterodorsally immarginate, dorsolaterally marginate, and laterally above spiracle marginate. Helcium in relation to tergosternal Pronotomesopleural suture placed at posttergite and supraaxial. Prora forming a simple U-shaped margin or reduced to small longitudinal ridge. Spiracle openings of abdominal segments IV–VI circular. Abdominal segment III anterodorsally immarginate and dorsolaterally immarginate. Abdominal segment III more than half size of succeeding segment IV, which is weakly constricted at presegmental portion (uninodal waist). Girdling constriction of segment IV present, i.e. pre- and postsclerites distinct. Cinctus of abdominal segment IV gutter-like, not sculptured or weakly cross-ribbed. Abdominal segment IV not conspicuously largest segment. Abdominal tergite IV not folding over sternite, and anterior portions of sternite and tergite equally well visible in lateral view. Girdling constriction between pre- and posttergites of abdominal segments V and VI absent. Girdling constriction between pre- and poststernites of abdominal segments V and VI absent or present. Pygidium large, with impressed medial field, and armed with modified setae. Hypopygium unarmed. *Legs*: Mid tibia with single pectinate spur. Hind tibia with single pectinate spur. Hind basitarsus not widening distally, circular in cross-section. Posterior flange of hind coxa not produced as raised lamella. Metatibial gland absent or oval patch of whitish cuticle. Metabasitarsal gland absent. Hind pretarsal claws simple. *Polymorphism*: Monomorphic.

***Male.*** *Head*: Antennae with 12 or 13 segments. Antennal scapes dorsoventrally flattened. Clypeus with cuticular apron, not translucent. Parafrontal ridges absent. Torulo-posttorular complex vertical. Maxillary palps 2-segmented. Labial palps 3-segmented. Mandibles triangular, edentate. Ventrolateral margins of head without lamella or ridge extending towards mandibles and beyond carina surrounding occipital foramen. Carina surrounding occipital foramen ventrally absent. *Mesosoma*: Pronotal flange not separated from collar by distinct ridge. Notauli absent or present. Transverse groove dividing mesopleuron absent or present. Propodeal declivity reduced, without distinct dorsal edge or margin. Metapleural gland opening present. Propodeal lobes present. *Metasoma*: Petiole anterodorsally immarginate, dorsolaterally immarginate, and laterally above spiracle immarginate. Helcium in relation to tergosternal Pronotomesopleural suture placed at posttergite and supraaxial. Prora forming a simple U-shaped margin or V-shaped protrusion. Spiracle openings of abdominal segments IV–VI circular. Abdominal segment III more than half size of succeeding segment IV; latter weakly constricted at presegmental portion (uninodal waist). Girdling constriction of segment IV present, i.e. pre- and postsclerites distinct. Cinctus of abdominal segment IV gutter-like, not sculptured. Girdling constriction between pre- and postsclerites of abdominal segments V and VI absent or present. Abdominal segment IV not conspicuously largest segment. Abdominal sternite VII simple. Abdominal sternite IX distally armed with two spines, with lateral apodemes about as long as medial apodeme, directed anteriorly (towards head). *Genitalia*: Cupula long relative to rest of genital capsule and shorter ventrally than dorsally. Basimere broadly fused to telomere, with no sulcus trace at junction, and ventrally with left and right arms abutting or separated. Telomere gradually tapering toward apex. Volsella gradually tapering toward apex. Penisvalva laterally flattened, at apex hooked ventrally. *Legs*: Mid tibia with single pectinate spur. Hind tibia with single pectinate spur. Posterior flange of hind coxa not produced as raised lamella. Metatibial gland absent. Metabasitarsal glands absent. Hind pretarsal claws simple. *Wings*: Tegula present, broad, demiovate or narrow, demilanceolate in shape. Abscissa R·f3 present and running toward distal wing margin but not enclosing cell with Rs·f5. Abscissae Rs·f2–3 absent or present, connecting with Rs+M&M·f2. Cross-vein 2r-rs present, connected to Rs·f2–3&Rs·f4, differentiated from Rs·f4 by presence of Rs·f2–3. Abscissae Rs·f4–5 present, fused in absence of 2rs-m or differentiated into Rs·f4 and Rs·f5 by 2rs-m. Abscissa M·f2 in fore wing contiguous with Rs+M. Abscissa M·f2 in fore wing present, separated from Rs+M by Rs·f2. Abscissa M·f4 in fore wing present, not reaching wing margin. Cross-vein 1m-cu in fore wing present. Cross-vein cu-a in fore wing absent or present, arising from M+Cu and proximal to M·f1. Vein Cu in fore wing present, with only Cu1 branch prominent or with both branches Cu1 and Cu2. Vein A in fore wing with abscissae A·f1 and A·f2 present. Vein C in hind wing absent. Vein Sc+R+Rs present. Vein R in hind wing absent. Vein Sc+R in hind wing present. Abscissa Rs·f1 in hind wing present, shorter than 1rs-m, sometimes a stub. Abscissa Rs·f1 in hind wing present, longer than 1rs-m. Abscissa Rs·f2 in hind wing present, not reaching wing margin. Cross-vein 1rs-m in hind wing absent or present, about as long as M·f1. Vein M+Cu in hind wing present. Abscissa M·f1 in hind wing absent or present. Abscissa M·f2 in hind wing absent or present. Cross-vein cu-a in hind wing absent or present. Vein Cu in hind wing absent or present. Vein A in hind wing absent or with abscissa A·f1 present.

***Gyne.*** *Acanthostichus* gynes are known either as alates or subdichthadiiform, i.e. ergatoid without wing sclerites but possessing hypertrophied gasters. The former are currently known for *Acanthostichus emmae* and *Acanthostichus texanus*, the latter in *Acanthostichus brevicornis*, *Acanthostichus quadratus*, and *Acanthostichus laticornis*. In the fully alate gynes the eyes are large and three ocelli are present and abdominal segment III is differentiated from succeeding segments. In the subdichthadiigynes the eyes are present but small, three small ocelli are present, the head is more round than in workers, and mandibles are falcate; there are no flight-associated sclerites, abdominal segment II (petiole) is broadly attached posteriorly to segment III, which is also enlarged, not separated from the rest of the gaster by a constriction (Emery 1895, MacKay 1996). The distinction between alate versus wingless gynes was the basis for the separation of the genus *Ctenopyga* from *Acanthostichus* (see above; Brown 1975, MacKay 1996).

***Larva.*** Described in Emery (1899c), Bruch (1925). Cocoons absent.

#### Distribution.

*Acanthostichus* is a genus of 24 described species, occurring in southern United States, Mexico, and most of South America. The genus has long been thought absent from Central America, but at least one specimen is known from Costa Rica. This is unlikely due to undersampling, as Central American countries have been the subject to some of the most intensive surveys of ant faunas (Longino et al. 2014). *Acanthostichus hispaniolicus* has been described from Dominican amber (Miocene) of Hispaniola.

#### Taxonomy and phylogeny.

*Acanthostichus* was erected by Mayr (1887) for the species *Typhlopone serratula* Smith 1858, then known only from workers. Ashmead (1906) later introduced the genus *Ctenopyga*, based on an alate gyne and males. He differentiated it from *Acanthostichus* based on the gyne morphology, as by then wingless gynes were found in *Acanthostichus* (Emery 1895l). MacKay (1996) synonymized the otherwise remarkably similar *Ctenopyga* under *Acanthostichus* and I follow his decision here. MacKay (1996) also revised the genus and provided keys to all species, later adding one more species and describing a gyne of *Acanthostichus brevicornis* (MacKay 2004). De Andrade described the species from Dominican amber (de Andrade 1998b).

Males described by Marion Smith (1942b) and attributed to ‘*Cerapachys*’ (here *Syscia*) *augustae* and *Cerapachys davisi* match the morphology of *Acanthostichus* males. A specimen of *Cerapachys davisi* was also included in a molecular phylogeny and was shown to be a close relative of *Acanthostichus punctiscapus*. Therefore *davisi* was transferred to *Acanthostichus* (Brady et al. 2014). It is possible that Smith’s putative males of *Syscia augustae* and *Acanthostichus davisi* will turn out to be conspecific with *Acanthostichus arizonensis* and *Acanthostichus punctiscapus*, respectively. MacKay (1996) collected numerous males of *Acanthostichus davisi* (then recognized as *Cerapachys*) at the type locality of *Acanthostichus punctiscapus*.

It is now established that *Acanthostichus* is most closely related to *Cylindromyrmex* (Brady and Ward 2005, Brady et al. 2006, Brady et al. 2014, Borowiec, in prep.). There have been no efforts to infer the internal phylogeny of the genus, but MacKay divided *Acanthostichus* into three species groups based on morphology (MacKay 1996).

#### Biology.

Along with their close relatives in *Cylindromyrmex*, *Acanthostichus* species are predators of termites, unlike most other doryline species which prey on ants (Kusnezov 1962, Brown 1975, MacKay 1996). *Acanthostichus truncatus* has been observed to raid an arboreal termite nest (MacKay 1996) and *Acanthostichus hispaniolicus* is known from multiple specimens in Dominican amber suggesting that this species was also an arboreal forager. Unlike *Cylindromyrmex*, however, they nest in soil, under stones, and in rotting wood (Kusnezov 1962a, MacKay 1996). These ants are rarely encountered and little is known of *Acanthostichus* ecology, nest size or other particulars of their biology. It is unclear whether brood production is synchronized.

#### Species of *Acanthostichus*

*Acanthostichus arizonensis* Mackay, W.P., 1996: United States

*Acanthostichus bentoni* Mackay, W.P., 1996: Brazil

*Acanthostichus brevicornis* Emery, 1894: French Guiana

*Acanthostichus brevinodis* Mackay, W.P., 1996: Brazil

*Acanthostichus concavinodis* Mackay, W.P., 1996: Bolivia

*Acanthostichus davisi* (Smith, M. R., 1942a): United States

*Acanthostichus emmae* Mackay, W.P., 1996: Mexico

*Acanthostichus femoralis* Kusnezov, 1962: Argentina

*Acanthostichus flexuosus* Mackay, W.P., 1996: Brazil

*Acanthostichus fuscipennis* Emery, 1895b: Brazil

†*Acanthostichus hispaniolicus* De Andrade, 1998b: Dominican amber

*Acanthostichus kirbyi* Emery, 1895b: Paraguay

*Acanthostichus laevigatus* Mackay, W.P., 1996: Venezuela

*Acanthostichus laticornis* Forel, 1908: Paraguay

*Acanthostichus lattkei* Mackay, W.P., 1996: Venezuela

*Acanthostichus longinodis* Mackay, W.P., 2004: Paraguay

*Acanthostichus punctiscapus* Mackay, W.P., 1996: United States

*Acanthostichus quadratus* Emery, 1895: Bolivia

*Acanthostichus quirozi* Mackay, W.P., 1996: Mexico

*Acanthostichus sanchezorum* Mackay, W.P., 1985: Colombia

*Acanthostichus serratulus* (Smith, F., 1858): Brazil

*Acanthostichus skwarrae* Wheeler, W. M., 1934: Mexico

*Acanthostichus texanus* Forel, 1904: United States

*Acanthostichus truncatus* Mackay, W.P., 1996: Colombia

### Aenictogiton

Taxon classificationAnimaliaHymenopteraFormicidae

Emery, 1901b

#### Type-species.

*Aenictogiton fossiceps*, by monotypy.

This Afrotropical genus was until recently known only from male specimens and little is known about its biology except that it is likely a subterranean nester and forager.

#### Diagnosis.

***Worker.*** The workers of the one *Aenictogiton* species for which this caste is known so far are unique in having propodeal spiracles situated high on the sclerite and propodeal lobes reduced, pygidium large but not armed with modified setae, and possessing marked constrictions between abdominal segments IV, V, and VI. Small body size is also characteristic, with mesosoma length under 0.65 mm in the only species known from workers. The same characters will serve to distinguish *Aenictogiton* from other Afrotropical dorylines that either have spiracles situated low on the propodeum, propodeal lobes well-developed and pygidium armed (*Eburopone*, *Lioponera*, *Ooceraea*, *Parasyscia*, *Zasphinctus*) or are markedly larger and have at most weakly impressed abdominal sternites at junction of segments IV, V, and VI, never conspicuous constrictions on both tergites and sternites (*Aenictus*, *Dorylus*).

***Male.*** *Aenictogiton* males have distinctive wing venation where cross-vein cu-a in the fore wing arises proximal to M·f1, R·f3 is absent and is Rs·f3 ‘hanging’ free in the submarginal cell in the absence of Rs·f2. This, combined with the ‘army ant-like’ habitus that includes the lack of constriction between abdominal segments III and IV (no postpetiole), will serve to distinguish it from all other dorylines. The two other army ant genera that occur in the Afrotropics, *Aenictus* and *Dorylus*, do not have free Rs·f3 in fore wings.

#### Description.

***Worker.*** *Head*: Antennae with 12 segments. Apical antennal segment moderately enlarged, broader than and about equal in length to two preceding segments combined. Clypeus without cuticular apron. Lateroclypeal teeth absent. Parafrontal ridges reduced. Torulo-posttorular complex vertical. Antennal scrobes absent. Labrum without median notch or concavity. Proximal face of stipes projecting beyond inner margin of sclerite, concealing prementum when mouthparts fully closed. Maxillary palps 1-segmented. Labial palps 1-segmented. Mandibles falcate, with teeth on elongated masticatory margin. Eyes absent. Ocelli absent. Head capsule with differentiated vertical posterior surface above occipital foramen. Ventrolateral margins of head without lamella or ridge extending towards mandibles and beyond carina surrounding occipital foramen. Posterior head corners dorsolaterally immarginate. Carina surrounding occipital foramen ventrally absent. *Mesosoma*: Pronotal flange not separated from collar by distinct ridge. Promesonotal connection with Pronotomesopleural suture conspicuous and complete, but immobile. Pronotomesopleural suture visible, unfused up to notal surface. Mesometapleural groove deeply impressed, conspicuous. Transverse groove dividing mesopleuron absent. Pleural endophragmal pit concavity absent, but a minute pit present. Mesosoma dorsolaterally immarginate. Metanotal depression or groove on mesosoma absent. Propodeal spiracle situated high on sclerite. Propodeal declivity without distinct dorsal edge or margin and rectangular in posterior view. Metapleural gland with bulla visible through cuticle. Propodeal lobes absent. *Metasoma*: Petiole anterodorsally immarginate, dorsolaterally immarginate, laterally above spiracle immarginate. Helcium in relation to tergosternal Pronotomesopleural suture placed at posttergite and axial. Prora forming a simple U-shaped margin. Spiracle openings of abdominal segments IV–VI circular. Abdominal segment III anterodorsally immarginate and dorsolaterally immarginate. Abdominal segment III more than half size of succeeding segment IV, which is weakly constricted at presegmental portion (uninodal waist). Girdling constriction of segment IV present, i.e. pre- and postsclerites distinct. Cinctus of abdominal segment IV gutter-like and sculptured but not cross-ribbed. Abdominal segment IV not conspicuously largest segment. Abdominal tergite IV not folding over sternite, and anterior portions of sternite and tergite equally well visible in lateral view. Girdling constriction between pre- and posttergites of abdominal segments V and VI present. Girdling constriction between pre- and poststernites of abdominal segments V and VI present. Pygidium large, without impressed medial field, and simple, not armed with cuticular spines or modified setae. Hypopygium unarmed. *Legs*: Mid tibia with single simple/barbulate spur. Hind tibia with single pectinate spur. Hind basitarsus not widening distally, circular in cross-section. Posterior flange of hind coxa not produced as raised lamella. Metatibial gland present as oval patch of whitish cuticle. Metabasitarsal gland absent. Hind pretarsal claws simple. *Polymorphism*: Apparently monomorphic.

***Male.*** *Head*: Antennae with 13 segments. Clypeus without cuticular apron. Parafrontal ridges absent. Torulo-posttorular complex vertical, reduced small, single vertical carina. Maxillary palps 1-segmented. Labial palps 1-segmented. Mandibles falcate. Ventrolateral margins of head without lamella or ridge extending towards mandibles and beyond carina surrounding occipital foramen. Carina surrounding occipital foramen ventrally absent. *Mesosoma*: Pronotal flange not separated from collar by distinct ridge. Notauli absent. Transverse groove dividing mesopleuron absent but horizontal depression may be present. Propodeal declivity reduced, without distinct dorsal edge or margin. Metapleural gland opening absent. Propodeal lobes absent. *Metasoma*: Petiole anterodorsally immarginate, dorsolaterally immarginate, and laterally above spiracle immarginate. Helcium in relation to tergosternal Pronotomesopleural suture placed at Pronotomesopleural suture and axial. Prora simple, not delimited by carina. Spiracle openings of abdominal segments IV–VI circular. Abdominal segment III more than half size of succeeding segment IV; latter weakly constricted at presegmental portion (uninodal waist). Girdling constriction of segment IV absent, i.e. pre- and postsclerites indistinct. Cinctus of abdominal segment IV absent, not impressed. Girdling constriction between pre- and postsclerites of abdominal segments V and VI absent. Abdominal segment IV not conspicuously largest segment. Abdominal sternite VII simple. Abdominal sternite IX distally armed with two spines, with lateral apodemes about as long as medial apodeme, directed anteriorly (towards head); all apodemes very short. *Genitalia*: Cupula long relative to rest of genital capsule and shorter ventrally than dorsally. Basimere broadly fused to telomere, with no sulcus trace at junction, and ventrally with left and right arms abutting. Telomere expanded at apex. Volsella narrow, hook-shaped, occasionally forming two hooks at apex. Penisvalva laterally compressed, narrow and lance-shaped at apex. *Legs*: Mid tibia with pectinate and simple spur. Hind tibia with pectinate and simple spur. Posterior flange of hind coxa not produced as raised lamella. Metatibial gland absent. Metabasitarsal glands absent. Hind pretarsal claws simple. *Wings*: Tegula present, broad, demiovate in shape. Vein C in fore wing present. Pterostigma broad. Abscissa R·f3 absent. Abscissae Rs·f2–3 present, disconnected from Rs+M. Cross-vein 2r-rs present, connected to Rs·f2–3&Rs·f4. Abscissae Rs·f4–5 differentiated into Rs·f4 and Rs·f5 by 2rs-m. Abscissa M·f2 in fore wing contiguous with Rs+M. Abscissa M·f4 in fore wing present, not reaching wing margin. Cross-vein 1m-cu in fore wing present. Cross-vein cu-a in fore wing present, arising from M+Cu and proximal to M·f1. Vein Cu in fore wing present, with only Cu1 branch prominent. Vein A in fore wing with abscissae A·f1 and A·f2 present. Vein C in hind wing absent. Vein Sc+R+Rs present. Vein R in hind wing present, extending past Sc+R but not reaching distal wing margin. Vein Sc+R in hind wing present. Abscissa Rs·f1 in hind wing present, contiguous with Rs·f2. Abscissa Rs·f2 in hind wing present, not reaching wing margin. Cross-vein 1rs-m in hind wing absent. Vein M+Cu in hind wing present. Abscissa M·f1 in hind wing present. Abscissa M·f2 in hind wing present. Cross-vein cu-a in hind wing present. Vein Cu in hind wing present. Vein A in hind wing with abscissae A·f1 and A·f2 present.

***Gyne.*** Not described.

***Larva.*** Not described.

#### Distribution.

This is an exclusively Afrotropical lineage and most species have been described from the Congo Basin but records extend to southern and eastern Africa.

#### Taxonomy and phylogeny.

The taxonomic history of *Aenictogiton* begins with Emery’s description of *Aenictogiton fossiceps*, a male-based taxon that he placed in the Dorylinae (Emery 1901d). Subsequently, six other male-based species were described from the territory of Democratic Republic of the Congo. Santschi (1924) gave a key to all the species then known from males. The worker caste of *Aenictogiton* remained a mystery for over a century, until it was discovered in Uganda in 2008 and then collected again in 2012 in the same country. The genus has been most often collected from the Congo Basin (Brown 1975), although there are records from southern Angola, northern Namibia (Parr et al. 2003), and southwestern Kenya (Hita Garcia et al. 2009).

*Aenictogiton* is the sister taxon to *Dorylus* (Brady et al. 2006, Brady et al. 2014, Borowiec, in prep.).

#### Biology.

Virtually nothing is known about the biology of *Aenictogiton*. Most records of males coming to light are associated with forest habitats (Brown 1975), except the savanna/woodland record from Namibia (Parr et al. 2003). The Ugandan workers collected in 2012 come from leaf litter sifted near a log in a moist evergreen forest in Kibale National Park. The mode of foraging, brood production, and colony life cycle remain unknown.

#### Species of *Aenictogiton*

*Aenictogiton attenuatus* Santschi, 1919b: Democratic Republic of the Congo

*Aenictogiton bequaerti* Forel, 1913a: Democratic Republic of the Congo

*Aenictogiton elongatus* Santschi, 1919b: Democratic Republic of the Congo

*Aenictogiton emeryi* Forel, 1913a: Democratic Republic of the Congo

*Aenictogiton fossiceps* Emery, 1901b: Democratic Republic of the Congo

*Aenictogiton schoutedeni* Santschi, 1924: Democratic Republic of the Congo

*Aenictogiton sulcatus* Santschi, 1919b: Democratic Republic of the Congo

### Aenictus

Taxon classificationAnimaliaHymenopteraFormicidae

Shuckard, 1840b

= Paraenictus Wheeler, W. M., 1929
= Typhlatta Smith, 1857

#### Type-species.

*Aenictus ambiguus*, by original designation.

This Old World lineage contains some of the more conspicuous army ants and is the largest doryline genus with 183 described species.

#### Diagnosis.

***Worker.*** The workers of *Aenictus* be recognized by a combination of 8 to 10-segmented antennae, propodeal spiracle positioned high on the propodeum, and conspicuously binodal waist (abdominal segment IV is conspicuously the largest abdominal segment). *Aenictus* is most similar to the New World genus *Neivamyrmex*, which can be distinguished by 12-segmented antennae. Two other army ant genera co-occur with *Aenictus*: *Aenictogiton* and *Dorylus*. In *Aenictogiton* there are also constrictions between abdominal segments IV–VI, absent from *Aenictus*. *Dorylus* has a uninodal waist with no tapering towards the anterior of abdominal segment IV.

***Male.*** The males of *Aenictus* are of decidedly army ant-like habitus and distinguishable from other dorylines by a combination of single segment in the waist, femora never extremely flattened relative to tibia, M·f1 vein of fore wing situated distal or near to cu-a, Rs·f2–3 absent, pterostigma broad and conspicuous. All New World army ant genera with similar habitus can be distinguished by fore wing venation, in particular presence of Rs·f2–3 and marginal cell closed along the leading edge by R·f3 connected to Rs·f5. In the Old World, *Aenictogiton* males can be easily told apart by their ‘hanging’ Rs·f2–3 vein in the fore wing, while *Dorylus* have a narrow pterostigma and dramatically flattened femora that contrast with tibiae that are more circular in cross-section.

#### Description.

***Worker.*** *Head*: Antennae with 8, 9, or 10 segments. Apical antennal segment not enlarged, not broader and longer than two preceding segments combined to moderately enlarged, broader than and about equal in length to two preceding segments combined. Clypeus with cuticular apron. Lateroclypeal teeth absent. Parafrontal ridges absent or reduced. Torulo-posttorular complex vertical. Antennal scrobes absent. Labrum with median notch or concavity. Proximal face of stipes not projecting beyond inner margin of sclerite, prementum exposed when mouthparts fully closed. Maxillary palps 2-segmented. Labial palps 2-segmented. Mandibles triangular, with teeth or with one median tooth, or falcate. Eyes absent. Ocelli absent. Head capsule with differentiated vertical posterior surface above occipital foramen; in some species differentiation weak. Ventrolateral margins of head without lamella or ridge extending towards mandibles and beyond carina surrounding occipital foramen. Posterior head corners dorsolaterally immarginate. Carina surrounding occipital foramen ventrally absent. *Mesosoma*: Pronotal flange not separated from collar by distinct ridge. Promesonotal connection with Pronotomesopleural suture completely fused. Pronotomesopleural suture completely fused; *Aenictus philippinensis* group species with grooved cuticular lip anteriorly. Mesometapleural groove not impressed to deeply impressed, conspicuous. Transverse groove dividing mesopleuron absent. Pleural endophragmal pit concavity absent. Mesosoma dorsolaterally immarginate. Metanotal depression or groove on mesosoma absent or present. Propodeal spiracle situated high on sclerite. Propodeal declivity with or without distinct dorsal edge or margin and triangular or broadly oval in posterior view. Metapleural gland with bulla visible through cuticle. Propodeal lobes present, short. *Metasoma*: Petiole anterodorsally marginate with carina low on anterior face, dorsolaterally immarginate, and laterally above spiracle immarginate or marginate. Helcium in relation to tergosternal Pronotomesopleural suture placed at posttergite and infraaxial. Prora forming a V-shaped protrusion or narrowed into anteriorly directed spine. Spiracle openings of abdominal segments IV–VI circular. Abdominal segment III anterodorsally immarginate, dorsolaterally immarginate. Abdominal segment III about half size of succeeding segment IV, which is strongly constricted at presegmental portion (binodal waist). Girdling constriction of segment IV present, i.e. pre- and postsclerites distinct. Cinctus of abdominal segment IV a gradual concavity, not gutter-like. Abdominal segment IV conspicuously the largest segment. Abdominal tergite IV not folding over sternite, and anterior portions of sternite and tergite equally well visible in lateral view. Girdling constriction between pre- and posttergites of abdominal segments V and VI absent. Girdling constriction between pre- and poststernites of abdominal segments V and VI absent. Pygidium small, reduced to narrow strip, without impressed medial field and simple, not armed with cuticular spines or modified setae. Hypopygium unarmed. *Legs*: Mid tibia with two spurs, one barbulate and one simple, or with two simple spurs. Hind tibia with two barbulate/simple spurs or with one barbulate and one pectinate spur. Hind basitarsus not widening distally, circular in cross-section. Posterior flange of hind coxa not produced as raised lamella. Metatibial gland present as oval patch of whitish cuticle to patch occupying at least half of tibia length. Metabasitarsal gland absent. Hind pretarsal claws simple. *Polymorphism*: Monomorphic to moderately polymorphic.

***Male.*** *Head*: Antennae with 13 segments. Clypeus without cuticular apron. Parafrontal ridges absent. Torulo-posttorular complex vertical, reduced to vertical carina or entirely absent. Maxillary palps 2-segmented. Labial palps 1-segmented. Mandibles falcate. Ventrolateral margins of head without lamella or ridge extending towards mandibles and beyond carina surrounding occipital foramen. Carina surrounding occipital foramen ventrally absent. *Mesosoma*: Pronotal flange not separated from collar by distinct ridge. Notauli absent. Transverse groove dividing mesopleuron absent. Propodeal declivity reduced, without distinct dorsal edge or margin. Metapleural gland opening absent. Propodeal lobes absent. *Metasoma*: Petiole anterodorsally immarginate, dorsolaterally immarginate, and laterally above spiracle immarginate. Helcium in relation to tergosternal Pronotomesopleural suture placed at Pronotomesopleural suture and axial. Prora simple, not delimited by carina. Spiracle openings of abdominal segments IV–VI circular, oval, or slit-shaped. Abdominal segment III more than half size of succeeding segment IV; latter weakly constricted at presegmental portion (uninodal waist). Girdling constriction of segment IV absent, i.e. pre- and postsclerites indistinct. Cinctus of abdominal segment IV absent, not impressed. Girdling constriction between pre- and postsclerites of abdominal segments V and VI absent. Abdominal segment IV not conspicuously largest segment. Abdominal sternite VII simple. Abdominal sternite IX distally armed with two spines, with lateral apodemes about as long as medial apodeme, directed anteriorly (towards head). *Genitalia*: Cupula strap-like, very short relative to rest of genital capsule and of approximately equal length on both dorsal and ventral surfaces. Basimere broadly fused to telomere, basimere with no sulcus trace at junction, and ventrally with left and right arms separated. Telomere expanded at apex. Volsella variable. Penisvalva not flattened at apex, expanded. *Legs*: Mid tibia without spurs or with two simple spurs. Hind tibia without spurs or with two simple spurs. Posterior flange of hind coxa not produced as raised lamella. Metatibial gland absent. Metabasitarsal glands absent. Hind pretarsal claws simple. *Wings*: Tegula present, narrow, demilanceolate in shape. Vein C in fore wing present. Pterostigma broad. Abscissa R·f3 absent. Abscissae Rs·f2–3 absent. Cross-vein 2r-rs present, connected to Rs·f2–3&Rs·f4. Abscissae Rs·f4–5 differentiated into Rs·f4 and Rs·f5 by 2rs-m. Abscissa M·f2 in fore wing contiguous with Rs+M. Abscissa M·f4 in fore wing present, reaching or not reaching wing margin. Cross-vein 1m-cu in fore wing present. Cross-vein cu-a in fore wing present, arising from Cu and distal to, at or near M·f1. Vein Cu in fore wing present, with only Cu1 branch prominent. Vein A in fore wing with abscissae A·f1 and A·f2 present. Vein C in hind wing absent. Vein R in hind wing absent or present, extending past Sc+R but not reaching distal wing margin. Vein Sc+R in hind wing present. Abscissa Rs·f1 in hind wing present, contiguous with Rs·f2. Abscissa Rs·f2 in hind wing present, not reaching wing margin. Cross-vein 1rs-m in hind wing present, about as long as M·f1. Vein M+Cu in hind wing present. Abscissa M·f1 in hind wing present. Abscissa M·f2 in hind wing present. Cross-vein cu-a in hind wing present. Vein Cu in hind wing present. Vein A in hind wing with abscissae A·f1 and A·f2 present.

***Gyne.*** Dichthadiiform, blind and with one or none ocelli, so far known in 13 species (Bharti 2003).

***Larva.*** Larvae of several Indomalayan and Australasian *Aenictus* species have been described (Wheeler 1943, Wheeler and Wheeler 1964b, 1984, 1990). Cocoons are absent.

#### Distribution.

*Aenictus* is widely distributed in the Old World. The vast majority of species is found in Southeast Asia, with the Afrotropics being the other center of diversity. A few species range into the southern parts of the Palearctic region, and there is a number of species known from Australia.

#### Taxonomy and phylogeny.

The phylogenetic position of *Aenictus* has been difficult to infer. Phylogenomic data suggests that it is sister to the *Aenictogiton* plus *Dorylus* clade but they also show that these two lineages diverged very long ago, most likely in the Cretaceous (Borowiec, in prep.). The comprehensive morphology-based study of Brady and Ward (2005) placed it sister to *Aenictogiton* plus *Dorylus*; subsequent molecular analyses recovered it sister to New World army ants (Brady et al. 2006) and, later, sister to the *Aenictogiton* plus *Dorylus* clade, although with low support (Brady et al. 2014). The internal phylogeny shows that the African species of *Aenictus* are nested within South East Asian forms (Munetoshi Maruyama pers. comm.; Borowiec, in prep.).

*Aenictus* was first described based on a male from India, named for its ‘aenigmatical structure’ by Shuckard (1840b). Shuckard correctly recognized its affinity to other doryline ants, but the worker caste was not known at the time. Frederick Smith (1857) described a new genus based on workers, *Typhlatta* from Borneo, from material collected by Alfred Russell Wallace. It was not until 1890 that the male and workers of these ants were collected together (Forel 1890c).

The trend of describing unassociated males unfortunately continued and *Aenictus* is an example of ‘dual taxonomy’. Many names are either worker- or male-based, and there is no single species known from workers, queens and males (Gotwald and Leroux 1980, Bolton 2003). The internal phylogeny of the genus has been tackled with the cladistics analysis of Wilson (1964) and a phenetic study of quantitative traits (Gotwald and Barr 1988). As of this writing, Munetoshi Maruyama (pers. comm.) is working on a comprehensive molecular phylogeny of the genus. The taxonomy of the Asian forms received most attention and was first the subject of a thorough revision of Wilson (1964), recently followed by a long series of studies that described many new taxa and provided new keys for most of the species groups (Bharti et al. 2012, Jaitrong and Eguchi 2010, Jaitrong and Hashimoto 2012, Jaitrong and Nur-Zati 2010, Jaitrong and Wiwatwitaya 2013, Jaitrong and Yamane 2010, 2011a, 2012b, 2013, Jaitrong et al. 2010, 2011, 2012, Li and Wang 2005, Liu et al. 2015, Mathew and Tiwari 2000, Staab 2014a, Staab 2015, Terayama and Yamane 1989, Terayama and Kubota 1993, Wang 2006, Wong and Guénard 2016, Wiwatwitaya and Jaitrong 2011, Yamane and Hashimoto 1999, Yamane and Wang 2015, Zettel and Sorger 2010, Zhou 2001, Zhou and Chen 1999). Jaitrong and Yamane (2011) established the current species-group classification and provided keys that make identifications in this large genus feasible. Shattuck (2008) revised the Australian species. In contrast to the Asian fauna, the taxonomy of African species has been largely neglected and never received a comprehensive treatment. Because of the above mentioned ‘dual taxonomy’ it is even difficult to give an estimate of the total number of species in the Afrotropical region, although Wilson (1964) estimated the number of species to be ‘at least 12’. Papers by Campione et al. (1983), Gotwald and Cunningham-van Someren (1976), and Gotwald and Leroux (1980) are the only modern references discussing taxonomy of Afrotropical *Aenictus*. Several species of the genus reach the Palearctic region; recently Aktaç et al. (2004) and Radchenko and Alipanah (2004) discussed the West Palearctic species and Sharaf et al. (2012) described an additional species from Saudi Arabia.

#### Biology.

Given the number of described species and their abundance and importance as insect predators in the Old World tropics, the biology of *Aenictus* is poorly studied. The impressive species and morphological diversity is likely reflected in the diversity of habits, although all thus far observed species seem to be specialized predators of other ants (but see Staab 2014b for a report on honeydew feeding). Members of some groups are known to form colonies of up to 80,000 individuals, forage above-ground in conspicuous columns and bivouac in semi-open spaces, while others are much more inconspicuous and cryptic. *Aenictus* queens synchronize brood production and colony life cycle goes through statary and nomadic phases (Schneirla and Reyes 1966). The nomadic phase lasts on average 14 days, about the same amount of time as in the Neotropical genera, but the statary phase is much longer and lasts 28 days, as opposed to 20 days in *Eciton*. During the nomadic phase in *Eciton* the daily colony emigrations always follow raids, whereas in *Aenictus* they can be initiated after a time of quiescence and occur without regularity, often multiple times a day. The descriptions of foraging behavior for several species are available; Wilson (1964) in his revision provides notes on foraging of selected species. Chapman (1965) recounts observations of a few species in the Philippines, mostly *Aenictus gracilis* and *Aenictus laeviceps*, and Schneirla and Reyes (1966, 1969) study these two epigaeic species in detail. Schneirla (1971) compares raiding and emigration behavior of *Aenictus laeviceps* to other army ants, *Eciton* and *Neivamyrmex*.

Rościszewski and Maschwitz (1994) and Hirosawa et al. (2000) studied prey specialization among sympatric *Aenictus* in Asia. Both studies found evidence of resource partitioning and observed differences in foraging strategies. Gotwald and Cunningham-van Someren (1976) and Gotwald (1976) are the only publications focusing on the behavior of African forms. At least some species support a community of myrmecophiles (Chapman 1965, Maruyama et al. 2009).

Billen and Gotwald (1988) described the anatomy of Dufour gland in three Asian *Aenictus* and argued that its structure, unusual among ants, shows affinity with *Dorylus*. Oldham et al. (1994) characterized the trail pheromone of *Aenictus* species related to *Aenictus laeviceps* and demonstrated that it is produced by the postpygidial gland and Billen et al. (1999) further studied the structure of this gland. Hölldobler et al. (1996) described the histology and ultrastructure of the metatibial gland in *Aenictus ceylonicus*.

#### Species of *Aenictus*

*Aenictus abeillei* (André, 1886): Algeria

*Aenictus acerbus* Shattuck, 2008: Australia

*Aenictus aitkenii* Forel, 1901a: India

*Aenictus alluaudi* Santschi, 1910c: Kenya

*Aenictus alluaudi falcifer* Santschi, 1924: Democratic Republic of the Congo

*Aenictus alticola* Wheeler, W. M. and Chapman, 1930a: Philippines

*Aenictus ambiguus* Shuckard, 1840b: India

*Aenictus anceps* Forel, 1910b: Eritrea

*Aenictus annae* Forel, 1911a: Indonesia (Java)

*Aenictus appressipilosus* Jaitrong andYamane, 2013: Malaysia (Sabah)

*Aenictus arabicus* Sharaf and Aldawood, 2012: Saudi Arabia

*Aenictus aratus* Forel, 1900a: Australia

*Aenictus artipus* Wilson, 1964: Thailand

*Aenictus arya* Forel, 1901a: India

*Aenictus asantei* Campione, Novak and Gotwald, 1983: Ghana

*Aenictus asperivalvus* Santschi, 1919a: Ivory Coast

*Aenictus bakeri* Menozzi, 1925: Philippines

*Aenictus baliensis* Jaitrong and Yamane, 2013: Indonesia (Bali)

*Aenictus bayoni* Menozzi, 1932: Uganda

*Aenictus binghami* Forel, 1900a: Myanmar

*Aenictus biroi* Forel, 1907a: Sri Lanka

*Aenictus bobaiensis* Zhou and Chen, 1999: China

*Aenictus bodongjaya* Jaitrong and Yamane, 2011a: Indonesia (Sumatra)

*Aenictus bottegoi* Emery, 1899a: Ethiopia

*Aenictus bottegoi noctivagus* Santschi, 1913: Ethiopia

*Aenictus brazzai* Santschi, 1910: Republic of the Congo

*Aenictus breviceps* Forel, 1912b: Indonesia (Java)

*Aenictus brevicornis* (Mayr, 1879): India

*Aenictus brevinodus* Jaitrong and Yamane, 2011a: Indonesia (Sulawesi)

*Aenictus brevipodus* Jaitrong and Yamane, 2013: Vietnam

*Aenictus buttelreepeni* Forel, 1913c: Indonesia (Sumatra)

*Aenictus buttgenbachi* Forel, 1913a: Democratic Republic of the Congo

*Aenictus camposi* Wheeler, W. M. and Chapman, 1925: Philippines

*Aenictus carolianus* Zettel and Sorger, 2010: Philippines

*Aenictus certus* Westwood, 1842: India

*Aenictus ceylonicus* (Mayr, 1866a): Sri Lanka

*Aenictus changmaianus* Terayama and Kubota, 1993: Thailand

*Aenictus chapmani* Wilson, 1964: Papua New Guinea

*Aenictus clavatus* Forel, 1901a: India

*Aenictus clavatus atripennis* Forel, 1913c: Indonesia (Sumatra)

*Aenictus clavatus kanariensis* Forel, 1901a: India

*Aenictus clavatus sundaicus* Forel, 1909c: Indonesia (Java)

*Aenictus clavitibia* Forel, 1901a: India

*Aenictus clavitibia facetus* Forel, 1911a: Indonesia (Java)

*Aenictus concavus* Jaitrong and Yamane, 2013: Thailand

*Aenictus congolensis* Santschi, 1911a: ‘Congo français’

*Aenictus cornutus* Forel, 1900a: Malaysia (Sarawak)

*Aenictus crucifer* Santschi, 1914a: Kenya

*Aenictus crucifer tuberculatus* Arnold, 1915: Zimbabwe

*Aenictus currax* Emery, 1900a: Papua New Guinea

*Aenictus cylindripetiolus* Jaitrong and Yamane, 2013: Thailand

*Aenictus decolor* (Mayr, 1879): ‘Ost-Afrika’

*Aenictus dentatus* Forel, 1911c: Malaysia (Negeri Sembilan)

*Aenictus diclops* Shattuck, 2008: Australia

*Aenictus dlusskyi* Arnol’di, 1968: Armenia

*Aenictus doryloides* Wilson, 1964: India

*Aenictus doydeei* Jaitrong and Yamane, 2011b: Laos,

*Aenictus duengkaei* Jaitrong and Yamane, 2012: Thailand

*Aenictus eguchii* Jaitrong and Yamane, 2013: Vietnam

*Aenictus eugenii* Emery, 1895a: South Africa

*Aenictus eugenii caroli* Forel, 1910b: Eritrea

*Aenictus eugenii henrii* Santschi, 1924: Democratic Republic of the Congo

*Aenictus exilis* Wilson, 1964: Papua New Guinea

*Aenictus feae* Emery, 1889: Myanmar

*Aenictus fergusoni* Forel, 1901a: India

*Aenictus foreli* Santschi, 1919a: Ivory Coast

*Aenictus formosensis* Forel, 1913b: Taiwan

*Aenictus fuchuanensis* Zhou, 2001: China

*Aenictus fulvus* Jaitrong and Yamane, 2011a: Thailand

*Aenictus furculatus* Santschi, 1919a: Senegal

*Aenictus furculatus andrieui* Santschi, 1930: Sudan

*Aenictus furibundus* Arnold, 1959: Zimbabwe

*Aenictus fuscipennis* Forel, 1913c: Indonesia (Sumatra)

*Aenictus fuscovarius* Gerstäcker, 1859: Mozambique

*Aenictus fuscovarius laetior* Forel, 1910b: Eritrea

*Aenictus fuscovarius magrettii* Emery, 1892: Sudan

*Aenictus fuscovarius sagittarius* Santschi, 1938: Egypt

*Aenictus gibbosus* Dalla Torre, 1893: Indonesia (Sumatra)

*Aenictus gibbosus ashaverus* Forel, 1913c: Indonesia

*Aenictus glabratus* Jaitrong and Nur-Zati, 2010: Malaysia (Selangor)

*Aenictus glabrinotum* Jaitrong and Yamane, 2011: Malaysia (Sabah)

*Aenictus gleadowii* Forel, 1901a: India

*Aenictus gonioccipus* Jaitrong and Yamane, 2013: Indonesia (Sulawesi)

*Aenictus gracilis* Emery, 1893b: Malaysia (Sarawak)

*Aenictus grandis* Bingham, 1903: Myanmar

*Aenictus gutianshanensis* Staab 2014a: China

*Aenictus hamifer* Emery, 1896d: Ethiopia/Somalia

*Aenictus hamifer spinosior* Stitz, 1917: Algeria

*Aenictus henanensis* Li and Wang, 2005: China

*Aenictus hilli* Clark, 1928: Australia

*Aenictus hodgsoni* Forel, 1901a: Myanmar

*Aenictus hoelldobleri* Staab, 2015: China

*Aenictus hottai* Terayama and Yamane, 1989: Indonesia (Sumatra)

*Aenictus humeralis* Santschi, 1910c: Mali

*Aenictus humeralis chevalieri* Santschi, 1910c: Senegal

*Aenictus humeralis viridans* Santschi, 1915: Benin

*Aenictus huonicus* Wilson, 1964: Papua New Guinea

*Aenictus icarus* Forel, 1911a: Indonesia (Java)

*Aenictus icarus incautus* Forel, 1911a: Indonesia (Java)

*Aenictus idoneus* Menozzi, 1928: Indonesia (Java)

*Aenictus inconspicuus* Westwood, 1845: South Africa

*Aenictus indicus* Bharti, Wachkoo and Kumar, 2012: India

*Aenictus inflatus* Yamane and Hashimoto, 1999: Malaysia (Sarawak)

*Aenictus itoi* Jaitrong and Yamane, 2013: Indonesia (Sumatra)

*Aenictus jacobsoni* Forel, 1909c: Indonesia (Java)

*Aenictus jarujini* Jaitrong and Yamane, 2010a: Thailand

*Aenictus javanus* Emery, 1896a: Indonesia (Java)

*Aenictus jawadwipa* Jaitrong and Yamane, 2013: Indonesia (Java)

*Aenictus khaoyaiensis* Jaitrong and Yamane, 2013: Thailand

*Aenictus kutai* Jaitrong and Yamane, 2013: Indonesia

*Aenictus laeviceps* (Smith, F., 1857): Malaysia (Sarawak)

*Aenictus latifemoratus* Terayama and Yamane, 1989: Indonesia (Sumatra)

*Aenictus latiscapus* Forel, 1901a: India

*Aenictus latiscapus fumatus* Wheeler, W. M., 1927: China

*Aenictus latiscapus sauteri* Forel, 1913b: Taiwan

*Aenictus leliepvrei* Bernard, 1953a: Algeria

*Aenictus leptotyphlatta* Jaitrong and Eguchi, 2010: Thailand

*Aenictus levior* (Karavaiev, 1926): Indonesia (Buru Is.)

*Aenictus lifuiae* Terayama, 1984: Taiwan

*Aenictus longi* Forel, 1901a: India

*Aenictus longi taivanae* Forel, 1913b: Taiwan

*Aenictus longicephalus* Jaitrong and Yamane, 2013: Indonesia (Lombok)

*Aenictus longinodus* Jaitrong and Yamane, 2012: Thailand

*Aenictus luteus* Emery, 1892: Sierra Leone

*Aenictus luteus moestus* Santschi, 1930: Mali

*Aenictus luzoni* Wheeler, W. M. and Chapman, 1925: Philippines

*Aenictus maneerati* Jaitrong and Yamane, 2013: Thailand

*Aenictus mariae* Emery, 1895a: South Africa

*Aenictus mariae natalensis* Forel, 1901c: South Africa

*Aenictus mauritanicus* Santschi, 1910c: probably Morocco

*Aenictus mentu* Weber, 1942: South Sudan

*Aenictus minimus* Jaitrong and Hashimoto, 2012: Vietnam

*Aenictus minipetiolus* Jaitrong and Yamane, 2013: Indonesia (Lombok)

*Aenictus minutulus* Terayama and Yamane, 1989: Indonesia (Sumatra)

*Aenictus mocsaryi* Emery, 1901c: Papua New Guinea

*Aenictus moebii* Emery, 1895b: Togo

*Aenictus moebii sankisianus* Forel, 1913a: Democratic Republic of the Congo

*Aenictus montivagus* Jaitrong and Yamane, 2011a: Malaysia (Sabah)

*Aenictus mutatus* Santschi, 1913: Ivory Coast

*Aenictus mutatus pudicus* Santschi, 1919a: Ivory Coast

*Aenictus nesiotis* Wheeler, W. M. and Chapman, 1930b: Philippines

*Aenictus nganduensis* Wilson, 1964: Papua New Guinea

*Aenictus nishimurai* Terayama and Kubota, 1993: Thailand

*Aenictus obscurus* Smith, F., 1865: ‘New Guinea’

*Aenictus orientalis* (Karavaiev, 1926): Indonesia (Aru Is.)

*Aenictus pachycerus* (Smith, F., 1858): India

*Aenictus pangantihoni* Zettel and Sorger, 2010: Philippines

*Aenictus paradentatus* Jaitrong and Yamane, 2012: Thailand

*Aenictus parahuonicus* Jaitrong and Yamane, 2011a: Thailand

*Aenictus peguensis* Emery, 1895c: Myanmar

*Aenictus pfeifferi* Zettel and Sorger, 2010: Malaysia (Sarawak)

*Aenictus pharao* Santschi, 1924: Sudan

*Aenictus philiporum* Wilson, 1964: Australia

*Aenictus philippinensis* Chapman, 1963: Philippines

*Aenictus piercei* Wheeler, W. M. and Chapman, 1930d: Philippines

*Aenictus pilosus* Jaitrong and Yamane, 2013: Philippines

*Aenictus pinkaewi* Jaitrong and Yamane, 2013: Thailand

*Aenictus porizonoides* Walker, 1860: Sri Lanka

*Aenictus powersi* Wheeler, W. M. and Chapman, 1930e: Philippines

*Aenictus prolixus* Shattuck, 2008: Australia

*Aenictus pubescens* Smith, F., 1859: India

*Aenictus punctatus* Jaitrong and Yamane, 2012: Brunei

*Aenictus punctiventris* Emery, 1901b: Indonesia (Laut Island)

*Aenictus punctiventris scutellaris* Forel, 1912d: Indonesia (Sumatra)

*Aenictus punensis* Forel, 1901a: India

*Aenictus rabori* Chapman, 1963: Philippines

*Aenictus raptor* Forel, 1913a: Democratic Republic of the Congo

*Aenictus reyesi* Chapman, 1963: Philippines

*Aenictus rhodiensis* Menozzi, 1936: Greece

*Aenictus rixator* Forel, 1901: South Africa

*Aenictus rotundatus* Mayr, 1901: South Africa

*Aenictus rotundatus guineensis* Santschi, 1924: Guinea

*Aenictus rotundatus merwei* Santschi, 1932: South Africa

*Aenictus rotundicollis* Jaitrong and Yamane, 2011a: Malaysia (Sarawak)

*Aenictus rougieri* André, 1893: Tunisia

*Aenictus sagei* Forel, 1901a: India

*Aenictus schneirlai* Wilson, 1964: Papua New Guinea

*Aenictus seletarius* Wong and Guénard, 2016: Singapore

*Aenictus shillongensis* Mathew and Tiwari, 2000: India

*Aenictus shuckardi* Forel, 1901a: India

*Aenictus siamensis* Jaitrong and Yamane, 2011a: Thailand

*Aenictus silvestrii* Wheeler, W. M., 1929: West Malaysia

*Aenictus sirenicus* Yamane and Wang, 2015: Malaysia (Sabah)

*Aenictus sonchaengi* Jaitrong and Yamane, 2011a: Thailand

*Aenictus soudanicus* Santschi, 1910c: Senegal?

*Aenictus soudanicus brunneus* Forel, 1913a: Democratic Republic of the Congo

*Aenictus spathifer* Santschi, 1928: Indonesia (Sumatra)

*Aenictus steindachneri* Mayr, 1901: South Africa

*Aenictus stenocephalus* Jaitrong, Yamane and Wiwatwitaya, 2010: Thailand

*Aenictus subterraneus* Jaitrong and Hashimoto, 2012: Malaysia (Sabah)

*Aenictus sulawesiensis* Jaitrong and Yamane, 2013: Indonesia (Sulawesi)

*Aenictus sumatrensis* Forel, 1913c: Indonesia (Sumatra)

*Aenictus sumatrensis maxillosus* Forel, 1913c: Indonesia (Sumatra)

*Aenictus sundalandensis* Jaitrong and Yamane, 2013: Indonesia (Java)

*Aenictus thailandianus* Terayama and Kubota, 1993: Thailand

*Aenictus togoensis* Santschi, 1915: Togo

*Aenictus trigonus* Forel, 1911a: Indonesia (Java)

*Aenictus turneri* Forel, 1900a: Australia

*Aenictus vagans* Santschi, 1924: Niger

*Aenictus vaucheri* Emery, 1915c: Morocco

*Aenictus vieti* Jaitrong and Yamane, 2010a: Vietnam

*Aenictus villiersi* Bernard, 1953b: Guinea

*Aenictus watanasiti* Jaitrong and Yamane, 2013: Thailand

*Aenictus wayani* Jaitrong and Yamane, 2011a: Indonesia (Sulawesi)

*Aenictus weissi* Santschi, 1910: Democratic Republic of the Congo

*Aenictus westwoodi* Forel, 1901a: India

*Aenictus wilaiae* Jaitrong and Yamane, 2013: Thailand

*Aenictus wilsoni* Bharti, Wachkoo and Kumar, 2012: India

*Aenictus wiwatwitayai* Jaitrong and Yamane, 2013: Thailand

*Aenictus wroughtonii* Forel, 1890: India

*Aenictus wudangshanensis* Wang, W., 2006: China

*Aenictus yamanei* Wiwatwitaya and Jaitrong, 2011: Malaysia (Sarawak)

*Aenictus yangi* Liu, Hita Garcia, Peng and Economo, 2015: China

*Aenictus zhengi* Zhang, 1995: China

### Cerapachys

Taxon classificationAnimaliaHymenopteraFormicidae

Smith, F., 1857

= Ceratopachys Schulz, 1906

#### Type-species.

*Cerapachys antennatus*, by subsequent designation of Bingham, 1903

This relatively species-poor lineage is apparently restricted to forest habitats of Southeast Asia.

#### Diagnosis.

***Worker.*** *Cerapachys* belongs to non-army ant dorylines with spiracle positioned below midheight of the propodeum and pygidium well-developed, armed with modified setae. It has a well-developed carina on the pronotal collar and a distinct pronotomesopleural Pronotomesopleural suture, a single pectinate spur on each mid and hind tibia, and helcium positioned supraaxially, above midheight of abdominal segment III. Some species have pretarsal claws armed with a tooth. *Cerapachys* is a genus of medium-sized, universally dark-colored ants that could be confused *Lividopone*. Distributions of the two genera do not overlap, however, with *Lividopone* being so far known only from Madagascar. *Lividopone* is further distinguished by almost complete fusion of pronotomesopleural Pronotomesopleural suture, which is unfused in *Cerapachys*. *Lioponera* overlaps in range with *Cerapachys* and certain species can be superficially similar but a more narrow and axially positioned helcium, dorsolaterally carinate petiole, and a flange on the posterior face of the coxae will distinguish *Lioponera*.

***Male.*** The male of *Cerapachys* has 12-segmented antennae, a transverse groove running diagonally across the mesopleuron, vein C in fore wing present, one submarginal cell closed by Rs·f2–3, 2rs-m absent, and marginal cell closed by R·f3 and Rs·f4–5. *Neocerapachys* males have similar wing venation but in *Cerapachys* the antennal segment III is similar in length to segment IV, while in *Neocerapachys* the segment III is conspicuously the shortest antennal segment. Furthermore, *Neocerapachys* is only found in the New World.

#### Description.

***Worker.*** *Head*: Antennae with 12 segments. Apical antennal segment conspicuously enlarged, much broader than and longer than two preceding segments combined. Clypeus with or without cuticular apron. Lateroclypeal teeth absent. Parafrontal ridges reduced. Torulo-posttorular complex vertical. Antennal scrobes absent. Labrum with median notch or concavity. Proximal face of stipes projecting beyond inner margin of sclerite, concealing prementum when mouthparts fully closed. Maxillary palps 3-segmented. Labial palps 2-segmented. Mandibles triangular, with teeth. Eyes present, composed of more than 20 ommatidia. Ocelli absent. Head capsule with differentiated vertical posterior surface above occipital foramen. Ventrolateral margins of head without lamella or ridge extending towards mandibles and beyond carina surrounding occipital foramen. Posterior head corners dorsolaterally immarginate. Carina surrounding occipital foramen ventrally absent. *Mesosoma*: Pronotal flange separated from collar by distinct ridge. Promesonotal connection with Pronotomesopleural suture completely fused. Pronotomesopleural suture visible, unfused partway to notal surface. Mesometapleural groove deeply impressed, conspicuous. Transverse groove dividing mesopleuron absent. Transverse groove dividing mesopleuron present. Pleural endophragmal pit concavity present. Mesosoma dorsolaterally immarginate. Metanotal depression or groove on mesosoma present. Propodeal spiracle situated low on sclerite. Propodeal declivity with distinct dorsal edge or margin and rectangular in posterior view. Metapleural gland without bulla visible through cuticle. Propodeal lobes present, well developed. *Metasoma*: Petiole anterodorsally marginate, dorsolaterally immarginate, and laterally above spiracle marginate. Helcium in relation to tergosternal Pronotomesopleural suture placed at posttergite and supraaxial. Prora forming a V-shaped protrusion. Spiracle openings of abdominal segments IV–VI circular. Abdominal segment III anterodorsally marginate, dorsolaterally immarginate. Abdominal segment III more than half size of succeeding segment IV, which is weakly constricted at presegmental portion (uninodal waist). Girdling constriction of segment IV present, i.e. pre- and postsclerites distinct. Cinctus of abdominal segment IV gutter-like and cross-ribbed. Abdominal segment IV not conspicuously largest segment. Abdominal tergite IV not folding over sternite, and anterior portions of sternite and tergite equally well visible in lateral view. Girdling constriction between pre- and posttergites of abdominal segments V and VI absent. Girdling constriction between pre- and poststernites of abdominal segments V and VI absent. Pygidium large, with impressed medial field, and armed with modified setae. Hypopygium unarmed. *Legs*: Mid tibia with single pectinate spur. Hind tibia with single pectinate spur. Hind basitarsus not widening distally, circular in cross-section. Posterior flange of hind coxa not produced as raised lamella. Metatibial gland present as oval patch of whitish cuticle. Metabasitarsal gland absent. Hind pretarsal claws simple or each claw armed with a tooth. *Polymorphism*: Monomorphic to moderately polymorphic.

***Male.*** *Head*: Antennae with 13 segments. Clypeus with cuticular apron. Parafrontal ridges present. Torulo-posttorular complex vertical. Maxillary palps 5-segmented. Labial palps likely 3-segmented (uncertain in-situ count). Mandibles triangular, edentate or crenulate. Ventrolateral margins of head with cuticular ridge extending towards mandibles and beyond carina surrounding occipital foramen. Carina surrounding occipital foramen ventrally present. *Mesosoma*: Pronotal flange separated from collar by distinct ridge or not separated. Notauli present. Transverse groove dividing mesopleuron present. Propodeal declivity reduced, with or without distinct dorsal edge or margin. Metapleural gland opening absent. Propodeal lobes present. *Metasoma*: Petiole anterodorsally immarginate, dorsolaterally immarginate or marginate, and laterally above spiracle marginate. Helcium in relation to tergosternal Pronotomesopleural suture placed at posttergite and supraaxial. Prora forming a V-shaped protrusion. Spiracle openings of abdominal segments IV–VI circular. Abdominal segment III more than half size of succeeding segment IV, occasionally slightly smaller; latter weakly constricted at presegmental portion (uninodal waist). Girdling constriction of segment IV present, i.e. pre- and postsclerites distinct. Cinctus of abdominal segment IV gutter-like, not sculptured or cross-ribbed. Girdling constriction between pre- and postsclerites of abdominal segments V and VI absent. Abdominal segment IV not conspicuously largest segment. Abdominal sternite VII simple. Abdominal sternite IX distally armed with two spines. with lateral apodemes about as long as medial apodeme, directed anteriorly (towards head). *Genitalia*: Cupula long relative to rest of genital capsule and shorter ventrally than dorsally. Basimere broadly fused to telomere, with sulcus discernable at junction, and ventrally with left and right arms abutting. Telomere gradually tapering toward apex. Volsella gradually tapering toward apex. Penisvalva laterally compressed, rounded at apex. *Legs*: Mid tibia with single pectinate spur. Hind tibia with single pectinate spur. Posterior flange of hind coxa not produced as raised lamella. Metatibial gland present as oval patch of whitish cuticle. Metabasitarsal glands absent. Hind pretarsal claws each armed with a tooth; simple claws not observed but presumably absent from certain species as in worker. *Wings*: Tegula present, broad, demiovate in shape. Vein C in fore wing present. Pterostigma broad. Abscissa R·f3 present, running toward distal wing margin and enclosing cell with Rs·f5. Abscissae Rs·f2–3 present, connecting with Rs+M&M·f2. Cross-vein 2r-rs present, differentiated from Rs·f4 by presence of Rs·f2–3. Abscissae Rs·f4–5 present, fused in absence of 2rs-m. Abscissa M·f2 in fore wing contiguous with Rs+M or separated from Rs+M by Rs·f2. Abscissa M·f4 in fore wing present, not reaching wing margin to almost reaching wing margin. Cross-vein 1m-cu in fore wing present. Cross-vein cu-a in fore wing present, arising from M+Cu and proximal to M·f1. Vein Cu in fore wing present, with both branches Cu1 and Cu2. Vein A in fore wing with abscissae A·f1 and A·f2 present. Vein C in hind wing absent. Vein R in hind wing absent. Vein Sc+R in hind wing present. Abscissa Rs·f1 in hind wing present, shorter than 1rs-m. Abscissa Rs·f2 in hind wing present, not reaching wing margin. Cross-vein 1rs-m in hind wing fused with M·f1. Vein M+Cu in hind wing present. Abscissa M·f1 in hind wing present. Abscissa M·f2 in hind wing present. Cross-vein cu-a in hind wing present. Vein Cu in hind wing absent. Vein A in hind wing with abscissae A·f1 and A·f2 present.

***Gyne.*** Alate, fully winged with large eyes and three ocelli, or also possibly ergatoid (Brown 1975), larger than worker, with large eyes and three ocelli but no wings.

***Larva.*** Not described. Cocoons absent.

#### Distribution.

*Cerapachys* is distributed from northwestern India and Tibet, through southern China to Java, Borneo and the Philippines.

#### Taxonomy and phylogeny.

This is the lineage where the type species of *Cerapachys*, *Cerapachys antennatus*, belongs. The type series was collected by A. R. Wallace in Sarawak and described by F. Smith in 1857.

*Cerapachys* is a member of a predominantly South East Asian clade that also includes *Chrysapace* and *Yunodorylus* (Borowiec, in prep.). All three lineages diverged long ago and although it seems that *Cerapachys* is the sister genus to *Chrysapace*, this relationship is not certain.

#### Biology.

Almost nothing is known about the biology of this group. Most records seem to come from forest habitats. Brood development may be synchronized, based on the author’s observation of brood of uniform size in the single examined nest collection of *Cerapachys antennatus*.

#### Species of *Cerapachys*

*Cerapachys antennatus* Smith, F., 1857: Malaysia (Sarawak)

*Cerapachys jacobsoni* Forel, 1912b: Indonesia (Java)

*Cerapachys manni* Crawley, 1926: Indonesia (Sumatra)

*Cerapachys sulcinodis* Emery, 1889c: Myanmar

*Cerapachys xizangensis* Tang and Li, 1982: Tibet

### Cheliomyrmex

Taxon classificationAnimaliaHymenopteraFormicidae

Mayr, 1870

#### Type-species.

*Cheliomyrmex nortoni* (junior synonym of *Labidus morosus*), by monotypy.

*Cheliomyrmex* is a rarely encountered genus of New World army ants that is a mostly subterranean predator with likely a specialized diet.

#### Diagnosis.

***Worker.*** Workers of *Cheliomyrmex* can be recognized by a combination of propodeal spiracle positioned high on the propodeum, propodeal declivity simple and not armed with cuticular ridges or denticles, abdominal segment III small but broad posteriorly and thus waist appearing one-segmented, pygidium small and armed with at most a pair of modified setae, and pretarsal claws armed with a tooth. *Cheliomyrmex* is perhaps most similar to *Labidus* and certain *Neivamyrmex* but it is unique among New World army ants in having abdominal segment III broadly attached to segment IV (i.e. it has a uninodal waist) and thus easily told apart from all other army ant genera in this region.

***Male.*** The males of *Cheliomyrmex* share the following wing venation characters with other New World army ants (*Eciton*, *Labidus*, *Neivamyrmex* and *Nomamyrmex*): costal (C) vein present in the fore wing, relatively narrow pterostigma, presence of vein 2rs-m and two closed submarginal cells, marginal cell closed by R·f3 and Rs·f4–5, 2rs-m present, and M·f1 vein arising from M+Cu at an angle lower than 45° and conspicuously proximal to cu-a. This characteristic venation pattern serves to distinguish New World army ants from the Old World army ants (*Aenictogiton*, *Aenictus*, *Dorylus*) that have no vein R·f3 and where M·f1 arises near cu-a and at an angle close to or higher than 45°. *Aenictus* and *Dorylus* additionally have no vein Rs·f2–3 and so only one submarginal cell that is closed distally by 2rs-m. In other dorylines with well-developed wing venation (e.g. *Chrysapace*, *Cylindromyrmex*) the vein M·f1 arises distal to cu-a and pterostigma is very broad and conspicuous. Within the New World army ants, wing venation is relatively conserved and thus of little use in discrimination of genera. Genitalic characters have been found to be the most reliable (Watkins 1976), although impossible to ascertain without dissection. A combination of absence of very long setae approaching femur length on the abdomen, apices of penisvalvae with setae, and the sternite of abdominal segment IX (subgenital plate) with four teeth, and a simple hind basitarsus will distinguish *Cheliomyrmex* males from all other army ant genera in the New World. The long setae on gaster are characteristic of *Nomamyrmex*. The penisvalvae with setae are also present in *Labidus* but the latter can be told apart by having only two teeth on the abdominal sternite IX and a complex hind basal tarsal segment, which has a conspicuous oblique groove that accommodates the hind tibial spur.

#### Description.

***Worker.*** *Head*: Antennae with 12 segments. Apical antennal segment not enlarged, not broader and longer than two preceding segments combined. Clypeus without cuticular apron. Lateroclypeal teeth absent. Parafrontal ridges reduced. Torulo-posttorular complex vertical. Antennal scrobes absent. Labrum with median notch or concavity. Proximal face of stipes not projecting beyond inner margin of sclerite, prementum exposed when mouthparts fully closed. Maxillary palps 2-segmented. Labial palps 3-segmented. Mandibles polymorphic, from triangular with teeth through triangular with median tooth to falcate, with teeth on elongated masticatory margin. Eyes present, composed of 1–5 ommatidia. Ocelli absent. Head capsule with differentiated vertical posterior surface above occipital foramen. Ventrolateral margins of head without lamella or ridge extending towards mandibles and beyond carina surrounding occipital foramen. Posterior head corners dorsolaterally immarginate. Carina surrounding occipital foramen ventrally absent. *Mesosoma*: Pronotal flange not separated from collar by distinct ridge. Promesonotal connection with Pronotomesopleural suture completely fused. Pronotomesopleural suture visible, unfused partway to notal surface. Mesometapleural groove weakly impressed. Transverse groove dividing mesopleuron present. Pleural endophragmal pit concavity present. Mesosoma dorsolaterally immarginate. Metanotal depression or groove on mesosoma present. Propodeal spiracle situated high on sclerite. Propodeal declivity without distinct dorsal edge or margin and rectangular in posterior view. Metapleural gland with bulla visible through cuticle. Propodeal lobes absent. *Metasoma*: Petiole anterodorsally immarginate, dorsolaterally immarginate, and laterally above spiracle marginate. Helcium in relation to tergosternal Pronotomesopleural suture placed at Pronotomesopleural suture and infraaxial. Prora narrowed into anteriorly directed spine. Spiracle openings of abdominal segments IV–VI oval. Abdominal segment III anterodorsally immarginate and dorsolaterally immarginate. Abdominal segment III more than half size of succeeding segment IV, which is weakly constricted at presegmental portion (uninodal waist). Girdling constriction of segment IV present, i.e. pre- and postsclerites distinct. Cinctus of abdominal segment IV gutter-like, not sculptured. Abdominal segment IV not conspicuously largest segment. Abdominal tergite IV not folding over sternite, and anterior portions of sternite and tergite equally well visible in lateral view. Girdling constriction between pre- and posttergites of abdominal segments V and VI absent. Girdling constriction between pre- and poststernites of abdominal segments V and VI absent. Pygidium small, reduced to narrow strip, without impressed medial field, and simple, not armed with cuticular spines or modified setae. Hypopygium unarmed. *Legs*: Mid tibia with single pectinate spur. Hind tibia with single pectinate spur. Hind basitarsus not widening distally, circular in cross-section. Posterior flange of hind coxa not produced as raised lamella. Metatibial gland present as oval patch of whitish cuticle. Metabasitarsal gland absent. Hind pretarsal claws each armed with a tooth. *Polymorphism*: Polymorphic.

***Male.*** *Head*: Antennae with 13 segments. Clypeus without cuticular apron. Parafrontal ridges absent. Torulo-posttorular complex vertical. Maxillary palps 2-segmented. Labial palps 3-segmented. Mandibles falcate. Ventrolateral margins of head without lamella or ridge extending towards mandibles and beyond carina surrounding occipital foramen. Carina surrounding occipital foramen ventrally absent. *Mesosoma*: Pronotal flange not separated from collar by distinct ridge. Notauli absent. Transverse groove dividing mesopleuron absent. Propodeal declivity reduced, without distinct dorsal edge or margin. Metapleural gland opening absent. Propodeal lobes absent. *Metasoma*: Petiole anterodorsally immarginate, dorsolaterally immarginate, and laterally above spiracle immarginate. Helcium in relation to tergosternal Pronotomesopleural suture placed at Pronotomesopleural suture and axial. Prora simple, not delimited by carina. Spiracle openings of abdominal segments IV–VI slit-shaped. Abdominal segment III more than half size of succeeding segment IV; latter weakly constricted at presegmental portion (uninodal waist). Girdling constriction of segment IV absent, i.e. pre- and postsclerites indistinct. Cinctus of abdominal segment IV absent, not impressed. Girdling constriction between pre- and postsclerites of abdominal segments V and VI absent. Abdominal segment IV not conspicuously largest segment. Abdominal sternite VII simple. Abdominal sternite IX distally armed with two outer spines and additional two inner denticles, with lateral apodemes longer than much reduced medial apodeme, directed anteriorly (towards head). *Genitalia*: Cupula very long, nearing or surpassing length of rest of genital capsule and shorter ventrally than dorsally. Basimere narrowly fused to telomere, with sulcus visible at least partway through junction, and ventrally with left and right arms abutting. Telomere expanded at apex. Volsella narrow, hook-shaped. Penisvalva not flattened at apex, expanded. *Legs*: Mid tibia with single pectinate spur. Hind tibia with single pectinate spur. Posterior flange of hind coxa not produced as raised lamella. Metatibial gland absent. Metabasitarsal glands absent. Hind pretarsal claws each armed with a tooth. *Wings*: Tegula present, broad, demiovate in shape. Vein C in fore wing present. Pterostigma narrow. Abscissa R·f3 present, running toward distal wing margin and enclosing cell with Rs·f5. Abscissae Rs·f2–3 present, connecting with Rs+M&M·f2. Cross-vein 2r-rs present, differentiated from Rs·f4 by presence of Rs·f2–3. Abscissae Rs·f4–5 differentiated into Rs·f4 and Rs·f5 by 2rs-m. Abscissa M·f2 in fore wing present, separated from Rs+M by Rs·f2. Abscissa M·f4 in fore wing present, reaching wing margin. Cross-vein 1m-cu in fore wing present. Cross-vein cu-a in fore wing present, arising from Cu and distal to, at or near M·f1. Vein Cu in fore wing present, with both branches Cu1 and Cu2. Vein A in fore wing with abscissae A·f1 and A·f2 present. Vein C in hind wing present. Vein R in hind wing present, extending past Sc+R but not reaching distal wing margin. Vein Sc+R in hind wing present. Abscissa Rs·f1 in hind wing present, shorter than 1rs-m. Abscissa Rs·f2 in hind wing present, reaching wing margin. Cross-vein 1rs-m in hind wing present, about as long as M·f1. Vein M+Cu in hind wing present. Abscissa M·f1 in hind wing present. Abscissa M·f2 in hind wing present. Cross-vein cu-a in hind wing present. Vein Cu in hind wing present. Vein A in hind wing with abscissae A·f1 and A·f2 present.

***Gyne.*** Not described.

***Larva.*** Larvae of *Cheliomyrmex megalonyx* have been described (Wheeler 1943, Wheeler and Wheeler 1984). Presence of cocoons unknown.

#### Distribution.

*Cheliomyrmex* is present in most of Central America, including southern Mexico, Belize, Guatemala, Honduras, and Panama, but so far it has not been collected in Nicaragua or Costa Rica. It is also known from northern and northwestern South America south to Peru and Bolivia.

#### Taxonomy and phylogeny.

*Cheliomyrmex* was introduced by Mayr in 1870 who described *Cheliomyrmex nortoni*, now a junior synonym of *Cheliomyrmex morosus* (Smith 1859) and recognized its affinity to other dorylines. The genus-level taxonomy of *Cheliomyrmex* has been relatively stable and there are four currently recognized species. Because of its morphology, notably the fact that *Cheliomyrmex* are the only New World army ants that possess only a single waist segment, the genus has been often considered of particular importance to army ant systematics (Wheeler 1921, Gotwald 1971, Gotwald and Kupiec 1975, Gotwald 1979). However, the current understanding of doryline phylogeny shows *Cheliomyrmex* nested within the New World army ants (Brady et al. 2014), sister to the (*Labidus* (*Eciton* plus *Nomamyrmex*)) clade.

#### Biology.

Ants in this lineage have been rarely observed or collected. The raids and emigrations of these ants are mostly subterranean, only occasionally seen above ground. Raids have been observed mostly under stones or rotting wood (Wheeler 1921, Gotwald 1971). A diverse fauna of associates was reported from an emigration column of *Cheliomyrmex morosus*, including phorid flies, staphylinid beetles, silverfish and mites (Berghoff and Franks 2007). The 2007 study and the only other published observation of a *Cheliomyrmex* emigration (*Cheliomyrmex megalonyx*; Wheeler 1921), described galleries of soil built by the ants to cover the areas where the ant columns had to proceed on the surface. Wheeler also reported a behavior where stationary major workers were guarding the emigration columns and compared it to that of African *Dorylus*, although this behavior is also known in *Labidus* (Rettenmeyer 1963). As Wheeler observed only larvae being carried by the workers, it has been postulated that brood production is synchronized (Rettenmeyer 1963). *Cheliomyrmex andicola* has been observed feeding on a dead snake and actively pursuing and killing a giant earthworm in Ecuador (O’Donnell et al. 2005). Given that no other prey has been observed for this genus, combined with the specialized mandibular morphology and potent sting, O’Donnell et al. (2005) proposed that *Cheliomyrmex* are specialized predators of large subterranean invertebrates or maybe even vertebrates.

#### Species of *Cheliomyrmex*

*Cheliomyrmex andicola* Emery, 1894: Peru

*Cheliomyrmex audax* Santschi, 1921b: Ecuador

*Cheliomyrmex megalonyx* Wheeler, W. M., 1921: Guyana

*Cheliomyrmex morosus* (Smith, 1859): Mexico

### Chrysapace

Taxon classificationAnimaliaHymenopteraFormicidae

Crawley, 1924a gen. rev.

#### Type-species.

*Chrysapace jacobsoni*, by monotypy.

*Chrysapace* is the only extant doryline genus also known from Baltic amber (late Eocene). These ants are extremely rarely collected and no observations of their biology have ever been published.

#### Diagnosis.

***Worker.*** The workers of this lineage are recognizable by a combination of prominent costate sculpture present on most of body surface, large eyes, exposed antennal sockets, two spurs on mid and hind tibiae, and pretarsal claws with a tooth. The New World *Cylindromyrmex* are the only other dorylines that have two pectinate tibial spurs and strongly costate or rugose sculpture but they are recognized by at least moderately developed antennal scrobes and horizontal torulo-posttorular complex that partly conceals antennal sockets. In *Chrysapace* there are no scrobes and antennal sockets are fully exposed.

***Male.*** The males share the characteristic spur formula with the workers, have a well-defined groove on the mesopleuron, two submarginal cells, the marginal cell enclosed by R·f1 and Rs·f4–5, and pretarsal claws armed with a tooth. *Acanthostichus* and *Cylindromyrmex* can have similar wing venation. The former has only one pectinate tibial spur on each mid and hind tibiae and the latter has no transverse groove on the mesopleuron and simple pretarsal claws. Males attributed to *Procerapachys* also have similar wing venation but only a single tibial spur and no transverse groove on the mesopleuron.

#### Description.

***Worker.*** *Head*: Antennae with 12 segments. Apical antennal segment moderately enlarged, broader than and about equal in length to two preceding segments combined. Clypeus without cuticular apron. Lateroclypeal teeth absent. Parafrontal ridges reduced. Torulo-posttorular complex vertical. Antennal scrobes absent. Labrum with median notch or concavity. Proximal face of stipes projecting beyond inner margin of sclerite, concealing prementum when mouthparts fully closed. Maxillary palps 5-segmented. Labial palps 3-segmented. Mandibles triangular, with teeth. Eyes present, composed of more than 20 ommatidia. Ocelli present. Head capsule with differentiated vertical posterior surface above occipital foramen. Ventrolateral margins of head without lamella or ridge extending towards mandibles and beyond carina surrounding occipital foramen. Posterior head corners dorsolaterally immarginate or marginate. Carina surrounding occipital foramen ventrally present. *Mesosoma*: Pronotal flange separated from collar by distinct ridge. Promesonotal connection with Pronotomesopleural suture completely fused. Pronotomesopleural suture visible, unfused up to notal surface. Mesometapleural groove replaced by cuticular ridge. Transverse groove dividing mesopleuron absent. Pleural endophragmal pit concavity absent. Mesosoma dorsolaterally immarginate. Metanotal depression or groove on mesosoma absent. Propodeal spiracle situated low on sclerite. Propodeal declivity with or without distinct dorsal edge or margin and rectangular in posterior view. Metapleural gland without bulla visible through cuticle. Propodeal lobes present, well developed. *Metasoma*: Petiole anterodorsally marginate, dorsolaterally immarginate, and laterally above spiracle immarginate. Helcium in relation to tergosternal Pronotomesopleural suture placed at posttergite and axial. Prora forming a V-shaped protrusion. Spiracle openings of abdominal segments IV–VI circular. Abdominal segment III anterodorsally marginate, dorsolaterally immarginate. Abdominal segment III more than half size of succeeding segment IV, which is weakly constricted at presegmental portion (uninodal waist). Girdling constriction of segment IV present, i.e. pre- and postsclerites distinct. Cinctus of abdominal segment IV gutter-like and sculptured, cross-ribbed or not. Abdominal segment IV not conspicuously largest segment. Abdominal tergite IV not folding over sternite, and anterior portions of sternite and tergite equally well visible in lateral view. Girdling constriction between pre- and posttergites of abdominal segments V and VI absent. Girdling constriction between pre- and poststernites of abdominal segments V and VI absent. Pygidium large, with impressed medial field and armed with modified setae and sometimes emarginate to deeply notched. Hypopygium unarmed. *Legs*: Mid tibia with two pectinate spurs. Hind tibia with two pectinate spurs or with one barbulate and one pectinate spur. Hind basitarsus not widening distally, circular in cross-section. Posterior flange of hind coxa not produced as raised lamella. Metatibial gland absent. Metabasitarsal gland absent. Hind pretarsal claws each armed with a tooth, sometimes very small. *Polymorphism*: Monomorphic.

***Male.*** *Head*: Antennae with 13 segments. Clypeus without cuticular apron. Parafrontal ridges present. Torulo-posttorular complex vertical. Maxillary palps unknown. Labial palps unknown. Mandibles triangular, edentate. Ventrolateral margins of head without lamella or ridge extending towards mandibles and beyond carina surrounding occipital foramen. Carina surrounding occipital foramen unknown. *Mesosoma*: Pronotal flange separated from collar by distinct ridge. Notauli present. Transverse groove dividing mesopleuron present. Propodeal declivity with distinct dorsal edge or margin. Metapleural gland opening present. Propodeal lobes present. *Metasoma*: Petiole anterodorsally marginate, dorsolaterally immarginate, and laterally above spiracle marginate. Helcium in relation to tergosternal Pronotomesopleural suture placed at posttergite and axial. Prora forming a V-shaped protrusion. Spiracle openings of abdominal segments IV–VI circular. Abdominal segment III more than half size of succeeding segment IV; latter weakly constricted at presegmental portion (uninodal waist). Girdling constriction of segment IV present, i.e. pre- and postsclerites distinct. Cinctus of abdominal segment IV gutter-like and cross-ribbed. Girdling constriction between pre- and postsclerites of abdominal segments V and VI absent. Abdominal segment IV conspicuously largest segment. Abdominal sternite VII simple. Abdominal sternite IX distally armed with two spines, with lateral apodemes about as long as medial apodeme, directed anteriorly (towards head). *Genitalia*: Cupula long relative to rest of genital capsule and shorter ventrally than dorsally. Basimere broadly fused to telomere, with sulcus discernable at junction, and ventrally with left and right arms separated. Telomere gradually tapering toward apex. Volsella gradually tapering toward apex. Penisvalva laterally compressed, rounded at apex. *Legs*: Mid tibia with two pectinate spurs. Hind tibia with two pectinate spurs. Posterior flange of hind coxa not produced as raised lamella. Metatibial gland absent. Metabasitarsal glands absent. Hind pretarsal claws each armed with a tooth. *Wings*: Tegula present, broad, demiovate in shape. Vein C in fore wing present. Pterostigma broad. Abscissa R·f3 present, running toward distal wing margin and enclosing cell with Rs·f5. Abscissae Rs·f2–3 present, connecting with Rs+M&M·f2. Cross-vein 2r-rs present, differentiated from Rs·f4 by presence of Rs·f2–3. Abscissae Rs·f4–5 differentiated into Rs·f4 and Rs·f5 by 2rs-m. Abscissa M·f2 in fore wing present, separated from Rs+M by Rs·f2. Abscissa M·f4 in fore wing present, reaching wing margin. Cross-vein 1m-cu in fore wing present. Cross-vein cu-a in fore wing present, arising from M+Cu and proximal to M·f1. Vein Cu in fore wing present, with both branches Cu1 and Cu2. Vein A in fore wing with abscissae A·f1 and A·f2 present. Vein C in hind wing absent. Vein R in hind wing absent. Vein Sc+R in hind wing present. Abscissa Rs·f1 in hind wing present, shorter than 1rs-m. Abscissa Rs·f2 in hind wing present, not reaching wing margin. Cross-vein 1rs-m in hind wing fused with M·f1. Vein M+Cu in hind wing present. Abscissa M·f1 in hind wing present. Abscissa M·f2 in hind wing present. Cross-vein cu-a in hind wing present. Vein Cu in hind wing present. Vein A in hind wing with abscissae A·f1 and A·f2 present.

***Gyne.*** Alate, similar to worker except for flight-adapted mesosoma. See Terayama et al. (1988) for a description of *Cerapachys sauteri* gyne.

***Larva.*** Not described. Presence of cocoons unknown.

#### Distribution.

This rarely collected lineage is represented by at least four extant species of unusual geographic distribution. *Chrysapace costatus*, *Chrysapace crawleyi* and *Cerapachys sauteri* occur in Asia, while an additional, undescribed species has been recently found in northern Madagascar (Brian Fisher pers. comm.). One species is known from Baltic amber.

#### Taxonomy and phylogeny.

The genus *Chrysapace* was proposed by Crawley in 1924 for *Cerapachys jacobsoni* from Sumatra as distinct from the then recognized *Cerapachys* and *Phyracaces*. The same year W. M. Wheeler (1924b) published a note where he pointed out that sometime in the future a synonymization of *Chrysapace* and *Cerapachys* seems likely, and that this synonymy would render *Cerapachys jacobsoni* Crawley a junior homonym of *Cerapachys jacobsoni* Forel, 1912. Wheeler thus proposed a replacement name *Chrysapace crawleyi*, which was accepted by Brown after he synonymized the genus under *Cerapachys* in 1975. *Cerapachys sauteri* Forel from Taiwan was recognized as a relative by Brown based on the original description, and later Terayama et al. (1988) provided a detailed redescription of this species, confirming its affinity with *Chrysapace crawleyi*. Until recently these two species from Southeast Asia were the only taxa known in this lineage, but the discovery of an undescribed species in Madagascar and the description of *Cerapachys costatus* from northwest India has significantly broadened the lineage’s known distribution. An additional species of *Chrysapace* has been recently discovered in Baltic amber (author’s unpublished observation).

*Chrysapace* is a member of a well-supported clade that also includes *Cerapachys* and *Yunodorylus*, and is possibly the sister genus of *Cerapachys* although this relationship received less support (Borowiec, in prep.).

#### Biology.

To the best of my knowledge, nothing on the foraging, nesting, or other aspects of *Chrysapace* biology has ever been published.

#### Species of *Chrysapace*

*Chrysapace costatus* (Bharti and Wachkoo, 2013): India, **comb. n.**

*Chrysapace jacobsoni* Crawley, 1924: Indonesia (Sumatra), **nom. rev.**

*Chrysapace sauteri* (Forel, 1913b): Taiwan, **comb. n.**

### Cylindromyrmex

Taxon classificationAnimaliaHymenopteraFormicidae

Mayr, 1870

= Holcoponera Cameron, 1891
= Hypocylindromyrmex Wheeler, W. M., 1924a
= Metacylindromyrmex Wheeler, W. M., 1924a

#### Type-species.

*Cylindromyrmex striatus*, by monotypy.

*Cylindromyrmex* is a genus of mostly arboreal-nesting termite hunters, rarely encountered but distributed throughout New World tropics, including the Antilles.

#### Diagnosis.

***Worker.*** With a combination of large eyes, conspicuously costate or striate sculpture, torulo-posttorular complex horizontal and concealing antennal sockets, two pectinate spurs on mid and hind tibiae, and simple pretarsal claws, the workers of *Cylindromyrmex* can be readily distinguished from all other dorylines. The only other genus with large eyes, conspicuously sulcate sculpture, and two tibial spurs is *Chrysapace*, but it has fully exposed antennal sockets, possesses toothed pretarsal claws, and occurs only in the Old World. The extinct *Procerapachys*, which can also have sulcate sculpturing, has a single pectinate spur on each mid and hind tibiae.

***Male.*** The males of *Cylindromyrmex* are also easily differentiated from all other genera by two tibial spurs, simple pretarsal claws, no transverse groove on the mesopleuron, and well-developed wing venation with costal (C) vein present in fore wing, two submarginal cells and marginal cell closed. The only other genus with two tibial spurs and similar venation is the Old World genus *Chrysapace*, but it has a transverse groove on the mesopleuron and pretarsal claws armed with a tooth. Putative males of the extinct *Procerapachys* have only one spur on each mid and hind tibiae.

#### Description.

***Worker.*** *Head*: Antennae with 12 segments. Apical antennal segment moderately enlarged, broader than and about equal in length to two preceding segments combined. Clypeus without cuticular apron. Lateroclypeal teeth absent. Parafrontal ridges absent. Torulo-posttorular complex horizontal. Antennal scrobes present. Labrum with median notch or concavity. Proximal face of stipes projecting beyond inner margin of sclerite, concealing prementum when mouthparts fully closed. Maxillary palps 3- or 2-segmented. Labial palps 2-segmented. Mandibles triangular, with teeth or edentate. Eyes present, always composed of more than 5 ommatidia and usually more than 20 ommatidia. Ocelli present or absent. Head capsule with differentiated vertical posterior surface above occipital foramen. Ventrolateral margins of head without lamella or ridge extending towards mandibles and beyond carina surrounding occipital foramen. Posterior head corners dorsolaterally immarginate. Carina surrounding occipital foramen ventrally present. *Mesosoma*: Pronotal flange separated from collar by distinct ridge or not. Promesonotal connection with Pronotomesopleural suture completely fused. Pronotomesopleural suture visible, unfused up to notal surface. Mesometapleural groove deeply impressed, conspicuous. Transverse groove dividing mesopleuron absent. Pleural endophragmal pit concavity present. Mesosoma dorsolaterally immarginate. Metanotal depression or groove on mesosoma absent. Propodeal spiracle situated low on sclerite. Propodeal declivity with distinct dorsal edge or margin and rectangular in posterior view. Metapleural gland bulla visible or not visible through cuticle. Propodeal lobes present, well developed. *Metasoma*: Petiole anterodorsally marginate, dorsolaterally immarginate, dorsolaterally marginate, and laterally above spiracle marginate. Helcium in relation to tergosternal Pronotomesopleural suture placed at posttergite and supraaxial. Prora forming a V-shaped protrusion. Spiracle openings of abdominal segments IV–VI circular. Abdominal segment III anterodorsally immarginate and dorsolaterally immarginate. Abdominal segment III more than half size of succeeding segment IV, which is weakly constricted at presegmental portion (uninodal waist). Girdling constriction of segment IV present, i.e. pre- and postsclerites distinct. Cinctus of abdominal segment IV gutter-like, not sculptured. Abdominal segment IV not conspicuously largest segment. Abdominal tergite IV not folding over sternite, and anterior portions of sternite and tergite equally well visible in lateral view. Girdling constriction between pre- and posttergites of abdominal segments V and VI absent. Girdling constriction between pre- and poststernites of abdominal segments V and VI present or absent. Pygidium large, with impressed medial field and armed with modified setae. Hypopygium unarmed. *Legs*: Mid tibia with two pectinate spurs. Hind tibia with two pectinate spurs. Hind basitarsus not widening distally, circular in cross-section. Posterior flange of hind coxa not produced as raised lamella. Metatibial gland present as oval patch of whitish cuticle. Metabasitarsal gland absent. Hind pretarsal claws simple. *Polymorphism*: Monomorphic.

***Male.*** *Head*: Antennae with 13 segments. Clypeus without cuticular apron. Parafrontal ridges absent. Torulo-posttorular complex vertical. Maxillary palps 2-segmented. Labial palps 3- or 2-segmented. Mandibles triangular, edentate. Ventrolateral margins of head without lamella or ridge extending towards mandibles and beyond carina surrounding occipital foramen. Carina surrounding occipital foramen ventrally present. *Mesosoma*: Pronotal flange separated from collar by distinct ridge or not separated. Notauli absent or present. Transverse groove dividing mesopleuron absent. Propodeal declivity with distinct dorsal edge or margin. Metapleural gland opening present. Propodeal lobes present. *Metasoma*: Petiole anterodorsally marginate, dorsolaterally immarginate, and laterally above spiracle marginate. Helcium in relation to tergosternal Pronotomesopleural suture placed at posttergite and supraaxial. Prora forming a U-shaped margin with median ridge. Spiracle openings of abdominal segments IV–VI circular. Abdominal segment III more than half size of succeeding segment IV; latter weakly constricted at presegmental portion (uninodal waist). Girdling constriction of segment IV present, i.e. pre- and postsclerites distinct. Cinctus of abdominal segment IV gutter-like, not sculptured or cross-ribbed. Girdling constriction between pre- and postsclerites of abdominal segments V and VI absent. Abdominal segment IV not conspicuously largest segment. Abdominal sternite VII simple. Abdominal sternite IX distally armed with two spines, with lateral apodemes about as long as medial apodeme, directed anteriorly (towards head). *Genitalia*: Cupula long relative to rest of genital capsule and shorter ventrally than dorsally. Basimere broadly fused to telomere, with sulcus discernable at junction, and ventrally with left and right arms abutting. Telomere gradually tapering toward apex. Volsella laterally flattened, at apex with dorsal lobe and hooked ventrally. Penisvalva laterally compressed, rounded at apex. *Legs*: Mid tibia with two pectinate spurs. Hind tibia with two pectinate spurs. Posterior flange of hind coxa not produced as raised lamella. Metatibial gland absent. Metabasitarsal glands absent. Hind pretarsal claws simple. *Wings*: Tegula present, broad, demiovate in shape. Vein C in fore wing present. Pterostigma broad. Abscissa R·f3 present, running toward distal wing margin and enclosing cell with Rs·f5. Abscissae Rs·f2–3 present, connecting with Rs+M&M·f2. Cross-vein 2r-rs present, differentiated from Rs·f4 by presence of Rs·f2–3. Abscissae Rs·f4–5 differentiated into Rs·f4 and Rs·f5 by 2rs-m. Abscissa M·f2 in fore wing contiguous with Rs+M. Abscissa M·f4 in fore wing present, reaching wing margin. Cross-vein 1m-cu in fore wing present. Cross-vein cu-a in fore wing present, arising from M+Cu and proximal to M·f1. Vein Cu in fore wing present, with both branches Cu1 and Cu2. Vein A in fore wing with abscissae A·f1 and A·f2 present. Vein C in hind wing absent. Vein R in hind wing present, extending past Sc+R but not reaching distal wing margin. Vein Sc+R in hind wing present. Abscissa Rs·f1 in hind wing present, shorter than 1rs-m. Abscissa Rs·f2 in hind wing present, not reaching wing margin. Cross-vein 1rs-m in hind wing fused with M·f1. Vein M+Cu in hind wing present. Abscissa M·f1 in hind wing present. Abscissa M·f2 in hind wing present. Cross-vein cu-a in hind wing present. Vein Cu in hind wing present. Vein A in hind wing with abscissae A·f1 and A·f2 present.

***Gyne.*** Alate, similar to worker except for the mesosoma; known for several species. See descriptions in De Andrade (1998a).

***Larva.*** Not described. Cocoons absent.

#### Distribution.

*Cylindromyrmex* is an exclusively Neotropical lineage with ten extant species and three extinct species known from Dominican amber (De Andrade 1998a). Its distribution extends from the state of Veracruz, Mexico to Rio Grande do Sul in southern Brazil (De Andrade 1998a, Quiroz-Robledo 2003). Known from Cuba and Hispaniola, *Cylindromyrmex darlingtoni* is also the only member of the Dorylinae endemic in the Antilles. *Cylindromyrmex whymperi* has been apparently introduced and established in Galapagos Islands (De Andrade 1998a).

#### Taxonomy and phylogeny.

*Cylindromyrmex* has three generic synonyms: *Holcoponera* Cameron, *Hypocylindromyrmex* Wheeler, and *Metacylindromyrmex* Wheeler. Cameron’s *Holcoponera* has been considered a synonym since the end of 19th century (Forel 1892a), and the two other names were introduced as subgenera by Wheeler (1924a) but have not been used as valid since Brown’s (1975) work on the ‘Cerapachyinae’. De Andrade (1998a) revised, illustrated, and keyed all the species of *Cylindromyrmex*, subsequently adding new records and a second fossil taxon from Dominican amber (De Andrade 2001).

*Cylindromyrmex* is the sister genus to *Acanthostichus* (Brady et al. 2006, Brady et al. 2014, Borowiec, in prep.). A morphology-based internal phylogeny is also available, inferred by De Andrade (1998a).

#### Biology.

Members of this lineage have been reported to be termite predators (De Andrade 1998a). Some authors described *Cylindromyrmex* as termite inquilines based on records of workers from termite nests (Wheeler 1936, Overal and Bandeira 1985). It seems possible, however, that these specimens represent raiding foragers of arboreal-nesting ants, as complete nest series containing brood and reproductives are so far known apparently only from wood (Fernández and Escobar 1997, De Andrade 1998a, Mariano et al. 2004, Philip Ward pers. comm.).

A colony of *Cylindromyrmex whymperi* has been recently found in Peru and studied in captivity by Josh Richards, an ant keeper from Lima, Peru. He has observed that these ants readily pursue and sting termites, which are brought to the nest paralyzed but apparently not dead. When outnumbered in a confrontation, *Cylindromyrmex* workers first sting as many termites as possible before attempting to carry some of them back to the nest (Josh Richards pers. comm.).

Gobin et al. (2001) described a novel type of gland between sternites VI and VII in *Cylindromyrmex whymperi* and demonstrated that this species employs mass recruitment to termite prey. Morgan *et al*. (2008) chemically analyzed Dufour’s gland secretions of the same species. Three species of *Cylindromyrmex* (*Cylindromyrmex brasiliensis*, *Cylindromyrmex brevitarsus* and *Cylindromyrmex longiceps*) have been reported occurring in sympatry, collected in Malaise traps in a single locality in Bahia, Brazil. The flying males and gynes were present in samples from the end of August to beginning of December, with at least one of the samples containing all the three species (Delabie and Reis 2000).

All known queens of *Cylindromyrmex* are winged and brood production is apparently synchronized (Mariano et al. 2004, Josh Richards pers. comm.).

#### Species of *Cylindromyrmex*

†*Cylindromyrmex antillanus* De Andrade, 1998a: Dominican amber

*Cylindromyrmex boliviae* Wheeler, W. M., 1924a: Bolivia

*Cylindromyrmex brasiliensis* Emery, 1901a: Brazil

*Cylindromyrmex brevitarsus* Santschi, 1925: Brazil

*Cylindromyrmex darlingtoni* Wheeler, W. M., 1937: Cuba

†*Cylindromyrmex electrinus* De Andrade, 1998a: Dominican amber

*Cylindromyrmex escobari* De Andrade, 1998a: Colombia

*Cylindromyrmex godmani* Forel, 1899: Panama

†*Cylindromyrmex inopinatus* De Andrade, 2001: Dominican amber

*Cylindromyrmex longiceps* André, 1892: Brazil

*Cylindromyrmex meinerti* Forel, 1905: Venezuela

*Cylindromyrmex striatus* Mayr, 1870: Suriname

*Cylindromyrmex whymperi* (Cameron, 1891): Ecuador

### Dorylus

Taxon classificationAnimaliaHymenopteraFormicidae

Fabricius, 1793

= Alaopone Emery, 1881, **syn. n.**
= Anomma Shuckard, 1840c, **syn. n.**
= Cosmaecetes Spinola, 1851
= Dichthadia Gerstäcker, 1863, **syn. n.**
= Rhogmus Shuckard, 1840c, **syn. n.**
= Shuckardia Emery, 1895b
= Sphecomyrmex Schulz, 1906
= Sphegomyrmex Imhoff, 1852
= Typhlopone Westwood, 1839, **syn. n.**

#### Type-species.

*Vespa helvola*, by monotypy.

The Afrotropical ‘driver ants’ of this genus epitomize the army ant lifestyle, but they represent only a fraction of the diversity of *Dorylus*. Most species are much less commonly observed, and forage underground or in leaf litter.

#### Diagnosis.

***Worker.*** The workers of *Dorylus* are readily recognized by a combination of well-developed promesonotal Pronotomesopleural suture, propodeal spiracle positioned high on the propodeum and lack of propodeal lobes, single waist segment, pygidium large and with a flattened surface and armed with two cuticular projections, and pretarsal claws simple. Other army ants of the Old World, *Aenictus* and *Aenictogiton*, are not easily confused with *Dorylus* as the former always has a well-differentiated second waist segment (postpetiole) and in *Aenictogiton* the gaster has more developed constrictions between gastral pre- and post-sclerites, resulting in apparent constriction between abdominal segments IV, V, and VI. *Yunodorylus* is superficially similar but is easily distinguished from all army ants by the propodeal spiracle situated low and presence of propodeal lobes. Among the New World army ants only *Cheliomyrmex* has one-segmented waist but *Cheliomyrmex* does not have a promesonotal Pronotomesopleural suture, its pygidium is reduced and never armed with cuticular projections, and its pretarsal claws are armed with a tooth.

***Male.*** In general appearance *Dorylus* males are similar to other army ant genera but possess flattened femora that are much broader and more compressed than the tibiae and tarsi. This trait alone is sufficient to separate them from all other male dorylines, but a combination of single-segmented waist, M·f1 vein of fore wing arising from M+Cu at about 45° and situated near to cu-a, Rs·f2–3 lost, pterostigma narrow and inconspicuous can also be used to recognize *Dorylus*. The Old World army ant genera *Aenictus* and *Aenictogiton* have similar fore wing venation but both have a well-developed and broad pterostigma and the latter has a ‘free-hanging’ Rs·f3 vein. In the New World army ants M·f1 arises at a lower angle and is conspicuously proximal to cu-a, and Rs·f2–3 are present, forming two submarginal cells. *Dorylus* males also possess unique genital capsule morphology, where a tiny diamond-shaped structure is formed from a fragment of the basimeres and visible dorsally over the aedeagus (‘patella’ of Birket-Smith 1981; Brendon Boudinot pers. comm.). The telomeres in lateral view do not conceal inner valves of the genital capsule as in most dorylines but instead form a characteristic shape of a spiral arm folding first proximally and then projecting distally over the rest of genital capsule thus concealing it from above.

#### Description.

***Worker.*** *Head*: Antennae with 8, 9, 11, or 12 segments. Apical antennal segment not enlarged, not broader and longer than two preceding segments combined. Clypeus without cuticular apron. Lateroclypeal teeth absent. Parafrontal ridges absent. Torulo-posttorular complex vertical. Antennal scrobes absent. Labrum without median notch or concavity. Labrum with median notch or concavity. Proximal face of stipes projecting beyond inner margin of sclerite, concealing prementum when mouthparts fully closed. Maxillary palps 2- or 1-segmented. Labial palps 2-segmented. Mandibles elongately triangular to falcate, with teeth on elongated masticatory margin. Eyes absent. Ocelli absent. Head capsule with differentiated vertical posterior surface above occipital foramen. Ventrolateral margins of head without lamella or ridge extending towards mandibles and beyond carina surrounding occipital foramen. Posterior head corners dorsolaterally immarginate. Carina surrounding occipital foramen entirely absent, including ventrally. *Mesosoma*: Pronotal flange not separated from collar by distinct ridge. Promesonotal connection with Pronotomesopleural suture conspicuous and complete, but immobile. Pronotomesopleural suture complete, continuous with promesonotal Pronotomesopleural suture. Mesometapleural groove deeply impressed, conspicuous. Transverse groove dividing mesopleuron absent. Pleural endophragmal pit concavity present. Mesosoma dorsolaterally immarginate. Metanotal depression or groove on mesosoma absent. Propodeal spiracle situated high on sclerite. Propodeal declivity without distinct dorsal edge or margin and rectangular in posterior view. Metapleural gland with bulla visible through cuticle in smaller workers, mostly obscured in large workers. Propodeal lobes absent. *Metasoma*: Petiole anterodorsally immarginate, dorsolaterally immarginate, and laterally above spiracle immarginate. Helcium in relation to tergosternal Pronotomesopleural suture placed at posttergite and axial. Prora forming a simple U-shaped margin. Spiracle openings of abdominal segments IV–VI circular. Abdominal segment III anterodorsally immarginate and dorsolaterally immarginate. Abdominal segment III more than half size of succeeding segment IV, which is weakly constricted at presegmental portion (uninodal waist). Girdling constriction of segment IV present, i.e. pre- and postsclerites distinct. Cinctus of abdominal segment IV gutter-like and sculptured but not cross-ribbed. Abdominal segment IV not conspicuously largest segment. Abdominal tergite IV not folding over sternite, and anterior portions of sternite and tergite equally well visible in lateral view. Girdling constriction between pre- and posttergites of abdominal segments V and VI present. Girdling constriction between pre- and poststernites of abdominal segments V and VI present. Pygidium large, with impressed medial field, and armed with cuticular spines. Hypopygium unarmed. *Legs*: Mid tibia with single pectinate spur. Hind tibia with single pectinate spur. Hind basitarsus not widening distally, circular in cross-section. Posterior flange of hind coxa not produced as raised lamella. Metatibial gland present as oval patch of whitish cuticle. Metabasitarsal gland absent. Hind pretarsal claws simple. *Polymorphism*: Highly polymorphic.

***Male.*** *Head*: Antennae with 13 segments. Clypeus without cuticular apron. Parafrontal ridges absent. Torulo-posttorular complex vertical, carinae separated by broad flat or convex area between exposed antennal sockets. Maxillary palps 2- or 1-segmented. Labial palps 1-segmented. Mandibles falcate. Ventrolateral margins of head without lamella or ridge extending towards mandibles and beyond carina surrounding occipital foramen. Carina surrounding occipital foramen ventrally absent. *Mesosoma*: Pronotal flange not separated from collar by distinct ridge. Notauli absent. Transverse groove dividing mesopleuron absent. Propodeal declivity reduced, without distinct dorsal edge or margin. Metapleural gland opening absent. Propodeal lobes absent. *Metasoma*: Petiole anterodorsally immarginate, dorsolaterally immarginate, and laterally above spiracle immarginate. Helcium in relation to tergosternal Pronotomesopleural suture placed at posttergite and axial. Prora simple, not delimited by carina. Spiracle openings of abdominal segments IV–VI slit-shaped. Abdominal segment III more than half size of succeeding segment IV; latter weakly constricted at presegmental portion (uninodal waist). Girdling constriction of segment IV absent, i.e. pre- and postsclerites indistinct. Cinctus of abdominal segment IV absent, not impressed. Girdling constriction between pre- and postsclerites of abdominal segments V and VI absent. Abdominal segment IV not conspicuously largest segment. Abdominal sternite VII simple. Abdominal sternite IX distally armed with two spines, with lateral apodemes short, directed sideways. *Genitalia*: Cupula short relative to rest of genital capsule and shorter ventrally than dorsally. Basimere fused basally, with a fragment reduced to tiny, plate-like sclerite. Telomere folding backwards and then over rest of genital capsule, concealing it dorsally. Volsella gradually tapering toward apex. Penisvalva laterally compressed, rounded at apex. *Legs*: Mid tibia with single pectinate spur. Hind tibia with single pectinate spur. Posterior flange of hind coxa not produced as raised lamella. Metatibial gland absent. Metabasitarsal glands absent. Hind pretarsal claws simple. *Wings*: Tegula present, broad, demiovate in shape. Vein C in fore wing present. Pterostigma narrow. Abscissa R·f3 absent. Abscissae Rs·f2–3 absent. Cross-vein 2r-rs present, connected to Rs·f2–3&Rs·f4. Abscissae Rs·f4–5 differentiated into Rs·f4 and Rs·f5 by 2rs-m. Abscissa M·f2 in fore wing contiguous with Rs+M. Abscissa M·f4 in fore wing present, reaching wing margin. Cross-vein 1m-cu in fore wing present. Cross-vein cu-a in fore wing present, arising from Cu and distal to, at or near M·f1. Vein Cu in fore wing present, with only Cu1 branch prominent. Vein Cu in fore wing present, with both branches Cu1 and Cu2. Vein A in fore wing with abscissae A·f1 and A·f2 present. Vein C in hind wing unknown. Vein C in hind wing present. Vein R in hind wing present, extending past Sc+R but not reaching distal wing margin. Vein Sc+R in hind wing present. Abscissa Rs·f1 in hind wing present, shorter than 1rs-m. Abscissa Rs·f2 in hind wing present, not reaching wing margin. Cross-vein 1rs-m in hind wing present, shorter than M·f1. Vein M+Cu in hind wing present. Abscissa M·f1 in hind wing present. Abscissa M·f2 in hind wing present. Cross-vein cu-a in hind wing present. Vein Cu in hind wing present. Vein A in hind wing with abscissae A·f1 and A·f2 present.

***Gyne.*** Dichthadiiform, blind, with median ocellus (see e.g. Barr et al. 1985).

***Larva.*** Larvae of *Dorylus* have been described in Wheeler (1943) and Wheeler and Wheeler (1984). Cocoons are absent.

#### Distribution.

*Dorylus* ranges from Sub-Saharan Africa throughout North Africa and Asia Minor to Borneo in Southeast Asia. The Afrotropics harbor the highest number of species and are the home of the surface- and leaf litter-foraging species.

#### Taxonomy and phylogeny.

The long and confusing taxonomic history of the genus begins with a male ant from South Africa, described as *Vespa helvola* by Linnaeus in 1764. Later Fabricius (1793) created the genus *Dorylus* for that species. Similarly to *Aenictus*, for a time the males and females were known under different generic names, with *Dorylus* being applied to males and *Anomma* and *Typhlopone* to the workers. 85 years after the original description of *Vespa helvola*, T. S. Savage observed males and workers together in the field and recognized that they belonged to one species (Savage 1849). A very readable overview of the early taxonomic history of *Dorylus* can be found in Gotwald (1995: 13). The modern subgeneric division of *Dorylus* was stabilized by Emery (1895b, 1910). This classification has come under scrutiny using molecular data in the recent decades, and two of the most speciose subgenera of *Dorylus*, *Anomma* and *Dorylus* *s. str.* were found to be not monophyletic (Kronauer et al. 2007). Because of these phylogenetic considerations, also backed up by morphological study (Caspar Schöning pers. comm.), I propose to abandon the traditional subgeneric classification. Although the surface-foraging (as opposed to leaf litter) species of *Anomma* species form a clade and it is even possible to differentiate it based on apomorphic morphological characters from other *Dorylus* (Schöning et al. in preparation), recognizing *Anomma* would likely leave the large *Dorylus* *s. str.* paraphyletic. Other *Dorylus* subgenera are likely monophyletic (Kronauer et al. 2007). Subgeneric classification is not currently adopted for any other doryline genus, and I propose the following informal species-groups to be recognized instead of the subgenera (for species known from the worker caste):

***Dorylus orientalis*-group** (equivalent of *Alaopone*), comprising species *acutus*, *aethiopicus*, *atriceps*, *attenuatus*, *brevis*, *buyssoni*, *conradti*, *diadema*, *distinctus*, *ductor*, *katanensis*, *montanus*, *orientalis*, *vishnui*.

***Dorylus nigricans*-group** (equivalent of *Anomma* excluding *emeryi* and *kohli* (Schöning et al. 2008)), comprising species *atratus*, *erraticus*, *funereus*, *mayri*, *niarembensis*, *nigricans*, *rufescens*, *stanleyi*, *wilverthi*.

***Dorylus laevigatus*-group** (equivalent of *Dichthadia*), comprising species *laevigatus*.

***Dorylus politus*-group** (species excluded from *Dorylus* *s. str.* based on phylogeny in Kronauer et al. 2007), comprising species *politus*, *spininodis*.

***Dorylus helvolus*-group** (equivalent of *Dorylus* *s. str.* but excluding species of *politus*-group and including two species previously assigned to *Anomma*), comprising species *affinis*, *agressor*, *alluaudi*, *bequaerti*, *bishyiganus*, *braunsi*, *brevipennis*, *congolensis*, *depilis*, *emeryi*, *faurei*, *furcatus*, *gaudens*, *ghanensis*, *gribodoi*, *helvolus*, *kohli*, *mandibularis*, *moestus*, *schoutedeni*, *stadelmani*, *staudingeri*, *striatidens*, *titan*.

***Dorylus fimbriatus*-group** (equivalent of *Rhogmus*), comprising species *fimbriatus*, *fuscipennis*, *leo*, *ocellatus*, *savagei*, *termitarius*.

***Dorylus fulvus*-group** (equivalent of *Typhlopone*), comprising species *fulvus*, *labiatus*.

Species unassigned to species-groups: *atratus*, *westwoodii*.

*Dorylus* is the sister taxon to *Aenictogiton* (Brady et al. 2006, 2014, Borowiec, in prep.). As explained above, the internal phylogeny of the genus (Kronauer et al. 2007) shows that the subgenera *Anomma* and *Dorylus* as they were traditionally defined are not monophyletic. The Asian species *Dorylus laevigatus* represents the earliest-branching lineage of the genus. The time-calibrated phylogeny of Kronauer et al. (2007) estimated crown group age of *Dorylus* to be between 30 and 64 million years, but more recent studies suggest much younger ages at about 22 million years (Brady et al. 2014) or even younger than 20 million years (Borowiec, in prep.).

#### Biology.

Because some species of this lineage are so conspicuous and are the most important arthropod predators of the Afrotropics, this group has attracted considerable attention.

The best studied species include the Afrotropical species that forage above ground (Raignier and Boven 1955, Raignier 1972, Gotwald 1995), but one subterranean species, *Dorylus laevigatus* has been the subject of some work (Berghoff et al. 2002a, b, 2003a, b, Weissflog et al. 2000). Good overviews of *Dorylus* biology can be found in Raignier and Boven (1955) and Gotwald (1995). The surface- and leaf litter-foraging species have been collectively referred to as ‘driver ants’ (Savage 1847), and traditionally classified in the polyphyletic subgenus *Anomma* (see Taxonomy and phylogeny above). Here I follow this convention and use the terms ‘driver ants’ and ‘surface-‘ or ’epigaeically-foraging species’ interchangeably.

The life cycle of *Dorylus* colony is similar to that of *Eciton* and many other army ants but there are no pronounced nomadic and statary phases. The brood production is not synchronized (Gotwald 1995, Schöning et al. 2005b), and the colonies move from old to new nesting sites at irregular intervals (Gotwald and Cunningham van Sommeren 1990, Schöning et al. 2005b). A mature colony will produce about a dozen virgin queens and eventually undergo fission. About half of the worker force will depart with the old, fertilized queen, while the other half will remain with the virgin queens. Ultimately, all except one of the new queens are cannibalized (Raignier 1972). The new colony does not produce sexual brood until the workers mothered by the old queen have died (Kronauer et al. 2004).

Copulation in *Dorylus* has been observed only once (Kronauer and Boomsma 2007a). Males collected at lights and two inseminated queens from established *Dorylus molestus* bivouacs were coupled under laboratory conditions. The male first uses his sickle-shaped mandibles to grasp the queen behind her petiole and performs bending movements, searching the tip of the queen’s abdomen. Once engaged, the pairs remained in copulation for five to ten hours. After this period, the male relaxes his grip on queen’s petiole but remains connected to the queen. Twenty hours after the copulations, the two pairs were killed and dissected, both males remaining attached to the queens. The males apparently succeeded in transferring sperm to the queens, and the dissections confirmed that the male accessory testes were empty after the copulations. Despite these observations, Kronauer and Boomsma (2007a) find little evidence for army ant queens re-mating later in life and point out that the males were not attracted to old queens in most trials.

The reproductive potential of *Dorylus* queens is impressive, at least in the surface-foraging species studied thus far. The queen mates between 15–20 times (Kronauer et al. 2004, 2006) early in her life and stores up to 880 million spermatozoa (Kronauer and Boomsma 2007a). A *Dorylus wilverthi* queen can produce an estimated 3–4 million eggs per month, for a total over 250 million eggs during her lifetime (Raignier and Boven 1955, Kronauer and Boomsma 2007b). This is even more than *Eciton* queens (see under *Eciton*; Schneirla 1971, Kronauer and Boomsma 2007b).

These army ants always occupy subterranean nests, either constructed by excavating large amounts of soil and/or taking advantage of a preexisting cavity (Schöning et al. 2005b, Boven and Lévieux 1968). Because of these underground habits, colony size estimates are rare. A single excavated colony of *Dorylus laevigatus* contained about 300,000 workers (Berghoff et al. 2002), and estimates of colony size for the surface foragers *Dorylus nigricans* and *Dorylus wilverthi* range from 1 million to over 20 million workers (Voessler 1905, Raignier and Boven 1955). The dry mass of *Dorylus nigricans* colonies has been estimated to be 9–15 kg (Leroux 1982). The underground nests of *Dorylus* are quite different from the above-ground bivouacs of *Eciton* (Gotwald 1995). Raignier and Boven (1955) categorized them as either occupying a single large chamber or dispersed among subterranean galleries and chambers. The first type is exemplified by *Dorylus wilverthi* and the second by *Dorylus nigricans*. Both nest types are often found among root systems of trees. These ants actively excavate soil and one estimate gives 20 kg of soil per day removed in the first week of a *Dorylus nigricans* colony settling into a new site (Leroux 1977).

*Dorylus* emigrate irregularly and the colony often returns to the same nesting spot. Gotwald and Cunningham van Sommeren (1990) followed a single colony of *Dorylus molestus* for 432 days and observed 38 emigrations during that time, spanning an area of about 5 hectares. One colony of *Dorylus nigricans* has been recorded to remain in one bivouac site for 125 consecutive days (Raignier and Boven 1955). The adaptive significance of the cycles in brood production and colony activity remains unclear, but it seems to be correlated with highly variable food availability (Kronauer 2009). While phasic species of *Aenictus*, *Eciton*, and other New World army ants rely heavily on brood of other social insects, *Dorylus* are more generalist (Gotwald 1995).

*Dorylus* gynes may or may not be able to move on their own during nest emigration. All queen specimens known so far are missing tarsal segments (Raignier 1972, Berghoff et al. 2002), so that they are assisted to a new site by the entourage of workers (Berghoff et al. 2002). Raignier (1972) observed missing tarsal segments in very young queens of *Dorylus nigricans*, prior to their first emigration. The causes and significance of this tarsal mutilation are not known.

A diversity of foraging habits and prey preferences has been documented for *Dorylus* (Gotwald 1995). According to the most popular classification (Schöning et al. 2005a, Kronauer et al. 2007), three major foraging strategies can be distinguished: subterranean, leaf litter, or surface foragers. The surface-swarming driver ants are generalist predators that will take any kind of prey, ranging from immatures of other insects to vertebrate carrion (Schöning and Moffett 2007). Seasonal, habitat, and intraspecific differences can be seen in prey composition and intake in these ants, but the proportion of social insect prey is small (Schöning et al. 2008). This is in contrast to *Eciton burchellii*, whose diet is general but it still relies heavily on this kind of prey (Rettenmeyer 1963). The few subterranean species of *Dorylys* that have been studied have also been recorded to be generalist predators but additionally often feeding on termites (Darlington 1985, Berghoff et al. 2002). *Dorylus orientalis* is recognized as a vegetable crop pest, apparently being mainly or exclusively herbivorous (Roonwal 1975). Variation in foraging can also be seen within the general foraging strategies. Schöning et al. (2008) reported that two surface-swarming species, *Dorylus wilverthi* and *Dorylus molestus*, differ in their diets and raiding behavior. *Dorylus molestus* is often seen capturing earthworms and exhibits digging behavior, while earthworms are rarely a major component of the diet for *Dorylus wilverthi*, whose workers have not been observed digging. Two sympatric, subterranean species of *Dorylus* from Asia have also been compared and shown to differ in their foraging behavior and prey preference (Berghoff et al. 2003).

Kronauer et al. (2007) used molecular phylogenetics and ancestral state reconstruction to address the evolution of the foraging niche in *Dorylus*. They categorized species as either subterranean, leaf litter, or surface foragers and inferred that subterranean foraging was the ancestral state for the genus. Both surface and leaf litter foraging strategies likely evolved once within *Dorylus*. The descendants of a leaf litter-dwelling ancestor gave rise to both surface foragers and species that reverted to subterranean foraging. An earlier study (Schöning et al. 2005a) showed how allometry in the worker caste is correlated with the foraging niche, although the authors did not examine this in a phylogenetic framework (Felsenstein 1985). These authors demonstrated that surface-adapted species possess appendages and mandibles that are longer relatively to their body size than in the leaf-litter and in the subterranean foragers. Kronauer et al. (2007) further assessed allometry in the context of *Dorylus* phylogeny and concluded that the species that reverted to underground foraging re-evolved morphology similar to the ancestral, short-limbed condition.

Similarly to New World army ants, *Dorylus* colonies have numerous invertebrate and vertebrate associates, although these companion faunas are not as well described (Gotwald 1995). Remarkably, the foragers of African driver ants are followed by several species of birds specializing on prey flushed by the ants, much like the swarms of *Eciton burchellii* in the New World (Peters et al. 2008). Other vertebrates, such as chimpanzees are known to rely on *Dorylus* for food (Kingdon 1997, Schöning et al. 2007, Sanz et al. 2010). Because the apes utilize sticks and plant stems to extract the ants, this is an important study system in the primate culture and tool use (Humle 2011).

A variety of other research has been carried out on *Dorylus*, but most of these studies are isolated in nature. Kronauer et al. (2011) documented significant amounts of hybridization between the driver ants *Dorylus wilverthi* and *Dorylus molestus*, and Barth et al. (2013) undertook a population genetics study on *Dorylus fulvus*.

#### Species of *Dorylus*

*Dorylus acutus* Santschi, 1937a: Democratic Republic of the Congo

*Dorylus aethiopicus* Emery, 1895b: ‘Sudan, Abessinien, Tunis’

*Dorylus affinis* Shuckard, 1840c: Gambia

*Dorylus affinis aegyptiacus* Mayr, 1865: Egypt

*Dorylus affinis denudatus* Santschi, 1910c: Niger

*Dorylus affinis exilis* Santschi, 1914a: Tanzania

*Dorylus affinis hirsutus* Wheeler, W. M., 1922a: Egypt, Ethiopia

*Dorylus affinis loewyi* Forel, 1907b: Tanzania

*Dorylus affinis parapsidalis* Santschi, 1917: Malawi

*Dorylus affinis pulliceps* Santschi, 1917: Ivory Coast

*Dorylus affinis sudanicus* Santschi, 1917: Chad

*Dorylus affinis ugandensis* Santschi, 1914a: Uganda

*Dorylus agressor* Santschi, 1923b: Democratic Republic of the Congo

*Dorylus alluaudi* Santschi, 1914a: Uganda

*Dorylus alluaudi lobatus* Santschi, 1919b: Democratic Republic of the Congo

*Dorylus atratus* Smith, F., 1859: Nigeria

*Dorylus atriceps* Shuckard, 1840c: Gambia

*Dorylus attenuatus* Shuckard, 1840c: Gambia

*Dorylus attenuatus acuminatus* Emery, 1899b: South Africa

*Dorylus attenuatus australis* Santschi, 1919a: South Africa

*Dorylus attenuatus bondroiti* Santschi, 1912: South Africa

*Dorylus attenuatus latinodis* Forel, 1920: Democratic Republic of the Congo

*Dorylus bequaerti* Forel, 1913a: Democratic Republic of the Congo

*Dorylus bishyiganus* (Boven, 1972): Rwanda

*Dorylus braunsi* Emery, 1895b: Liberia

*Dorylus braunsi anceps* Forel, 1914: Zimbabwe

*Dorylus brevipennis* Emery, 1895b: Tanzania

*Dorylus brevipennis marshalli* Emery, 1901d: Zimbabwe

*Dorylus brevipennis zimmermanni* Santschi, 1910c: Republic of the Congo

*Dorylus brevis* Santschi, 1919b: Democratic Republic of the Congo

*Dorylus buyssoni* Santschi, 1910c: Kenya

*Dorylus buyssoni conjugens* Santschi, 1910c: Kenya

*Dorylus congolensis* Santschi, 1910: Republic of the Congo

*Dorylus conradti* Emery, 1895b: Togo

*Dorylus conradti berlandi* Santschi, 1926a: Ivory Coast

*Dorylus depilis* Emery, 1895b: Cameroon

*Dorylus depilis clarior* Santschi, 1917: Democratic Republic of the Congo

*Dorylus diadema* Gerstäcker, 1859: Mozambique

*Dorylus diadema arnoldi* Forel, 1914: Zimbabwe

*Dorylus diadema fusciceps* Emery, 1899b: Malawi

*Dorylus distinctus* Santschi, 1910c: Guinea

*Dorylus ductor* Santschi, 1939: ‘Congo’

*Dorylus emeryi* Mayr, 1896: Cameroon

*Dorylus emeryi opacus* Forel, 1909b: Democratic Republic of the Congo

*Dorylus emeryi pulsi* (Forel, 1904): ‘Afrique occidentale’

*Dorylus erraticus* (Smith, F., 1865): ‘New Guinea’ (labeling error: Wilson 1964: 443)

*Dorylus faurei* Arnold, 1946: South Africa

*Dorylus fimbriatus* (Shuckard, 1840c): Gambia

*Dorylus fimbriatus crampeli* Santschi, 1919a: Central African Republic

*Dorylus fimbriatus laevipodex* Santschi, 1919a: Kenya

*Dorylus fimbriatus poweri* Forel, 1914: South Africa

*Dorylus fulvus* (Westwood, 1839): ‘North Africa’

*Dorylus fulvus badius* Gerstäcker, 1859: Mozambique

*Dorylus fulvus crosi* Santschi, 1926b: Algeria

*Dorylus fulvus dentifrons* Wasmann, 1904: Democratic Republic of the Congo

*Dorylus fulvus eurous* Emery, 1915b: Ethiopia

*Dorylus fulvus glabratus* Shuckard, 1840c: Gambia

*Dorylus fulvus juvenculus* Shuckard, 1840c: Morocco

*Dorylus fulvus mordax* Santschi, 1931: Ivory Coast

*Dorylus fulvus obscurior* Wheeler, W. M., 1925a: Guinea

*Dorylus fulvus punicus* Santschi, 1926b: Tunisia

*Dorylus fulvus ruficeps* Santschi, 1926b: Lebanon

*Dorylus fulvus saharensis* Santschi, 1926b: ‘Sahara’

*Dorylus funereus* Emery, 1895b: Ghana

*Dorylus funereus acherontus* Santschi, 1937b: Cameroon

*Dorylus funereus pardus* Santschi, 1937b: Democratic Republic of the Congo

*Dorylus funereus stygis* Santschi, 1937b: Democratic Republic of the Congo

*Dorylus funereus zumpti* Santschi, 1937b: Cameroon

*Dorylus furcatus* (Gerstäcker, 1872): South Africa

*Dorylus fuscipennis* (Emery, 1892): Ghana

*Dorylus fuscipennis lugubris* Santschi, 1919a: Ivory Coast

*Dorylus fuscipennis marginiventris* Santschi, 1919a: Ivory Coast

*Dorylus gaudens* Santschi, 1919b: Democratic Republic of the Congo

*Dorylus ghanensis* Boven, 1975: Ghana

*Dorylus gribodoi* Emery, 1892: Togo

*Dorylus helvolus* (Linnaeus, 1764): South Africa

*Dorylus helvolus pretoriae* Arnold, 1946: South Africa

*Dorylus katanensis* Stitz, 1911: Democratic Republic of the Congo

*Dorylus kohli* Wasmann, 1904: Democratic Republic of the Congo

*Dorylus kohli chapini* Wheeler, W. M., 1922a: Democratic Republic of the Congo

*Dorylus kohli frenisyi* Forel, 1916: Democratic Republic of the Congo

*Dorylus kohli indocilis* Santschi, 1933: Democratic Republic of the Congo

*Dorylus kohli langi* Wheeler, W. M., 1922a: Democratic Republic of the Congo

*Dorylus kohli militaris* Santschi, 1923b: Democratic Republic of the Congo

*Dorylus kohli minor* Santschi, 1911a: Angola

*Dorylus kohli victoriae* Santschi, 1921a: Uganda

*Dorylus labiatus* Shuckard, 1840c: India

*Dorylus laevigatus* (Smith, F., 1857): Malaysia (Sarawak)

*Dorylus leo* Santschi, 1919a: Ivory Coast

*Dorylus mandibularis* Mayr, 1896: Cameroon

*Dorylus mandibularis pulchellus* Santschi, 1920a: Ivory Coast

*Dorylus mayri* Santschi, 1912: Cameroon

*Dorylus moestus* Emery, 1895b: Democratic Republic of the Congo

*Dorylus moestus claripennis* Santschi, 1919b: Democratic Republic of the Congo

*Dorylus moestus morio* Santschi, 1919b: Republic of the Congo

*Dorylus moestus schereri* Forel, 1911d: Liberia

*Dorylus montanus* Santschi, 1910c: Tanzania

*Dorylus niarembensis* (Boven, 1972): Democratic Republic of the Congo

*Dorylus nigricans* Illiger, 1802: Sierra Leone

*Dorylus nigricans arcens* (Westwood, 1847): Liberia

*Dorylus nigricans burmeisteri* (Shuckard, 1840c): Sierra Leone

*Dorylus nigricans molestus* (Gerstäcker, 1859): Mozambique

*Dorylus nigricans pallidus* Santschi, 1921a: Cameroon

*Dorylus nigricans rubellus* (Savage, 1849): Gabon

*Dorylus nigricans sjoestedti* Emery, 1899b: Cameroon

*Dorylus nigricans sjostedtiwilverthi* (Wasmann, 1917): Cameroon

*Dorylus nigricans terrificus* Santschi, 1923b: Democratic Republic of the Congo

*Dorylus ocellatus* (Stitz, 1910): Cameroon

*Dorylus orientalis* Westwood, 1835: India

*Dorylus orientalis obscuriceps* Santschi, 1920b: India

*Dorylus politus* Emery, 1901d: Cameroon

*Dorylus rufescens* Santschi, 1915: Cameroon

*Dorylus savagei* Emery, 1895b: ‘Gabon und Congo’

*Dorylus savagei mucronatus* Emery, 1899b: Nigeria

*Dorylus schoutedeni* Santschi, 1923b: Democratic Republic of the Congo

*Dorylus spininodis* Emery, 1901d: Cameroon

*Dorylus spininodis longiceps* Viehmeyer, 1914: Tanzania

*Dorylus stadelmanni* Emery, 1895b: Democratic Republic of the Congo

*Dorylus stanleyi* Forel, 1909b: Democratic Republic of the Congo

*Dorylus staudingeri* Emery, 1895b: Democratic Republic of the Congo

*Dorylus striatidens* Santschi, 1910c: Senegal

*Dorylus termitarius* Wasmann, 1911: Democratic Republic of the Congo

*Dorylus titan* Santschi, 1923b: Democratic Republic of the Congo

*Dorylus titan vinalli* Santschi, 1933: Democratic Republic of the Congo

*Dorylus vishnui* Wheeler, W. M., 1913: Myanmar

*Dorylus westwoodii* (Shuckard, 1840b): ‘South America’ (locality incorrect)

*Dorylus wilverthi* Emery, 1899b: Democratic Republic of the Congo

### Eburopone gen. n.

Taxon classificationAnimaliaHymenopteraFormicidae

http://zoobank.org/973EFBC7-99F7-418D-B49A-9D4D948EC167

#### Type-species.

*Cerapachys wroughtoni*, by present designation.

Only one species of this group has been described from Afrotropics, but Madagascar harbors a considerable undescribed diversity.

#### Diagnosis.

***Worker.*** Workers of *Eburopone* are most easily recognized from other dorylines by a unique whitish patch of cuticle of presumably glandular function present on the posterior edge of abdominal sternite IV, although the patch may be faint in small or pale-colored specimens. A combination of 12-segmented antennae, propodeal spiracle placed low on the sclerite and propodeal lobes present, petiole dorsolaterally immarginate, lack of conspicuous constrictions posterior to abdominal segment IV, helcium narrow and placed at about mid-height of the segment, pronotomesopleural Pronotomesopleural suture present, and mid and hind tibiae each with a single pectinate spur will serve to distinguish *Eburopone* workers from other dorylines. In the Afrotropics and in Madagascar other non-army dorylines include *Ooceraea*, *Parasyscia*, *Lividopone*, *Lioponera*, and *Zasphinctus*. None of these genera possesses the characteristic, apparently glandular, patch on the underside of gaster, but if that character is not obvious or obscured, it is still relatively easy to distinguish *Eburopone*: *Ooceraea* found in this region (*Ooceraea biroi*) have 9-segmented antennae, *Parasyscia* and *Lividopone* have pronotomesopleural sutures fused, and *Lioponera* has a dorsolaterally marginate petiole and a raised flange on hind coxa. *Zasphinctus* belongs to the genera with pronounced constrictions between abdominal segments IV, V, and VI.

***Male.*** The male morphology of *Eburopone* is very variable, including wing venation, but the following combination of characters usually allows separation from other genera: Antennae with 13 segments, at least weak constriction present anterior to abdominal segment IV, costal vein (C) present in the fore wing, submarginal cell open, presence of R·f3 and a free ‘stigmal vein’ formed by 2r-rs and Rs·f4–5 in the absence of Rs·f2–3 or 2rs-m, not running to the wing margin. Among non-army ant dorylines that overlap in range with *Eburopone*, *Lioponera* and *Ooceraea* can have a free stigmal vein but these genera never have costal vein running along the anterior margin of the fore wing in combination with R·f3 present past pterostigma.

#### Description.

***Worker.*** *Head*: Antennae with 12 segments. Apical antennal segment moderately enlarged, broader than and about equal in length to two preceding segments combined to conspicuously enlarged, much broader than and longer than two preceding segments combined. Clypeus without cuticular apron. Lateroclypeal teeth present. Parafrontal ridges reduced. Torulo-posttorular complex vertical. Antennal scrobes absent. Labrum with median notch or concavity. Proximal face of stipes projecting beyond inner margin of sclerite, concealing prementum when mouthparts fully closed. Maxillary palps 2-segmented. Labial palps 2-segmented. Mandibles triangular, edentate. Eyes absent or present, composed of at most several weakly differentiated ommatidia. Ocelli absent. Head capsule with differentiated vertical posterior surface above occipital foramen. Ventrolateral margins of head without lamella or ridge extending towards mandibles and beyond carina surrounding occipital foramen. Posterior head corners dorsolaterally immarginate. Carina surrounding occipital foramen ventrally present. *Mesosoma*: Pronotal flange often separated from collar by ridge, usually distinct but rarely poorly developed or absent. Promesonotal connection with Pronotomesopleural suture present, weakly differentiated or with Pronotomesopleural suture conspicuous and complete but immobile. Pronotomesopleural suture visible as groove but not unfused. Mesometapleural groove deeply impressed, conspicuous. Transverse groove dividing mesopleuron present. Pleural endophragmal pit concavity present. Mesosoma dorsolaterally immarginate. Metanotal depression or groove on mesosoma absent. Propodeal spiracle situated low on sclerite. Propodeal declivity with distinct dorsal edge or margin and rectangular in posterior view. Metapleural gland usually with bulla visible through cuticle, sometimes obscured. Propodeal lobes present, well developed. *Metasoma*: Petiole anterodorsally immarginate or marginate, dorsolaterally immarginate, and laterally above spiracle marginate. Helcium in relation to tergosternal Pronotomesopleural suture placed at posttergite and axial. Prora forming a U-shaped margin with median ridge. Spiracle openings of abdominal segments IV–VI circular. Abdominal segment III anterodorsally immarginate and dorsolaterally immarginate. Abdominal segment III more than half size of succeeding segment IV, which is weakly constricted at presegmental portion (uninodal waist). Abdominal segment III about half size of succeeding segment IV, which is strongly constricted at presegmental portion (binodal waist). Girdling constriction of segment IV present, i.e. pre- and postsclerites distinct. Cinctus of abdominal segment IV gutter-like and cross-ribbed; sculpturing may be weak. Abdominal segment IV not conspicuously largest segment. Abdominal segment IV conspicuously largest segment. Abdominal tergite IV not folding over sternite, and anterior portions of sternite and tergite equally well visible in lateral view. Girdling constriction between pre- and posttergites of abdominal segments V and VI absent. Girdling constriction between pre- and poststernites of abdominal segments V and VI absent. Pygidium large, with impressed medial field, and armed with modified setae. Hypopygium unarmed. *Legs*: Mid tibia with single pectinate spur. Hind tibia with single pectinate spur. Hind basitarsus not widening distally, circular in cross-section. Posterior flange of hind coxa not produced as raised lamella. Metatibial gland present as oval patch of whitish cuticle. Metabasitarsal gland absent. Hind pretarsal claws simple. *Polymorphism*: Monomorphic.

***Male.*** *Head*: Antennae with 13 segments. Clypeus with or without cuticular apron. Parafrontal ridges absent. Torulo-posttorular complex vertical. Maxillary palps 4- or 3-segmented. Labial palps 3- or 2-segmented. Mandibles triangular with teeth or falcate. Ventrolateral margins of head without lamella or ridge extending towards mandibles and beyond carina surrounding occipital foramen. Carina surrounding occipital foramen ventrally absent or present. *Mesosoma*: Pronotal flange not separated from collar by distinct ridge. Notauli present at least anteriorly, very rarely absent. Transverse groove dividing mesopleuron absent or present. Propodeal declivity reduced, without distinct dorsal edge or margin. Metapleural gland opening absent. Propodeal lobes present. *Metasoma*: Petiole anterodorsally immarginate or marginate, dorsolaterally immarginate, and laterally above spiracle immarginate. Helcium in relation to tergosternal Pronotomesopleural suture placed at posttergite and axial. Prora simple, not delimited by carina. Spiracle openings of abdominal segments IV–VI circular. Abdominal segment III more than half size of succeeding segment IV; latter weakly constricted at presegmental portion (uninodal waist). Girdling constriction of segment IV present, i.e. pre- and postsclerites distinct. Cinctus of abdominal segment IV gutter-like, not sculptured. Girdling constriction between pre- and postsclerites of abdominal segments V and VI absent. Abdominal segment IV not conspicuously largest segment. Abdominal sternite VII simple. Abdominal sternite IX distally armed with two spines, with lateral apodemes about as long as medial apodeme, directed anteriorly (towards head). *Genitalia*: Cupula long relative to rest of genital capsule and of approximately equal length on both dorsal and ventral surfaces. Basimere broadly fused to telomere, with no sulcus trace at junction, and ventrally with left and right arms abutting. Telomere not apically expanded, very reduced relative to basimere. Volsella variable. Penisvalva laterally compressed, rounded at apex. *Legs*: Mid tibia with single pectinate spur. Hind tibia with single pectinate spur. Posterior flange of hind coxa not produced as raised lamella. Metatibial gland present as oval patch of whitish cuticle. Metabasitarsal glands absent. Hind pretarsal claws simple. *Wings*: Tegula present, broad, demiovate in shape. Vein C in fore wing present. Pterostigma broad. Abscissa R·f3 present and running toward distal wing margin but not enclosing cell with Rs·f5 or rarely absent. Abscissae Rs·f2–3 absent. Cross-vein 2r-rs present, forming base of ‘free stigmal vein’ (2r-rs&Rs·f4–5) in absence of Rs·f3 and 2rs-m or rarely absent. Abscissae Rs·f4–5 fused in absence of 2rs-m or rarely absent. Abscissa M·f2 in fore wing contiguous with Rs+M or rarely absent. Abscissa M·f4 in fore wing present, reaching wing margin or not, rarely entirely absent. Cross-vein 1m-cu in fore wing present or rarely absent. Cross-vein cu-a in fore wing present, arising from M+Cu and proximal or near M·f1. Vein Cu in fore wing present, with only Cu1 branch prominent or absent past M+Cu. Vein A in fore wing with abscissae A·f1 and A·f2 or only A·f1 present. Vein C in hind wing absent. Vein R in hind wing present, extending past Sc+R but not reaching distal wing margin. Vein Sc+R in hind wing absent or present. Abscissa Rs·f1 in hind wing present, shorter than 1rs-m. Abscissa Rs·f2 in hind wing absent or present, not reaching wing margin. Cross-vein 1rs-m in hind wing fused with M·f1 or absent. Vein M+Cu in hind wing absent or present. Abscissa M·f1 in hind wing absent or present. Abscissa M·f2 in hind wing absent. Cross-vein cu-a in hind wing absent or present. Vein Cu in hind wing absent or present. Vein A in hind wing present with abscissa A·f1 present or absent.

***Gyne.*** At least one dealate gyne specimen with fully developed wing sclerites is known, but ergatoid queens have also been collected (Peter Hawkes pers. comm.).

***Larva.*** Larvae have not been described. Cocoons present.

#### Distribution.

One species of *Eburopone*, *Eburopone wroughtoni*, has been described so far from South Africa and Zimbabwe, but more species are evidently to be found throughout Sub-Saharan Africa, as evidenced by unassociated males and gynes present in collections. Specimens belonging to this group have also been collected in Cameroon and Mozambique, suggesting that *Eburopone* is widely distributed in Africa. This lineage is also represented by a major radiation in Madagascar with dozens of species, none of which has been described.

#### Taxonomy and phylogeny.

*Cerapachys wroughtoni* was originally described by Forel from South Africa and the same author subsequently described Cerapachys wroughtoni var. rhodesiana and *Cerapachys roberti*, both considered junior synonyms of *wroughtoni* by Brown (1975).

The position of *Eburopone* on the doryline tree is uncertain (Brady et al. 2014, Borowiec, in prep.) and the internal phylogeny of the group has never been investigated in detail, although it appears that the Madagascar species are nested within Afrotropical lineages and that the crown group of this genus is very old (Borowiec, in prep.).

#### Biology.

There are no published reports on the biology of this lineage, although field observations suggest that most species are subterranean, have relatively populous colonies, and forage on brood of other ants. Based on several nest samples of undescribed Malagasy species where only larvae or pupae were collected, brood production appears to be synchronized (Brian Fisher pers. comm., author’s observations).

#### Species of *Eburopone*

*Eburopone wroughtoni* (Forel, 1910c): South Africa, **comb. n.**

### Eciton

Taxon classificationAnimaliaHymenopteraFormicidae

Latreille, 1804

= Camptognatha Grey, 1832
= Holopone Santschi, 1925
= Mayromyrmex Ashmead, 1905

#### Type-species.

*Formica hamata*, by subsequent designation of Shuckard, in Swainson and Shuckard, 1840.

*Eciton* comprises the most conspicuous army ants in the New World. The huge colony size combined with epigaeic nesting and foraging habits makes these ants major invertebrate predators and key species of the tropical ecosystems.

#### Diagnosis.

***Worker.*** *Eciton* is recognized by a combination of 12-segmented antennae, propodeal spiracle high on the propodeum, propodeal declivity armed with cuticular tubercles or lamellae, binodal waist, pretarsal claws armed with a tooth and presence of a prominent metatibial gland visible as an elongate patch of whitish or yellowish cuticle on the flexor (inner) surface of tibia. Among New World army ants, *Eciton* is similar to its closest relative *Nomamyrmex*, with which it shares propodeal armament, but workers of all sizes are easily separated by a conspicuous white stripe on inner hind tibiae that is absent in *Nomamyrmex*. *Labidus* species can be distinguished from *Eciton* by their smooth, unarmed propodeum.

***Male.*** The males of *Eciton* possess wing venation characteristic of all the New World army ants (also see under *Cheliomyrmex* male diagnosis). A combination of absence of very long setae approaching femur length on abdomen, apices of penisvalvae without setae, gradually tapering volsellae, and deeply concave dorsal surface of the petiole will distinguish *Eciton* males from all other army ant genera in the New World. The dense tufts of long setae on abdomen are characteristic of *Nomamyrmex*, although *Eciton setigaster* also has long setae abdominal setae; those are not quite as long and abundant as in *Nomamyrmex*, however, not approaching fore femur length. The penisvalvae without setae are also found in *Neivamyrmex* but in that genus the volsellae taper to a sharp point and often turn downwards towards the apex or are forked, not simply gradually narrowing to a blunt apex as in *Eciton*. In addition, *Eciton* males have a very conspicuously excavated dorsal surface of the petiole, which is usually more flattened in *Neivamyrmex*.

#### Description.

***Worker.*** *Head*: Antennae with 12 segments. Apical antennal segment not enlarged, not broader and longer than two preceding segments combined. Clypeus with cuticular apron. Lateroclypeal teeth absent. Parafrontal ridges reduced. Torulo-posttorular complex vertical. Antennal scrobes absent. Labrum with median notch or concavity. Proximal face of stipes projecting beyond inner margin of sclerite, concealing prementum when mouthparts fully closed. Maxillary palps 2-segmented. Labial palps 3-segmented. Mandibles polymorphic, from triangular with teeth through falcate with teeth on masticatory margin, to falcate without teeth on elongated masticatory margin. Eyes present, appearing as single large and convex ommatidium, in reality composed from fused ommatidia. Ocelli absent. Head capsule with differentiated vertical posterior surface above occipital foramen. Ventrolateral margins of head without lamella or ridge extending towards mandibles and beyond carina surrounding occipital foramen. Posterior head corners dorsolaterally immarginate. Carina surrounding occipital foramen ventrally absent. *Mesosoma*: Pronotal flange not separated from collar by distinct ridge. Promesonotal connection with Pronotomesopleural suture completely fused. Pronotomesopleural suture completely fused. Mesometapleural groove not impressed. Transverse groove dividing mesopleuron absent. Pleural endophragmal pit concavity present. Mesosoma dorsolaterally immarginate. Metanotal depression or groove on mesosoma present. Propodeal spiracle situated high on sclerite. Propodeal declivity with distinct dorsal edge or margin and in form of narrow strip. Metapleural gland with bulla visible through cuticle. Propodeal lobes present, short. *Metasoma*: Petiole anterodorsally immarginate or marginate, dorsolaterally immarginate, and laterally above spiracle immarginate. Helcium in relation to tergosternal Pronotomesopleural suture placed at posttergite and axial. Prora narrowed into anteriorly directed spine. Spiracle openings of abdominal segments IV–VI slit-shaped or oval in small workers. Abdominal segment III anterodorsally immarginate and dorsolaterally immarginate. Abdominal segment III about half size of succeeding segment IV, which is strongly constricted at presegmental portion (binodal waist). Girdling constriction of segment IV present, i.e. pre- and postsclerites distinct. Cinctus of abdominal segment IV a gradual concavity, not gutter-like. Abdominal segment IV conspicuously largest segment. Abdominal tergite IV not folding over sternite, and anterior portions of sternite and tergite equally well visible in lateral view. Girdling constriction between pre- and posttergites of abdominal segments V and VI absent. Girdling constriction between pre- and poststernites of abdominal segments V and VI absent. Pygidium small, reduced to narrow strip, without impressed medial field and simple, not armed with cuticular spines or modified setae. Hypopygium unarmed. *Legs*: Mid tibia with single pectinate spur. Hind tibia with single pectinate spur. Hind basitarsus not widening distally, circular in cross-section. Posterior flange of hind coxa not produced as raised lamella. Metatibial gland present as patch of whitish cuticle occupying at least half of tibia length. Metabasitarsal gland absent. Hind pretarsal claws each armed with a tooth. *Polymorphism*: Highly polymorphic.

***Male.*** *Head*: Antennae with 13 segments. Clypeus without cuticular apron. Parafrontal ridges absent. Torulo-posttorular complex vertical. Maxillary palps 2-segmented. Labial palps 2-segmented. Mandibles falcate. Ventrolateral margins of head without lamella or ridge extending towards mandibles and beyond carina surrounding occipital foramen. Carina surrounding occipital foramen ventrally absent. *Mesosoma*: Pronotal flange not separated from collar by distinct ridge. Notauli absent. Transverse groove dividing mesopleuron absent. Propodeal declivity reduced, without distinct dorsal edge or margin. Metapleural gland opening absent. Propodeal lobes present. *Metasoma*: Petiole anterodorsally immarginate, dorsolaterally immarginate, and laterally above spiracle immarginate. Helcium in relation to tergosternal Pronotomesopleural suture placed at Pronotomesopleural suture and axial. Prora forming a simple, wide U-shaped margin not delimited by ridge. Spiracle openings of abdominal segments IV–VI slit-shaped. Abdominal segment III more than half size of succeeding segment IV; latter weakly constricted at presegmental portion (uninodal waist). Girdling constriction of segment IV absent, i.e. pre- and postsclerites indistinct. Cinctus of abdominal segment IV absent, not impressed. Girdling constriction between pre- and postsclerites of abdominal segments V and VI absent. Abdominal segment IV not conspicuously largest segment. Abdominal sternite VII simple. Abdominal sternite IX distally armed with two spines, with lateral apodemes longer than much reduced medial apodeme, directed anteriorly (towards head). *Genitalia*: Cupula very long, nearing or surpassing length of rest of genital capsule and of approximately equal length on both dorsal and ventral surfaces. Basimere narrowly fused to telomere, with Pronotomesopleural suture modified into membrane at junction, and ventrally with left and right arms abutting. Telomere expanded at apex. Volsella laterally flattened, narrow and tapered towards tip. Penisvalva hook-like, strongly curved ventrally. *Legs*: Mid tibia with single pectinate spur. Hind tibia with single pectinate spur. Posterior flange of hind coxa not produced as raised lamella. Metatibial gland absent. Metabasitarsal glands absent. Hind pretarsal claws each armed with a tooth. *Wings*: Tegula present, broad, demiovate in shape. Vein C in fore wing present. Pterostigma narrow. Abscissa R·f3 present, running toward distal wing margin and enclosing cell with Rs·f5. Abscissae Rs·f2–3 present, connecting with Rs+M&M·f2. Cross-vein 2r-rs present, differentiated from Rs·f4 by presence of Rs·f2–3. Abscissae Rs·f4–5 differentiated into Rs·f4 and Rs·f5 by 2rs-m. Abscissa M·f2 in fore wing present, separated from Rs+M by Rs·f2. Abscissa M·f4 in fore wing present, reaching wing margin. Cross-vein 1m-cu in fore wing present. Cross-vein cu-a in fore wing present, arising from Cu and distal to, at or near M·f1. Vein Cu in fore wing present, with both branches Cu1 and Cu2. Vein A in fore wing with abscissae A·f1 and A·f2 present. Vein C in hind wing absent. Vein R in hind wing present, reaching distal wing margin. Vein Sc+R in hind wing present. Abscissa Rs·f1 in hind wing present, shorter than 1rs-m. Abscissa Rs·f2 in hind wing present, reaching wing margin. Cross-vein 1rs-m in hind wing fused with M·f1. Vein M+Cu in hind wing present. Abscissa M·f1 in hind wing present. Abscissa M·f2 in hind wing present. Cross-vein cu-a in hind wing present. Vein Cu in hind wing present. Vein A in hind wing with abscissae A·f1 and A·f2 present.

***Gyne.*** Dichthadiiform, with eyes but no ocelli (see e.g. Wheeler 1921, 1925b, Borgmeier 1958). See Hölldobler (2016) for a description of queen exocrine glands in *Eciton*.

***Larva.*** Larvae of several *Eciton* species have been described by Wheeler (1943) and Wheeler and Wheeler (1964b, 1984). Cocoons are present.

#### Distribution.

From northern Mexico to northern Argentina.

#### Taxonomy and phylogeny.

*Eciton* is the sister lineage to *Nomamyrmex* (Brady et al. 2014, Borowiec, in prep.). An effort to infer the internal phylogeny is currently under way (Daniel Kronauer, Max Winston pers. comm.).

#### Biology.

*Eciton* is the best studied lineage of the dorylines, owing to the lifetime efforts by pioneers of army ant biology, including Thomas Schneirla, Thomas Borgmeier and Carl Rettenmeyer.

Among the twelve described species, *Eciton burchellii* has attracted the most attention, followed by *Eciton hamatum*, although most species have been at least briefly observed in the field. Most accounts of *Eciton* biology are based on the two well-known species.

The literature on *Eciton* is vast, and it is impossible to cite all of the even more significant original contributions. Good overviews of *Eciton* biology can be found in Rettenmeyer (1963), Schneirla (1971), Telles Da Silva (1977a, 1977b), Rettenmeyer et al. (1983) and Gotwald (1982, 1995). The account below is based on these sources, unless noted otherwise.

The life of an *Eciton* colony can be summarized as follows. The colony alternates between the so-called statary and nomadic phases. The cycles are understood to be regulated by brood development rather than an endogenous rhythm in adult ants. During the statary phase a single queen is laying eggs and the brood inside the nest consists of pupae and eggs; foraging does not happen every day and raids are relatively much less intensive. There are no emigrations to new nesting sites. In the nomadic phase, the queen stops producing new eggs and her abdomen contracts; the colony contains many developing larvae that need nutrition. Raids and emigrations usually occur every day. In *Eciton burchellii*, the statary phase lasts on average 20 days and the nomadic phase is 14 days long.

A mature colony containing a single mated queen will eventually produce up to six virgin queens and hundreds to thousands of males, depending on the species. Usually the queen that emerges first leaves the colony with workers clustered around her. She has the best chance to survive and lead the fissioning part of the nest. About half of the workers eventually leave with the virgin queen. Because the colony is divided into approximately equal halves, the workers represent a substantial part of the reproductive investment. This explains the highly male-biased sex ratio, also typical of other social insects with colony fission (Pamilo 1991). The older, mated queen emigrates together with brood while the virgin queen disperses with the remaining workers. Shortly after the fission, the colony will accept multiple males that enter the bivouac. The males must first be accepted by the workers and they lose their wings before mating. Each male can mate only once, but *Eciton burchellii* queens are known to mate with a dozen males on average, this mating frequency being among the highest in eusocial Hymenoptera (Kronauer et al. 2006).

Although mature colonies have been observed to occasionally admit new males, there is strong evidence that all of the mating occurs when the queen is young (Kronauer and Boomsma 2007a). A fertilized queen can produce up to 225,000 eggs per 35-day cycle and 14 million eggs during her lifetime (Schneirla 1971, Kronauer and Boomsma 2007a).

Colony structure and nesting behavior has been studied in some detail in several species. Temporary nests are made up of bodies of workers, hanging together by their legs from a supporting structure. These bivouacs can be found in a variety of microhabitats, but common nesting sites include hollow logs, spaces between buttresses of large trees, and empty soil cavities such as abandoned mammal burrows. *Eciton* species vary in their preferences for bivouac sites, with *Eciton burchellii* and *Eciton hamatum* nesting in exposed sites, the former often hanging above ground without touching the surface. *Eciton dulcius* and *Eciton mexicanum* are known to nest only in underground cavities, and *Eciton vagans* is intermediate, sometimes found in relatively exposed sites, but often nesting under logs and in rock crevices.

Colony size estimates vary widely and reliable data exists only for *Eciton burchellii* and *Eciton hamatum*. Rettenmeyer estimated that mature colonies of *Eciton burchellii* contain from 300,000 to 700,000 worker ants before fission and 100,000 to 500,000 for *Eciton hamatum*. Colony densities have been estimated in several localities for *Eciton burchellii*, ranging from 3.5 colonies per 100 ha on Barro Colorado Island, Panama, to 11 colonies in Corcovado, Costa Rica (Franks 1982, Vidal-Riggs and Chaves-Campos 2008).

Foraging behavior in *Eciton* has been studied extensively. Workers forage either mostly above ground (*Eciton burchellii*, *Eciton hamatum*, *Eciton rapax*) or with some part of the raid unfolding underground. The latter mode has been reported for most other species, but the paucity of data precludes comparisons. The surface foragers also ascend vegetation and are capable of foraging arboreally. Ant brood constitutes a major portion of *Eciton* prey, although other arthropods, especially other social insects, are often targeted. *Eciton burchellii* is the most generalist predator, still hunting ants, but also actively preying on a variety of other arthropods and even opportunistically killing small vertebrates.

At the beginning of a raid, foragers emerge from the nest and gradually assemble into narrow trails that often branch and extend for up to 100 m (200 m in *Eciton rapax*) from the bivouac. These columns are typical of most species except for *Eciton burchellii* where the front of each raid progresses as a ‘swarm’, a continuous front up to 10 m wide. Group foraging is a self-organizing process with no scouts to guide the ants to a particular source of food, but the workers do follow trail pheromones produced by sternal glands (Billen and Gobin 1996). The progress of an advancing ant column can be rapid, and was estimated at up to 20 m per hour in *Eciton hamatum*. A remarkable adaptation for improving the efficiency of foraging is found in *Eciton burchellii*. Workers of this species have the ability to form living plugs over gaps in the substrate, significantly smoothening the surface and allowing faster movement of fellow foragers (Powell and Franks 2007). *Eciton* foragers are also extremely efficient at cooperative transport of prey. As a group they are able to carry more than a combined mass of what they could transport individually. Although cooperative transport has been documented for many ant species, this type of ‘superefficient’ transport is rare (Czaczkes and Ratnieks 2013, McCreery and Breed 2014). *Eciton* raids also establish caches for temporary storage of prey along the trail. In the species foraging in columns there can be more than one trail radiating from a bivouac at any given time, whereas an *Eciton burchellii* colony conducts one swarm raid at a time. The direction of raids of *Eciton burchellii* during the statary phase has been also shown to systematically change each day, apparently minimizing the overlap of foraging area (Willson et al. 2011).

During the nomadic phase, *Eciton* conducts raids every day and at some point these raids transition into an exodus of workers and finally an emigration of the entire colony. The emigration doesn not always follow the same route as the day’s raid and can be sustained by agitated returning foragers carrying booty past the bivouac. Other workers follow these foragers and eventually start to carry brood away from the bivouac. When the transport of brood is well advanced, myrmecophiles appear in the emigration column and the queen passes, surrounded by an entourage of workers. The duration of emigration is dependent on the colony size and species, and distances covered vary greatly as well; Schneirla (1971) reported emigration trail lengths from 100 to 450 m in *Eciton hamatum*.

*Eciton* colonies have an extraordinarily rich associate fauna and over 300 species, from mites to birds, have been recorded to depend on *Eciton burchellii* (Kistner 1982, Rettenmeyer et al. 2011). Remarkably, as many as 29 species are birds that rely almost exclusively on insect prey flushed out of the leaf litter by *Eciton* raids. This behavior evolved multiple times, and obligate ‘antbirds’ are found in the families Thamnophilidae, Formicariidae, and Furnariidae (Willis and Oniki 1978, Rettenmeyer et al. 2011). The bird droppings in turn attract many butterflies, especially skippers (family Hesperiidae; DeVries et al. 2009). A multitude of fly, wasp, beetle, and other arthropod species are found preying on the insects fleeing from a raid or scavenging in the refuse piles of *Eciton* bivouacs. It seems that relatively very few of these are predators or parasites of the ants themselves, although rove beetles in the genus *Tetradonia* are known to kill and feed on injured workers. Within the colony, some mites are known to suck on the ant hemolymph. *Macrocheles rettenmeyeri* is a parasitic mite found with *Eciton dulcius*. It is remarkable because it functionally replaces the ant’s distal tarsal segment. The mite attaches itself to the membrane of hind leg pulvilli and its curved hind legs serve as the ant’s claws without affecting the host’s behavior. As documented for the staphylinid genus *Vatesus*, some myrmecophiles synchronize their life cycle with the nomadic and statary phases of their host *Eciton* colonies (von Beeren et al. 2016).

*Eciton* species are important predators of ants and other social insects and elicit a wide range of responses from its prey. Chadab-Crepet and Rettenmeyer (1982) studied behavior of social wasps affected by army ant raids and found that many species exhibit coordinated alarm response allowing the adult wasps to survive and reestablish the nest later. Dejean et al. (2013) review the antipredatory behaviors of ants to army ants in general and to *Eciton burchellii* and *Eciton hamatum* in particular. They show that many species evacuate the nest in the face of an *Eciton* raid. This behavior ranges from well-organized evacuations starting in advance of the attack and resulting in no casualties on either side to cases where a substantial portion of brood is lost by the defending species. *Paratrechina longicornis* is an example of the former, while the less efficient *Pachycondyla harpax* represents the latter. Some species of ants are ignored by *Eciton*, particularly the enormous colonies of leaf-cutting *Atta*, and some can have a repellent effect, like the antplant-associated *Pseudomyrmex ferrugineus* and *Azteca alfari*. A few species, such as the arboreal *Azteca chartifex* and *Dolichoderus bispinosus*, manage to resist *Eciton* raids by attacking the raiding army ants (Dejean et al. 2013).

#### Species of *Eciton*

*Eciton burchellii* (Westwood, 1842): Brazil

*Eciton burchellii cupiens* Santschi, 1923a: French Guiana

*Eciton burchellii foreli* Mayr, 1886b: Panama

*Eciton burchellii parvispinum* Forel, 1899: Guatemala

*Eciton burchellii urichi* Forel, 1899: Trinidad and Tobago

*Eciton drepanophorum* Smith, F., 1858: Brazil

*Eciton dulcium* Forel, 1912a: Brazil

*Eciton dulcium crassinode* Borgmeier, 1955: Panama

*Eciton hamatum* (Fabricius, 1782): French Guiana

*Eciton jansoni* Forel, 1912a: Nicaragua

*Eciton lucanoides* Emery, 1894: Peru

*Eciton lucanoides conquistador* Weber, 1949b: Panama

*Eciton mexicanum* Roger, 1863: Mexico

*Eciton mexicanum argentinum* Borgmeier, 1955: Argentina

*Eciton mexicanum goianum* Borgmeier, 1955: Brazil

*Eciton mexicanum latidens* Santschi, 1911b: French Guiana

*Eciton mexicanum moralum* Santschi, 1923c: French Guiana

*Eciton mexicanum panamense* Borgmeier, 1955: Panama

*Eciton quadriglume* (Haliday, 1836): Brazil

*Eciton rapax* Smith, F., 1855: Brazil

*Eciton setigaster* Borgmeier, 1953: Brazil

*Eciton uncinatum* Borgmeier, 1953: Ecuador

*Eciton vagans* (Olivier, 1792): French Guiana

*Eciton vagans allognathum* Borgmeier, 1955: Venezuela

*Eciton vagans angustatum* Roger, 1863: Mexico

*Eciton vagans dispar* Borgmeier, 1955: Brazil

*Eciton vagans dubitatum* Emery, 1896b: Paraguay

*Eciton vagans fur* Borgmeier, 1955: Brazil

*Eciton vagans mutatum* Borgmeier, 1955: Costa Rica

### Eusphinctus

Taxon classificationAnimaliaHymenopteraFormicidae

Emery, 1893a gen. rev.

#### Type-species.

*Eusphinctus furcatus*, by monotypy.

*Eusphinctus* is a species-poor South East Asian genus with apparently small colonies.

#### Diagnosis.

***Worker.*** *Eusphinctus* workers belong to dorylines with conspicuous gastral constrictions visible between abdominal segments IV, V, and VI. This morphology is also seen in *Aenictogiton*, certain species of *Leptanilloides*, *Sphinctomyrmex*, and *Zasphinctus. Eusphinctus* is unique in the combination of propodeal spiracle situated low on the sclerite and propodeal lobes present, a large pygidium armed with modified setae, pronotomesopleural Pronotomesopleural suture present, and cinctus of abdominal segment IV simple and not cross-ribbed. This genus is thus far known only from India, Bangladesh, Myanmar, and Thailand and the only lineage with gastral constriction that is currently known to overlap with it is *Zasphinctus*. In *Zasphinctus* the pronotomesopleural Pronotomesopleural suture is fused, in *Aenictogiton* the propodeal spiracle is positioned high and there are no propodeal lobes, *Leptanilloides* has a reduced and unarmed pygidium, and the neotropical *Sphinctomyrmex* has 12-segmented antennae and the cinctus on abdominal segment IV smooth. The relative proportions of abdominal segments are also different, with segments IV, V, and VI being about equal in size in *Sphinctomyrmex* and *Zasphinctus*, while in *Eusphinctus* segment IV is the largest of the three.

***Male.*** The male of *Eusphinctus* can be recognized by a combination of 12-segmented antennae, pronounced propodeal lobes, narrow axial helcium, conspicuous constrictions present between abdominal segments IV, V, and VI, costal (C) cell present in the fore wing, submarginal cell closed by Rs·f2–3, R·f3 present past pterostigma, and marginal cell open. Abdominal sternite IX (subgenital plate) in *Eusphinctus* gradually tapers caudad and has simple, straight spines directed posteriorly. *Sphinctomyrmex* and *Zasphinctus* also have constrictions between abdominal segments IV, V, and VI but the former always has 13-segmented antennae and the latter lacks veins C and R·f3 in the fore wing.

#### Description.

***Worker.*** *Head*: Antennae with 11 segments. Apical antennal segment moderately enlarged, broader than and about equal in length to two preceding segments combined. Clypeus without cuticular apron. Lateroclypeal teeth present. Parafrontal ridges reduced. Torulo-posttorular complex vertical. Antennal scrobes absent. Labrum unknown. Proximal face of stipes unknown. Maxillary palps unknown. Labial palps unknown. Mandibles triangular, with teeth. Eyes present, composed of fewer than five ommatidia. Ocelli absent. Head capsule with differentiated vertical posterior surface above occipital foramen. Ventrolateral margins of head without lamella or ridge extending towards mandibles and beyond carina surrounding occipital foramen. Posterior head corners dorsolaterally immarginate. Carina surrounding occipital foramen ventrally present. *Mesosoma*: Pronotal flange separated from collar by distinct ridge. Promesonotal connection with Pronotomesopleural suture completely fused. Pronotomesopleural suture visible, unfused up to notal surface. Mesometapleural groove weakly impressed. Transverse groove dividing mesopleuron present. Pleural endophragmal pit concavity present. Mesosoma dorsolaterally immarginate. Metanotal depression or groove on mesosoma absent. Propodeal spiracle situated low on sclerite. Propodeal declivity with distinct dorsal edge or margin and rectangular in posterior view. Metapleural gland without bulla visible through cuticle. Propodeal lobes present, well developed. *Metasoma*: Petiole anterodorsally immarginate, dorsolaterally immarginate, and laterally above spiracle marginate. Helcium in relation to tergosternal Pronotomesopleural suture placed at posttergite and axial. Prora forming a simple U-shaped margin. Spiracle openings of abdominal segments IV–VI circular. Abdominal segment III anterodorsally immarginate and dorsolaterally immarginate. Abdominal segment III more than half size of succeeding segment IV, which is weakly constricted at presegmental portion (uninodal waist). Girdling constriction of segment IV present, i.e. pre- and postsclerites distinct. Cinctus of abdominal segment IV gutter-like, not sculptured. Abdominal segment IV not conspicuously largest segment. Abdominal tergite IV not folding over sternite, and anterior portions of sternite and tergite equally well visible in lateral view. Girdling constriction between pre- and posttergites of abdominal segments V and VI present. Girdling constriction between pre- and poststernites of abdominal segments V and VI present. Pygidium large, with impressed medial field, armed with modified setae, and deeply notched at apex. Hypopygium unarmed. *Legs*: Mid tibia with single pectinate spur. Hind tibia with single pectinate spur. Hind basitarsus not widening distally, circular in cross-section. Posterior flange of hind coxa not produced as raised lamella. Metatibial gland absent. Metabasitarsal gland absent. Hind pretarsal claws simple. *Polymorphism*: Monomorphic.

***Male.*** *Head*: Antennae with 12 segments. Clypeus with cuticular apron. Parafrontal ridges absent. Torulo-posttorular complex vertical. Maxillary palps unknown. Labial palps unknown. Mandibles triangular, edentate. Ventrolateral margins of head without lamella or ridge extending towards mandibles and beyond carina surrounding occipital foramen. Carina surrounding occipital foramen ventrally present. *Mesosoma*: Pronotal flange not separated from collar by distinct ridge. Notauli present. Transverse groove dividing mesopleuron present. Propodeal declivity with distinct dorsal edge or margin. Metapleural gland opening absent. Propodeal lobes present. *Metasoma*: Petiole anterodorsally immarginate, dorsolaterally immarginate, and laterally above spiracle marginate. Helcium in relation to tergosternal Pronotomesopleural suture placed at posttergite and supraaxial. Prora forming a V-shaped protrusion. Spiracle openings of abdominal segments IV–VI circular. Abdominal segment III more than half size of succeeding segment IV; latter weakly constricted at presegmental portion (uninodal waist). Girdling constriction of segment IV present, i.e. pre- and postsclerites distinct. Cinctus of abdominal segment IV gutter-like, not sculptured. Girdling constriction between pre- and postsclerites of abdominal segments V and VI present. Abdominal segment IV not conspicuously largest segment. Abdominal sternite VII simple. Abdominal sternite IX distally armed with two spines, with lateral apodemes about as long as medial apodeme, directed anteriorly (towards head). *Genitalia*: Cupula long relative to rest of genital capsule and of approximately equal length on both dorsal and ventral surfaces. Basimere broadly fused to telomere, with sulcus discernable at junction, and ventrally with left and right arms abutting. Telomere gradually tapering toward apex. Volsella narrow, hook-shaped. Penisvalva laterally compressed, rounded at apex. *Legs*: Mid tibia with single pectinate spur. Hind tibia with single pectinate spur. Posterior flange of hind coxa not produced as raised lamella. Metatibial gland absent. Metabasitarsal glands absent. Hind pretarsal claws simple. *Wings*: Tegula present, broad, demiovate in shape. Vein C in fore wing present. Pterostigma broad. Abscissa R·f3 absent. Abscissae Rs·f2–3 present, disconnected from Rs+M. Cross-vein 2r-rs present, differentiated from Rs·f4 by presence of Rs·f2–3. Abscissae Rs·f4–5 present, fused in absence of 2rs-m. Abscissa M·f2 in fore wing contiguous with Rs+M. Abscissa M·f4 in fore wing present, reaching wing margin. Cross-vein 1m-cu in fore wing present. Cross-vein cu-a in fore wing present, arising from Cu and distal to, at or near M·f1. Vein Cu in fore wing present, with only Cu1 branch prominent. Vein A in fore wing with abscissae A·f1 and A·f2 present. Vein C in hind wing absent. Vein R in hind wing absent. Vein Sc+R in hind wing present. Abscissa Rs·f1 in hind wing present, shorter than 1rs-m. Abscissa Rs·f2 in hind wing absent. Cross-vein 1rs-m in hind wing fused with M·f1. Vein M+Cu in hind wing present. Abscissa M·f1 in hind wing present. Abscissa M·f2 in hind wing present. Cross-vein cu-a in hind wing present. Vein Cu in hind wing present. Vein A in hind wing with abscissae A·f1 and A·f2 present.

***Gyne.*** Ergatoid, ‘scarcely different in size from the workers’ (Brown 1975) except slightly larger eyes and wider abdominal segment II (petiole). Presence of ocelli unknown.

***Larva.*** Larva not known. Presence of cocoons unknown.

#### Distribution.

Indomalayan, known from India, Bangladesh, Myanmar, and Thailand.

#### Taxonomy and phylogeny.

Based on morphological and molecular evidence (Borowiec in prep.), I revive *Eusphinctus* from synonymy with *Sphinctomyrmex*. The taxonomic history of taxa classified under *Sphinctomyrmex* is somewhat complicated. Detailed discussions can be found in Wheeler (1918) and Brown (1975), and I only briefly recount the history of taxonomic changes to provide a background for an arrangement proposed here. The genus *Sphinctomyrmex* was established by Mayr (1866b) based on a single dealate gyne specimen from Brazil. Mayr emphasized the prominent constrictions between abdominal segments present in the specimen in the description, which gave inspiration for the name. Later, Emery (1893a) described a new genus from Myanmar, *Eusphinctus*, for an ant with similar constrictions. Other species from the Old World followed, described under either name. Wheeler in 1918 decided (after André 1905) to reserve *Sphinctomyrmex* for all New World forms and further split *Eusphinctus* into three subgenera, *Eusphinctus* *s. str.*, *Nothosphinctus*, and *Zasphinctus*, according to various combinations of the gyne morphology, number of antennal segments (11 or 12), and presence or absence of eyes in the worker. Brown (1975) discussed the taxonomic history of the genus and all of Wheeler’s characters in detail. He pointed out that the characters used to differentiate these vary and the combinations enumerated by Wheeler do not hold as generic diagnoses with newly discovered species. Brown thus concluded that it was most sensible to synonymize all the genus-level names under *Sphinctomyrmex* until more evidence, particularly from male morphology, was gathered. However, he allowed for a possibility that two species, *Eusphinctus furcatus* and *Eusphinctus taylori* indeed deserved a separate generic status (Brown 1975).

Here I propose a new classification where all the New World species are retained in *Sphinctomyrmex*, while most of the described Old World forms are relegated to *Zasphinctus*. The two remaining above mentioned Old World species are separated from *Zasphinctus* as *Eusphinctus*. Molecular data shows that all three genera arose independently on the dorylomorph tree (see Figure 1; Borowiec, in prep.). Despite sharing characteristic gastral constrictions, these lineages are also discrete in worker and male morphology (see diagnosis above). Both morphology and molecules support the notion that abdominal constrictions have been independently derived several times in the Dorylinae: in *Eusphinctus*, *Sphinctomyrmex*, and *Zasphinctus* and also in *Aenictogiton* and some *Leptanilloides*.

*Eusphinctus* belongs to a clade that also includes *Ooceraea* and *Syscia* (Borowiec, in prep.). The members of this clade share the universal reduction in the number of antennal segments, from 12 to 11 or fewer in the worker caste and from 13 to 12 or fewer in males.

There are only two species of *Eusphinctus*, both quite similar, and Brown (1975: 75) allowed the possibility that specimens described as *Eusphinctus taylori* may be just small workers of *Eusphinctus furcatus*, but he decided not to synonymize them until more specimens are available.

#### Biology.

A. B. Soans and W. L. Brown collected two colonies of *Eusphinctus furcatus* in Kottiyoor, Kerala, India. One was located in leaf litter near a rotting log and the other one was found under a stone in a shaded creek bottom. There were about 50 workers in each of the observed nests, and one colony contained two ergatoid gynes (Brown 1975).

#### Species of *Eusphinctus*

*Eusphinctus furcatus* Emery, 1893a: Myanmar, **comb. rev.**

*Eusphinctus taylori* (Forel, 1900b): Bangladesh, India, **comb. n.**

### Labidus

Taxon classificationAnimaliaHymenopteraFormicidae

Jurine, 1807

= Nycteresia Roger, 1861
= Pseudodichthadia André, 1885

#### Type-species.

*Labidus latreillii* (junior synonym of *Formica coeca*), by monotypy.

With seven described species, *Labidus* is a relatively small but widely distributed genus. Its members are more generalized predators than most other New World army ants and may have the greatest overall ecological impact due to high densities.

#### Diagnosis.

***Worker.*** *Labidus* workers are easily recognized by a combination of spiracle positioned high on the propodeum, 12-segmented antennae, propodeum not armed with spines or cuticular lamellae, short propodeal lobes, two-segmented waist, metatibial gland present, and pretarsal claws with a tooth. *Labidus* belongs to New World army ants with an unarmed propodeum and it could only be confused with *Cheliomyrmex* and certain larger species of *Neivamyrmex*. The former have one-segmented waist, and the latter always lack teeth on pretarsal claws.

***Male.*** *Labidus* males have the army ant habitus with abdominal segment III much larger than the preceding segment II (petiole), and head small relative to mesosoma. See discussion under *Cheliomyrmex* for characters differentiating New World army ant males from those of Old World *Aenictus*, *Aenictogiton*, and *Dorylus*. Among New World army ants, *Labidus* possesses the following unique character combination: no conspicuous tufts of long setae on gaster, apices of penisvalvae with setae, abdominal sternite IX (subgenital plate) with two spines, and hind basitarsus with a groove that accommodates the tibial spur. The lack of long gastral setae differentiates *Labidus* from *Nomamyrmex*, the apices of penisvalvae are hairy in *Eciton* and *Neivamyrmex*, and in *Cheliomyrmex* there are four spines on the abdominal sternite IX and hind basitarsus has no oblique grooves.

#### Description.

***Worker.*** *Head*: Antennae with 12 segments. Apical antennal segment not enlarged, not broader and longer than two preceding segments combined. Clypeus with or without cuticular apron. Lateroclypeal teeth absent. Parafrontal ridges reduced. Torulo-posttorular complex vertical. Antennal scrobes absent. Labrum with median notch or concavity. Proximal face of stipes projecting beyond inner margin of sclerite, concealing prementum when mouthparts fully closed. Maxillary palps 2-segmented. Labial palps 3-segmented. Mandibles polymorphic, from triangular with teeth to falcate with teeth on elongated masticatory margin. Eyes present, composed of seemingly single large ommatidium, in reality composed from multiple fused ommatidia. Ocelli absent. Head capsule with differentiated vertical posterior surface above occipital foramen. Ventrolateral margins of head without lamella or ridge extending towards mandibles and beyond carina surrounding occipital foramen. Posterior head corners dorsolaterally immarginate. Carina surrounding occipital foramen ventrally absent. *Mesosoma*: Pronotal flange not separated from collar by distinct ridge. Promesonotal connection with Pronotomesopleural suture completely fused. Pronotomesopleural suture completely fused. Mesometapleural groove not impressed. Transverse groove dividing mesopleuron absent. Pleural endophragmal pit concavity present. Mesosoma dorsolaterally immarginate. Metanotal depression or groove on mesosoma present. Propodeal spiracle situated high on sclerite. Propodeal declivity without distinct dorsal edge or margin and rectangular in posterior view. Metapleural gland with bulla visible through cuticle. Propodeal lobes absent or very short. *Metasoma*: Petiole anterodorsally marginate with carina low on anterior face, dorsolaterally immarginate, and laterally above spiracle immarginate. Helcium in relation to tergosternal Pronotomesopleural suture placed at posttergite and axial. Prora forming a V-shaped protrusion. Spiracle openings of abdominal segments IV–VI oval. Abdominal segment III anterodorsally immarginate and dorsolaterally immarginate. Abdominal segment III about half size of succeeding segment IV, which is strongly constricted at presegmental portion (binodal waist). Girdling constriction of segment IV present, i.e. pre- and postsclerites distinct. Cinctus of abdominal segment IV gutter-like and sculptured but not cross-ribbed. Abdominal segment IV conspicuously largest segment. Abdominal tergite IV not folding over sternite, and anterior portions of sternite and tergite equally well visible in lateral view. Girdling constriction between pre- and posttergites of abdominal segments V and VI absent. Girdling constriction between pre- and poststernites of abdominal segments V and VI absent. Pygidium small, reduced to narrow strip, without impressed medial field and armed with modified setae. Hypopygium unarmed. *Legs*: Mid tibia with single pectinate spur. Hind tibia with single pectinate spur. Hind basitarsus not widening distally, circular in cross-section. Posterior flange of hind coxa not produced as raised lamella. Metatibial gland present as patch of whitish cuticle occupying at least half of tibia length. Metabasitarsal gland absent. Hind pretarsal claws each armed with a tooth. *Polymorphism*: Highly polymorphic.

***Male.*** *Head*: Antennae with 13 segments. Clypeus without cuticular apron. Parafrontal ridges absent. Torulo-posttorular complex vertical. Maxillary palps 2-segmented. Labial palps 2-segmented. Mandibles falcate. Ventrolateral margins of head without lamella or ridge extending towards mandibles and beyond carina surrounding occipital foramen. Carina surrounding occipital foramen ventrally absent. *Mesosoma*: Pronotal flange not separated from collar by distinct ridge. Notauli absent. Transverse groove dividing mesopleuron absent. Propodeal declivity reduced, without distinct dorsal edge or margin. Metapleural gland opening absent. Propodeal lobes present. *Metasoma*: Petiole anterodorsally immarginate, dorsolaterally immarginate, and laterally above spiracle immarginate. Helcium in relation to tergosternal Pronotomesopleural suture placed at Pronotomesopleural suture and axial. Prora forming a simple U-shaped margin or a broad cuticular lip, not delimited by carina; central protuberance may be present. Spiracle openings of abdominal segments IV–VI slit-shaped. Abdominal segment III more than half size of succeeding segment IV; latter weakly constricted at presegmental portion (uninodal waist). Girdling constriction of segment IV absent, i.e. pre- and postsclerites indistinct. Cinctus of abdominal segment IV absent, not impressed. Girdling constriction between pre- and postsclerites of abdominal segments V and VI absent. Abdominal segment IV not conspicuously largest segment. Abdominal sternite VII simple. Abdominal sternite IX distally armed with two spines, with lateral apodemes longer than much reduced medial apodeme, directed anteriorly (towards head). *Genitalia*: Cupula very long, nearing or surpassing length of rest of genital capsule and of approximately equal length on both dorsal and ventral surfaces. Basimere narrowly fused to telomere, with sulcus discernable at junction, and ventrally with left and right arms abutting. Telomere expanded at apex. Volsella laterally flattened, narrow and tapered towards tip. Penisvalva not flattened at apex, expanded. *Legs*: Mid tibia with single pectinate spur. Hind tibia with single pectinate spur. Posterior flange of hind coxa not produced as raised lamella. Metatibial gland absent. Metabasitarsal glands absent. Hind pretarsal claws each armed with a tooth. *Wings*: Tegula present, broad, demiovate in shape. Vein C in fore wing present. Pterostigma narrow. Abscissa R·f3 present, running toward distal wing margin and enclosing cell with Rs·f5. Abscissae Rs·f2–3 present, connecting with Rs+M&M·f2. Cross-vein 2r-rs present, differentiated from Rs·f4 by presence of Rs·f2–3. Abscissae Rs·f4–5 differentiated into Rs·f4 and Rs·f5 by 2rs-m. Abscissa M·f2 in fore wing present, separated from Rs+M by Rs·f2. Abscissa M·f4 in fore wing present, reaching wing margin. Cross-vein 1m-cu in fore wing present. Cross-vein cu-a in fore wing present, arising from Cu and distal to, at or near M·f1. Vein Cu in fore wing present, with both branches Cu1 and Cu2. Vein A in fore wing with abscissae A·f1 and A·f2 present. Vein C in hind wing present. Vein R in hind wing present, reaching distal wing margin. Vein Sc+R in hind wing present. Abscissa Rs·f1 in hind wing present, shorter than 1rs-m. Abscissa Rs·f2 in hind wing present, reaching wing margin. Cross-vein 1rs-m in hind wing fused with M·f1. Vein M+Cu in hind wing present. Abscissa M·f1 in hind wing present. Abscissa M·f2 in hind wing present. Cross-vein cu-a in hind wing present. Vein Cu in hind wing present. Vein A in hind wing with abscissae A·f1 and A·f2 present.

***Gyne.*** Dichthadiiform, with minute eyes and no ocelli. The queen is known for *Labidus coecus* and *Labidus praedator*. For more details and a description of the former see Weber’s (1941) and Borgmeier (1958) for a description of *Labidus praedator* queen.

***Larva.*** Larvae of *Labidus* have been described in Wheeler (1943) and Wheeler and Wheeler (1964b, 1984). Cocoons present.

#### Distribution.

Sout Central United States to northern Argentina.

#### Taxonomy and phylogeny.

The species-level taxonomy of *Labidus* requires revision. There are currently seven valid species names and three of those are based only on males. In addition, morphology and preliminary molecular analyses suggest that the widely distributed *Labidus praedator* may be in fact a complex of reproductively isolated species (Barth et al. 2015). The phylogenetic position of *Labidus* is well-established as the sister group to the *Eciton* plus *Nomamyrmex* clade (Brady et al. 2014, Borowiec, in prep.).

#### Biology.

*Labidus* are often the most common army ants throughout their range, with up to three species occurring in a given area (do Nascimiento et al. 2004, author’s personal observations). They nest mostly underground (Fowler 1979) and forage in swarm raids. It is unclear whether brood production is synchronized; colonies appear to emigrate infrequently, their bivouacs staying in place for prolonged periods of time (Fowler 1979).

Rettenmeyer (1963) and Fowler (1979) detailed the biology of *Labidus praedator*. The bivouacs are found in rotten logs or are subterranean, occupying preformed cavities such as abandoned nest chambers of *Atta* leaf-cutting ants (Rettenmeyer 1963, Monteiro et al. 2008). Mature colonies have been estimated to contain up to a million individuals (Fowler 1979).

*Labidus* forages in swarm raids similar to those of *Eciton burchellii* (Rettenmeyer 1963) and its species are even more generalized predators that in addition to ant brood will take a variety of other arthropods, sugar, and plant parts, including flowers, seeds, fruit, and even processed food such as boiled rice (Borgmeier 1955, Monteiro et al. 2008). The two best-studies species, *Labidus coecus* and *Labidus praedator*, are similar in this respect and data on other species is lacking. Henry Walter Bates (1863) described *Labidus coecus* constructing soil tunnels over its raiding columns. Monteiro et al. (2008) studied *Labidus praedator* in agricultural lands in Brazil, finding that Lepidoptera caterpillars were the most common type of prey, followed by arils of many plant species and various non-Lepidopteran arthropods, both in adult and larval stages. Fowler (1979) observed the same species in Paraguay and reported that it frequently raided other ant colonies. The raids occur mostly during the day, although nocturnal activity is also substantial (O’Donnell et al. 2009). Perfecto (1992) observed an underground raid of *Labidus coecus* on several ant species.

The reproductive biology of *Labidus* is poorly known. There is conflicting evidence as to whether brood production is synchronized or not, with available brood samples consisting of immatures at one or multiple stages of development and queen specimens with either extended or contracted gasters (Rettenmeyer 1963). Given the rarity of emigrations and confirmed existence of long-term bivouac sites, lasting up to eight months (Fowler 1979), it is possible that *Labidus* queens retain the ability to lay eggs in pulses but do not cease brood production long enough for non-overlapping brood cohorts to emerge and for colonies to exhibit the nomadic-statary cycle characteristic of *Eciton*.

#### Species of *Labidus*

*Labidus auropubens* (Santschi, 1920a): French Guiana

*Labidus coecus* (Latreille, 1802): ‘Amérique méridionale’

*Labidus curvipes* (Emery, 1900b): Costa Rica

*Labidus mars* (Forel, 1912a): Brazil

*Labidus mars denticulatus* Borgmeier, 1955: Brazil

*Labidus praedator* (Smith, F., 1858): Brazil

*Labidus praedator sedulus* (Menozzi, 1926): Colombia

*Labidus spininodis* (Emery, 1890): Costa Rica

*Labidus truncatidens* (Santschi, 1920a): French Guiana

### Leptanilloides

Taxon classificationAnimaliaHymenopteraFormicidae

Mann, 1923

= Amyrmex Kusnezov, 1953, **syn. n.**
= Asphinctanilloides Brandão, Diniz, Agosti and Delabie, 1999, **syn. n.**

#### Type-species.

*Leptanilloides biconstricta*, by original designation.

This is a lineage of subterranean ants from Central and South America. Its members had been extremely rarely encountered before collecting methods targeting soil-dwelling ants were popularized: 17 out of the 19 currently known species were described after 1998.

#### Diagnosis.

***Worker.*** The workers of *Leptanilloides* are rather variable but can be distinguished from all other dorylines by a combination of promesonotal Pronotomesopleural suture variously developed but often conspicuous, propodeal spiracles positioned low, lack of metanotal groove, absence of propodeal lobes, and small and unarmed pygidium. The positioning of the propodeal spiracle may be rather high on the sclerite in some species and so these could conceivably be mistaken for small *Neivamyrmex*. The latter, however, can be distinguished by a complete lack of a promesonotal Pronotomesopleural suture in dorsal view and abdominal segment IV much larger than the succeeding segment V, with no apparent constrictions between abdominal segments IV, V, and VI. Although some larger *Leptanilloides* species can have an inconspicuous promesonotal Pronotomesopleural suture, those will have visible constrictions on the gaster and abdominal segment IV and that segment is never much larger than succeeding gastral segments.

***Male.*** The males of *Leptanilloides* are distinct in their extreme reduction of tegulae. The wing venation is reduced and unusual among dorylines because of the combination of costal cell (C) present in fore wing and discal cell always being open. The males of *Leptanilloides* also lack conspicuously bispinose abdominal sternite IX (subgenital plate) characteristic of almost all other male dorylines.

#### Description.

***Worker.*** *Head*: Antennae with 12 segments. Apical antennal segment not enlarged, not broader and longer than two preceding segments combined. Clypeus with cuticular apron. Lateroclypeal teeth absent or present. Parafrontal ridges absent or reduced. Torulo-posttorular complex vertical. Antennal scrobes absent. Labrum without median notch or concavity. Proximal face of stipes projecting beyond inner margin of sclerite, concealing prementum when mouthparts fully closed. Maxillary palps 2- or, rarely, 1-segmented. Labial palps 2-segmented. Mandibles triangular, with teeth or triangular with median tooth. Eyes absent. Ocelli absent. Head capsule with differentiated vertical posterior surface above occipital foramen. Ventrolateral margins of head without lamella or ridge extending towards mandibles and beyond carina surrounding occipital foramen. Posterior head corners dorsolaterally immarginate. Carina surrounding occipital foramen ventrally absent or present. *Mesosoma*: Pronotal flange not separated from collar by distinct ridge. Promesonotal connection variable, with Pronotomesopleural suture present, weakly differentiated, immobile or with Pronotomesopleural suture conspicuous and complete, but immobile, or with Pronotomesopleural suture complete and mobile. Pronotomesopleural suture visible, unfused up to notal surface. Mesometapleural groove weakly impressed to deeply impressed and conspicuous. Transverse groove dividing mesopleuron absent. Pleural endophragmal pit concavity absent. Mesosoma dorsolaterally immarginate. Metanotal depression or groove on mesosoma absent. Metanotal depression or groove on mesosoma present. Propodeal spiracle situated low on sclerite. Propodeal declivity without distinct dorsal edge or margin and rectangular in posterior view. Metapleural gland with bulla visible through cuticle. Propodeal lobes absent. *Metasoma*: Petiole anterodorsally immarginate or marginate, dorsolaterally immarginate, and laterally above spiracle immarginate. Helcium in relation to tergosternal Pronotomesopleural suture placed at posttergite and axial or slightly supraaxial. Prora simple, not delimited by carina, a simple U-shaped margin, or a V-shaped protrusion. Spiracle openings of abdominal segments IV–VI circular. Abdominal segment III anterodorsally immarginate and dorsolaterally immarginate. Abdominal segment III variable, more than half size of succeeding segment IV, which is weakly constricted at presegmental portion (uninodal waist) or abdominal segment III about half size of succeeding segment IV, which is strongly constricted at presegmental portion (binodal waist). Girdling constriction of segment IV present, i.e. pre- and postsclerites distinct. Cinctus of abdominal segment IV gutter-like, not sculptured or a gradual concavity, not gutter-like. Abdominal segment IV not conspicuously largest segment. Abdominal tergite IV not folding over sternite, and anterior portions of sternite and tergite equally well visible in lateral view. Girdling constriction between pre- and posttergites of abdominal segments V and VI absent or present. Girdling constriction between pre- and poststernites of abdominal segments V and VI absent or present. Pygidium small, reduced to narrow strip, without impressed medial field and simple, not armed with cuticular spines or modified setae. Hypopygium unarmed. *Legs*: Mid tibia with single simple/barbulate spur, with single pectinate spur, or rarely with two simple spurs. Hind tibia with pectinate spur or rarely with one barbulate and one pectinate spur. Hind basitarsus not widening distally, circular in cross-section. Posterior flange of hind coxa not produced as raised lamella. Metatibial gland present as oval patch of whitish cuticle. Metabasitarsal gland absent. Hind pretarsal claws simple. *Polymorphism*: Monomorphic.

***Male.*** *Head*: Antennae with 13 segments. Clypeus with or without cuticular apron. Parafrontal ridges absent. Torulo-posttorular complex vertical. Maxillary palps unknown. Labial palps unknown. Mandibles falcate or, more rarely, elongately triangular, edentate, or intermediate. Ventrolateral margins of head without lamella or ridge extending towards mandibles and beyond carina surrounding occipital foramen. Carina surrounding occipital foramen ventrally absent. *Mesosoma*: Pronotal flange not separated from collar by distinct ridge. Notauli absent. Transverse groove dividing mesopleuron absent. Propodeal declivity reduced, without distinct dorsal edge or margin. Metapleural gland opening absent. Propodeal lobes absent. *Metasoma*: Petiole anterodorsally immarginate, dorsolaterally immarginate, and laterally above spiracle immarginate. Helcium in relation to tergosternal Pronotomesopleural suture placed at posttergite or at Pronotomesopleural suture and axial. Prora simple, not delimited by carina. Spiracle openings of abdominal segments IV–VI circular. Abdominal segment III more than half size of succeeding segment IV; latter weakly constricted at presegmental portion (uninodal waist). Girdling constriction of segment IV absent, i.e. pre- and postsclerites indistinct. Cinctus of abdominal segment IV absent, not impressed. Girdling constriction between pre- and postsclerites of abdominal segments V and VI absent. Abdominal segment IV not conspicuously largest segment. Abdominal sternite VII simple. Abdominal sternite IX simple or cleft, with lateral apodemes reduced, only medial apodeme conspicuous, short. *Genitalia*: Cupula short relative to rest of genital capsule and of approximately equal length on both dorsal and ventral surfaces. Basimere broadly fused to telomere, with sulcus discernable at junction or no sulcus trace at junction, and ventrally with left and right arms abutting. Telomere gradually tapering toward apex. Volsella gradually tapering toward apex. Penisvalva laterally compressed, rounded at apex. *Legs*: Mid tibia with single simple/barbulate spur or with single pectinate spur. Hind tibia with single pectinate spur. Posterior flange of hind coxa not produced as raised lamella. Metatibial gland absent. Metabasitarsal glands absent. Hind pretarsal claws simple. *Wings*: Tegula absent or extremely small. Vein C in fore wing present. Pterostigma narrow or broad. Abscissa R·f3 absent. Abscissae Rs·f2–3 present, connecting with Rs+M&M·f2. Cross-vein 2r-rs present, differentiated from Rs·f4 by presence of Rs·f2–3. Abscissae Rs·f4–5 present, fused in absence of 2rs-m. Abscissa M·f2 in fore wing contiguous with Rs+M or absent. Abscissa M·f4 in fore wing absent or present, not reaching wing margin. Cross-vein 1m-cu in fore wing absent. Cross-vein cu-a in fore wing present, arising from Cu and distal to, at or near M·f1. Vein Cu in fore wing present, with only Cu1 branch prominent. Vein A in fore wing with abscissa A·f1 present. Vein C in hind wing absent or present. Vein R in hind wing absent. Vein Sc+R in hind wing absent or present. Abscissa Rs·f1 in hind wing absent or present, shorter than 1rs-m. Abscissa Rs·f2 in hind wing absent or present, not reaching wing margin. Cross-vein 1rs-m in hind wing absent. Vein M+Cu in hind wing absent. Abscissa M·f1 in hind wing absent. Abscissa M·f2 in hind wing absent. Cross-vein cu-a in hind wing absent. Vein Cu in hind wing absent. Vein A in hind wing absent.

***Gyne.*** The queens of *Leptanilloides* collected so far have been ‘subdichthadiiform’, or wingless ergatoids with eyes and hypertrophied gaster, including abdominal segment III. The gynes possess eyes but no ocelli. See description of *Leptanilloides erinys* gyne in Borowiec and Longino (2011); Donoso et al. (2006) also reported blind intercastes in addition to a subdichthadiigyne in *Leptanilloides nubecula*.

***Larva.*** Larva was described by Brandão et al. (1999a). Presence of cocoons unknown.

#### Distribution.

*Leptanilloides* is a genus found throughout Central America, from Chiapas, Mexico to Panama, and from scattered, mostly high-elevation records from Bolivia, Colombia, Ecuador, and Venezuela. It is also present in the Amazon Basin and one species has been described from the Atlantic forest habitat of São Paulo, Brazil. A recent collection from western Texas (MacGown et al. 2015) is a remarkable range extension for this lineage.

#### Taxonomy and phylogeny.

*Leptanilloides* was described with one species, *Leptanilloides biconstricta*, in 1923 (Mann 1923). Subsequently a closely related genus *Asphinctanilloides* was described, along with new *Leptanilloides* species (Brandão et al. 1999a). *Asphinctanilloides* was originally differentiated from *Leptanilloides* by several characters. The genus *Amyrmex*, known only from males, was originally placed erroneously in the Dolichoderinae (Ward and Brady 2009). Recent descriptions of additional *Leptanilloides* species reveal greater morphological diversity of the genus and blur the distinction from *Asphinctanilloides* (Longino 2003, Donoso et al. 2006, Borowiec and Longino 2011). Because of this it has been suggested that *Leptanilloides* may eventually prove paraphyletic with regards to *Asphinctanilloides* and it has already been shown to be paraphyletic with respect to *Amyrmex* (see Ward and Brady 2009). No *Asphinctanilloides* have ever been included in a molecular analysis but it is possible that the males described as *Amyrmex* in fact correspond to *Asphinctanilloides*. Here I propose synonymy of both *Amyrmex* and *Asphinctanilloides* under *Leptanilloides*. *Leptanilloides* in this new sense is easily identified in both worker and males and guarantees more stable taxonomy in the face of potential paraphyly issues, although it encompasses morphologically disparate forms. It is possible that future workers will feel justified to split this genus once again after a better understanding of worker and male diversity is attained. Delsinne et al. (2015) provide the most recent key to species of *Leptanilloides* excluding *Asphinctanilloides*, but the species that were placed in the latter can still be identified using Brandão et al. (1999a).

The phylogenetic position of *Leptanilloides* within Dorylinae was difficult to ascertain with Sanger sequencing data (Brady et al. 2014) but genomic data suggest that it forms a clade with *Sphinctomyrmex* and New World army ants. Several species have been included in morphology-based and molecular phylogenies (Brandão et al. 1999a, Ward 2007, Ward and Brady 2009, Delsinne et al. 2015, MacGown et al. 2015, Borowiec, in prep.) but the availability of fresh material precludes a comprehensive analysis.

#### Biology.

*Leptanilloides nomada* was observed foraging at night in partly subterranean, partly exposed, columns but the ants did not carry any prey (Donoso et al. 2006). In *Leptanilloides nubecula* the colonies are apparently polygynous, with subdichthadiigyne queens and intercastes present (Donoso et al. 2006), but a complete colony of *Leptanilloides erinys* contained only single subdichthadiiform queen (Borowiec and Longino 2011). Brood apparently develops in synchrony, as all nest collections so far contain larvae of uniform size (Brandão et al. 1999a, b, Donoso et al. 2006, Borowiec and Longino 2011). Brandão et al. (1999b) summarized the scant information available on species then classified in *Asphinctanilloides*. They report workers of *Leptanilloides anae* moving in columns similar to that of army ants, preying on an unidentified, dismembered arthropod under cow dung. This would suggest that this species may not be a specialized predator of other ants, like most dorylines, although it is clear that more observations are needed. As evidence for hypogaeic habits Brandão et al. (1999b) note that where intensive surveys of leaf litter ants have been carried out specimens have not been collected by that method but only from soil samples. Specimens have also been extracted from stomachs of subterranean amphisbaenians, giving further evidence for the underground lifestyle.

#### Species of *Leptanilloides*

*Leptanilloides amazona* Brandão, Diniz, Agosti and Delabie, 1999: Brazil, **comb. n.**

*Leptanilloides anae* Brandão, Diniz, Agosti and Delabie, 1999: Brazil, **comb. n.**

*Leptanilloides atlantica* Silva, Brandão, Feitosa and Freitas, 2013: Brazil

*Leptanilloides biconstricta* Mann, 1923: Bolivia

*Leptanilloides caracola* Donoso, Vieira and Wild, 2006: Ecuador

*Leptanilloides chihuahuaensis* MacGown, Schiefer and Branstetter 2015: United States

*Leptanilloides copalinga* Delsinne and Donoso 2015: Ecuador

*Leptanilloides erinys* Borowiec and Longino, 2011: Ecuador

*Leptanilloides femoralis* Borowiec and Longino, 2011: Venezuela

*Leptanilloides golbachi* Kusnezov, 1953: Argentina, **comb. n.**

*Leptanilloides gracilis* Borowiec and Longino, 2011: Mexico

*Leptanilloides improvisa* Brandão, Diniz, Agosti and Delabie, 1999: Ecuador

*Leptanilloides legionaria* Brandão, Diniz, Agosti and Delabie, 1999: Colombia

*Leptanilloides manaura* Brandão, Diniz, Agosti and Delabie, 1999: Brazil, **comb. n.**

*Leptanilloides mckennae* Longino, 2003: Costa Rica

*Leptanilloides nomada* Donoso, Vieira and Wild, 2006: Ecuador

*Leptanilloides nubecula* Donoso, Vieira and Wild, 2006: Ecuador

*Leptanilloides prometea* Delsinne and Donoso, 2015: Ecuador

*Leptanilloides sculpturata* Brandão, Diniz, Agosti and Delabie, 1999: Colombia

### Lioponera

Taxon classificationAnimaliaHymenopteraFormicidae

Mayr, 1879 gen. rev.

= Neophyracaces Clark, 1941, **syn. n.**
= Phyracaces Emery, 1902, **syn. n.**

#### Type-species.

*Lioponera longitarsus*, by monotypy.

This is the most species-rich genus that is revived here from synonymy under *Cerapachys. Lioponera* occurs only in the Old World and all species observed thus far prey on other ants.

#### Diagnosis.

***Worker.*** The workers of *Lioponera* are distinguishable using the combination of a unique cuticular flange present on the posterior edge of the hind coxa, just posterior to the femur attachment, at least the anterior half of the petiole being dorsolaterally marginate, and a peculiar development of the metatibial gland that forms an open slit in the cuticle. The coxal flange should not to be confused with the elevated faces of coxa that can be conspicuous when there is a deep trench leading to the articulation with the femur, as can be sometimes seen in e.g. *Simopone*. The coxal flange and metatibial gland have been reduced in a handful of Australasian species, but in these the dorsolateral carinae of petiole and (usually) also postpetiole and mesosoma, are prominent. The dorsolateral margination of the body is characteristic and in most species very conspicuous on the abdominal segments II (petiole) and III. In a few species the margination extends from the head to the abdominal segment IV. The only other genera that can possess somewhat similar dorsolateral carinae on the petiole are *Acanthostichus*, *Cerapachys*, and *Cylindromyrmex*. The workers of those groups, however, do not possess a coxal flange.

***Male.*** The males of the many species of *Lioponera* are variable. Several characteristics can point to the affinity with this genus: antennae are 13-segmented, costal vein (C) is always absent from the fore wing, a ‘free stigmal vein’ (2r-rs&Rs·f4–5) is present and R·f3 and Rs·f2–3 are always absent. Cross-vein 2rs-m is usually absent but its traces can be rarely seen as a weak spectral vein arising close to 2r-rs. There is a constriction between abdominal segments III and IV but no succeeding segments and the middle tibiae are armed with a single spur. The notauli are present or absent, pretarsal claws are unarmed, and the palp formula is either 4,3 or 3,2. Compare diagnoses of the genera where a free stigmal vein is also found (*Eburopone*, *Ooceraea*, *Syscia*, *Tanipone*). *Eburopone* can be distinguished by costal (C) vein always present in the fore wing, *Ooceraea* and *Syscia* have 12- or 11-segmented antennae, and *Tanipone* has very long, 6-segmented maxillary palps that are extruded in mounted specimens and reach the occipital foramen.

#### Description.

***Worker.*** *Head*: Antennae with 12 segments. Apical antennal segment not enlarged, not broader and longer than two preceding segments combined to moderately enlarged, broader than and about equal in length to two preceding segments combined. Clypeus with cuticular apron. Lateroclypeal teeth absent. Parafrontal ridges reduced. Torulo-posttorular complex vertical. Antennal scrobes absent. Labrum with median notch or concavity. Proximal face of stipes projecting beyond inner margin of sclerite, concealing prementum when mouthparts fully closed. Maxillary palps 4- or 3-segmented. Labial palps 3- or 2-segmented. Mandibles triangular, with teeth. Eyes present, composed of more than 20 ommatidia. Ocelli absent or more rarely present. Head capsule with differentiated vertical posterior surface above occipital foramen. Ventrolateral margins of head with or without lamella or ridge extending towards mandibles and beyond carina surrounding occipital foramen. Posterior head corners dorsolaterally immarginate or marginate. Carina surrounding occipital foramen ventrally absent. *Mesosoma*: Pronotal flange separated from collar by distinct ridge. Promesonotal connection with Pronotomesopleural suture completely fused. Pronotomesopleural suture visible, unfused partway to notal surface. Mesometapleural groove deeply impressed, conspicuous. Transverse groove dividing mesopleuron present. Pleural endophragmal pit concavity present. Mesosoma dorsolaterally immarginate, weakly marginate, or conspicuously marginate. Metanotal depression or groove on mesosoma absent. Propodeal spiracle situated low on sclerite. Propodeal declivity with or without distinct dorsal edge or margin and rectangular in posterior view. Metapleural gland with bulla visible through cuticle. Propodeal lobes present, well developed. *Metasoma*: Petiole anterodorsally immarginate, dorsolaterally marginate, and laterally above spiracle immarginate or marginate. Helcium in relation to tergosternal Pronotomesopleural suture placed at Pronotomesopleural suture and axial. Prora simple, not delimited by carina or a V-shaped protrusion. Spiracle openings of abdominal segments IV–VI circular. Abdominal segment III anterodorsally immarginate or marginate and dorsolaterally immarginate or marginate. Abdominal segment III more than half size of succeeding segment IV, which is weakly constricted at presegmental portion (uninodal waist). Girdling constriction of segment IV present, i.e. pre- and postsclerites distinct. Cinctus of abdominal segment IV gutter-like, not sculptured or cross-ribbed. Abdominal segment IV not conspicuously largest segment. Abdominal tergite IV not folding over sternite, and anterior portions of sternite and tergite equally well visible in lateral view. Girdling constriction between pre- and posttergites of abdominal segments V and VI absent. Girdling constriction between pre- and poststernites of abdominal segments V and VI absent. Pygidium large, with impressed medial field and armed with modified setae. Hypopygium unarmed. *Legs*: Mid tibia with pectinate spur or, more rarely, with barbulate spur and simple spur. Hind tibia with single pectinate spur. Hind basitarsus not widening distally, circular in cross-section. Posterior flange of hind coxa produced as raised lamella or rarely without lamella. Metatibial gland present in form of slit or orifice in cuticle or rarely absent. Metabasitarsal gland absent. Hind pretarsal claws simple. *Polymorphism*: Monomorphic.

***Male.*** *Head*: Antennae with 13 segments. Clypeus with cuticular apron. Parafrontal ridges absent. Torulo-posttorular complex vertical. Maxillary palps 3-segmented. Labial palps 2-segmented. Mandibles triangular, edentate. Ventrolateral margins of head with or without cuticular ridge extending towards mandibles and beyond carina surrounding occipital foramen. Carina surrounding occipital foramen ventrally absent. *Mesosoma*: Pronotal flange not separated from collar by distinct ridge. Notauli absent or present. Transverse groove dividing mesopleuron present. Propodeal declivity with distinct dorsal edge or margin. Metapleural gland opening present. Propodeal lobes present. *Metasoma*: Petiole anterodorsally immarginate or marginate, dorsolaterally marginate, and laterally above spiracle marginate. Helcium in relation to tergosternal Pronotomesopleural suture placed at Pronotomesopleural suture and axial. Prora forming a simple U-shaped margin or U-shaped protrusion. Spiracle openings of abdominal segments IV–VI circular. Abdominal segment III more than half size of succeeding segment IV; latter weakly constricted at presegmental portion (uninodal waist). Girdling constriction of segment IV present, i.e. pre- and postsclerites distinct. Cinctus of abdominal segment IV gutter-like and cross-ribbed. Girdling constriction between pre- and postsclerites of abdominal segments V and VI absent. Abdominal segment IV not conspicuously largest segment. Abdominal sternite VII simple. Abdominal sternite IX cleft or modified into two spines, with lateral apodemes about as long as medial apodeme, directed anteriorly (towards head). *Genitalia*: Cupula long relative to rest of genital capsule and of approximately equal length on both dorsal and ventral surfaces. Basimere broadly fused to telomere, with no sulcus trace at junction, and ventrally with left and right arms abutting. Telomere gradually tapering toward apex. Volsella gradually tapering toward apex. Penisvalva constricted basally, distally a widening triangle, serrated ventrally. *Legs*: Mid tibia with single pectinate spur. Hind tibia with single pectinate spur. Posterior flange of hind coxa produced as raised lamella or not. Metatibial gland absent. Metabasitarsal glands absent. Hind pretarsal claws simple. *Wings*: Tegula present, broad, demiovate in shape. Vein C in fore wing absent. Pterostigma broad. Abscissa R·f3 absent. Abscissae Rs·f2–3 absent. Cross-vein 2r-rs most often present and forming base of ‘free stigmal vein’ (2r-rs&Rs·f4–5) in absence of Rs·f3 and 2rs-m or present and connected to Rs·f2–3&Rs·f4 or, rarely, absent. Abscissae Rs·f4–5 absent or present, fused in absence of 2rs-m or, more rarely, differentiated into Rs·f4 and Rs·f5 by 2rs-m. Abscissa M·f2 in fore wing absent or contiguous with Rs+M. Abscissa M·f4 in fore wing present, not reaching wing margin. Cross-vein 1m-cu in fore wing present or more rarely absent. Cross-vein cu-a in fore wing present, arising from M+Cu and proximal to M·f1 or more rarely absent. Vein Cu in fore wing present, with only Cu1 branch prominent or absent past M+Cu. Vein A in fore wing with abscissa A·f1 present with abscissae A·f1 and A·f2 present. Vein C in hind wing absent. Vein R in hind wing absent. Vein Sc+R in hind wing present. Abscissa Rs·f1 in hind wing absent or present, shorter than 1rs-m. Abscissa Rs·f2 in hind wing absent or present, not reaching wing margin. Cross-vein 1rs-m in hind wing absent or present, about as long as M·f1. Vein M+Cu in hind wing absent or present. Abscissa M·f1 in hind wing absent or present. Abscissa M·f2 in hind wing absent. Cross-vein cu-a in hind wing absent or present. Vein Cu in hind wing absent or present. Vein A in hind wing absent or with abscissa A·f1 present.

***Gyne.*** Alate or ergatoid; apparent intercastes have also been reported. Alate or apparently alate (known from dealated specimens) gynes are known in a number of species, for example in *Lioponera clarki*, *Lioponera daikoku*, *Lioponera fervida*, *Lioponera huode*, *Lioponera pubescens*, *Lioponera turneri*. Ergatoid gynes are known to vary in morphology, from scarcely different from the worker, through possessing relatively larger eyes and ocelli, to having the mesosomal morphology identical to that of winged queens but possessing no wings. Ergatoids have been reported in, for example, *Lioponera angustata*, *Lioponera bicolor*, *Lioponera constricta*, *Lioponera elegans*, *Lioponera gilesi*, *Lioponera nigriventris*, *Lioponera punctatissima*, *Lioponera simmonsae*. Malagasy species related to *Lioponera kraepelini* and *Lioponera mayri* also possess ergatoid gynes (author’s observations). Intercastes with morphologies intermediate between workers and alate gynes have been reported to occur along with fully developed gynes in *Lioponera clarki* (Clark 1924a, 1924b). See Clark (1924a, 1924b) for example descriptions and illustrations of *Lioponera* gynes.

***Larva.*** Larvae of *Lioponera* have been described by Wheeler and Wheeler (1964a). Cocoons are present.

#### Distribution.

This is the most species-rich lineage outside the true army ants, distributed throughout the Old World, from Africa to Oceania, with a major radiation in Australia.

#### Taxonomy and phylogeny.

Originally *Lioponera* was described for *Lioponera longitarsus*, a species from India (Mayr 1879). In Genera Insectorum, Emery (1911) recognized *Lioponera*, *Phyracaces* and *Cerapachys* in his tribe Cerapachyini. Since then, several species were described in *Lioponera*, but many more taxa placed here under that name were originally described in *Phyracaces*. Brown (1975) synonymized both taxa under *Cerapachys*.

*Lioponera* is part of a well-supported Old World clade where the intergeneric relationships are known (Brady et al. 2014, Borowiec, in prep.). Within this clade, *Lioponera* branches off first and is sister to the (*Lividopone* (*Parasyscia* plus *Zasphinctus*) clade. A phylogeny of the relatively few species that have been sequenced suggests that the genus may have originated in Africa and later spread to the Indomalayan and Australasian regions.

#### Biology.

Members of this lineage have been observed both in the field and in the laboratory (Brown 1975, Clark, 1924a and 1924b, Hölldobler 1982, Wilson 1958). As with most other dorylines, they are predators of other ants and a variety of prey species have been reported. Hölldobler (1982) studied an Australian Lioponera species near turneri under laboratory conditions. He showed that scouts recruit nestmates to raids via a pheromone trail, the species exhibited a preference for *Pheidole* when presented with a variety of other ants, and that the brood of the prey was paralyzed by stinging and stored alive for up to two months. Brood production is apparently not synchronized, at least in some species. Clark (1924a, 1924b) observed the Australian species *Lioponera clarki* and *Lioponera punctatissima* foraging singly around its nest but did not mention any prey; he reported that the workers were peculiar in holding their abdomens over the mesosma when foraging in both species. Some Australian species are said to be crepuscular foragers, active in either morning or evening, while others are capable of raiding during the hottest parts of the day (Clark 1924b).

*Lioponera* nests are found in a variety of microhabitats, including soil, under stones, in rotting logs or arboreally in hollow twigs (Wilson 1958, Brown 1975).

#### Species of *Lioponera*

*Lioponera aberrans* (Clark, 1934): Australia, **comb. n.**

*Lioponera adama* (Forel, 1910a): Australia, **comb. n.**

*Lioponera angustata* (Clark, 1924b): Australia, **comb. n.**

*Lioponera anokha* (Bharti and Akbar, 2013): India, **comb. n.**

*Lioponera bakeri* Wheeler, W. M. and Chapman, 1925: Philippines, **comb. rev.**

*Lioponera bicolor* (Clark, 1924b): Australia, **comb. n.**

*Lioponera binodis* (Forel, 1910a): Australia, **comb. n.**

*Lioponera braunsi* (Emery, 1902): South Africa, **comb. n.**

*Lioponera braytoni* (Weber, 1949a): Kenya, **comb. n.**

*Lioponera brevicollis* (Clark, 1924a): Australia, **comb. n.**

*Lioponera brevis* (Clark, 1924b): Australia, **comb. n.**

*Lioponera clarki* (Crawley, 1922): Australia, **comb. n.**

*Lioponera clarus* (Clark, 1930): Australia, **nom. rev.**

*Lioponera cohici* (Wilson, 1957): New Caledonia, **comb. n.**

*Lioponera collingwoodi* (Sharaf, 2007): Egypt, **comb. n.**

*Lioponera constricta* (Clark, 1924a): Australia, **comb. n.**

*Lioponera coxalis* (Arnold, 1926): Zimbabwe, **comb. n.**

*Lioponera crassa* (Clark, 1941): Australia, **comb. n.**

*Lioponera daikoku* (Terayama, 1996): Japan, **comb. n.**

*Lioponera decorsei* Santschi, 1912: Chad, **comb. rev.**

*Lioponera desertorum* (Dlussky, 1990): Uzbekistan, **comb. n.**

*Lioponera dumbletoni* (Wilson, 1957): New Caledonia, **comb. n.**

*Lioponera elegans* (Wheeler, W. M., 1918): Australia, **comb. n.**

*Lioponera emeryi* (Viehmeyer, 1914): Australia, **nom. rev.**

*Lioponera fervida* (Wheeler, W. M., 1918): Australia, **comb. n.**

*Lioponera ficosa* (Wheeler, W. M., 1918): Australia, **comb. n.**

*Lioponera flammea* (Clark, 1930): Australia, **comb. n.**

*Lioponera foreli* (Santschi, 1914b): Ghana, **comb. n.**

*Lioponera gilesi* (Clark, 1924a): Australia, **comb. n.**

*Lioponera grandis* (Clark, 1934): Australia, **comb. n.**

*Lioponera greavesi* (Clark, 1934): Australia, **comb. n.**

*Lioponera gwynethae* (Clark, 1941): Australia, **comb. n.**

*Lioponera heros* (Wheeler, W. M., 1918): Australia, **comb. n.**

*Lioponera hewitti* (Wheeler, W. M., 1919): Malaysia (Sarawak), **comb. n.**

*Lioponera huode* (Terayama, 2009): Taiwan, **comb. n.**

*Lioponera inconspicua* (Clark, 1924b): Australia, **nom. rev.**

*Lioponera jovis* (Forel, 1915): Australia, **comb. n.**

*Lioponera kraepelinii* (Forel, 1895d): Madagascar, **comb. n.**

*Lioponera krombeini* (Donisthorpe, 1947): Papua New Guinea, **comb. n.**

*Lioponera larvata* (Wheeler, W. M., 1918): Australia, **comb. n.**

*Lioponera longitarsus* Mayr, 1879: India, **comb. rev.**

*Lioponera luzuriagae* Wheeler, W. M. and Chapman, 1925: Philippines, **comb. rev.**

*Lioponera macrops* (Clark, 1941): Australia, **comb. n.**

*Lioponera marginata* (Emery, 1897): Papua New Guinea, **comb. n.**

*Lioponera mayri* (Forel, 1892b): Madagascar, **comb. n.**

*Lioponera mjoebergi* (Forel, 1915): Australia, **comb. n.**

*Lioponera mullewana* (Wheeler, W. M., 1918): Australia, **comb. n.**

*Lioponera nayana* (Bharti and Akbar, 2013): India, **comb. n.**

*Lioponera nigra* Santschi, 1914a: Kenya, **comb. rev.**

*Lioponera nigriventris* (Clark, 1924b): Australia, **comb. n.**

*Lioponera nkomoensis* (Forel, 1916): Democratic Republic of the Congo, **comb. n.**

*Lioponera noctambula* Santschi, 1910a: Tunisia, **comb. rev.**

*Lioponera picipes* (Clark, 1924b): Australia, **comb. n.**

*Lioponera picta* (Clark, 1934): Australia, **comb. n.**

*Lioponera piliventris* (Clark, 1941): Australia, **comb. n.**

*Lioponera potteri* (Clark, 1941): Australia, **comb. n.**

*Lioponera pruinosa* (Brown, 1975): Philippines, **comb. n.**

*Lioponera pubescens* (Emery, 1902): Indonesia (Laut Island), **comb. n.**

*Lioponera punctatissima* (Clark, 1924a): Australia, **comb. n.**

*Lioponera reticulata* (Clark, 1926): Australia, **nom. rev.**

*Lioponera ruficornis* (Clark, 1924a): Australia, **comb. n.**

*Lioponera rugulinodis* (Wheeler, W. M., 1918): Australia, **comb. n.**

*Lioponera senescens* (Wheeler, W. M., 1918): Australia, **comb. n.**

*Lioponera similis* Santschi, 1930: Ivory Coast, **comb. rev.**

*Lioponera simmonsae* (Clark, 1924a): Australia, **comb. n.**

*Lioponera singaporensis* (Viehmeyer, 1916): Singapore, **comb. n.**

*Lioponera singularis* (Forel, 1900a): Australia, **comb. n.**

*Lioponera sjoestedti* (Forel, 1915): Australia, **comb. n.**

*Lioponera suscitata* (Viehmeyer, 1913): Indonesia (Sulawesi, in copal), **comb. n.**

*Lioponera turneri* (Forel, 1902): Australia, **comb. n.**

*Lioponera varians* (Clark, 1924b): Australia, **comb. n.**

*Lioponera versicolor* Donisthorpe, 1948: Indonesia (Papua), **comb. rev.**

*Lioponera vespula* (Weber, 1949a): Kenya, **comb. n.**

### Lividopone

Taxon classificationAnimaliaHymenopteraFormicidae

Fisher & Bolton, 2016

#### Type-species.

*Cerapachys lividus*, by subsequent designation.

This relatively speciose lineage includes species nesting in a variety of substrates, from soil to twigs. It is known that they prey on brood of other ants.

#### Diagnosis.

***Worker.*** The workers of *Lividopone* are recognized by a combination of 12-segmented antennae, pronotomesopleural Pronotomesopleural suture fused, propodeal spiracle positioned low and presence of propodeal lobes, pygidium large and armed with modified setae, helcium broad and positioned supraaxially on the sclerite, middle tibiae with a single pectinate spur, and pretarsal claws simple. *Cerapachys* is similar in general habitus but it is easily differentiated because of its unfused pronotomesopleural Pronotomesopleural suture. *Parasyscia*, certain *Lioponera* and *Simopone* may have a similar habitus but those genera never have a broad, highly positioned helcium.

***Male.*** The male of *Lividopone* shares the broad supraaxial helcium with the worker caste, which makes it easy to distinguish from any other doryline when combined with well-developed propodeal lobes, antennal toruli exposed in full-face view, one spur on each mid and hind tibia, and no C or R·f3 veins in the fore wing. Males of *Lividopone* can potentially be confused with *Lioponera* and *Parasyscia* which also lack veins C and R·f3 and with which it co-occurs, but the highly broad positioned helcium alone can distinguish it from those two lineages.

#### Description.

***Worker.*** *Head*: Antennae with 12 segments. Apical antennal segment conspicuously enlarged, much broader than and longer than two preceding segments combined. Clypeus with or without cuticular apron. Lateroclypeal teeth absent. Parafrontal ridges reduced. Torulo-posttorular complex vertical. Antennal scrobes absent. Labrum with median notch or concavity. Proximal face of stipes projecting beyond inner margin of sclerite, concealing prementum when mouthparts fully closed. Maxillary palps 3-segmented. Labial palps 2-segmented. Mandibles triangular, with teeth or edentate. Eyes present, composed of more than 20 ommatidia. Ocelli absent. Head capsule with differentiated vertical posterior surface above occipital foramen. Ventrolateral margins of head without lamella or ridge extending towards mandibles and beyond carina surrounding occipital foramen. Ventrolateral margins of head with cuticular ridge extending towards mandibles and beyond carina surrounding occipital foramen. Posterior head corners dorsolaterally immarginate. Carina surrounding occipital foramen ventrally absent or present. *Mesosoma*: Pronotal flange separated or not from collar by distinct ridge. Promesonotal connection with Pronotomesopleural suture completely fused. Pronotomesopleural suture completely fused. Mesometapleural groove deeply impressed, conspicuous. Transverse groove dividing mesopleuron present. Pleural endophragmal pit concavity present. Mesosoma dorsolaterally immarginate. Metanotal depression or groove on mesosoma absent. Propodeal spiracle situated low on sclerite. Propodeal declivity with or without distinct dorsal edge or margin and rectangular in posterior view. Metapleural gland without bulla visible through cuticle. Propodeal lobes present, well developed. *Metasoma*: Petiole anterodorsally immarginate, dorsolaterally immarginate, and laterally above spiracle marginate. Helcium in relation to tergosternal Pronotomesopleural suture placed at posttergite and supraaxial. Prora forming a simple U-shaped margin or V-shaped protrusion. Spiracle openings of abdominal segments IV–VI circular. Abdominal segment III anterodorsally immarginate and dorsolaterally immarginate. Abdominal segment III more than half size of succeeding segment IV, which is weakly constricted at presegmental portion (uninodal waist). Girdling constriction of segment IV present, i.e. pre- and postsclerites distinct. Cinctus of abdominal segment IV gutter-like and cross-ribbed. Abdominal segment IV not conspicuously largest segment. Abdominal tergite IV not folding over sternite, and anterior portions of sternite and tergite equally well visible in lateral view. Girdling constriction between pre- and posttergites of abdominal segments V and VI absent. Girdling constriction between pre- and poststernites of abdominal segments V and VI absent. Pygidium large, with impressed medial field, and armed with modified setae, and in some species deeply notched at apex. Hypopygium unarmed. *Legs*: Mid tibia with single pectinate spur. Hind tibia with single pectinate spur. Hind basitarsus not widening distally, circular in cross-section. Posterior flange of hind coxa not produced as raised lamella. Metatibial gland absent. Metabasitarsal gland absent. Hind pretarsal claws simple. *Polymorphism*: Monomorphic.

***Male.*** *Head*: Antennae with 13 segments. Clypeus with cuticular apron. Parafrontal ridges present. Torulo-posttorular complex vertical. Maxillary palps unknown. Labial palps unknown. Mandibles triangular, edentate. Ventrolateral margins of head with cuticular ridge extending towards mandibles and beyond carina surrounding occipital foramen. Carina surrounding occipital foramen unknown. *Mesosoma*: Pronotal flange separated from collar by distinct ridge. Notauli present. Transverse groove dividing mesopleuron present. Propodeal declivity with distinct dorsal edge or margin. Metapleural gland opening absent. Propodeal lobes present. *Metasoma*: Petiole anterodorsally marginate, dorsolaterally immarginate, and laterally above spiracle marginate. Helcium in relation to tergosternal Pronotomesopleural suture placed at Pronotomesopleural suture and supraaxial. Prora forming a U-shaped protrusion. Spiracle openings of abdominal segments IV–VI circular. Abdominal segment III more than half size of succeeding segment IV; latter weakly constricted at presegmental portion (uninodal waist). Girdling constriction of segment IV present, i.e. pre- and postsclerites distinct. Cinctus of abdominal segment IV gutter-like and cross-ribbed. Girdling constriction between pre- and postsclerites of abdominal segments V and VI absent. Abdominal segment IV not conspicuously largest segment. Abdominal sternite VII simple. Abdominal sternite IX distally armed with two spines, with lateral apodemes about as long as medial apodeme, directed anteriorly (towards head). *Genitalia*: Cupula long relative to rest of genital capsule and of approximately equal length on both dorsal and ventral surfaces. Basimere broadly fused to telomere, with sulcus discernable at junction, and ventrally with left and right arms abutting. Telomere gradually tapering toward apex. Volsella gradually tapering toward apex. Penisvalva constricted basally, distally a widening to narrow triangle, serrated ventrally. *Legs*: Mid tibia with single pectinate spur. Hind tibia with single pectinate spur. Posterior flange of hind coxa not produced as raised lamella. Metatibial gland absent. Metabasitarsal glands absent. Hind pretarsal claws simple. *Wings*: Tegula present, broad, demiovate in shape. Vein C in fore wing absent. Pterostigma broad. Abscissa R·f3 absent. Abscissae Rs·f2–3 present, connecting with Rs+M&M·f2 or more rarely absent. Cross-vein 2r-rs absent or present, forming base of ‘free stigmal vein’ (2r-rs&Rs·f4–5) in absence of Rs·f3 and 2rs-m, or (most commonly) present, differentiated from Rs·f4 by presence of Rs·f2–3. Abscissae Rs·f4–5 absent or more often present, fused in absence of 2rs-m. Abscissa M·f2 in fore wing absent or present, separated from Rs+M by Rs·f2. Abscissa M·f4 in fore wing absent or a stub. Cross-vein 1m-cu in fore wing absent or present. Cross-vein cu-a in fore wing absent or fore wing present, arising from M+Cu and proximal to M·f1. Vein Cu in fore wing present, with only Cu1 branch prominent. Vein A in fore wing absent or with abscissae A·f1 and A·f2 present. Vein C in hind wing absent. Vein R in hind wing absent. Vein Sc+R in hind wing absent. Abscissa Rs·f1 in hind wing absent or present, longer than 1rs-m. Abscissa Rs·f2 in hind wing absent or stub present. Cross-vein 1rs-m in hind wing absent or present, about as long as M·f1. Vein M+Cu in hind wing absent or present. Abscissa M·f1 in hind wing absent or present. Abscissa M·f2 in hind wing absent or present. Cross-vein cu-a in hind wing absent. Vein Cu in hind wing absent or present. Vein A in hind wing absent.

***Gyne.*** Alate with large eyes and ocelli or ergatoid. The latter variously developed, from forms possessing flight-associated mesosomal sutures but no wings to extremely worker-like, without ocelli and of similar size, with only erect pubescence distinguishing the gyne from worker. In some species intercastes occur in addition to alate gynes (Barry Bolton pers. comm.).

***Larva.*** Not described. Cocoons are present.

#### Distribution.

This group is relatively species-rich with only one named species but some 30 more species awaiting description. The genus appears to be restricted to Madagascar.

#### Taxonomy and phylogeny.

Brown (1975) described *Cerapachys lividus* from Madagascar, for which the name *Lividopone* was recently proposed (Fisher and Bolton 2016).

*Lividopone* is sister to the *Zasphinctus* plus *Parasyscia* clade (Brady et al. 2014, Borowiec, in prep.).

#### Biology.

Brown (1975) observed about 20 workers of *Lividopone livida* raiding a *Pheidole* nest in a rainforest habitat. I discovered a nest of *Lividopone* in a dead log in mid-elevation forest, containing only about 15 workers, one dealate gyne and no brood. Similarly small colonies were recorded for an undescribed arboreal *Lividopone*. All four nests of that species I collected were in hollow twigs situated above forest floor. These colonies each contained multiple apparent gynes, which were extremely worker-like and differed from other individuals only in conspicuously erect pilosity. One of the colonies contained brood of *Monomorium termitobium* as prey. This arboreal species also apparently synchronizes brood production and nest samples containing brood of the same stage of development are known for *Lividopone livida* and other undescribed species (author’s observations)

#### Species of *Lividopone*

*Lividopone livida* (Brown, 1975): Madagascar

### Neivamyrmex

Taxon classificationAnimaliaHymenopteraFormicidae

Borgmeier, 1940

= Acamatus Emery, 1894
= Woitkowskia Enzmann, 1952

#### Type-species.

Eciton (Acamatus) schmitti (junior synonym of *Labidus nigrescens*), by subsequent designation of Ashmead, 1906.

*Neivamyrmex* is the most species-rich and widely distributed genus of New World army ants. The biology of the vast majority of the 130 described species is unknown, but *Neivamyrmex nigrescens* has become one of the best studied dorylines.

#### Diagnosis.

***Worker.*** *Neivamyrmex* have 12-segmented antennae, propodeal spiracle high on the sclerite, lack conspicuous propodeal lobes, pygidium small and without a central impressed field, waist two-segmented, and pretarsal claws without a tooth. The simple claws distinguish *Neivamyrmex* workers from all other New World army ant genera (*Cheliomyrmex*, *Eciton*, *Labidus*, and *Nomamyrmex*). *Aenictus* in the Old World will also match some of these diagnostic characters but workers of this genus never have more than 10 antennal segments.

***Male.*** *Neivamyrmex* males share the army ant-like habitus with other members of the *Eciton* genus-group. See discussion under *Cheliomyrmex* male diagnosis for characters differentiating New World army ant males from those of the Old World. Among the New World army ants, *Neivamyrmex* can most reliably distinguished by a combination of apex penisvalvae without setae, no dense setation on gaster, and abdominal segment II (petiole) without a deeply notched or concave surface. The bare penisvalvae are shared only with *Eciton* and *Nomamyrmex* but the former always has a deeply excavated petiole and the latter has conspicuous tufts of dense setae on the gaster.

#### Description.

***Worker.*** *Head*: Antennae with 12 segments. Apical antennal segment not enlarged, not broader and longer than two preceding segments combined. Clypeus with cuticular apron. Lateroclypeal teeth absent. Parafrontal ridges reduced. Torulo-posttorular complex vertical. Antennal scrobes absent. Labrum with median notch or concavity. Proximal face of stipes projecting beyond inner margin of sclerite, concealing prementum when mouthparts fully closed. Maxillary palps 2-segmented. Labial palps 3- or 2-segmented. Mandibles triangular, with teeth. Eyes absent or present, composed of few poorly defined ommatidia. Ocelli absent. Head capsule with differentiated vertical posterior surface above occipital foramen; differentiation sometimes weak. Ventrolateral margins of head without lamella or ridge extending towards mandibles and beyond carina surrounding occipital foramen. Posterior head corners dorsolaterally immarginate. Carina surrounding occipital foramen ventrally absent. *Mesosoma*: Pronotal flange not separated from collar by distinct ridge or separated by ridge that is low on pronotum. Promesonotal connection with Pronotomesopleural suture completely fused or Pronotomesopleural suture weakly differentiated, immobile. Pronotomesopleural suture completely fused or unfused partway to notal surface. Mesometapleural groove not impressed to weakly impressed. Transverse groove dividing mesopleuron absent or present. Pleural endophragmal pit concavity present. Mesosoma dorsolaterally immarginate. Metanotal depression or groove on mesosoma absent. Metanotal depression or groove on mesosoma present. Propodeal spiracle situated high on sclerite. Propodeal declivity without distinct dorsal edge or margin and rectangular in posterior view. Metapleural gland with bulla visible through cuticle. Propodeal lobes present, short. *Metasoma*: Petiole anterodorsally immarginate, dorsolaterally immarginate, and laterally above spiracle immarginate. Helcium in relation to tergosternal Pronotomesopleural suture placed at posttergite and axial. Prora forming a simple U-shaped margin or V-shaped protrusion. Spiracle openings of abdominal segments IV–VI circular, oval, or slit-shaped. Abdominal segment III anterodorsally immarginate and dorsolaterally immarginate. Abdominal segment III about half size of succeeding segment IV, which is strongly constricted at presegmental portion (binodal waist). Girdling constriction of segment IV present, i.e. pre- and postsclerites distinct. Cinctus of abdominal segment IV gutter-like and sculptured but not cross-ribbed. Abdominal segment IV conspicuously largest segment. Abdominal tergite IV not folding over sternite, and anterior portions of sternite and tergite equally well visible in lateral view. Girdling constriction between pre- and posttergites of abdominal segments V and VI absent. Girdling constriction between pre- and poststernites of abdominal segments V and VI absent. Pygidium small, reduced to narrow strip, without impressed medial field and simple, usually not armed with cuticular spines or modified setae but occasionally with one or two pairs of thick modified setae. Hypopygium unarmed. *Legs*: Mid tibia with single pectinate spur. Hind tibia with single pectinate spur. Hind basitarsus not widening distally, circular in cross-section. Posterior flange of hind coxa not produced as raised lamella. Metatibial gland absent to conspicuous patch of whitish cuticle occupying at least half of tibia length. Metabasitarsal gland absent. Hind pretarsal claws simple. *Polymorphism*: Monomorphic to polymorphic.

***Male.*** *Head*: Antennae with 13 segments. Clypeus without cuticular apron. Parafrontal ridges absent. Torulo-posttorular complex vertical. Maxillary palps 2-segmented. Labial palps 3- or 2-segmented. Mandibles falcate. Ventrolateral margins of head without lamella or ridge extending towards mandibles and beyond carina surrounding occipital foramen. Carina surrounding occipital foramen ventrally absent. *Mesosoma*: Pronotal flange not separated from collar by distinct ridge. Notauli absent. Transverse groove dividing mesopleuron absent. Propodeal declivity reduced, without distinct dorsal edge or margin. Metapleural gland opening absent. Propodeal lobes absent or present. *Metasoma*: Petiole anterodorsally immarginate, dorsolaterally immarginate, and laterally above spiracle immarginate. Helcium in relation to tergosternal Pronotomesopleural suture placed at posttergite and axial. Prora simple, not delimited by carina. Spiracle openings of abdominal segments IV–VI oval or slit-shaped. Abdominal segment III more than half size of succeeding segment IV; latter weakly constricted at presegmental portion (uninodal waist). Girdling constriction of segment IV absent, i.e. pre- and postsclerites indistinct. Cinctus of abdominal segment IV absent, not impressed. Girdling constriction between pre- and postsclerites of abdominal segments V and VI absent. Abdominal segment IV not conspicuously largest segment. Abdominal sternite VII simple. Abdominal sternite IX distally armed with two spines, often with additional projections such as medial spine or paired median denticles, with lateral apodemes longer than much reduced medial apodeme, directed anteriorly (towards head). *Genitalia*: Cupula very long, nearing or surpassing length of rest of genital capsule and of approximately equal length on both dorsal and ventral surfaces. Basimere narrowly fused to telomere, with sulcus visible at least partway through junction, and ventrally with left and right arms abutting. Telomere expanded at apex. Volsella narrow, hook-shaped or laterally flattened, triangular in lateral view, narrowing towards tip. Penisvalva not flattened at apex, expanded. *Legs*: Mid tibia with single pectinate spur. Hind tibia with single pectinate spur. Posterior flange of hind coxa not produced as raised lamella. Metatibial gland absent. Metabasitarsal glands absent. Hind pretarsal claws simple or each armed with a tooth. *Wings*: Tegula present, broad, demiovate in shape. Vein C in fore wing present. Pterostigma broad. Abscissa R·f3 present, running toward distal wing margin and enclosing cell with Rs·f5. Abscissae Rs·f2–3 present, connecting with Rs+M&M·f2. Cross-vein 2r-rs present, differentiated from Rs·f4 by presence of Rs·f2–3. Abscissae Rs·f4–5 differentiated into Rs·f4 and Rs·f5 by 2rs-m. Abscissa M·f2 in fore wing present, separated from Rs+M by Rs·f2. Abscissa M·f4 in fore wing present, reaching wing margin. Cross-vein 1m-cu in fore wing present. Cross-vein cu-a in fore wing present, arising from Cu and distal to, at or near M·f1. Vein Cu in fore wing present, with both branches Cu1 and Cu2. Vein A in fore wing with abscissae A·f1 and A·f2 present. Vein C in hind wing present. Vein R in hind wing present, reaching distal wing margin. Vein Sc+R in hind wing present. Abscissa Rs·f1 in hind wing present, shorter than 1rs-m. Abscissa Rs·f2 in hind wing present, reaching wing margin. Cross-vein 1rs-m in hind wing present, about as long as M·f1. Vein M+Cu in hind wing present. Abscissa M·f1 in hind wing present. Abscissa M·f2 in hind wing present. Cross-vein cu-a in hind wing present. Vein Cu in hind wing present. Vein A in hind wing with abscissae A·f1 and A·f2 present.

***Gyne.*** Dichthadiiform, blind or with very small eyes, without ocelli. Known for several species. See e.g. Watkins (1972) and Wheeler (1921) for descriptions and illustrations of *Neivamyrmex* gynes.

***Larva.*** Described in Wheeler and Wheeler 1984.

#### Distribution.

Central and southern United States, south to central Argentina.

#### Taxonomy and phylogeny.

First species now classified in *Neivamyrmex* were described from males by William Shuckard in his 1840 series ‘Monograph of the Dorylidae’. Borgmeier later erected the genus (Borgmeier 1940) and cemented its future use with his classification of New World army ants presented in his monographs (Borgmeier 1953, 1955). Borgmeier (1955) also presented an internal classification for the genus with fourteen informal species groups, including five based solely on males as, typically for army ants, numerous names have been proposed for males without associations with workers. Later work on *Neivamyrmex* taxonomy has been dominated by Julian Watkins who published many new isolated species descriptions and also provided updated identification resources for this and other New World army ant genera, first for New World in general (Watkins 1976) and later for United States in particular (Watkins 1985). This latter resource was recently updated with a publication by Snelling and Snelling (2007). *Neivamyrmex* is the most species-rich of the *Eciton* genus-group with 127 extant species. The genus is the sister group to the clade of the other four New World army ant genera and is monophyletic (Borowiec, in prep.). There is no comprehensive internal phylogeny, but preliminary data indicates that some of the species groups proposed by Borgmeier are not monophyletic (Borowiec, in prep.).

#### Biology.

The majority of species has never been studied in any detail, and much of what we know comes from the observations made on one relatively common species, *Neivamyrmex nigrescens*, studied extensively by Howard Topoff and his students (Gotwald 1995). The biology of other species has been summarized by Rettenmeyer (1963).

If *Neivamyrmex nigrescens* is representative of this genus, the lineage’s habits are similar to those of other New World army ants. There are marked nomadic and statary phases, lasting about 16 and 20 days, respectively. The colonies are of moderate size, containing 80,000 to 140,000 workers (Rettenmeyer 1963; Topoff et al. 1980 estimate 10,000–50,000) and bivouacs are subterranean. The prey consists of almost exclusively other ants’ brood.

Nesting sites of *Neivamyrmex* are rarely observed. Rettenmeyer (1963) reported that although known bivouac sites of *Neivamyrmex nigrescens* in Kansas are typically at least 1 meter below the surface, two bivouacs were discovered that were completely contained within the upper 30 cm of the soil. Emigration behavior in this species has been very well described. Environmental factors, such as prey availability/density and nest site availability, influence the emigration behavior (Topoff and Mirenda 1980, Mirenda and Topoff 1980). *Neivamyrmex carolinensis* and *Neivamyrmex kiowapache* are unusual among army ants in that they are the only species known to be polygynous, with colonies reported to contain over a dozen queens (Rettenmeyer and Watkins 1978, see also Snelling and Snelling 2007). The queens of *Neivamyrmex kiowapache* have been shown to mate with much lower frequency than other army ants. This is in accordance with the prediction that the costly multiple matings will be reduced or lost if genetic diversity of workers can be achieved through polygyny (Kronauer and Boomsma 2007b).

The foraging biology of *Neivamyrmex nigrescens* in Arizona was studied in detail by Mirenda et al. (1980). They reported that these army ants forage at night and raid nests of many other ants and termites. The ants in the genus *Pheidole* were shown to be the preferred prey, being taken twice as often as expected based on colony density. *Pogonomyrmex*, *Forelius*, and *Myrmecocystus* were reported to be avoided. The authors observed temporal variation in prey composition, noting that as the season progressed and conditions became drier, many of the prey *Pheidole* species ceased activity and sealed their nests. *Neivamyrmex nigrescens* was then observed to rely more heavily on *Novomessor cockerelli* as prey.

Several *Neivamyrmex* species can occur sympatrically, and it is likely that a diversity of prey preferences exists in the genus. Mirenda et al. (1980) also observed multiple raids of *Neivamyrmex harrisii*, sympatric with *Neivamyrmex nigrescens*, and noted that *Solenopsis xyloni* was the only species being attacked.

*Neivamyrmex nigrescens* uses both tactile and chemical cues in orientation (Topoff and Lawson 1979).

Many species of ants respond to a *Neivamyrmex* attack by nest evacuation and this behavior has been highlighted as a tool for collecting colonies of soil-nesting species that are normally difficult to excavate. Smith and Haight (2008) showed that 150-300 *Neivamyrmex nigrescens* workers poured into the nest entrance of *Novomessor cockerelli* induced evacuation of a mature colony, including brood and queen.

#### Species of *Neivamyrmex*

*Neivamyrmex adnepos* (Wheeler, W. M., 1922b): Trinidad and Tobago

*Neivamyrmex agilis* Borgmeier, 1953: Mexico

*Neivamyrmex albacorpus* Varela-Hernández and Castaño-Meneses, 2011: Mexico

*Neivamyrmex alfaroi* (Emery, 1890): Costa Rica

*Neivamyrmex andrei* (Emery, 1901b): Mexico

*Neivamyrmex angulimandibulatus* Watkins, 1974: Mexico

*Neivamyrmex angustinodis* (Emery, 1888): Argentina

*Neivamyrmex antillanus* (Forel, 1897): Grenada

*Neivamyrmex asper* Borgmeier, 1955: Costa Rica

*Neivamyrmex balzani* (Emery, 1894): Bolivia

*Neivamyrmex baylori* Watkins, 1973: United States

*Neivamyrmex bohlsi* (Emery, 1896c): Paraguay

*Neivamyrmex bruchi* (Forel, 1912c): Argentina

*Neivamyrmex bureni* (Enzmann, E.V., 1952): Peru

*Neivamyrmex californicus* (Mayr, 1870): United States

*Neivamyrmex carettei* (Forel, 1913d): Argentina

*Neivamyrmex carinifrons* Borgmeier, 1953: Brazil

*Neivamyrmex carolinensis* (Emery, 1894): United States

*Neivamyrmex chamelensis* Watkins, 1986: Mexico

*Neivamyrmex clavifemur* Borgmeier, 1953: Brazil

*Neivamyrmex cloosae* (Forel, 1912a): Mexico

*Neivamyrmex coeca* (Buckley, 1867): United States

*Neivamyrmex compressinodis* Borgmeier, 1953: Costa Rica

*Neivamyrmex cornutus* Watkins, 1975a: Mexico

*Neivamyrmex crassiscapus* Watkins, 1990: Mexico

*Neivamyrmex cratensis* Borgmeier, 1953: Brazil

*Neivamyrmex cristatus* (André, 1889): ‘Amérique du Sud’

*Neivamyrmex curvinotus* Watkins, 1994: Peru

*Neivamyrmex densepunctatus* (Borgmeier, 1933): Brazil

*Neivamyrmex detectus* Borgmeier, 1953: Brazil

*Neivamyrmex diabolus* (Forel, 1912a): Mexico

*Neivamyrmex diana* (Forel, 1912c): Brazil

*Neivamyrmex digitistipus* Watkins, 1975b: Costa Rica

*Neivamyrmex diversinodis* (Borgmeier, 1933): Bolivia

*Neivamyrmex dorbignii* (Shuckard, 1840b): No locality given

†*Neivamyrmex ectopus* Wilson, 1985: Dominican amber

*Neivamyrmex emersoni* (Wheeler, W. M., 1921): Guyana

*Neivamyrmex emeryi* (Santschi, 1921b): Bolivia, Peru

*Neivamyrmex erichsonii* (Westwood, 1842): Brazil

*Neivamyrmex falcifer* (Emery, 1900b): Bolivia

*Neivamyrmex foveolatus* Borgmeier, 1953: Panama

*Neivamyrmex fumosus* (Forel, 1913d): Guatemala

*Neivamyrmex fuscipennis* (Smith, M.R., 1942b): United States

*Neivamyrmex genalis* Borgmeier, 1953: Bolivia

*Neivamyrmex gibbatus* Borgmeier, 1953: Costa Rica

*Neivamyrmex goeldii* (Forel, 1901d): Brazil

*Neivamyrmex graciellae* (Mann, 1926): Mexico

*Neivamyrmex gracilis* Borgmeier, 1955: Brazil

*Neivamyrmex gradualis* Borgmeier, 1953: Brazil

*Neivamyrmex guerinii* (Shuckard, 1840d): Brazil

*Neivamyrmex guyanensis* (Santschi, 1916): French Guiana

*Neivamyrmex halidaii* (Shuckard, 1840a): Brazil

*Neivamyrmex harrisii* (Haldeman, 1852): United States

*Neivamyrmex hetschkoi* (Mayr, 1886a): Brazil

*Neivamyrmex hopei* (Shuckard, 1840b): Brazil

*Neivamyrmex humilis* (Borgmeier, 1939): Costa Rica

*Neivamyrmex iheringi* (Forel, 1908): Brazil

*Neivamyrmex imbellis* (Emery, 1900b): Peru, Venezuela

*Neivamyrmex impudens* (Mann, 1922): Honduras

*Neivamyrmex inca* (Santschi, 1921b): Peru

*Neivamyrmex inflatus* Borgmeier, 1958: Mexico

*Neivamyrmex iridescens* Borgmeier, 1950: Guyana

*Neivamyrmex jerrmanni* (Forel, 1901e): Paraguay

*Neivamyrmex kiowapache* Snelling, G.C. and Snelling, R.R., 2007: United States

*Neivamyrmex klugii* (Shuckard, 1840b): Saint Vincent and the Grenadines

*Neivamyrmex klugii distans* Borgmeier, 1953: Costa Rica

*Neivamyrmex kuertii* (Enzmann, E.V., 1952): Peru

*Neivamyrmex laevigatus* (Borgmeier, 1948): Argentina

*Neivamyrmex latiscapus* (Emery, 1901b): Brazil

*Neivamyrmex legionis* (Smith, F., 1855): Argentina

*Neivamyrmex leonardi* (Wheeler, W. M., 1915a): United States

*Neivamyrmex leptognathus* (Emery, 1900b): Bolivia

*Neivamyrmex lieselae* (Forel, 1913d): Argentina

*Neivamyrmex longiscapus* Borgmeier, 1953: Costa Rica

*Neivamyrmex macrodentatus* (Menozzi, 1931): Costa Rica

*Neivamyrmex mandibularis* (Smith, M.R., 1942b): United States

*Neivamyrmex manni* (Wheeler, W. M., 1914): Mexico

*Neivamyrmex maroccanus* (Santschi, 1926b): Morocco (labeling error)

*Neivamyrmex maxillosus* (Emery, 1900b): Brazil

*Neivamyrmex megathrix* Kempf, 1961: Suriname

*Neivamyrmex melanocephalus* (Emery, 1895d): Mexico

*Neivamyrmex melshaemeri* (Haldeman, 1852): United States

*Neivamyrmex micans* Borgmeier, 1953: Brazil

*Neivamyrmex microps* Borgmeier, 1955: United States

*Neivamyrmex minensis* (Borgmeier, 1928): Brazil

*Neivamyrmex minor* (Cresson, 1872): United States

*Neivamyrmex modestus* (Borgmeier, 1933): Brazil

*Neivamyrmex mojave* (Smith, M.R., 1943): United States

*Neivamyrmex moseri* Watkins, 1969: United States

*Neivamyrmex ndeh* Snelling, G.C. and Snelling, R.R., 2007: United States

*Neivamyrmex nigrescens* (Cresson, 1872): United States

*Neivamyrmex nordenskioldii* (Holmgren, 1908): Peru

*Neivamyrmex nyensis* Watkins, 1977: United States

*Neivamyrmex opacithorax* (Emery, 1894): United States

*Neivamyrmex orthonotus* (Borgmeier, 1933): Brazil

*Neivamyrmex pacificus* Borgmeier, 1955: Peru

*Neivamyrmex pauxillus* (Wheeler, W. M., 1903a): United States

*Neivamyrmex perplexus* Borgmeier, 1953: Brazil

*Neivamyrmex pertii* (Shuckard, 1840b) : Brazil

*Neivamyrmex physognathus* (Emery, 1900b): Bolivia

*Neivamyrmex pilosus* (Smith, F., 1858): Brazil

*Neivamyrmex piraticus* Borgmeier, 1953: Brazil

*Neivamyrmex planidens* Borgmeier, 1953: Ecuador

*Neivamyrmex planidorsus* (Emery, 1906): Paraguay

*Neivamyrmex postangustatus* (Borgmeier, 1934): Suriname

*Neivamyrmex postcarinatus* Borgmeier, 1953: Panama

*Neivamyrmex pseudops* (Forel, 1909a): Paraguay

*Neivamyrmex puerulus* Borgmeier, 1955: Panama

*Neivamyrmex pulchellus* Borgmeier, 1955: Panama

*Neivamyrmex pullus* Borgmeier, 1953: Panama

*Neivamyrmex punctaticeps* (Emery, 1894): Brazil

*Neivamyrmex quadratoocciputus* Watkins, 1975c: El Salvador

*Neivamyrmex radoszkowskii* (Emery, 1900b): Peru

*Neivamyrmex raptor* (Forel, 1911b): Brazil

*Neivamyrmex romandii* (Shuckard, 1840b): Brazil

*Neivamyrmex rosenbergi* (Forel, 1911d): Ecuador

*Neivamyrmex rugulosus* Borgmeier, 1953: Mexico

*Neivamyrmex scutellaris* Borgmeier, 1953: Panama

*Neivamyrmex shuckardi* (Emery, 1900b): Paraguay

*Neivamyrmex spatulatus* (Borgmeier, 1939): Costa Rica

*Neivamyrmex spoliator* (Forel, 1899): Costa Rica

*Neivamyrmex sulcatus* (Mayr, 1868): Argentina

*Neivamyrmex sumichrasti* (Norton, 1868): Mexico

*Neivamyrmex swainsonii* (Shuckard, 1840a): Brazil

*Neivamyrmex tenuis* Borgmeier, 1953: Brazil

*Neivamyrmex texanus* Watkins, 1972: United States

*Neivamyrmex tristis* (Forel, 1901e): Mexico

*Neivamyrmex vicinus* Borgmeier, 1953: Brazil

*Neivamyrmex walkerii* (Westwood, 1842): Brazil

*Neivamyrmex wilsoni* Snelling, G.C. and Snelling, R.R., 2007: United States

### Neocerapachys gen. n.

Taxon classificationAnimaliaHymenopteraFormicidae

http://zoobank.org/E28AB8E9-C01D-451F-B54C-6295CF5D9F2F

#### Type-species.

Cerapachys (Cerapachys) neotropicus, by present designation.

*Neocerapachys* is a rarely encountered Neotropical lineage with unknown habits.

#### Diagnosis.

***Worker.*** *Neocerapachys* can be recognized by a combination of relatively low-positioned propodeal spiracle, propodeal lobes present, constriction present between abdominal segments III and IV, middle tibiae with a single spur, pretarsal claws unarmed, petiole dorsolaterally rounded (not marginate), constriction absent from between abdominal segments IV, V, and VI, pronotomesopleural Pronotomesopleural suture fused, helcium axial, abdominal segment III anterodorsally often marginate, and two spots where pilosity is denser than the surrounding hairs present laterally on abdominal tergite IV. *Neocerapachys* is superficially very similar to certain species of *Parasyscia* of the Old World but the latter never has lateral clumps of hair on abdominal tergite IV and its metapleural gland trench is broader than in *Neocerapachys*. Palp formulae also differ in these two lineages with 3,3 in *Neocerapachys* and 3,2 or 2,2 in *Parasyscia*. The neotropical *Sphinctomyrmex* shares several characters with *Neocerapachys* but is distinguished by constrictions between abdominal segments IV, V, and VI.

***Male.*** Males of *Neocerapachys* possess well-developed propodeal lobes, mid and hind tibiae each with one spur, C and R·f3 veins in the fore wing, Rs·f2–3 abscissae present, cross-vein 2rs-m absent, third antennal segment conspicuously the shortest segment, and conspicuously marginate propodeal declivity. This combination will serve to distinguish it from all other lineages. Indomalayan *Cerapachys* is a relatively similar genus but it differs in longer, normally developed third antennal segment and eyes situated further away from mandibular insertions. In the Neotropics, *Sphinctomyrmex* males have similar wing venation but are easily told apart by constrictions visible between abdominal segments IV, V, and VI.

#### Description.

***Worker.*** *Head*: Antennae with 12 segments. Apical antennal segment conspicuously enlarged, much broader than and longer than two preceding segments combined. Clypeus without cuticular apron. Lateroclypeal teeth absent. Parafrontal ridges reduced. Torulo-posttorular complex vertical. Antennal scrobes absent. Labrum with median notch or concavity. Proximal face of stipes projecting beyond inner margin of sclerite, concealing prementum when mouthparts fully closed. Maxillary palps 3-segmented. Labial palps 3-segmented. Mandibles triangular, with teeth. Eyes present, composed of 1–5 ommatidia. Ocelli absent. Head capsule with differentiated vertical posterior surface above occipital foramen. Ventrolateral margins of head with cuticular ridge extending towards mandibles and beyond carina surrounding occipital foramen. Posterior head corners dorsolaterally immarginate. Carina surrounding occipital foramen ventrally present. *Mesosoma*: Pronotal flange separated from collar by distinct ridge. Promesonotal connection with Pronotomesopleural suture completely fused. Pronotomesopleural suture visible, unfused partway to notal surface. Mesometapleural groove weakly impressed. Transverse groove dividing mesopleuron present. Pleural endophragmal pit concavity present. Mesosoma dorsolaterally immarginate. Metanotal depression or groove on mesosoma absent. Propodeal spiracle situated low on sclerite. Propodeal declivity with distinct dorsal edge or margin and rectangular in posterior view. Metapleural gland with bulla partially obscured but often discernable through cuticle. Propodeal lobes present, well developed. *Metasoma*: Petiole anterodorsally marginate, dorsolaterally immarginate, and laterally above spiracle marginate. Helcium in relation to tergosternal Pronotomesopleural suture placed at posttergite and axial. Prora forming a simple U-shaped margin. Spiracle openings of abdominal segments IV–VI circular. Abdominal segment III anterodorsally marginate and dorsolaterally immarginate. Abdominal segment III more than half size of succeeding segment IV, which is weakly constricted at presegmental portion (uninodal waist). Girdling constriction of segment IV present, i.e. pre- and postsclerites distinct. Cinctus of abdominal segment IV gutter-like and cross-ribbed. Abdominal segment IV not conspicuously largest segment. Abdominal tergite IV not folding over sternite, and anterior portions of sternite and tergite equally well visible in lateral view. Girdling constriction between pre- and posttergites of abdominal segments V and VI absent. Girdling constriction between pre- and poststernites of abdominal segments V and VI absent. Pygidium large, with impressed medial field, and armed with modified setae. Hypopygium unarmed. *Legs*: Mid tibia with single pectinate spur. Hind tibia with single pectinate spur. Hind basitarsus not widening distally, circular in cross-section. Posterior flange of hind coxa not produced as raised lamella. Metatibial gland present as oval patch of whitish cuticle. Metabasitarsal gland absent. Hind pretarsal claws simple. *Polymorphism*: Monomorphic.

***Male.*** *Head*: Antennae with 13 segments. Clypeus with cuticular apron. Parafrontal ridges present. Torulo-posttorular complex vertical. Maxillary palps 4-segmented. Labial palps 3-segmented. Mandibles triangular, edentate. Ventrolateral margins of head with cuticular ridge extending towards mandibles and beyond carina surrounding occipital foramen. Carina surrounding occipital foramen ventrally present. *Mesosoma*: Pronotal flange separated from collar by distinct ridge. Notauli present. Transverse groove dividing mesopleuron present. Propodeal declivity with distinct dorsal edge or margin. Metapleural gland opening absent. Propodeal lobes present. *Metasoma*: Petiole anterodorsally marginate, dorsolaterally immarginate, and laterally above spiracle marginate. Helcium in relation to tergosternal Pronotomesopleural suture placed at Pronotomesopleural suture and supraaxial. Prora forming a simple U-shaped margin. Spiracle openings of abdominal segments IV–VI circular. Abdominal segment III more than half size of succeeding segment IV or about half size; latter weakly or strongly constricted at presegmental portion (transitional between uninodal waist and binodal waist). Girdling constriction of segment IV present, i.e. pre- and postsclerites distinct. Cinctus of abdominal segment IV gutter-like and cross-ribbed. Girdling constriction between pre- and postsclerites of abdominal segments V and VI absent. Abdominal segment IV not conspicuously largest segment. Abdominal sternite VII simple. Abdominal sternite IX distally armed with two spines curving dorsally at apices, with lateral apodemes about as long as medial apodeme, directed anteriorly (towards head). *Genitalia*: Cupula long relative to rest of genital capsule and of approximately equal length on both dorsal and ventral surfaces. Basimere broadly fused to telomere, basimere with no sulcus trace at junction, and ventrally with left and right arms abutting. Telomere gradually tapering toward apex. Volsella laterally flattened, at apex with dorsal lobe and hooked ventrally. Penisvalva laterally compressed, rounded at apex. *Legs*: Mid tibia with single pectinate spur. Hind tibia with single pectinate spur. Posterior flange of hind coxa not produced as raised lamella. Metatibial gland present as oval patch of whitish cuticle. Metabasitarsal glands absent. Hind pretarsal claws simple. *Wings*: Tegula present, broad, demiovate in shape. Vein C in fore wing present. Pterostigma broad. Abscissa R·f3 present and running toward distal wing margin and enclosing marginal cell with Rs·f5 or not. Abscissae Rs·f2–3 present, connecting with Rs+M&M·f2 or disconnected from Rs+M. Cross-vein 2r-rs absent. Abscissae Rs·f4–5 present, fused in absence of 2rs-m. Abscissa M·f2 in fore wing contiguous with Rs+M. Abscissa M·f4 in fore wing present, not reaching wing margin. Cross-vein 1m-cu in fore wing present. Cross-vein cu-a in fore wing present, arising from M+Cu and proximal to M·f1. Vein Cu in fore wing present, with both branches Cu1 and Cu2. Vein A in fore wing with abscissae A·f1 and A·f2 present. Vein C in hind wing absent. Vein R in hind wing present, extending past Sc+R but not reaching distal wing margin. Vein Sc+R in hind wing present. Abscissa Rs·f1 in hind wing present, shorter than 1rs-m. Abscissa Rs·f2 in hind wing present, not reaching wing margin. Cross-vein 1rs-m in hind wing present, about as long as M·f1. Vein M+Cu in hind wing present. Abscissa M·f1 in hind wing present. Abscissa M·f2 in hind wing absent or present. Cross-vein cu-a in hind wing present. Vein Cu in hind wing present. Vein A in hind wing with abscissae A·f1 and A·f2 present.

***Gyne.*** Apparently alate or ergatoid with well-developed mesosomal sutures; with large eyes and three ocelli. This interpretation is based on one gyne specimen from Venezuela (John T. Longino personal collection, LACMENT 142669).

***Larva.*** Not described. Presence of cocoons unknown.

#### Distribution.

This lineage ranges from Costa Rica south to southern Brazil and apparently is not very species-rich with only two species described.

#### Taxonomy and phylogeny.

Both currently named species have been described under *Cerapachys* and later discussed by Brown (1975) as similar to the ‘*dohertyi*-*cribrinodis* group’ (here *Parasyscia*). Brown even speculated that *Neocerapachys neotropicus* had been introduced from the Old World, but molecular data (Brady et al. 2014, Borowiec, in prep.) prove that the resemblance to *Parasyscia* is superficial.

The exact phylogenetic position of *Neocerapachys* is not known with certainty, but in molecular analyses based on genomic data it is a part of a large New World clade that includes *Acanthostichus*, *Cylindromyrmex*, *Leptanilloides*, *Sphinctomyrmex*, and the *Eciton* genus-group (Borowiec, in prep.).

#### Biology.

I am not aware of any nest collections or observations of behavior of *Neocerapachys*.

#### Species of *Neocerapachys*

*Neocerapachys neotropicus* (Weber, 1939): Trinidad and Tobago, **comb. n.**

*Neocerapachys splendens* (Borgmeier, 1957): Brazil, **comb. n.**

### Nomamyrmex

Taxon classificationAnimaliaHymenopteraFormicidae

Borgmeier, 1936

#### Type-species.

*Eciton crassicornis* (junior synonym of *Labidus esenbeckii*), by original designation.

*Nomamyrmex* is a relatively commonly observed genus with only two species and two additional subspecies recognized. It is the only army ant genus that has been reported to successfully attack well-defended and often enormous colonies of *Atta* leaf cutter ants.

#### Diagnosis.

***Worker.*** The workers of *Nomamyrmex* are easily recognized by a combination of highly positioned spiracle and lack of pronounced propodeal lobes, propodeum armed with cuticular projections, two-segmented waist, armed pretarsal claws, and absence of metatibial gland. The lack of conspicuous lighter area of cuticle on the inner side of hind tibia (the metatibial gland) distinguishes this genus from all other *Eciton* genus-group ants except for some *Neivamyrmex*, but those always have simple pretarsal claws.

***Male.*** *Nomamyrmex* males possess traits characteristic of New World army ants; see discussion under *Cheliomyrmex* for characters distinguishing New World army ant males from those of the Old World. *Nomamyrmex* is also easily told apart from other New World army ant males by its dense tufts of very long hairs present on the gaster. *Eciton setigaster* is one species that could be mistaken for a *Nomamyrmex*, but the setae on its gaster are not as dense or as long, not approaching front femur length.

#### Description.

***Worker.*** *Head*: Antennae with 12 segments. Apical antennal segment not enlarged, not broader and longer than two preceding segments combined. Clypeus with cuticular apron. Lateroclypeal teeth absent. Parafrontal ridges reduced. Torulo-posttorular complex vertical. Antennal scrobes absent. Labrum with median notch or concavity. Proximal face of stipes projecting beyond inner margin of sclerite, concealing prementum when mouthparts fully closed. Maxillary palps 2-segmented. Labial palps 3-segmented. Mandibles triangular, with teeth. Eyes present, appearing as single large and convex ommatidium, in reality composed from fused ommatidia. Ocelli absent. Head capsule with differentiated vertical posterior surface above occipital foramen. Ventrolateral margins of head without lamella or ridge extending towards mandibles and beyond carina surrounding occipital foramen. Posterior head corners dorsolaterally immarginate. Carina surrounding occipital foramen ventrally absent. *Mesosoma*: Pronotal flange separated from collar by distinct ridge or not. Promesonotal connection with Pronotomesopleural suture completely fused. Pronotomesopleural suture visible, unfused partway to notal surface. Mesometapleural groove not impressed. Transverse groove dividing mesopleuron absent. Pleural endophragmal pit concavity present. Mesosoma dorsolaterally immarginate. Metanotal depression or groove on mesosoma present. Propodeal spiracle situated high on sclerite. Propodeal declivity with or without distinct dorsal edge or margin and rectangular in posterior view. Metapleural gland with bulla visible through cuticle. Propodeal lobes present, short. *Metasoma*: Petiole anterodorsally marginate, dorsolaterally immarginate, and laterally above spiracle immarginate. Helcium in relation to tergosternal Pronotomesopleural suture placed at Pronotomesopleural suture and axial. Prora forming a simple U-shaped margin or V-shaped protrusion. Spiracle openings of abdominal segments IV–VI oval to slit-shaped. Abdominal segment III anterodorsally immarginate and dorsolaterally immarginate. Abdominal segment III about half size of succeeding segment IV, which is strongly constricted at presegmental portion (binodal waist). Girdling constriction of segment IV present, i.e. pre- and postsclerites distinct. Cinctus of abdominal segment IV gutter-like and sculptured but not cross-ribbed. Abdominal segment IV conspicuously largest segment. Abdominal tergite IV not folding over sternite, and anterior portions of sternite and tergite equally well visible in lateral view. Girdling constriction between pre- and posttergites of abdominal segments V and VI absent. Girdling constriction between pre- and poststernites of abdominal segments V and VI absent. Pygidium small, reduced to narrow strip, without impressed medial field and simple, not armed with cuticular spines or modified setae. Hypopygium unarmed. *Legs*: Mid tibia with single pectinate spur. Hind tibia with single pectinate spur. Hind basitarsus not widening distally, circular in cross-section. Posterior flange of hind coxa not produced as raised lamella. Metatibial gland absent. Metabasitarsal gland absent. Hind pretarsal claws each armed with a tooth. *Polymorphism*: Polymorphic.

***Male.*** *Head*: Antennae with 13 segments. Clypeus without cuticular apron. Parafrontal ridges absent. Torulo-posttorular complex vertical. Maxillary palps 2-segmented. Labial palps 3- or 2-segmented. Mandibles falcate. Ventrolateral margins of head without lamella or ridge extending towards mandibles and beyond carina surrounding occipital foramen. Carina surrounding occipital foramen ventrally absent. *Mesosoma*: Pronotal flange not separated from collar by distinct ridge. Notauli absent. Transverse groove dividing mesopleuron absent. Propodeal declivity reduced, without distinct dorsal edge or margin. Metapleural gland opening absent. Propodeal lobes present. *Metasoma*: Petiole anterodorsally immarginate, dorsolaterally immarginate, and laterally above spiracle immarginate. Helcium in relation to tergosternal Pronotomesopleural suture placed at Pronotomesopleural suture and axial. Prora forming a simple U-shaped margin. Spiracle openings of abdominal segments IV–VI slit-shaped. Abdominal segment III more than half size of succeeding segment IV; latter weakly constricted at presegmental portion (uninodal waist). Girdling constriction of segment IV present, i.e. pre- and postsclerites distinct. Cinctus of abdominal segment IV gutter-like and cross-ribbed. Girdling constriction between pre- and postsclerites of abdominal segments V and VI absent. Abdominal segment IV not conspicuously largest segment. Abdominal sternite VII simple. Abdominal sternite IX distally armed with two spines, with lateral apodemes about as long as medial apodeme, directed anteriorly (towards head). *Genitalia*: Cupula very long, nearing or surpassing length of rest of genital capsule and of approximately equal length on both dorsal and ventral surfaces. Basimere narrowly fused to telomere, with sulcus discernable at junction, and ventrally with left and right arms abutting. Telomere expanded at apex. Volsella laterally flattened, narrowly triangular in lateral view, narrowing towards tip. Penisvalva curved ventrally at apex, with short dorsal and longer ventral process. *Legs*: Mid tibia with single pectinate spur. Hind tibia with single pectinate spur. Posterior flange of hind coxa not produced as raised lamella. Metatibial gland absent. Metabasitarsal glands absent. Hind pretarsal claws each armed with a tooth. *Wings*: Tegula present, broad, demiovate in shape. Vein C in fore wing present. Pterostigma narrow. Abscissa R·f3 present, running toward distal wing margin and enclosing cell with Rs·f5. Abscissae Rs·f2–3 present, connecting with Rs+M&M·f2. Cross-vein 2r-rs present, differentiated from Rs·f4 by presence of Rs·f2–3. Abscissae Rs·f4–5 differentiated into Rs·f4 and Rs·f5 by 2rs-m. Abscissa M·f2 in fore wing present, separated from Rs+M by Rs·f2. Abscissa M·f4 in fore wing present, reaching wing margin. Cross-vein 1m-cu in fore wing present. Cross-vein cu-a in fore wing present, arising from Cu and distal to, at or near M·f1. Vein Cu in fore wing present, with both branches Cu1 and Cu2. Vein A in fore wing with abscissae A·f1 and A·f2 present. Vein C in hind wing present. Vein R in hind wing present, reaching distal wing margin. Vein Sc+R in hind wing present. Abscissa Rs·f1 in hind wing present, shorter than 1rs-m. Abscissa Rs·f2 in hind wing present, reaching wing margin. Cross-vein 1rs-m in hind wing present, about as long as M·f1. Vein M+Cu in hind wing present. Abscissa M·f1 in hind wing present. Abscissa M·f2 in hind wing present. Cross-vein cu-a in hind wing present. Vein Cu in hind wing present. Vein A in hind wing with abscissae A·f1 and A·f2 present.

***Gyne.*** Dichthadiiform, with falcate mandibles, small eyes, and no ocelli. Known for *Nomamyrmex esenbeckii* (Borgmeier 1958).

***Larva.*** Not described. Cocoons present.

#### Distribution.

Both *Nomamyrmex* species are widely distributed and the genus is found from Texas to northern Argentina.

#### Taxonomy and phylogeny.

The two species of *Nomamyrmex* were known since Westwood described them in 1842, but he treated them under *Labidus*. Borgmeier introduced *Nomamyrmex* as a subgenus of *Eciton* (Borgmeier 1936). Several names have been published for these widely distributed insects but the species-level taxonomy has been in relative stability thanks to the monumental efforts of Borgmeier (1953, 1955) who examined much of the type material available and recognized the extensive synonymy, reducing the number of species to the two originally described by Westwood, *Nomamyrmex esenbeckii* and *Nomamyrmex hartigii*. There is a marked variation in the morphology of *Nomamyrmex esenbeckii* and this led Borgmeier and subsequent authors to recognize three or four subspecies (see Watkins 1977b). Borgmeier reported sympatry of some of those subspecies (Borgmeier 1955, 1958) but recently the view has expressed that this variation is seen in largely allopatric populations with numerous intermediates known and that the subspecies are best treated as synonyms of *esenbeckii* (Gordon Snelling pers. comm., Wild 2007). However, the formal synonymization of two of these subspecies, *Nomamyrmex esenbeckii wilsoni* and *Nomamyrmex esenbeckii mordax* has yet to be made. The species-level taxonomy of *Nomamyrmex* would benefit from a thorough morphometric and molecular phylogenetic study.

Brady et al. (2014) and genomic data (Borowiec, in prep.) recover a well-resolved clade of *Labidus* sister to *Nomamyrmex* plus *Eciton*. It may be noted that Borgmeier (1955: 137) wrote ‘*Nomamyrmex* stands between *Labidus* and *Eciton*’ when referring to the genital morphology of *Nomamyrmex* as showing similarities to the latter two genera.

#### Biology.

Henry Walter Bates was perhaps the first to report on the habits of *Nomamyrmex* in his famous narrative (Bates 1863), describing a ‘(...) very stout-limbed *Eciton*, the *Eciton crassicornis*, whose eyes are sunk in rather deep sockets’ that ‘(...) goes on foraging expeditions like the rest of its tribe, and attacks even the nests of other stinging species (*Myrmica*), but it avoids the light, moving always in concealment under leaves and fallen branches’.

Borgmeier (1955) and Rettenmeyer (1963) summarize what was known about *Nomamyrmex* to date, most observations being on *Nomamyrmex esenbeckii*. The summary below regarding raids and emigrations is based on these resources unless stated otherwise. *Nomamyrmex* presumably forms bivouacs which are always subterranean and have never been directly observed. Based on the durations of emigrations observed, Rettenmeyer (1963) estimated that the colonies must be enormous, perhaps in the excess of a million workers. The diet of these army ants consists mostly of immatures of multiple species of other ants, although they have been observed raiding nests of other social insects, including termites and bees (see also Souza and Moura 2008). It appears that raids are primarily subterranean, although columns of these ants are also observed above ground. The raid columns are narrow, not forming swarms. The raids have been observed both at night and during the day and often last throughout the day. Rettenmeyer reports that *Nomamyrmex esenbeckii* on Barro Colorado, Panama conducted raids mostly during the day but there are reports of the same species raiding at night and being strongly photophobic (Sánchez-Peña and Mueller 2002). Given the large size of the colonies, raids and emigrations can take a very long time and last well over 24 hours (Rettenmeyer 1963, Powell and Clark 2004). Numerous myrmecophiles have been observed in emigration columns, including multiple limulodid beetles riding the emigrating queen. The brood is synchronized.

A remarkable aspect of *Nomamyrmex* biology is the capability to successfully raid the huge colonies of leaf-cutting ants in the genus *Atta*, otherwise mostly ignored by army ants. Most published records of *Nomamyrmex* foraging contain observations of raids on leaf cutters (Swartz 1998, Sánchez-Peña and Mueller 2002 and references therein) and Powell and Clark (2004) conducted the most comprehensive study of interactions between these ants to date. They show that *Nomamyrmex* is capable of successfully raiding both young and mature colonies of *Atta* and that the latter respond in a specific manner to the presence of workers of *Nomamyrmex* but not *Eciton*. The leafcutters defend their nests by mobilizing large numbers of major workers and plugging nest entrances with cut leaves. *Nomamyrmex* and *Atta* workers that directly engage in combat are most often the largest ants in the colonies of both species and the encounters usually result in the ants becoming locked head-to-head. Furthermore, slightly smaller workers of both species also participate in combat but in a slightly different way. On the *Atta* side, they assist in spread-eagling the attacking army ants while the ‘primary combatants’ are locked with their mandibles. On the *Nomamyrmex* side they overrun and sting the leaf-cutter majors to death.

*Nomamyrmex* is capable of inflicting significant damage on a raided *Atta* colony. A subterranean raid on a partially excavated *Atta mexicana* colony was observed where the army ants killed a large proportion of adult *Atta*, including the queen (Rettenmeyer et al. 1983). Swartz (1998) reported that an army ant raid on a young *Atta cephalotes* colony extirpated the leaf-cutters and eventually turned into an emigration, the *Nomamyrmex* colony relocating into the abandoned nest. Powell and Clark (2004) estimated that during one nearly 36-hour raid the *Nomamyrmex* removed over 60,000 brood items from an *Atta cephalotes* colony, possibly over a half of all the brood present in the nest.

#### Species of *Nomamyrmex*

*Nomamyrmex esenbeckii* (Westwood, 1842): Brazil

*Nomamyrmex esenbeckii mordax* (Santschi, 1929): Mexico

*Nomamyrmex esenbeckii wilsoni* (Santschi, 1920a): United States

*Nomamyrmex hartigii* (Westwood, 1842): Brazil

### Ooceraea

Taxon classificationAnimaliaHymenopteraFormicidae

Roger, 1862 gen. rev.

= Cysias Emery, 1902, **syn. n.**

#### Type-species.

*Ooceraea fragosa*, by monotypy.

*Ooceraea* is an Old World lineage that contains a species emerging as the only model organism among dorylines, the clonal *Ooceraea biroi*.

#### Diagnosis.

***Worker.*** The workers of *Ooceraea* can be distinguished by a combination of propodeal spiracle positioned low on the sclerite and pygidium armed with modified setae, antennae with 11 or fewer segments, pronotomesopleural Pronotomesopleural suture developed, two-segmented waist with abdominal segment III strongly tubulated, and no constrictions between abdominal segments IV, V, and VI. The abdominal segment IV is conspicuously the largest and its tergite does not fold over the sternite anteriorly. The habitus of *Ooceraea* is distinctive, with conspicuously differentiated abdominal segment III forming a postpetiole, eyes small or absent, and coarse cuticular sculpturing. Among the non-army ant dorylines that exhibit reduction in antennomere count *Ooceraea* can only be confused with *Syscia* and *Parasyscia*. The former exhibits a conspicuous folding of the anterior portion of abdominal tergite IV and possesses a unique mid-tibial gland (see below). The few *Parasyscia* species that have 11 antennal segments can be distinguished from *Ooceraea* by fused pronotomesopleural Pronotomesopleural suture and larger abdominal segment III.

***Male.*** The males of *Ooceraea* commonly have only 11 antennal segments, which is unique among male dorylines, but a few have 12-segmented antennae, a state shared with most *Acanthostichus* and all *Eusphinctus*, *Simopone*, and *Syscia*. In *Acanthostichus* and *Eusphinctus* the costal vein of fore wing is always present, while missing from the majority of *Ooceraea*. Additionally, in *Acanthostichus* the vein R·f3 is visible beyond pterostigma and in *Eusphinctus* the submarginal cell is closed by Rs·f2–3. Both of these veins are always absent in *Ooceraea*. Distinguishing between males of *Ooceraea* and *Syscia* is difficult. As mentioned above, the majority of species in *Ooceraea* have 11-segmented antennae, while in *Syscia* these seem to be always 12-segmented. In *Ooceraea* the discal cell is closed by cross-vein 1m-cu in the majority of males examined, except for the smallest of specimens, while in the limited material of *Syscia* I have examined this vein appears to be universally absent. Additionally, most *Ooceraea* males have a unique specialization of abdominal sternite VII, ranging from a deep cleft in the middle of the posterior margin and denser pilosity on lateral sides, to conspicuous cuticular projections with a brush of hairs. No *Syscia* have such modifications but in certain *Ooceraea* this character is not obvious (e.g. *Ooceraea biroi*) or absent (a male tentatively associated with *Ooceraea coeca*).

#### Description.

***Worker.*** *Head*: Antennae with 9, 10 (rarely) or 11 segments. Apical antennal segment conspicuously enlarged, much broader than and longer than two preceding segments combined. Clypeus with or without cuticular apron. Lateroclypeal teeth present. Parafrontal ridges reduced. Torulo-posttorular complex vertical. Antennal scrobes absent. Labrum with median notch or concavity. Proximal face of stipes projecting beyond inner margin of sclerite, concealing prementum when mouthparts fully closed. Maxillary palps 3-segmented. Labial palps 2-segmented. Mandibles triangular, with teeth. Eyes absent or present but small, composed of 1–5 ommatidia, very rarely composed of 6–20 ommatidia. Ocelli absent. Head capsule with differentiated vertical posterior surface above occipital foramen. Ventrolateral margins of head without lamella or ridge extending towards mandibles and beyond carina surrounding occipital foramen. Posterior head corners dorsolaterally immarginate. Carina surrounding occipital foramen ventrally absent. *Mesosoma*: Pronotal flange not separated from collar by distinct ridge or not. Promesonotal connection with Pronotomesopleural suture completely fused or Pronotomesopleural suture present, weakly differentiated, immobile. Pronotomesopleural suture visible, unfused up to notal surface. Mesometapleural groove not impressed to weakly impressed. Transverse groove dividing mesopleuron absent. Pleural endophragmal pit concavity present. Mesosoma dorsolaterally immarginate. Metanotal depression or groove on mesosoma absent. Propodeal spiracle situated low on sclerite. Propodeal declivity with or without distinct dorsal edge or margin and rectangular in posterior view. Metapleural gland bulla visible or not through cuticle. Propodeal lobes present, well developed. *Metasoma*: Petiole anterodorsally immarginate, dorsolaterally immarginate, and laterally above spiracle marginate. Helcium in relation to tergosternal Pronotomesopleural suture placed at posttergite and axial. Prora simple, not delimited by carina or a U-shaped margin with median ridge. Spiracle openings of abdominal segments IV–VI circular. Abdominal segment III anterodorsally immarginate and dorsolaterally immarginate. Abdominal segment III about half size of succeeding segment IV, which is strongly constricted at presegmental portion (binodal waist). Girdling constriction of segment IV present, i.e. pre- and postsclerites distinct. Cinctus of abdominal segment IV gutter-like and cross-ribbed. Abdominal segment IV conspicuously largest segment. Abdominal tergite IV not folding over sternite, and anterior portions of sternite and tergite equally well visible in lateral view. Girdling constriction between pre- and posttergites of abdominal segments V and VI absent. Girdling constriction between pre- and poststernites of abdominal segments V and VI absent. Pygidium medium-sized, with impressed medial field, and armed with modified setae. Hypopygium unarmed or armed with modified setae. *Legs*: Mid tibia with single pectinate spur. Hind tibia with single pectinate spur. Hind basitarsus not widening distally, circular in cross-section. Posterior flange of hind coxa not produced as raised lamella. Metatibial gland present as oval patch of whitish cuticle. Metabasitarsal gland absent. Hind pretarsal claws simple. *Polymorphism*: Monomorphic.

***Male.*** *Head*: Antennae with 11 segments or more rarely with 12 segments. Clypeus with cuticular apron. Parafrontal ridges absent. Torulo-posttorular complex vertical. Maxillary palps 5-segmented. Labial palps 3-segmented. Mandibles triangular, edentate. Ventrolateral margins of head without lamella or ridge extending towards mandibles and beyond carina surrounding occipital foramen. Carina surrounding occipital foramen ventrally absent. *Mesosoma*: Pronotal flange not separated from collar by distinct ridge, occasionally ridge marked on sides. Notauli present. Transverse groove dividing mesopleuron present. Propodeal declivity reduced, with or without distinct dorsal edge or margin. Metapleural gland opening absent. Propodeal lobes present. *Metasoma*: Petiole anterodorsally immarginate, dorsolaterally immarginate, and laterally above spiracle marginate, inconspicuously in small species. Helcium in relation to tergosternal Pronotomesopleural suture placed at posttergite and axial. Prora forming a simple U-shaped margin or a U-shaped margin with median ridge. Spiracle openings of abdominal segments IV–VI circular. Abdominal segment III about half size of succeeding segment IV or less; latter strongly constricted at presegmental portion (binodal waist). Girdling constriction of segment IV present, i.e. pre- and postsclerites distinct. Cinctus of abdominal segment IV gutter-like and cross-ribbed. Girdling constriction between pre- and postsclerites of abdominal segments V and VI absent. Abdominal segment IV conspicuously largest segment. Abdominal sternite VII modified, rarely simple. Abdominal sternite IX cleft to modified into two spines, sometimes with additional medial projection or spine, with lateral apodemes about as long as medial apodeme, directed anteriorly (towards head). *Genitalia*: Cupula long relative to rest of genital capsule and shorter ventrally than dorsally. Basimere broadly fused to telomere, with no sulcus trace at junction, and ventrally with left and right arms abutting. Telomere gradually tapering toward apex. Volsella gradually tapering toward apex. Penisvalva laterally compressed, rounded at apex. *Legs*: Mid tibia with single pectinate spur. Hind tibia with single pectinate spur. Posterior flange of hind coxa not produced as raised lamella. Metatibial gland present as oval patch of whitish cuticle. Metabasitarsal glands absent. Hind pretarsal claws simple. *Wings*: Tegula present, broad, demiovate in shape. Vein C in fore wing present or absent. Pterostigma broad. Abscissa R·f3 absent. Abscissae Rs·f2–3 absent. Cross-vein 2r-rs present, forming base of ‘free stigmal vein’ (2r-rs&Rs·f4–5) in absence of Rs·f3 and 2rs-m, although 2rs-m may be present as stub. Abscissae Rs·f4–5 present, fused in absence of 2rs-m or absent. Abscissa M·f2 in fore wing contiguous with Rs+M. Abscissa M·f4 in fore wing absent. Abscissa M·f4 in fore wing present, not reaching wing margin. Cross-vein 1m-cu in fore wing absent or present. Cross-vein cu-a in fore wing present, arising from M+Cu and proximal to M·f1. Vein Cu in fore wing present, with only Cu1 branch prominent. Vein A in fore wing with abscissa A·f1 or with abscissae A·f1 and A·f2 present. Vein C in hind wing absent. Vein R in hind wing absent or present, extending past Sc+R but not reaching distal wing margin. Vein Sc+R in hind wing absent. Vein Sc+R in hind wing present. Abscissa Rs·f1 in hind wing absent. Abscissa Rs·f1 in hind wing present, shorter than 1rs-m. Abscissa Rs·f2 in hind wing absent or present, not reaching wing margin. Cross-vein 1rs-m in hind wing absent. Vein M+Cu in hind wing absent or present. Abscissa M·f1 in hind wing absent. Abscissa M·f2 in hind wing absent. Cross-vein cu-a in hind wing absent or present. Vein Cu in hind wing absent. Vein A in hind wing absent or with abscissa A·f1 present.

***Gyne.*** Ergatoid or replaced by fertile workers (Tsuji and Yamauchi 1995). Mesosomal morphology with wing remnants in one undescribed species suggests that brachypterous or fully winged queens may also occur in this genus. In *Ooceraea crypta* the ergatoid queen possesses multifaceted eyes, three ocelli, no sign of additional sutures on the mesosoma, and an enlarged abdominal segment III (Mann 1921); this morphology could perhaps be called ‘subdichthadiigyne’, although the presence of three well-developed ocelli is atypical. In *Ooceraea besucheti* the only differences between ergatoid gynes and workers include presence of ocelli and enlarged gaster (Brown 1975).

***Larva.*** Larva has been described for *Ooceraea australis* (Wheeler and Wheeler 1964a, 1973). Cocoons absent.

#### Distribution.

*Ooceraea* is a lineage confined to the Indomalayan and Australasian regions, including the Fijian archipelago. *Ooceraea biroi* is a tramp species that has been more widely introduced across tropical regions of the world.

#### Taxonomy and phylogeny.

The taxonomic history of *Ooceraea* is complicated. The genus was originally described by Roger (1862) to include *Ooceraea coeca* from Sri Lanka. Roger did not place *Ooceraea* in a particular group but subsequent authors classified the genus in Myrmicinae (Mayr 1865, Emery 1877), most likely due to the relatively small abdominal segment III (postpetiole) present in these ants. Dalla Torre (1893) considered it a member of the Ponerinae. Later Forel (1893a) established the tribe ‘Cerapachysii’ within Ponerinae, where he included *Ooceraea* along with *Cerapachys* and others. Starting with Emery (1902), *Ooceraea* was treated as a subgenus of *Cerapachys* until Brown’s provisional (1973) and formal (1975) synonymizations of all *Cerapachys* subgenera.

*Cysias* is a name introduced by Emery (1902) for *Ooceraea papuana* and *Ooceraea pusilla* as a subgenus of *Cerapachys*. In Genera Insectorum (Emery 1911) he considered it a synonym of *Syscia*, but explained in his diagnosis of the latter that it encompassed species with two distinct morphologies: ‘Antennae with 9 segments. Without eyes. Basal segment of gaster not much larger than postpetiole (*Syscia*), or much larger and longer than the latter and covering almost all of gaster (*Cysias*)’. This was because of his inclusion of species here placed in *Syscia*, *Syscia typhla*, which also has 9-segmented antennae but a relatively large abdominal segment III (postpetiole). Based on morphology, *papuana* and *pusilla* are here considered species of *Ooceraea*. See also the discussion of Emery’s Genera Insectorum classifications in the section on doryline taxonomy above.

Genomic data show that *Ooceraea* is most closely related to *Eusphinctus* and *Syscia* (Borowiec, in prep.). No attempts to investigate the internal phylogeny have been made.

#### Biology.

The members of this lineage are found primarily in leaf litter and soil core samples. Worker morphology (eyes often very small or absent) is also suggestive of subterranean habits.

*Ooceraea biroi* is perhaps the best studied doryline species. The army ants *Eciton* and *Dorylus* have been extensively researched in the field, but their huge colonies are exceptionally difficult to manipulate in laboratory conditions. In contrast, *Ooceraea biroi* is a species much more amenable to experimental manipulation and has been the focal organism for multiple published laboratory-based studies.

*Ooceraea biroi* is a clonal species where all workers in a colony have reproductive potential and multiple individuals are active egg layers (Tsuji and Yamauchi 1995). Brood is synchronized and alternating cycles of reproductive and foraging phases occur, much like in *Eciton* (Ravary and Jaisson 2002, 2004, Ravary et al. 2006). Much like most other dorylines, *Ooceraea biroi* is a specialist predator on other ants’ brood, although it can attack other soft-bodied insects (Wetterer et al. 2012). The workers are blind and, like many dorylines, rely solely on chemical communication. A recent study found that *Ooceraea biroi* has the largest number of odorant receptor genes of any insect sequenced (Oxley et al. 2014). The colonies number between a hundred and several hundred individuals. *Ooceraea biroi* is also a ‘tramp species’ whose native range is likely limited to mainland southeast Asia (Kronauer et al. 2012), but it has been established in numerous tropical islands throughout the world, including Japan, Hawaii, Madagascar and Seychelles, and the West Indies (Wetterer et al. 2012). It is the only member of the subfamily whose genome has been published (Oxley et al. 2014).

In addition to offering a rare opportunity for studying the habits of a non-army ant doryline, *Ooceraea biroi* has also provided some important insights into social insect biology in general. A study by Ravary et al. (2007) showed that division of labor is influenced by learning in this species. Individuals that experienced high success rates in foraging would specialize in this task, whereas ants failing at prey discovery would decrease their foraging activity and spend more time on brood care. Teseo et al. (2013) demonstrated that *Ooceraea biroi* workers will execute their genetically identical sisters if they fail to conform to the reproductive activity cycles necessary for synchronized brood development. This behavior in the absence of genetic conflict highlights the importance of worker policing for the economics of a social insect colony (Oldroyd 2013).

It is unknown whether the clonal reproduction and brood production synchronicity in *Ooceraea biroi* is representative of other *Ooceraea* and if the species is a part of an older clade of parthenogenetic lineages or an exception. Subdichthadiigyne queens of *Ooceraea crypta* suggest more traditional reproduction in at least one other species. Many males of *Ooceraea* have a highly modified abdominal sternite VII, suggesting its involvement in copulation (see discussion of male characters above). An Australian species *Ooceraea australis* is relatively common throughout the continent and has been reported to form colonies with thousands of individuals (Heterick 2009).

#### Species of *Ooceraea*

*Ooceraea alii* (Bharti and Akbar, 2013): India, **comb. n.**

*Ooceraea australis* (Forel, 1900a): Australia, **nom. rev.**

*Ooceraea biroi* (Forel, 1907a): Singapore, **comb. n.**

*Ooceraea besucheti* (Brown, 1975): India, **comb. n.**

*Ooceraea coeca* Mayr, 1897: Sri Lanka, **comb. rev.**

*Ooceraea crypta* (Mann, 1921): Fiji, **comb. n.**

*Ooceraea fuscior* (Mann, 1921): Fiji, **comb. n.**

*Ooceraea fragosa* Roger, 1862: Sri Lanka, **comb. rev.**

*Ooceraea papuana* Emery, 1897: Papua New Guinea, **comb. rev.**

*Ooceraea pawa* (Mann, 1919): Solomon Islands, **comb. n.**

*Ooceraea pusilla* Emery, 1897: Papua New Guinea, **comb. n.**

### Parasyscia

Taxon classificationAnimaliaHymenopteraFormicidae

Emery, 1882 gen. rev.

#### Type-species.

*Parasyscia piochardi*, by monotypy.

After *Lioponera* this is the most species-rich lineage formerly included in *Cerapachys*.

#### Diagnosis.

***Worker.*** *Parasyscia* workers are distinguished by a combination of propodeal spiracle positioned low on the sclerite and propodeal lobes present, constriction between abdominal segments III and IV, petiole dorsolaterally not marginate, no constriction between abdominal segments IV, V, and VI, pronotomesopleural Pronotomesopleural suture fused, helcium axial, middle tibiae with a single pectinate spur, pretarsal claws unarmed, and abdominal segment III anterodorsally often marginate. *Parasyscia* is an exclusively Old World group and is most similar to *Neocerapachys* of the New World, which can be differentiated by narrower trench leading to the metapleural gland orifice, the presence of two patches of denser pilosity on abdominal tergite IV, and different palp formula (3,3 in *Neocerapachys* versus 3,2 or 2,2 in *Parasyscia*).

***Male.*** The males of *Parasyscia* have variably developed wing venation and are generally diverse, making them somewhat challenging to identify. Most species share characteristic venation: C and R·f3 are absent, Rs·f2–3 is present and runs all the way from Rs+M to 2r-rs, closing a submarginal cell. In addition, *Parasyscia* males have 13-segmented antennae, antennal toruli fully exposed, no constrictions between abdominal segments IV, V, and VI, narrow and axial helcium, and a single spur on each middle and hind tibia. *Lividopone* and *Zasphinctus* may have similar wing venation but the former has a broad supraaxial helcium and the latter has pronounced constrictions between abdominal segments IV, V, and VI. Along with *Lioponera*, *Parasyscia* males are among the most common of non-army ant doryline males in collections from the Old World. Except for specimens with much reduced wing venation, *Lioponera* can be distinguished by a ‘free stigmal vein’ formed in complete absence of Rs·f2–3.

#### Description.

***Worker.*** *Head*: Antennae with 11 or 12 segments. Apical antennal segment conspicuously enlarged, much broader than and longer than two preceding segments combined. Clypeus with cuticular apron. Lateroclypeal teeth absent. Parafrontal ridges reduced. Torulo-posttorular complex vertical. Antennal scrobes absent. Labrum with median notch or concavity. Proximal face of stipes projecting beyond inner margin of sclerite, concealing prementum when mouthparts fully closed. Maxillary palps 3- or 2-segmented. Labial palps 2-segmented. Mandibles triangular, with teeth. Mandibles triangular, edentate. Eyes present, composed of 1 to more than 20 ommatidia. Ocelli absent. Head capsule with differentiated vertical posterior surface above occipital foramen. Ventrolateral margins of head with or without lamella or ridge extending towards mandibles and beyond carina surrounding occipital foramen. Posterior head corners dorsolaterally immarginate. Carina surrounding occipital foramen ventrally present. *Mesosoma*: Pronotal flange separated or not from collar by distinct ridge. Promesonotal connection with Pronotomesopleural suture completely fused. Pronotomesopleural suture completely fused. Mesometapleural groove weakly impressed. Transverse groove dividing mesopleuron absent or present. Pleural endophragmal pit concavity present. Mesosoma dorsolaterally immarginate. Metanotal depression or groove on mesosoma absent. Propodeal spiracle situated low on sclerite. Propodeal declivity with or without distinct dorsal edge or margin and rectangular in posterior view. Metapleural gland without bulla visible through cuticle. Propodeal lobes present, well developed. *Metasoma*: Petiole anterodorsally immarginate or marginate, dorsolaterally immarginate, and laterally above spiracle marginate. Helcium in relation to tergosternal Pronotomesopleural suture placed at Pronotomesopleural suture and axial. Prora forming a U-shaped margin with median ridge. Spiracle openings of abdominal segments IV–VI circular. Abdominal segment III anterodorsally immarginate and dorsolaterally immarginate. Abdominal segment III more than half size of succeeding segment IV, which is weakly constricted at presegmental portion (uninodal waist). Girdling constriction of segment IV present, i.e. pre- and postsclerites distinct. Cinctus of abdominal segment IV gutter-like and cross-ribbed. Abdominal segment IV not conspicuously largest segment. Abdominal tergite IV not folding over sternite, and anterior portions of sternite and tergite equally well visible in lateral view. Girdling constriction between pre- and posttergites of abdominal segments V and VI absent. Girdling constriction between pre- and poststernites of abdominal segments V and VI absent. Pygidium large, with impressed medial field and armed with modified setae. Hypopygium unarmed. *Legs*: Mid tibia with single pectinate spur. Hind tibia with single pectinate spur. Hind basitarsus not widening distally, circular in cross-section. Posterior flange of hind coxa not produced as raised lamella. Metatibial gland present as oval patch of whitish cuticle. Metabasitarsal gland absent. Hind pretarsal claws simple. *Polymorphism*: Monomorphic.

***Male.*** *Head*: Antennae with 13 segments. Clypeus with cuticular apron. Parafrontal ridges present. Torulo-posttorular complex vertical. Maxillary palps 2-segmented. Labial palps 2-segmented. Mandibles triangular, edentate. Ventrolateral margins of head without lamella or ridge extending towards mandibles and beyond carina surrounding occipital foramen. Carina surrounding occipital foramen ventrally absent. *Mesosoma*: Pronotal flange separated from collar by distinct ridge mostly on sides or not separated. Notauli absent or present. Transverse groove dividing mesopleuron present. Propodeal declivity reduced, with or without distinct dorsal edge or margin. Metapleural gland opening absent. Propodeal lobes present. *Metasoma*: Petiole anterodorsally immarginate or marginate, dorsolaterally immarginate, and laterally above spiracle marginate. Helcium in relation to tergosternal Pronotomesopleural suture placed at posttergite and axial. Prora forming a U-shaped margin with median ridge. Spiracle openings of abdominal segments IV–VI circular. Abdominal segment III more than half size of succeeding segment IV; latter weakly constricted at presegmental portion (uninodal waist). Girdling constriction of segment IV present, i.e. pre- and postsclerites distinct. Cinctus of abdominal segment IV gutter-like and cross-ribbed. Girdling constriction between pre- and postsclerites of abdominal segments V and VI absent. Abdominal segment IV conspicuously largest segment. Abdominal sternite VII simple. Abdominal sternite IX distally armed with two spines, with lateral apodemes about as long as medial apodeme, directed anteriorly (towards head). *Genitalia*: Cupula long relative to rest of genital capsule and shorter ventrally than dorsally. Basimere broadly fused to telomere, with no sulcus trace at junction, and ventrally with left and right arms separated. Telomere gradually tapering toward apex. Volsella gradually tapering toward apex. Penisvalva laterally flattened, at apex hooked ventrally. *Legs*: Mid tibia with single pectinate spur. Hind tibia with single pectinate spur. Posterior flange of hind coxa not produced as raised lamella. Metatibial gland absent. Metabasitarsal glands absent. Hind pretarsal claws simple. *Wings*: Tegula present, broad, demiovate in shape. Vein C in fore wing absent. Pterostigma broad. Abscissa R·f3 absent. Abscissae Rs·f2–3 present, disconnected from Rs+M or connecting with Rs+M&M·f2. Cross-vein 2r-rs present, differentiated from Rs·f4 by presence of Rs·f2–3. Abscissae Rs·f4–5 present, fused in absence of 2rs-m. Abscissa M·f2 in fore wing present, separated from Rs+M by Rs·f2 or contiguous with Rs+M. Abscissa M·f4 in fore wing present, not reaching wing margin. Cross-vein 1m-cu in fore wing absent or present. Cross-vein cu-a in fore wing present, arising from M+Cu at variable distance, proximal, distal to, at or near M·f1. Vein Cu in fore wing present, with only Cu1 branch prominent. Vein A in fore wing with abscissa A·f1 present or with abscissae A·f1 and A·f2 present; A·f2 short. Vein C in hind wing absent. Vein R in hind wing absent. Vein Sc+R in hind wing absent. Abscissa Rs·f1 in hind wing absent. Abscissa Rs·f2 in hind wing present, not reaching wing margin. Cross-vein 1rs-m in hind wing present, about as long as M·f1. Vein M+Cu in hind wing present. Abscissa M·f1 in hind wing present. Abscissa M·f2 in hind wing absent or present. Cross-vein cu-a in hind wing present. Vein Cu in hind wing present. Vein A in hind wing with abscissa A·f1 or with abscissae A·f1 and A·f2 present.

***Gyne.*** Alate gynes are known in a number of species, e.g. *Parasyscia afer*, *Parasyscia imerinensis*, *Parasyscia reticulata*. Ergatoid gynes are also known, for example in *Parasyscia indica*, *Parasyscia nayana*, *Parasyscia schoedli*, *Parasyscia seema*, and *Parasyscia sudanensis*. The ergatoid gyne of *Parasyscia schoedli* is extremely worker-like, does not possess ocelli, and differs form the worker mostly in relatively larger gaster and more erect pilosity. In *Parasyscia seema* intercastes or ergatoids with ocelli but no modifications to the mesosoma are known in addition to a gyne with well-developed mesosomal sutures. It is unclear, however, whether the latter is dealated or never possessed wings (Bharti and Akbar 2013).

***Larva.*** The larva of *Parasyscia opaca* has been described (Wheeler and Wheeler 1964a). Cocoon presence unknown.

#### Distribution.

50 species of *Parasyscia* are known, distributed throughout the warm temperate and tropical Old World, extending into New Guinea and many Pacific islands but absent from Australia.

#### Taxonomy and phylogeny.

*Parasyscia* was described as a genus by Emery in 1882 and shortly afterwards treated as a subgenus by Forel 1892. Most authors adopted Forel’s decision and finally Kempf synonymized it under *Cerapachys* in his catalog of Neotropical ants (Kempf 1972). Relatively few species have been described under this name, but in the sense proposed here it is equivalent to Brown’s ‘*dohertyi*-*cribrinodis* group’ (Brown 1975), keyed with other *Cerapachys* in the same publication (Brown 1975).

*Parasyscia* has been identified as the sister group of *Zasphinctus* (Brady et al. 2014), but there have been no attempts to reconstruct the internal phylogeny. Material examined in collections suggests that many species remain to be described (author’s unpublished observations).

#### Biology.

Two *Parasyscia* species, *Parasyscia flavaclavata* and *Parasyscia opaca*, were observed in the field in New Guinea (Wilson 1958). *Parasyscia flavaclavata* was seen raiding a colony of a *Pheidole* species. A nest of *Parasyscia opaca* was collected from a rotting log, containing <100 workers, a single queen, and brood and adults of the apparent prey, *Strumigenys loriae*. The brood of *Parasyscia opaca* consisted of larvae of the same size, suggesting synchronized brood production. Other species have been collected from rotten logs and under stones (Brown 1975), and at least one, *Parasyscia zimmermanni*, is an arboreal nester (Sarnat and Economo 2012). *Parasyscia imerinensis* was observed in an urban habitat of the botanical garden and zoo in Antananarivo, Madagascar. Two workers were seen slowly walking on pavement stones shortly after dark. It is difficult to assess whether these individuals represented scouts or if this species forages solitarily (author’s observations).

#### Species of *Parasyscia*

*Parasyscia afer* (Forel, 1907a): Tanzania, **comb. n.**

*Parasyscia aitkenii* (Forel, 1900b): India, **comb. n.**

*Parasyscia arnoldi* (Forel, 1914): South Africa, **comb. n.**

*Parasyscia browni* (Bharti and Wachkoo, 2013): India, **comb. n.**

*Parasyscia bryanti* (Wheeler, W. M., 1919): Malaysia (Sarawak), **comb. n.**

*Parasyscia centurio* (Brown, 1975): Democratic Republic of the Congo, **comb. n.**

*Parasyscia conservata* (Viehmeyer, 1913): Indonesia (Sulawesi, in copal), **comb. n.**

*Parasyscia cribrinodis* Emery, 1899b: Cameroon, **comb. rev.**

*Parasyscia desposyne* (Wilson, 1959): Papua New Guinea, **comb. n.**

*Parasyscia dohertyi* (Emery, 1902): Indonesia (Laut Island), **comb. n.**

*Parasyscia dominula* (Wilson, 1959): Indonesia (Papua), **comb. n.**

*Parasyscia faurei* (Arnold, 1949): South Africa, **comb. n.**

*Parasyscia flavaclavata* (Donisthorpe, 1938): Indonesia (Papua), **comb. n.**

*Parasyscia fossulata* (Forel, 1895a): Sri Lanka, **comb. n.**

*Parasyscia foveolata* (Radchenko, 1993): Vietnam, **comb. n.**

*Parasyscia hashimotoi* (Terayama, 1996): Japan, **comb. n.**

*Parasyscia imerinensis* Forel, 1891: Madagascar, **comb. rev.**

*Parasyscia inconspicua* Emery, 1901c: Papua New Guinea, **comb. n.**

*Parasyscia indica* (Brown, 1975): India, **comb. n.**

*Parasyscia kenyensis* (Consani, 1951): Kenya, **comb. n.**

*Parasyscia keralensis* (Karmaly, 2012): India, **comb. n.**

*Parasyscia kodecorum* (Brown, 1975): Indonesia (South Kalimantan), **comb. n.**

*Parasyscia lamborni* (Crawley, 1923): Malawi, **comb. n.**

*Parasyscia lindrothi* (Wilson, 1959): Fiji, **comb. n.**

*Parasyscia luteoviger* (Brown, 1975): Sri Lanka, **comb. n.**

*Parasyscia majuscula* (Mann, 1921): Fiji, **comb. n.**

*Parasyscia muiri* (Wheeler, W. M. and Chapman, 1925): Philippines, **comb. n.**

*Parasyscia natalensis* (Forel, 1901d): South Africa, **comb. n.**

*Parasyscia nitens* (Donisthorpe, 1949): Indonesia (Papua), **comb. n.**

*Parasyscia nitidula* (Brown, 1975): Democratic Republic of the Congo, **comb. n.**

*Parasyscia opaca* (Emery, 1901c): Papua New Guinea, **comb. n.**

*Parasyscia peringueyi* Emery, 1886: South Africa, **comb. rev.**

*Parasyscia piochardi* Emery, 1882: Syrian Arab Republic, **comb. rev.**

*Parasyscia polynikes* (Wilson, 1959): Papua New Guinea, **comb. n.**

*Parasyscia reticulata* (Emery, 1923): Taiwan, **comb. n.**

*Parasyscia rufithorax* (Wheeler, W. M. and Chapman, 1925): Philippines, **comb. n.**

*Parasyscia salimani* (Karavaiev, 1925): Indonesia (Java), **comb. n.**

*Parasyscia seema* (Bharti and Akbar, 2013): India, **comb. n.**

*Parasyscia schoedli* (Bharti and Akbar, 2013): India, **comb. n.**

*Parasyscia sculpturata* (Mann, 1921): Fiji, **comb. n.**

*Parasyscia sudanensis* (Weber, 1942): South Sudan, **comb. n.**

*Parasyscia sylvicola* (Arnold, 1955): Zimbabwe, **comb. n.**

*Parasyscia superata* (Wilson, 1959): Papua New Guinea, **comb. n.**

*Parasyscia terricola* (Mann, 1919): Solomon Islands, **comb. n.**

*Parasyscia valida* (Arnold, 1960): South Africa, **comb. n.**

*Parasyscia villiersi* (Bernard, 1953b): Guinea, **comb. n.**

*Parasyscia vitiensis* (Mann, 1921): Fiji, **comb. n.**

*Parasyscia wighti* (Bharti and Akbar, 2013): India, **comb. n.**

*Parasyscia wittmeri* (Collingwood, 1985): Saudi Arabia, **comb. n.**

*Parasyscia zimmermani* (Wilson, 1959): Fiji, **comb. n.**

### Procerapachys

Taxon classificationAnimaliaHymenopteraFormicidae

Wheeler, W. M. 1915b

#### Type-species.

*Procerapachys annosus*, by original designation.

*Procerapachys* is an extinct genus known from Baltic amber.

#### Diagnosis.

***Worker.*** The extinct *Procerapachys* is apparently unique among non-army ant dorylines in having a large but unarmed pygidium. All other dorylines with unarmed pygidium have either highly positioned propodeal spiracles and no propodeal lobes (*Aenictus*, *Aenictogiton*, *Dorylus*) or a reduced pygidium (*Leptanilloides*). When pygidium is not clearly visible, these often heavily-sculptured ants can be confused with *Chrysapace*, which also occurs in Eocene ambers (see under that taxon above). *Chrysapace* and *Procerapachys* differ in spur formula, however. The former has two pectinate spurs on each mid and hind tibia, and the latter has only one pectinate spur. *Procerapachys* specimens were also reported to have palp formula 5,4, which is different from 5,3 counted in one of the extant *Chrysapace*.

***Male.*** The status of the putative males of *Procerapachys* is uncertain, but the specimens originally attributed to this genus had well-developed wing venation with two submarginal cells and the marginal cell closed, similar to *Chrysapace* and *Cylindromyrmex*. The most reliable character that separates these males from these two genera is a single pectinate tibial spur in *Procerapachys* and two spurs present in both *Chrysapace* and *Cylindromyrmex*.

#### Description.

***Worker.*** *Head*: Antennae with 12 segments. Apical antennal segment not enlarged, not broader and longer than two preceding segments combined. Clypeal apron unknown. Lateroclypeal teeth unknown. Parafrontal ridges reduced. Torulo-posttorular complex vertical. Antennal scrobes absent. Labrum shape unknown. Proximal face of stipes unknown. Maxillary palps 5-segmented. Labial palps 4-segmented. Mandibles triangular, edentate. Eyes present, composed of more than 20 ommatidia. Ocelli absent or present. Head capsule with differentiated vertical posterior surface above occipital foramen. Ventrolateral margins of head unknown. Posterior head corners dorsolaterally immarginate. Carina surrounding occipital foramen unknown. *Mesosoma*: Pronotal flange separated from collar by distinct ridge. Promesonotal connection with Pronotomesopleural suture conspicuous and complete, but immobile. Pronotomesopleural suture complete, continuous with promesonotal Pronotomesopleural suture. Mesometapleural groove not impressed. Transverse groove dividing mesopleuron absent. Pleural endophragmal pit concavity unknown. Mesosoma dorsolaterally immarginate. Metanotal depression or groove on mesosoma absent. Propodeal spiracle situated low on sclerite. Propodeal declivity with distinct dorsal edge or margin and rectangular in posterior view. Metapleural gland unknown. Propodeal lobes present, well developed. *Metasoma*: Petiole anterodorsally unknown, dorsolaterally immarginate, and laterally above spiracle marginate. Placement of helcium unknown. Prora unknown. Spiracle openings of abdominal segments IV–VI unknown. Abdominal segment III anterodorsally unknown and dorsolaterally unknown. Abdominal segment III more than half size of succeeding segment IV, which is weakly constricted at presegmental portion (uninodal waist). Girdling constriction of segment IV present, i.e. pre- and postsclerites distinct. Cinctus of abdominal segment IV unknown. Abdominal segment IV not conspicuously largest segment. Abdominal tergite IV not folding over sternite, and anterior portions of sternite and tergite equally well visible in lateral view. Girdling constriction between pre- and posttergites of abdominal segments V and VI absent. Girdling constriction between pre- and poststernites of abdominal segments V and VI absent. Pygidium large, with impressed medial field and simple, not armed with cuticular spines or modified setae. Hypopygium unknown. *Legs*: Mid tibia with single pectinate spur. Hind tibia with single pectinate spur. Hind basitarsus not widening distally, circular in cross-section. Posterior flange of hind coxa unknown. Metatibial gland unknown. Metabasitarsal gland unknown. Hind pretarsal claws unknown. Hind pretarsal claws simple. *Polymorphism*: Unknown.

***Male.*** (putative, see under Taxonomy and phylogeny below) *Head*: Antennae with 13 segments. Clypeal lamella unknown. Parafrontal ridges unknown. Torulo-posttorular complex vertical. Maxillary palps unknown. Labial palps unknown. Mandibles triangular, edentate. Ventrolateral margins of head unknown. Carina surrounding occipital foramen unknown. *Mesosoma*: Pronotal flange separated from collar by distinct ridge. Notauli unknown. Transverse groove dividing mesopleuron absent. Propodeal declivity with distinct dorsal edge or margin. Metapleural gland opening unknown. Propodeal lobes present. *Metasoma*: Petiole anterodorsally immarginate, dorsolaterally immarginate, and laterally above spiracle unknown. Helcium in relation to tergosternal Pronotomesopleural suture placed at posttergite and axial. Prora unknown. Spiracle openings of abdominal segments IV–VI circular. Abdominal segment III more than half size of succeeding segment IV; latter weakly constricted at presegmental portion (uninodal waist). Girdling constriction of segment IV present, i.e. pre- and postsclerites distinct. Cinctus of abdominal segment IV unknown. Girdling constriction between pre- and postsclerites of abdominal segments V and VI absent. Abdominal segment IV not conspicuously largest segment. Abdominal sternite VII simple. Abdominal sternite IX unknown. *Genitalia*: Genital morphology unknown. *Legs*: Mid tibia with single pectinate spur. Hind tibia with single pectinate spur. Posterior flange of hind coxa unknown. Metatibial gland unknown. Metabasitarsal glands unknown. Hind pretarsal claws unknown. *Wings*: Tegula unknown. Vein C in fore wing present. Pterostigma broad. Abscissa R·f3 present, running toward distal wing margin and enclosing cell with Rs·f5. Abscissae Rs·f2–3 present, connecting with Rs+M&M·f2. Cross-vein 2r-rs present, differentiated from Rs·f4 by presence of Rs·f2–3. Abscissae Rs·f4–5 differentiated into Rs·f4 and Rs·f5 by 2rs-m. Abscissa M·f2 in fore wing present, separated from Rs+M by Rs·f2. Abscissa M·f4 in fore wing present, reaching wing margin. Cross-vein 1m-cu in fore wing present. Cross-vein cu-a in fore wing present, arising from Cu and distal to, at or near M·f1. Vein Cu in fore wing present, with both branches Cu1 and Cu2. Hind wing venation unknown.

***Gyne.*** Not described. Wheeler (1915) mentioned that some of the specimens possessed ocelli while others did not and suggesting that these may represent ergatogynes.

***Larva.*** Not described. Presence of cocoons unknown.

#### Taxonomy and phylogeny.

*Procerapachys* was described based on several workers and two male specimens by W. M. Wheeler (1915) in his monograph on the Baltic amber collection of the Geological Institute of Königsberg (now Kaliningrad, Russia). Unfortunately, most of this collection was destroyed during WWII, including the *Procerapachys* material (Dlussky, 2009). Dlussky (2009) redescribed the genus based on additional specimens of what he identified as the type species, *Procerapachys annosus*, designated a neotype for it, and added a new species, *Procerapachys sulcatus*. Both worker and putative male morphologies of *Procerapachys annosus* and *Procerapachys sulcatus* are reminiscent of the extant genus *Chrysapace*. If the published descriptions are accurate, however, there are important differences that include a single pectinate spur on each mid and hind tibiae (mentioned by both Wheeler and Dlussky), different palp formula, and, perhaps most importantly, a pygidium not impressed and without modified spine-like setae in the worker. According to the descriptions it also appears that at least some specimens of *Procerapachys* lack ocelli, while ocelli are present in all *Chrysapace* material I examined in the course of this study. In addition, one of the species, *Procerapachys favosus*, lacks the coarse sulcate sculpturing characteristic of *Chrysapace*. In fact, there are amber doryline specimens without coarse sculpturing that fit the original *Procerapachys* by having a single tibial spur and a smooth pygidium, for which I was able to examine high-quality photographs and consult these characters with Vincent Perrichot, who was able to confirm them directly on the specimens. Unfortunately, I was not able to examine any of the specimens on which Dlussky based his descriptions. I have examined a specimen identified as *Procerapachys annosus* from the collection of Senckenberg Forschungsinstitut und Naturmuseum Frankfurt and found it to be a typical *Chrysapace* with two conspicuous tibial spurs. I have also examined photographs of a specimen (Vincent Perrichot pers. comm.) from a private collection that fits the original description of *Procerapachys annosus* and its habitus appears to be distinct from *Chrysapace*, although I could not assess the shape of the pygidium or tibial spur configuration. Thus at least some of the species attributed in the past to *Procerapachys* indeed represent a distinct doryline lineage. In the absence of strong evidence to the contrary, I treat *Procerapachys* as distinct from *Chrysapace* or any other genus recognized here. However, a careful reevaluation of the amber fossil dorylines, most crucially the neotype of *Procerapachys annosus*, as well as the putative males, is much needed.

#### Distribution.

Eocene Baltic and Bitterfeld ambers.

#### Species of *Procerapachys*

†*Procerapachys annosus* Wheeler, W. M. 1915b: Baltic amber

†*Procerapachys favosus* Wheeler, W. M. 1915b: Baltic amber

†*Procerapachys sulcatus* Dlussky, 2009: Baltic amber

### Simopone

Taxon classificationAnimaliaHymenopteraFormicidae

Forel, 1891

#### Type-species.

*Simopone grandidieri*, by monotypy.

*Simopone* is a genus of arboreal predators of other ants, found in the Old World tropics.

#### Diagnosis.

***Worker.*** Workers of *Simopone* are unique among all dorylines in the combination of 11-segmented antennae, eyes and ocelli present, no spur on mid tibia, and the lack of metatibial gland. *Simopone* also possess a conspicuous groove on the interior surface of hind basitarsus. Other dorylines lacking mid tibial spur include certain species of *Lioponera*, all *Tanipone*, and *Vicinopone*. All these genera have 12-segmented antennae.

***Male.*** The males are easily identified by a combination of antennal sockets partially concealed by the torulo-posttorular complex in full-face view, 12-segmented antennae, presence of notauli, and lack of spurs on middle tibiae. The only other non-army ant doryline genus that lacks spurs on middle tibiae is *Tanipone*, although it is possible that *Vicinopone* males will turn out to lack them also, when discovered. All *Tanipone* males known thus far have fully exposed antennal sockets, 13-segmented antennae, and no notauli, in addition to characteristically long maxillary palps that reach the occipital foramen.

#### Description.

***Worker.*** *Head*: Antennae with 11 segments. Apical antennal segment not enlarged, not broader and longer than two preceding segments combined. Clypeus without cuticular apron. Lateroclypeal teeth absent. Parafrontal ridges absent or reduced. Torulo-posttorular complex horizontal. Antennal scrobes absent or present. Labrum with median notch or concavity. Proximal face of stipes projecting beyond inner margin of sclerite, concealing prementum when mouthparts fully closed. Maxillary palps 6- or 5-segmented. Labial palps 4- or 3-segmented. Mandibles triangular, edentate. Eyes present, composed of more than 20 ommatidia. Ocelli present. Head capsule without differentiated vertical posterior surface above occipital foramen. Ventrolateral margins of head without lamella or ridge extending towards mandibles and beyond carina surrounding occipital foramen. Posterior head corners dorsolaterally immarginate. Carina surrounding occipital foramen ventrally absent. *Mesosoma*: Pronotal flange often separated from collar by distinct ridge, occasionally ridge absent. Promesonotal connection with Pronotomesopleural suture present, weakly differentiated, immobile. Pronotomesopleural suture visible, unfused up to notal surface. Mesometapleural groove weakly impressed or not impressed. Transverse groove dividing mesopleuron absent or present. Pleural endophragmal pit concavity present. Mesosoma dorsolaterally immarginate. Metanotal groove on mesosoma absent or shallowly impressed but well-defined line. Propodeal spiracle situated low on sclerite. Propodeal declivity often without distinct dorsal edge or margin but occasionally marginate, rectangular in posterior view. Metapleural gland without bulla visible through cuticle. Metapleural gland with bulla visible through cuticle. Propodeal lobes present, well developed. *Metasoma*: Petiole anterodorsally immarginate or marginate, dorsolaterally immarginate, and laterally above spiracle marginate. Helcium in relation to tergosternal Pronotomesopleural suture placed at posttergite and axial. Prora simple, not delimited by carina, a U-shaped margin, or U-shaped margin with median ridge. Spiracle openings of abdominal segments IV–VI circular. Abdominal segment III anterodorsally immarginate and dorsolaterally immarginate. Abdominal segment III more than half size of succeeding segment IV, which is weakly constricted at presegmental portion (uninodal waist). Girdling constriction of segment IV present, i.e. pre- and postsclerites distinct. Cinctus of abdominal segment IV gutter-like, not sculptured. Abdominal segment IV not conspicuously largest segment. Abdominal tergite IV not folding over sternite, and anterior portions of sternite and tergite equally well visible in lateral view. Girdling constriction between pre- and posttergites of abdominal segments V and VI absent. Girdling constriction between pre- and poststernites of abdominal segments V and VI absent. Pygidium large, with impressed medial field, armed with modified setae, and in some species deeply notched at apex. Hypopygium unarmed. *Legs*: Mid tibia without spurs. Hind tibia with single pectinate spur. Hind basitarsus not widening distally, circular in cross-section. Posterior flange of hind coxa not produced as raised lamella. Metatibial gland absent. Metabasitarsal gland present. Hind pretarsal claws each armed with a tooth. *Polymorphism*: Monomorphic.

***Male.*** *Head*: Antennae with 12 segments. Clypeus without cuticular apron. Parafrontal ridges present. Torulo-posttorular complex horizontal. Maxillary palps 6- or 5-segmented. Labial palps 4- or 3-segmented. Mandibles triangular, edentate. Ventrolateral margins of head without lamella or ridge extending towards mandibles and beyond carina surrounding occipital foramen. Carina surrounding occipital foramen ventrally absent. *Mesosoma*: Pronotal flange separated from collar by distinct ridge. Notauli present. Transverse groove dividing mesopleuron absent. Propodeal declivity with distinct dorsal edge or margin. Metapleural gland opening absent. Propodeal lobes present. *Metasoma*: Petiole anterodorsally marginate, dorsolaterally immarginate, and laterally above spiracle marginate. Helcium in relation to tergosternal Pronotomesopleural suture placed at posttergite and axial. Prora forming a U-shaped protrusion. Spiracle openings of abdominal segments IV–VI circular. Abdominal segment III more than half size of succeeding segment IV; latter weakly constricted at presegmental portion (uninodal waist). Girdling constriction of segment IV present, i.e. pre- and postsclerites distinct. Cinctus of abdominal segment IV gutter-like and cross-ribbed. Girdling constriction between pre- and postsclerites of abdominal segments V and VI absent. Abdominal segment IV not conspicuously largest segment. Abdominal sternite VII simple. Abdominal sternite IX distally armed with two spines, with lateral apodemes about as long as medial apodeme, directed anteriorly (towards head). *Genitalia*: Cupula long relative to rest of genital capsule and shorter ventrally than dorsally. Basimere broadly fused to telomere, with no sulcus trace at junction, and ventrally with left and right arms separated. Telomere expanded at apex. Volsella variable. Penisvalva laterally compressed, rounded at apex. *Legs*: Mid tibia without spurs. Hind tibia with single pectinate spur. Posterior flange of hind coxa not produced as raised lamella. Metatibial gland absent. Metabasitarsal glands absent. Hind pretarsal claws each armed with a tooth. *Wings*: Tegula present, broad, demiovate in shape. Vein C in fore wing absent. Pterostigma broad. Abscissa R·f3 absent. Abscissae Rs·f2–3 present, connecting with Rs+M&M·f2. Cross-vein 2r-rs present, differentiated from Rs·f4 by presence of Rs·f2–3 or absent. Abscissae Rs·f4–5 present, fused in absence of 2rs-m. Abscissa M·f2 in fore wing present, separated from Rs+M by Rs·f2. Abscissa M·f4 in fore wing present, reaching wing margin. Cross-vein 1m-cu in fore wing present or absent. Cross-vein cu-a in fore wing present, arising from M+Cu and proximal to M·f1. Vein Cu in fore wing present, with both branches Cu1 and Cu2. Vein A in fore wing with abscissae A·f1 and A·f2 present. Vein C in hind wing absent. Vein R in hind wing absent. Vein Sc+R in hind wing present. Abscissa Rs·f1 in hind wing unknown. Abscissa Rs·f2 in hind wing unknown. Cross-vein 1rs-m in hind wing present, about as long as M·f1, never tubular. Vein M+Cu in hind wing present. Abscissa M·f1 in hind wing present. Abscissa M·f2 in hind wing present. Cross-vein cu-a in hind wing present. Vein Cu in hind wing present. Vein A in hind wing with abscissae A·f1 and A·f2 present.

***Gyne.*** Alate or extremely ergatoid/ replaced by fertile workers. Alate and dealated queen specimens are known in all three *Simopone* species-groups recognized by Bolton and Fisher (2012), e.g. in *Simopone annettae* of the *schoutedeni* group, *Simopone latiscapa* of the *emeryi* group, and *Simopone bakeri* of the *grandidieri* group. Members of all three species-groups occur also on Madagascar. However, no morphologically distinct gynes have ever been collected among the 16 species occurring on the island, despite multiple nest samples available. It is possible that queens have been replaced there by reproductively active workers, the so-called gamergates (Bolton and Fisher 2012).

***Larva.*** Not described. Cocoons absent.

#### Distribution.

*Simopone* is limited to the Old World and currently contains 39 named species. Most occur in Madagascar and in the Afrotropics but five rare species have been recorded from the Indomalayan Region (China, Thailand, Vietnam, Singapore, Philippines) and New Guinea.

#### Taxonomy and phylogeny.

Bolton and Fisher (2012) revised and keyed all species in the Afrotropical and Malagasy regions. They also classified *Simopone* species into three groups based on morphology, but it is unknown whether these are monophyletic. The phylogenetic position of *Simopone* is not well understood (Brady et al. 2014, Borowiec, in prep.).

#### Biology.

Despite relatively high species diversity very little is known about the biology of *Simopone*, although several species have been recorded nesting arboreally (Brown 1975, Bolton and Fisher 2012). One species, the Madagascan *Simopone sicaria*, was observed during a raid. The ants took the brood of arboreal *Terataner alluaudi* as prey (Bolton and Fisher 2012).

Brood production appears not to be synchronized (author’s observations).

#### Species of *Simopone*

*Simopone amana* Bolton and Fisher, 2012: Gabon

*Simopone annettae* Kutter, 1976: Cameroon

*Simopone bakeri* Menozzi, 1926: Singapore

*Simopone brunnea* Bolton and Fisher, 2012: Gabon

*Simopone chapmani* Taylor, 1966: Philippines

*Simopone conradti* Emery, 1899b: Cameroon

*Simopone consimilis* Bolton and Fisher, 2012: Madagascar

*Simopone dignita* Bolton and Fisher, 2012: Madagascar

*Simopone dryas* Bolton and Fisher, 2012: Kenya

*Simopone dux* Bolton and Fisher, 2012: Madagascar

*Simopone elegans* Bolton and Fisher, 2012: Madagascar

*Simopone emeryi* Forel, 1892b: Madagascar

*Simopone fera* Bolton and Fisher, 2012: Madagascar

*Simopone fulvinodis* Santschi, 1923b: Democratic Republic of the Congo

*Simopone grandidieri* Forel, 1891: Madagascar

*Simopone grandis* Santschi, 1923b: Democratic Republic of the Congo

*Simopone gressitti* Taylor, 1965: Indonesia (Papua)

*Simopone inculta* Bolton and Fisher, 2012: Madagascar

*Simopone laevissima* Arnold, 1954: Uganda

*Simopone latiscapa* Bolton and Fisher, 2012: Ghana

*Simopone marleyi* Arnold, 1915: South Africa

*Simopone matthiasi* Kutter, 1977: Cameroon

*Simopone mayri* Emery, 1911: Madagascar

*Simopone merita* Bolton and Fisher, 2012: Madagascar

*Simopone miniflava* Bolton and Fisher, 2012: Gabon

*Simopone nonnihil* Bolton and Fisher, 2012: Madagascar

*Simopone occulta* Bolton and Fisher, 2012: Gabon

*Simopone oculata* Radchenko, 1993: Vietnam

*Simopone persculpta* Bolton and Fisher, 2012: Kenya

*Simopone rabula* Bolton and Fisher, 2012: Tanzania

*Simopone rex* Bolton and Fisher, 2012: Madagascar

*Simopone schoutedeni* Santschi, 1923b: Democratic Republic of the Congo

*Simopone sicaria* Bolton and Fisher, 2012: Madagascar

*Simopone silens* Bolton and Fisher, 2012: Madagascar

*Simopone trita* Bolton and Fisher, 2012: Madagascar

*Simopone vepres* Bolton and Fisher, 2012: Ghana

*Simopone victrix* Bolton and Fisher, 2012: Madagascar

*Simopone wilburi* Weber, 1949a: Democratic Republic of the Congo

*Simopone yunnanensis* Chen, Zhou and Liang, 2015: China

### Sphinctomyrmex

Taxon classificationAnimaliaHymenopteraFormicidae

Mayr, 1866b

#### Type-species.

*Sphinctomyrmex stali*, by monotypy.

*Sphinctomyrmex* is a Neotropical lineage of extremely rarely encountered ants. Nothing is known about their biology.

#### Diagnosis.

***Worker.*** Workers of *Sphinctomyrmex* are among the dorylines with prominent girdling constrictions between abdominal segments IV, V, and VI. These include *Aenictogiton*, *Eusphinctus*, *Leptanilloides*, and *Zasphinctus*. *Sphinctomyrmex* can be differentiated from these genera by a combination of presence of propodeal lobes and propodeal spiracle positioned high (no lobes and spiracle low on propodeum in *Aenictogiton*), metapleural gland trench narrow (broad in *Zasphinctus*), large pygidium armed with modified setae (pygidium unarmed and reduced to a narrow strip in *Leptanilloides*), and girdling constriction between abdominal segments III and IV cross-ribbed and segment IV similar in size to segments V and VI (girdling constriction smooth and segment IV larger than V and VI in *Eusphinctus*). Among these genera, only *Leptanilloides* occurs in sympatry with *Sphinctomyrmex*.

***Male.*** The males of *Sphinctomyrmex* also show girdling constrictions between abdominal segments III, IV, and V. Among male dorylines, this state is restricted to the Old World taxa *Eusphinctus* and *Zasphinctus*. They differ in wing venation, shape of abdominal sternite IX and genitalia and the venation characters are the easiest to assess for identification. The marginal cell is closed in *Sphinctomyrmex* (open in *Eusphinctus*) and the costal vein (C) is present in the fore wing (absent in *Zasphinctus*). Malagasy *Tanipone* may also have weak abdominal constrictions but are distinguished by very long, 6-segmented maxillary palps that are visible in mounted specimens and reach occipital foramen. Some *Acanthostichus* or *Cylindromyrmex* males may also have gastral constrictions but in the former helcium is broad and supraaxial and in the latter there are two spurs on hind tibiae.

#### Description.

***Worker.*** *Head*: Antennae with 12 segments. Apical antennal segment conspicuously enlarged, much broader than and longer than two preceding segments combined. Clypeus with cuticular apron. Lateroclypeal teeth absent. Parafrontal ridges reduced. Torulo-posttorular complex vertical. Antennal scrobes absent. Labrum with median notch or concavity. Proximal face of stipes projecting beyond inner margin of sclerite, concealing prementum when mouthparts fully closed. Maxillary palps 3-segmented. Labial palps 3-segmented. Mandibles triangular, edentate. Eyes present, composed of 1–5 ommatidia. Ocelli absent. Head capsule with differentiated vertical posterior surface above occipital foramen. Ventrolateral margins of head without lamella or ridge extending towards mandibles and beyond carina surrounding occipital foramen. Posterior head corners dorsolaterally immarginate. Carina surrounding occipital foramen ventrally present. *Mesosoma*: Pronotal flange separated from collar by distinct ridge. Promesonotal connection with Pronotomesopleural suture completely fused or with Pronotomesopleural suture present, weakly differentiated, immobile. Pronotomesopleural suture completely fused but impressed line present. Mesometapleural groove weakly impressed. Transverse groove dividing mesopleuron present. Pleural endophragmal pit concavity present. Mesosoma dorsolaterally immarginate. Metanotal depression or groove on mesosoma absent. Propodeal spiracle situated low on sclerite. Propodeal declivity with distinct dorsal edge or margin and rectangular in posterior view. Metapleural gland with bulla visible through cuticle. Propodeal lobes present, well developed. *Metasoma*: Petiole anterodorsally immarginate, dorsolaterally immarginate, and laterally above spiracle marginate. Helcium in relation to tergosternal Pronotomesopleural suture placed at posttergite and axial. Prora forming a U-shaped margin with median ridge. Spiracle openings of abdominal segments IV–VI circular. Abdominal segment III anterodorsally immarginate and dorsolaterally immarginate. Abdominal segment III more than half size of succeeding segment IV, which is weakly constricted at presegmental portion (uninodal waist). Girdling constriction of segment IV present, i.e. pre- and postsclerites distinct. Cinctus of abdominal segment IV gutter-like and cross-ribbed. Abdominal segment IV not conspicuously largest segment. Abdominal tergite IV not folding over sternite, and anterior portions of sternite and tergite equally well visible in lateral view. Girdling constriction between pre- and posttergites of abdominal segments V and VI present. Girdling constriction between pre- and poststernites of abdominal segments V and VI present. Pygidium large, with impressed medial field, and armed with modified setae. Hypopygium unarmed. *Legs*: Mid tibia with single pectinate spur. Hind tibia with single pectinate spur. Hind basitarsus not widening distally, circular in cross-section. Posterior flange of hind coxa not produced as raised lamella. Metatibial gland absent. Metabasitarsal gland absent. Hind pretarsal claws simple. *Polymorphism*: Monomorphic.

***Male.*** *Head*: Antennae with 13 segments. Clypeus with cuticular apron. Parafrontal ridges absent. Torulo-posttorular complex vertical. Maxillary palps unknown. Labial palps unknown. Mandibles triangular, edentate. Ventrolateral margins of head without lamella or ridge extending towards mandibles and beyond carina surrounding occipital foramen. Carina surrounding occipital foramen ventrally absent. *Mesosoma*: Pronotal flange not separated from collar by distinct ridge. Notauli present. Transverse groove dividing mesopleuron present. Propodeal declivity with dorsal edge present, incomplete. Metapleural gland opening present. Propodeal lobes present. *Metasoma*: Petiole anterodorsally immarginate, dorsolaterally immarginate, and laterally above spiracle immarginate. Helcium in relation to tergosternal Pronotomesopleural suture placed at posttergite and axial. Prora forming a U-shaped margin with median ridge. Spiracle openings of abdominal segments IV–VI circular. Abdominal segment III more than half size of succeeding segment IV; latter weakly constricted at presegmental portion (uninodal waist). Girdling constriction of segment IV present, i.e. pre- and postsclerites distinct. Cinctus of abdominal segment IV gutter-like and cross-ribbed. Girdling constriction between pre- and postsclerites of abdominal segments V and VI present. Abdominal segment IV not conspicuously largest segment. Abdominal sternite VII simple. Abdominal sternite IX distally armed with two spines, with lateral apodemes about as long as medial apodeme, directed anteriorly (towards head). *Genitalia*: Cupula long relative to rest of genital capsule and shorter ventrally than dorsally. Basimere broadly fused to telomere, with no sulcus trace at junction, ventrally with left and right arms abutting. Telomere gradually tapering toward apex. Volsella laterally flattened, at apex with dorsal lobe and hooked ventrally. Penisvalva laterally compressed, rounded at apex. *Legs*: Mid tibia with single pectinate spur. Hind tibia with single pectinate spur. Posterior flange of hind coxa not produced as raised lamella. Metatibial gland absent. Metabasitarsal glands absent. Hind pretarsal claws simple. *Wings*: Tegula present, broad, demiovate in shape. Vein C in fore wing present. Pterostigma broad. Abscissa R·f3 present, running toward distal wing margin and enclosing cell with Rs·f5. Abscissae Rs·f2–3 absent or present, very short and disconnected from Rs+M. Cross-vein 2r-rs present, forming base of 2r-rs&Rs·f4–5 in absence of 2rs-m or differentiated from Rs·f4 by presence of short Rs·f2–3. Abscissae Rs·f4–5 present, fused in absence of 2rs-m. Abscissa M·f2 in fore wing contiguous with Rs+M. Abscissa M·f4 in fore wing present, not reaching wing margin. Cross-vein 1m-cu in fore wing present. Cross-vein cu-a in fore wing present, arising from M+Cu and proximal to M·f1. Vein Cu in fore wing present, with only Cu1 branch prominent. Vein A in fore wing with abscissae A·f1 and A·f2 present. Vein C in hind wing absent. Vein R in hind wing present, extending past Sc+R but not reaching distal wing margin. Vein Sc+R in hind wing present. Abscissa Rs·f1 in hind wing present, longer than 1rs-m. Abscissa Rs·f2 in hind wing present, short, not reaching wing margin. Cross-vein 1rs-m in hind wing absent. Vein M+Cu in hind wing present. Abscissa M·f1 in hind wing absent. Abscissa M·f2 in hind wing absent. Cross-vein cu-a in hind wing absent or stub present. Vein Cu in hind wing present. Vein A in hind wing with abscissa A·f1 present.

***Gyne.*** Gynes are so far only known for *Sphinctomyrmex stali*. One apparently dealated gyne has been collected in this species, in addition to ergatoid/intercaste specimens with relatively large eyes and ocelli. For a detailed discussion see Feitosa et al. (2011).

***Larva.*** Not described. Cocoons unknown.

#### Distribution.

So far only known from Amazonas, Santa Catarina, and São Paulo states in Brazil, and Jujuy province in Argentina but likely present throughout most of South America.

#### Taxonomy and phylogeny.

For taxonomic history see under *Eusphinctus*.

The three known species of *Sphinctomyrmex* are reviewed and keyed in Feitosa et al. (2011).

The affinities of the genus are not known exactly but genomic data suggests that it forms a clade with *Leptanilloides* and the *Eciton* genus-group (Borowiec, in prep.).

#### Biology.

Virtually nothing is known of this lineage’s biology, and no nest series have ever been collected (Feitosa et al. 2011). Several workers have been collected by digging in soil in a dry forest habitat in Jujuy, Argentina (Brian Fisher pers. comm.).

#### Species of *Sphinctomyrmex*

*Sphinctomyrmex marcoyi* Feitosa, Brandão, Fernández and Delabie, 2011: Brazil

*Sphinctomyrmex schoerederi* Feitosa, Brandão, Fernández and Delabie, 2011: Brazil

*Sphinctomyrmex stali* Mayr, 1866b: Brazil

### Syscia

Taxon classificationAnimaliaHymenopteraFormicidae

Roger, 1861 gen. rev.

#### Type-species.

*Syscia typhla*, by monotypy.

*Syscia* is the only doryline genus with a disjunct distribution between the Old and New World, and includes many cryptic, undescribed species.

#### Diagnosis.

***Worker.*** *Syscia* workers have 11- or 9-segmented antennae, eyes small to absent, and are usually heavily sculptured with abundant body pilosity. Body is usually uniformly colored and ranges from yellow through reddish to dark brown but never black. They possess apparently autapomorphic characters that serve to easily distinguish this lineage from all other dorylines: basal segment of hind tarsus widening distally with a light patch of cuticle on the inner (flexor) side, and abdominal tergite IV anteriorly folding over sternite. This combination is unique to *Syscia* and although species in other lineages may have similar habitus (*Ooceraea*, *Parasyscia*), none of these possess these characteristics.

***Male.*** The males of *Syscia* have the number of antennal segments reduced to 12. They can be difficult to distinguish from *Ooceraea* (see under diagnosis for that genus), but a lack of constrictions between abdominal segments IV, V, and VI, presence of a spur on middle tibia, and no costal vein (C) in the fore wing will distinguish them from the other genera where a reduction in the number of antennal segments is currently known, which include *Acanthostichus*, *Eusphinctus*, and *Simopone*.

#### Description.

***Worker.*** *Head*: Antennae with 9 or 11 segments. Apical antennal segment conspicuously enlarged, much broader than and longer than two preceding segments combined. Clypeus with cuticular apron. Lateroclypeal teeth present. Parafrontal ridges reduced. Torulo-posttorular complex vertical. Antennal scrobes absent. Labrum with median notch or concavity. Proximal face of stipes projecting beyond inner margin of sclerite, concealing prementum when mouthparts fully closed. Maxillary palps 2-segmented. Labial palps 2-segmented. Mandibles triangular, edentate. Eyes absent or present, composed of 1–5 ommatidia. Ocelli absent. Head capsule with differentiated vertical posterior surface above occipital foramen. Ventrolateral margins of head without lamella or ridge extending towards mandibles and beyond carina surrounding occipital foramen. Posterior head corners dorsolaterally immarginate. Carina surrounding occipital foramen ventrally present. *Mesosoma*: Pronotal flange not separated from collar by distinct ridge. Promesonotal connection with Pronotomesopleural suture completely fused. Pronotomesopleural suture visible, unfused up to notal surface. Mesometapleural groove not impressed. Mesometapleural groove weakly impressed. Transverse groove dividing mesopleuron present or absent. Pleural endophragmal pit concavity present. Mesosoma dorsolaterally immarginate. Metanotal depression or groove on mesosoma absent. Propodeal spiracle situated low on sclerite. Propodeal declivity without distinct dorsal edge or margin and rectangular in posterior view. Metapleural gland with bulla visible through cuticle. Propodeal lobes present, well developed. *Metasoma*: Petiole anterodorsally immarginate, dorsolaterally immarginate, and laterally above spiracle immarginate. Helcium in relation to tergosternal Pronotomesopleural suture placed at posttergite and axial. Prora forming a U-shaped margin with median ridge. Spiracle openings of abdominal segments IV–VI circular. Abdominal segment III anterodorsally immarginate and dorsolaterally immarginate. Abdominal segment III more than half size of succeeding segment IV, which is weakly constricted at presegmental portion (uninodal waist). Girdling constriction of segment IV present, i.e. pre- and postsclerites distinct. Cinctus of abdominal segment IV gutter-like, not sculptured or cross-ribbed. Abdominal segment IV not conspicuously largest segment or conspicuously largest segment. Abdominal tergite IV folding over sternite, anterior portion of sternite concealing tergite in lateral view. Girdling constriction between pre- and posttergites of abdominal segments V and VI absent. Girdling constriction between pre- and poststernites of abdominal segments V and VI absent. Pygidium medium-sized, with impressed medial field, and armed with modified setae. Hypopygium unarmed or armed with modified setae. *Legs*: Mid tibia with single pectinate spur. Hind tibia with single pectinate spur. Hind basitarsus widening distally, oval in cross-section. Posterior flange of hind coxa not produced as raised lamella. Metatibial gland present as oval patch of whitish cuticle. Metabasitarsal gland present. Hind pretarsal claws simple. *Polymorphism*: Monomorphic.

***Male.*** *Head*: Antennae with 12 segments. Clypeus with cuticular apron. Parafrontal ridges present. Torulo-posttorular complex vertical. Maxillary palps 4-segmented. Labial palps 2-segmented. Mandibles triangular, edentate. Ventrolateral margins of head without lamella or ridge extending towards mandibles and beyond carina surrounding occipital foramen. Carina surrounding occipital foramen ventrally present. *Mesosoma*: Pronotal flange not separated from collar by distinct ridge. Notauli present. Transverse groove dividing mesopleuron present. Propodeal declivity reduced, without distinct dorsal edge or margin. Metapleural gland opening absent. Propodeal lobes present. *Metasoma*: Petiole anterodorsally immarginate, dorsolaterally immarginate, and laterally above spiracle marginate. Helcium in relation to tergosternal Pronotomesopleural suture placed at posttergite and axial. Prora forming a U-shaped margin with median ridge. Spiracle openings of abdominal segments IV–VI circular. Abdominal segment III more than half size of succeeding segment IV; latter weakly constricted at presegmental portion (uninodal waist). Abdominal segment III about half size of succeeding segment IV or less; latter strongly constricted at presegmental portion (binodal waist). Girdling constriction of segment IV present, i.e. pre- and postsclerites distinct. Cinctus of abdominal segment IV gutter-like and cross-ribbed. Girdling constriction between pre- and postsclerites of abdominal segments V and VI absent. Abdominal segment IV conspicuously largest segment. Abdominal sternite VII simple. Abdominal sternite IX distally armed with two spines. with lateral apodemes about as long as medial apodeme, directed anteriorly (towards head). *Genitalia*: Cupula long relative to rest of genital capsule and shorter ventrally than dorsally. Basimere broadly fused to telomere, with no sulcus trace at junction, and ventrally with left and right arms abutting. Telomere gradually tapering toward apex. Volsella not tapering much toward apex, relatively broad. Penisvalva laterally flattened, at apex hooked ventrally, in Neotropical forms also apparently curving outwards. *Legs*: Mid tibia with single pectinate spur. Hind tibia with single pectinate spur. Posterior flange of hind coxa not produced as raised lamella. Metatibial gland present as oval patch of whitish cuticle. Metabasitarsal glands absent. Hind pretarsal claws simple. *Wings*: Tegula present, broad, demiovate in shape. Vein C in fore wing absent. Pterostigma broad. Abscissa R·f3 absent. Abscissae Rs·f2–3 absent. Cross-vein 2r-rs present, forming base of ‘free stigmal vein’ (2r-rs&Rs·f4–5) in absence of Rs·f3 and 2rs-m. Abscissae Rs·f4–5 present, fused in absence of 2rs-m. Abscissa M·f2 in fore wing contiguous with Rs+M. Abscissa M·f4 in fore wing present, short, not reaching wing margin. Cross-vein 1m-cu in fore wing absent. Cross-vein cu-a in fore wing present, arising from M+Cu and proximal to M·f1. Vein Cu in fore wing absent past M+Cu. Vein A in fore wing with abscissae A·f1 and A·f2 present. Vein C in hind wing absent. Vein R in hind wing absent. Vein Sc+R in hind wing present. Abscissa Rs·f1 in hind wing present, longer than 1rs-m. Abscissa Rs·f2 in hind wing present, not reaching wing margin. Cross-vein 1rs-m in hind wing absent or present, about as long as M·f1. Vein M+Cu in hind wing present. Abscissa M·f1 in hind wing absent. Abscissa M·f2 in hind wing absent. Cross-vein cu-a in hind wing absent. Vein Cu in hind wing absent. Vein A in hind wing with abscissa A·f1 present.

***Gyne.*** Alate, brachypterous, or ergatoid with eyes of variable size and with or without ocelli. Gynes are known for *Syscia augustae*, *Syscia honduriana*, *Syscia humicola*, and *Syscia typhla*. In *Syscia typhla* the gynes have well-developed flight sclerites on the mesosoma but I have never examined a specimen with wings. Field observations of *Syscia augustae* or a closely related species suggest that virgin queens may be brachypterous (Michael Branstetter pers. comm.). Ergatoid queens have been reported in *Syscia humicola* (Ogata 1983), and their morphology is very similar to that of the worker, except for larger size, presence of compound eyes and a single ocellus in some but not all gynes. Confirmed alate or apparently dealated gynes are so far known only in undescribed forms from both Old and New World.

***Larva.*** Described for *Syscia augustae* (Wheeler 1903b). Cocoons absent.

#### Distribution.

This is the only doryline genus with a disjunct distribution between the New and Old Worlds (except for tramp *Ooceraea biroi*), with one center of diversity in Central America, with records from the Antilles (Cuba and Dominican Republic) and as far north as Arkansas, United States, and the other center in Southeast Asia west of Wallace’s Line, especially Borneo, reaching Japan to the north and southern India to the west.

#### Taxonomy and phylogeny.

*Syscia* was described by Roger (1861) in his paper on ‘*Ponera*-like ants’. Forel (1893a) included it in his newly erected ‘Cerapachysii’ and subsequently it was treated as either a genus (e.g. Forel 1900b, Bingham 1903) or a subgenus of *Cerapachys* (e.g. Wheeler 1902, Emery 1911e) until Kempf (1972) treated it as a synonym of the latter.

*Syscia* is here recognized as a valid genus, following the molecular evidence that Neotropical and Indomalayan species form a clade (Brady et al. 2014, Borowiec, in prep.) and because species related to *typhla* are easily distinguished from a closely related group, *Ooceraea*. There are five species currently described but at least fifteen additional morphospecies present in collections from the Old World and more than 30 undescribed species in the New World (Theodore Sumnicht pers. comm.).

This lineage belongs to a clade with *Eusphinctus* and *Ooceraea* (Borowiec, in prep.). No attempts of reconstructing the internal phylogeny have been made.

#### Biology.

*Syscia* species are found in leaf litter samples and soil cores. The foraging habits are not known. *Syscia augustae*, a species present in southern United States, has been observed on diurnal emigrations and briefly studied under laboratory conditions (Clint Penick pers. comm.). The brood production in *Syscia augustae* is synchronized. Gyne morphology varies in this lineage, with ergatoid, brachypterous, and fully winged individuals known. There is an observation of a brachypterous gyne aggregation under a stone (Michael Branstetter pers. comm.). It is unknown whether this represents cooperative colony foundation.

#### Species of *Syscia*

*Syscia augustae* (Wheeler, W. M., 1902): United States, **comb. n.**

*Syscia honduriana* (Mann, 1922): Honduras, **comb. n.**

*Syscia humicola* (Ogata, 1983): Japan, **comb. n.**

*Syscia tolteca* (Forel, 1909a): Guatemala, **comb. n.**

*Syscia typhla* Roger, 1861: Sri Lanka, **comb. rev.**

### Tanipone

Taxon classificationAnimaliaHymenopteraFormicidae

Bolton & Fisher, 2012

#### Type-species.

*Tanipone hirsuta*, by original designation.

*Tanipone* is a small genus with unknown biology, endemic to arid and semi-arid habitats of Madagascar.

#### Diagnosis.

***Worker.*** *Tanipone* workers are distinctive ants with large eyes and ocelli, very long palps and unique glandular patches on abdominal segment III. The latter are present in all species except one (*Tanipone aglandula*). The body coloration is black or bicolored reddish and black, with a light band or two light spots present at the posterior edge of abdominal segment III. The workers of *Tanipone* lack a conspicuous mid tibial spur. Other genera without the spur include *Simopone* and *Vicinopone*. Additionally, in certain species of *Lioponera* the mid tibial spur may be reduced and not easily discernible. *Tanipone* workers can be easily distinguished from all three lineages by remarkably long palps that are always visible on preserved specimens, reaching the occipital foramen.

***Male.*** The male of *Tanipone* is also easily distinguished from all other dorylines by the long maxillary palps, almost always extruded and reaching occipital foramen. The wing venation is variously developed but the submarginal cell (SMC) is open or closed by a faint 2rs-m cross-vein. There are no notauli and no spurs on middle tibiae.

#### Description.

***Worker.*** *Head*: Antennae with 12 segments. Apical antennal segment not enlarged, not broader and longer than two preceding segments combined. Clypeus without cuticular apron. Lateroclypeal teeth absent. Parafrontal ridges reduced. Torulo-posttorular complex vertical. Antennal scrobes absent. Labrum with median notch or concavity. Proximal face of stipes projecting beyond inner margin of sclerite, concealing prementum when mouthparts fully closed. Maxillary palps 6-segmented. Labial palps 4-segmented. Mandibles triangular, edentate. Eyes present, composed of more than 20 ommatidia. Ocelli present. Head capsule with differentiated vertical posterior surface above occipital foramen. Ventrolateral margins of head without lamella or ridge extending towards mandibles and beyond carina surrounding occipital foramen. Posterior head corners dorsolaterally immarginate. Carina surrounding occipital foramen ventrally present. *Mesosoma*: Pronotal flange not separated from collar by distinct ridge. Promesonotal connection with Pronotomesopleural suture completely fused. Pronotomesopleural suture visible, unfused up to notal surface. Mesometapleural groove deeply impressed, conspicuous. Transverse groove dividing mesopleuron present. Pleural endophragmal pit concavity present. Mesosoma dorsolaterally immarginate. Metanotal depression or groove on mesosoma absent or a weakly impressed line. Propodeal spiracle situated low on sclerite. Propodeal declivity with distinct dorsal edge or margin and rectangular in posterior view. Metapleural gland without bulla visible through cuticle. Propodeal lobes present, well developed. *Metasoma*: Petiole anterodorsally marginate, dorsolaterally immarginate, and laterally above spiracle marginate. Helcium in relation to tergosternal Pronotomesopleural suture placed at posttergite and axial. Prora simple, not delimited by carina. Spiracle openings of abdominal segments IV–VI circular. Abdominal segment III anterodorsally immarginate and dorsolaterally immarginate. Abdominal segment III more than half size of succeeding segment IV, which is weakly constricted at presegmental portion (uninodal waist). Girdling constriction of segment IV present, i.e. pre- and postsclerites distinct. Cinctus of abdominal segment IV gutter-like, not sculptured. Cinctus of abdominal segment IV gutter-like and smooth or cross-ribbed. Abdominal segment IV not conspicuously largest segment. Abdominal tergite IV not folding over sternite, and anterior portions of sternite and tergite equally well visible in lateral view. Girdling constriction between pre- and posttergites of abdominal segments V and VI absent. Girdling constriction between pre- and poststernites of abdominal segments V and VI present or absent. Pygidium large, with weakly impressed medial field, and armed with few modified setae restricted to apex. Hypopygium unarmed. *Legs*: Mid tibia without spurs. Hind tibia with single pectinate spur. Hind basitarsus not widening distally, circular in cross-section. Posterior flange of hind coxa not produced as raised lamella. Metatibial gland present as oval patch of whitish cuticle. Metabasitarsal gland absent. Hind pretarsal claws each armed with a tooth. *Polymorphism*: Monomorphic.

***Male.*** *Head*: Antennae with 13 segments. Clypeus without cuticular apron. Parafrontal ridges absent. Torulo-posttorular complex vertical. Maxillary palps 6-segmented. Labial palps 4-segmented. Mandibles triangular, edentate. Ventrolateral margins of head without lamella or ridge extending towards mandibles and beyond carina surrounding occipital foramen. Carina surrounding occipital foramen ventrally present. *Mesosoma*: Pronotal flange separated from collar by distinct ridge or not. Notauli absent. Transverse groove dividing mesopleuron present. Propodeal declivity with distinct dorsal edge or margin. Metapleural gland opening absent. Propodeal lobes present. *Metasoma*: Petiole anterodorsally immarginate, dorsolaterally immarginate, and laterally above spiracle marginate. Helcium in relation to tergosternal Pronotomesopleural suture placed at posttergite and axial. Prora forming a simple U-shaped margin or U-shaped protrusion. Spiracle openings of abdominal segments IV–VI circular. Abdominal segment III more than half size of succeeding segment IV; latter weakly constricted at presegmental portion (uninodal waist). Girdling constriction of segment IV present, i.e. pre- and postsclerites distinct. Cinctus of abdominal segment IV gutter-like and cross-ribbed. Girdling constriction between pre- and postsclerites of abdominal segments V and VI absent. Abdominal segment IV not conspicuously largest segment. Abdominal sternite VII simple. Abdominal sternite IX distally armed with two spines, with lateral apodemes about as long as medial apodeme, directed anteriorly (towards head). *Genitalia*: Cupula long relative to rest of genital capsule and shorter ventrally than dorsally. Basimere broadly fused to telomere, with no sulcus trace at junction, and ventrally with left and right arms separated. Telomere gradually tapering toward apex. Volsella gradually tapering toward apex. Penisvalva laterally compressed, rounded at apex. *Legs*: Mid tibia without spurs. Hind tibia with single pectinate spur. Posterior flange of hind coxa not produced as raised lamella. Metatibial gland absent. Metabasitarsal glands absent. Hind pretarsal claws simple. Hind pretarsal claws each armed with a tooth. *Wings*: Tegula present, broad, demiovate in shape. Vein C in fore wing absent. Pterostigma broad. Abscissa R·f3 absent. Abscissae Rs·f2–3 absent. Cross-vein 2r-rs absent, present and forming base of ‘free stigmal vein’ (2r-rs&Rs·f4–5) in absence of Rs·f3 and 2rs-m, or present and connected to Rs·f2–3&Rs·f4. Abscissae Rs·f4–5 absent or differentiated into Rs·f4 and Rs·f5 by 2rs-m. Abscissa M·f2 in fore wing absent or contiguous with Rs+M. Abscissa M·f4 in fore wing absent or present, reaching wing margin. Cross-vein 1m-cu in fore wing absent or present. Cross-vein cu-a in fore wing present, arising from M+Cu and proximal to M·f1. Vein Cu in fore wing present, with only Cu1 branch prominent. Vein A in fore wing with only abscissa A·f1 present or with abscissae A·f1 and A·f2 present. Vein C in hind wing absent. Vein R in hind wing absent. Vein Sc+R in hind wing present. Abscissa Rs·f1 in hind wing present, as long as 1rs-m or longer than 1rs-m. Abscissa Rs·f2 in hind wing absent or present, reaching wing margin. Cross-vein 1rs-m in hind wing absent or present, about as long as M·f1. Vein M+Cu in hind wing present. Abscissa M·f1 in hind wing present. Abscissa M·f2 in hind wing absent. Cross-vein cu-a in hind wing absent or present. Vein Cu in hind wing present. Vein A in hind wing abscissa A·f1 or with abscissae A·f1 and A·f2 present.

***Gyne.*** Presumably extremely ergatoid in all species; specimens identified as putative gynes differ from workers only in sculpturation. See Bolton and Fisher (2012) for a discussion.

***Larva.*** Not described. Cocoons absent.

#### Distribution.

Endemic to Madagascar.

#### Taxonomy and phylogeny.

There are ten *Tanipone* species currently known, all confined to Madagascar (Bolton and Fisher 2012). The position of this lineage is not known, and internal phylogeny has never been investigated, although Bolton and Fisher (2012) assigned the species to three species-groups.

#### Biology.

The known specimens have been collected mostly in a variety of drier habitats in Madagascar, including dry tropical forest, savannah, and spiny forest. Most workers were collected on low vegetation, on the ground or in rot holes on tree trunks. The sole nest sample of *Tanipone* (*Tanipone zona*) was collected under a stone. Nothing is known about feeding habits of this lineage. The putative queen specimens are ergatoid. Based on nest collections where larvae of different sizes and pupae were collected together, known for *Tanipone hirsuta*, *Tanipone subpilosa*, and *Tanipone zona*, brood development appears not synchronized.

#### Species of *Tanipone*

*Tanipone aglandula* Bolton and Fisher, 2012: Madagascar

*Tanipone aversa* Bolton and Fisher, 2012: Madagascar

*Tanipone cognata* Bolton and Fisher, 2012: Madagascar

*Tanipone hirsuta* Bolton and Fisher, 2012: Madagascar

*Tanipone maculata* Bolton and Fisher, 2012: Madagascar

*Tanipone pilosa* Bolton and Fisher, 2012: Madagascar

*Tanipone scelesta* Bolton and Fisher, 2012: Madagascar

*Tanipone subpilosa* Bolton and Fisher, 2012: Madagascar

*Tanipone varia* Bolton and Fisher, 2012: Madagascar

*Tanipone zona* Bolton and Fisher, 2012: Madagascar

### Vicinopone

Taxon classificationAnimaliaHymenopteraFormicidae

Bolton & Fisher, 2012

#### Type-species.

*Simopone conciliatrix*, by original designation.

*Vicinopone* is a monotypic lineage of arboreal ants.

#### Diagnosis.

***Worker.*** The workers of the sole species of *Vicinopone* are slender and small yellowish ants with large eyes and no ocelli. The cuticle is sculptured with weak punctation. *Vicinopone* is recognized by a combination of propodeal spiracle high on the sclerite and propodeal lobes present, no constrictions between abdominal segments IV, V, and VI, large pygidium armed with modified setae, no ocelli in the worker, 12-segmented antennae and no spur on the middle tibiae. This genus is most similar to *Simopone* with which it shares the lack of a mid tibial spur, but in *Simopone* the antennae are 11-segmented and workers have ocelli.

***Male.*** The male of *Vicinopone* is unknown.

#### Description.

***Worker.*** *Head*: Antennae with 12 segments. Apical antennal segment moderately enlarged, broader than and about equal in length to two preceding segments combined. Clypeus without cuticular apron. Lateroclypeal teeth absent. Parafrontal ridges reduced. Torulo-posttorular complex vertical. Antennal scrobes absent. Labrum with median notch or concavity. Proximal face of stipes not projecting beyond inner margin of sclerite, prementum exposed when mouthparts fully closed. Maxillary palps 3-segmented. Labial palps 2-segmented. Mandibles triangular, edentate. Eyes present, composed of more than 20 ommatidia. Ocelli absent. Head capsule without differentiated vertical posterior surface above occipital foramen. Ventrolateral margins of head without lamella or ridge extending towards mandibles and beyond carina surrounding occipital foramen. Posterior head corners dorsolaterally immarginate. Carina surrounding occipital foramen ventrally present. *Mesosoma*: Pronotal flange separated from collar by distinct ridge. Promesonotal connection with Pronotomesopleural suture completely fused. Pronotomesopleural suture visible, unfused partway to notal surface. Mesometapleural groove weakly impressed. Transverse groove dividing mesopleuron absent. Pleural endophragmal pit concavity present. Mesosoma dorsolaterally immarginate. Metanotal depression or groove on mesosoma absent. Propodeal spiracle situated low on sclerite. Propodeal declivity with distinct dorsal edge or margin and rectangular in posterior view. Metapleural gland with bulla visible through cuticle. Propodeal lobes present, well developed. *Metasoma*: Petiole anterodorsally marginate, dorsolaterally immarginate, and laterally above spiracle marginate. Helcium in relation to tergosternal Pronotomesopleural suture placed at posttergite and axial. Prora forming a U-shaped margin with median ridge. Spiracle openings of abdominal segments IV–VI circular. Abdominal segment III anterodorsally immarginate and dorsolaterally immarginate. Abdominal segment III more than half size of succeeding segment IV, which is weakly constricted at presegmental portion (uninodal waist). Girdling constriction of segment IV present, i.e. pre- and postsclerites distinct. Cinctus of abdominal segment IV gutter-like and cross-ribbed. Abdominal segment IV not conspicuously largest segment. Abdominal tergite IV not folding over sternite, and anterior portions of sternite and tergite equally well visible in lateral view. Girdling constriction between pre- and posttergites of abdominal segments V and VI absent. Girdling constriction between pre- and poststernites of abdominal segments V and VI absent. Pygidium large, with impressed medial field, armed with modified setae. Hypopygium unarmed. *Legs*: Mid tibia without spurs. Hind tibia with single pectinate spur. Hind basitarsus not widening distally, circular in cross-section. Posterior flange of hind coxa not produced as raised lamella. Metatibial gland absent. Metabasitarsal gland absent. Hind pretarsal claws each armed with a tooth. *Polymorphism*: Monomorphic.

***Male.*** Not described.

***Gyne.*** Apparently alate, with ocelli and flight sclerites but only dealated specimens known (Bolton and Fisher 2012).

***Larva.*** Not described. Cocoons absent.

#### Distribution.

So far *Vicinopone* has been collected in Ghana, Gabon, Democratic Republic of the Congo, Uganda, and Tanzania but it is likely that it is more widely distributed in sub-Saharan Africa.

#### Taxonomy and phylogeny.

*Vicinopone* is a genus recently established by Bolton and Fisher (2012) to accommodate the only currently known species, *Vicinopone conciliatrix*.

The phylogenetic position of *Vicinopone* is uncertain (Brady et al. 2014, Borowiec, in prep.).

#### Biology.

Little is known of the species’ habits, but the two known nest samples have been taken from dead twigs on trees, suggesting that this is an obligatory arboreal nester. Two dealate gynes were collected with the type nest series (Brown 1975), suggesting that the ant may be polygynous. Brood production is not synchronized, as larvae and pupae of various stages were present in the nests at times of collection.

#### Species of *Vicinopone*

*Vicinopone conciliatrix* (Brown, 1975): Ghana

### Yunodorylus

Taxon classificationAnimaliaHymenopteraFormicidae

Xu, 2000 gen. rev.

#### Type-species.

*Yunodorylus sexspinus*, by original designation.

This is a poorly known genus with striking morphology somewhat reminiscent of *Dorylus*.

#### Diagnosis.

***Worker.*** Workers of *Yunodorylus* are stout ants with no eyes and body coloration ranging from yellow to reddish, with cuticle sculpture and pilosity moderate. *Yunodorylus* is the only non-army ant doryline with a single waist segment and no or very weak girdling constriction on abdominal segment IV. It can be distinguished from army ant dorylines by relatively high positioned propodeal spiracle and presence of propodeal lobes.

***Male.*** The males of *Yunodorylus* have a distinctive habitus with abdominal segment III very broadly attached posteriorly to segment IV and gaster widest towards the apex, posterior of abdominal segment IV. A combination of propodeal lobes well-developed, 13-segmented antennae, helcium relatively narrow and axial, no constrictions between abdominal segments IV, V, and VI, and wings always with veins C, R·f3, and at least a stub of Rs·f2–3 will distinguish *Yunodorylus* males from other genera. Although there may be gutter-like constrictions on abdominal segment IV and beyond, these are restricted to the tergites and do not produce constricted appearance in lateral view as in, for example, *Zasphinctus*. Sharp and conspicuously ventrally hooked penisvalvae appear to be unique among dorylines.

#### Description.

***Worker.*** *Head*: Antennae with 11 or 12 segments. Apical antennal segment not enlarged, not broader and longer than two preceding segments combined. Clypeus without cuticular apron. Lateroclypeal teeth present. Parafrontal ridges absent or reduced. Torulo-posttorular complex vertical. Antennal scrobes absent. Labrum with median notch or concavity. Proximal face of stipes projecting beyond inner margin of sclerite, concealing prementum when mouthparts fully closed. Maxillary palps 2-segmented. Labial palps 2-segmented. Mandibles triangular, with teeth or falcate, with teeth on elongated masticatory margin. Eyes absent. Ocelli absent. Head capsule with differentiated vertical posterior surface above occipital foramen. Ventrolateral margins of head without lamella or ridge extending towards mandibles and beyond carina surrounding occipital foramen. Posterior head corners dorsolaterally immarginate. Carina surrounding occipital foramen ventrally absent. *Mesosoma*: Pronotal flange not separated from collar by distinct ridge. Promesonotal connection with Pronotomesopleural suture completely fused. Pronotomesopleural suture visible, unfused partway to notal surface. Mesometapleural groove weakly impressed. Transverse groove dividing mesopleuron absent. Pleural endophragmal pit concavity present. Mesosoma dorsolaterally immarginate. Metanotal depression or groove on mesosoma absent. Propodeal spiracle situated low on sclerite. Propodeal declivity without distinct dorsal edge or margin and rectangular in posterior view. Metapleural gland with bulla visible through cuticle. Propodeal lobes present, short. *Metasoma*: Petiole anterodorsally, dorsolaterally immarginate, and laterally above spiracle immarginate. Helcium in relation to tergosternal Pronotomesopleural suture placed at posttergite and axial or supraaxial. Prora forming a vertical carina. Spiracle openings of abdominal segments IV–VI circular. Abdominal segment III anterodorsally immarginate and dorsolaterally immarginate. Abdominal segment III more than half size of succeeding segment IV, which is weakly constricted at presegmental portion (uninodal waist). Girdling constriction of segment IV absent, i.e. pre- and postsclerites indistinct. Cinctus of abdominal segment IV not impressed. Abdominal segment IV not conspicuously largest segment. Abdominal tergite IV not folding over sternite, and anterior portions of sternite and tergite equally well visible in lateral view. Girdling constriction between pre- and posttergites of abdominal segments V and VI absent. Girdling constriction between pre- and poststernites of abdominal segments V and VI absent. Pygidium medium-sized, with impressed medial field, and armed with cuticular spines. Hypopygium unarmed. *Legs*: Mid tibia with pectinate and simple spur. Hind tibia with pectinate and simple spur. Hind basitarsus not widening distally, circular in cross-section. Posterior flange of hind coxa not produced as raised lamella. Metatibial gland present as oval patch of whitish cuticle. Metabasitarsal gland absent. Hind pretarsal claws simple. *Polymorphism*: Monomorphic to moderately polymorphic.

***Male.*** *Head*: Antennae with 13 segments. Clypeus with cuticular apron. Parafrontal ridges absent. Torulo-posttorular complex vertical. Maxillary palps 3-segmented. Labial palps 2-segmented. Mandibles triangular, edentate to falcate. Ventrolateral margins of head without lamella or ridge extending towards mandibles and beyond carina surrounding occipital foramen. Carina surrounding occipital foramen ventrally absent. *Mesosoma*: Pronotal flange not separated from collar by distinct ridge. Notauli absent. Transverse groove dividing mesopleuron absent or present. Propodeal declivity reduced, without distinct dorsal edge or margin. Metapleural gland opening absent or present. Propodeal lobes present. *Metasoma*: Petiole anterodorsally immarginate, dorsolaterally immarginate, and laterally above spiracle immarginate or marginate. Helcium in relation to tergosternal Pronotomesopleural suture placed at posttergite and axial. Prora forming a simple U-shaped margin. Spiracle openings of abdominal segments IV–VI circular. Abdominal segment III more than half size of succeeding segment IV; latter weakly constricted at presegmental portion (uninodal waist). Girdling constriction of segment IV present, i.e. pre- and postsclerites distinct. Cinctus of abdominal segment IV gutter-like, not sculptured. Girdling constriction between pre- and postsclerites of abdominal segments V and VI absent. Abdominal segment IV not conspicuously largest segment. Abdominal sternite VII simple. Abdominal sternite IX distally armed with two spines, with lateral apodemes about as long as medial apodeme, directed anteriorly (towards head). *Genitalia*: Cupula short relative to rest of genital capsule and shorter ventrally than dorsally. Basimere broadly fused to telomere, with no sulcus trace at junction, and ventrally with left and right arms abutting. Telomere gradually tapering toward apex. Volsella gradually tapering toward apex. Penisvalva hook-like, strongly curved ventrally. *Legs*: Mid tibia with single pectinate spur. Hind tibia with single pectinate spur. Posterior flange of hind coxa not produced as raised lamella. Metatibial gland absent. Metabasitarsal glands absent. Hind pretarsal claws simple. *Wings*: Tegula present, broad, demiovate in shape. Vein C in fore wing present. Pterostigma broad. Abscissa R·f3 present and running toward distal wing margin and enclosing cell with Rs·f5 or not. Abscissae Rs·f2–3 present, disconnected from Rs+M, rarely closely approaching Rs+M. Cross-vein 2r-rs present, connected to Rs·f2–3&Rsf4. Abscissae Rs·f4–5 present, fused in absence of 2rs-m. Abscissa M·f2 in fore wing contiguous with Rs+M. Abscissa M·f4 in fore wing present, reaching wing margin. Cross-vein 1m-cu in fore wing present. Cross-vein cu-a in fore wing present, arising from M+Cu and proximal to M·f1. Vein Cu in fore wing present, with only Cu1 branch prominent. Vein A in fore wing with abscissae A·f1 and A·f2 present. Vein C in hind wing absent. Vein R in hind wing absent. Vein Sc+R in hind wing present. Abscissa Rs·f1 in hind wing present, shorter than 1rs-m. Abscissa Rs·f2 in hind wing present, not reaching wing margin. Cross-vein 1rs-m in hind wing present, about as long as M·f1. Vein M+Cu in hind wing present. Abscissa M·f1 in hind wing present. Abscissa M·f2 in hind wing present. Cross-vein cu-a in hind wing absent. Vein Cu in hind wing present. Vein A in hind wing with abscissa A·f1 present.

***Gyne.*** A detailed description of a gyne of *Yunodorylus* is currently in preparation (Eguchi et al. in press).

***Larva.*** Not described. Presence of cocoons unknown.

#### Distribution.

This is a species-poor lineage, apparently restricted to mainland Southeast Asia and Borneo, although it is possible it will be eventually found on other islands.

#### Taxonomy and phylogeny.

The genus was originally established for *Yunodorylus sexspinus* by Xu (2000) from Yunnan, China and synonymized by Bolton (2003) under *Cerapachys*. In a recent contribution I provided descriptions and a key to the four species so far known from workers (Borowiec 2009).

Based on genomic data, *Yunodorylus* is part of a well-supported clade also including *Cerapachys* and *Chrysapace*.

#### Biology.

Very little is known about this lineage. *Yunodorylus eguchii* nests in soil in evergreen forests and dry dwarf forests in southern Vietnam. A colony sample (type series) of *Yunodorylus eguchii* contain larvae of various sizes. The field and laboratory observations of two queen-right colonies of *Yunodorylus eguchii* will be reported by Eguchi et al. (in press) and Mizuno et al. (in prep.).

#### Species of *Yunodorylus*

*Yunodorylus doryloides* (Borowiec, 2009): Malaysia (Sarawak), **comb. n.**

*Yunodorylus eguchii* (Borowiec, 2009): Vietnam, **comb. n.**

*Yunodorylus paradoxus* (Borowiec, 2009): Malaysia (Sarawak), **comb. n.**

*Yunodorylus sexspinus* Xu, 2000: China, **comb. rev.**

### Zasphinctus

Taxon classificationAnimaliaHymenopteraFormicidae

Wheeler, W. M., 1918 gen. rev.

= Aethiopopone Santschi, 1930, **syn. n.**
= Nothosphinctus Wheeler, W. M. 1918, **syn. n.**

#### Type-species.

*Sphinctomyrmex turneri*, by monotypy.

*Zasphinctus* is a moderately speciose lineage of specialized ant predators, most prominent in Australia.

#### Diagnosis.

***Worker.*** The workers of *Zasphinctus* are ants of variable size, color, and sculpturation, but always possessing conspicuous girdling constrictions between abdominal segments IV, V, and VI. The eyes absent in most species. *Zasphinctus* can be distinguished from other lineages with pronounced abdominal constrictions by highly-positioned propodeal spiracles, propodeal lobes present, pygidium large and armed with modified setae, and pronotomesopleural Pronotomesopleural suture fused. See also diagnoses of *Eusphinctus* and *Sphinctomyrmex*.

***Male.*** The males of *Zasphinctus* also possess the characteristic abdominal constrictions between abdominal segments IV, V, and VI and can be recognized by a combination of costal vein (C) absent from the fore wing, submarginal cell (SMC) closed by Rs·f2-f3, vein 2rs-m absent, pronotum not marginate anterodorsally, and antennae 13-segmented. This venation is similar to *Lividopone* and *Parasyscia* but *Zasphinctus* can be recognized by the presence of abdominal constrictions and different appearance of abdominal sternite IX (subgenital plate). In *Zasphinctus*, the sternite is abruptly constricted proximal to where spines arise and is much wider at midlength. In *Lividopone* and *Parasyscia* in contrast, the sternite IX is usually gradually narrowing to the point of bifurcation. The males of *Eusphinctus* and *Sphinctomyrmex* have similar abdominal constrictions but the former has 12-segmented antennae and the latter has different wing venation with costal vein present.

#### Description.

***Worker.*** *Head*: Antennae with 11 or 12 segments. Apical antennal segment conspicuously enlarged, much broader than and longer than two preceding segments combined. Clypeus with cuticular apron. Lateroclypeal teeth absent or present. Parafrontal ridges absent or reduced. Torulo-posttorular complex vertical. Antennal scrobes absent. Labrum with median notch or concavity. Proximal face of stipes not projecting beyond inner margin of sclerite, prementum exposed when mouthparts fully closed. Maxillary palps 3-segmented. Labial palps 3-segmented. Mandibles triangular, with teeth or edentate. Eyes absent or present, composed of more than 20 ommatidia. Ocelli absent. Head capsule with differentiated vertical posterior surface above occipital foramen. Ventrolateral margins of head without lamella or ridge extending towards mandibles and beyond carina surrounding occipital foramen. Posterior head corners dorsolaterally immarginate. Carina surrounding occipital foramen ventrally present. *Mesosoma*: Pronotal flange separated from collar by distinct ridge or not. Promesonotal connection with Pronotomesopleural suture completely fused. Pronotomesopleural suture completely fused. Mesometapleural groove not impressed or weakly impressed. Transverse groove dividing mesopleuron absent or present. Pleural endophragmal pit concavity present. Mesosoma dorsolaterally immarginate. Metanotal depression or groove on mesosoma absent. Propodeal spiracle situated low on sclerite. Propodeal declivity with distinct dorsal edge or margin and rectangular in posterior view. Metapleural gland without bulla visible through cuticle. Propodeal lobes present, well developed. *Metasoma*: Petiole anterodorsally marginate or immarginate, dorsolaterally immarginate, and laterally above spiracle marginate or rarely immarginate. Helcium in relation to tergosternal Pronotomesopleural suture placed at posttergite and helcium axial, occasionally slightly supraaxial. Prora simple, not delimited by carina. Prora forming a U-shaped margin with median ridge. Spiracle openings of abdominal segments IV–VI circular. Abdominal segment III anterodorsally immarginate and dorsolaterally immarginate. Abdominal segment III more than half size of succeeding segment IV, which is weakly constricted at presegmental portion (uninodal waist). Girdling constriction of segment IV present, i.e. pre- and postsclerites distinct. Cinctus of abdominal segment IV not impressed. Abdominal segment IV not conspicuously largest segment. Abdominal tergite IV not folding over sternite, and anterior portions of sternite and tergite equally well visible in lateral view. Girdling constriction between pre- and posttergites of abdominal segments V and VI present. Girdling constriction between pre- and poststernites of abdominal segments V and VI present. Pygidium large, with impressed medial field, and armed with modified setae, sometimes notched. Hypopygium armed with modified setae. *Legs*: Mid tibia with single pectinate spur. Hind tibia with single pectinate spur. Hind basitarsus not widening distally, circular in cross-section. Posterior flange of hind coxa not produced as raised lamella. Metatibial gland absent or an oval patch of whitish cuticle. Metabasitarsal gland absent. Hind pretarsal claws simple. *Polymorphism*: Monomorphic.

***Male.*** *Head*: Antennae with 12 or 13 segments. Clypeus with cuticular apron. Parafrontal ridges absent. Torulo-posttorular complex vertical. Maxillary palps 3-segmented. Labial palps 3-segmented. Mandibles triangular, edentate to falcate. Ventrolateral margins of head without lamella or ridge extending towards mandibles and beyond carina surrounding occipital foramen. Carina surrounding occipital foramen ventrally present. *Mesosoma*: Pronotal flange separated from collar by distinct ridge. Notauli present or, more rarely, absent. Transverse groove dividing mesopleuron present. Propodeal declivity with distinct dorsal edge or margin. Metapleural gland opening absent. Propodeal lobes present. *Metasoma*: Petiole anterodorsally marginate, dorsolaterally immarginate, and laterally above spiracle marginate. Helcium in relation to tergosternal Pronotomesopleural suture placed at Pronotomesopleural suture and axial. Prora forming a V-shaped protrusion. Spiracle openings of abdominal segments IV–VI circular. Abdominal segment III more than half size of succeeding segment IV; latter weakly constricted at presegmental portion (uninodal waist). Girdling constriction of segment IV present, i.e. pre- and postsclerites distinct. Cinctus of abdominal segment IV gutter-like, not sculptured. Girdling constriction between pre- and postsclerites of abdominal segments V and VI present. Abdominal segment IV not conspicuously largest segment. Abdominal sternite VII simple. Abdominal sternite IX distally armed with two spines curved dorsally at apices, with lateral apodemes shorter than or about as long as medial apodeme, directed anteriorly (towards head); all apodemes long. *Genitalia*: Cupula long relative to rest of genital capsule and shorter ventrally than dorsally. Basimere broadly fused to telomere, with sulcus discernable at junction, and ventrally with left and right arms abutting. Telomere gradually tapering toward apex. Volsella laterally flattened, at apex with dorsal lobe and hooked ventrally. Penisvalva laterally compressed, rounded at apex. *Legs*: Mid tibia with single pectinate spur. Hind tibia with single pectinate spur. Posterior flange of hind coxa not produced as raised lamella. Metatibial gland present as oval patch of whitish cuticle. Metabasitarsal glands absent. Hind pretarsal claws simple. *Wings*: Tegula present, broad, demiovate in shape. Vein C in fore wing absent. Pterostigma broad. Abscissa R·f3 absent. Abscissae Rs·f2–3 present, connecting with Rs+M&M·f2 or disconnected from Rs+M. Cross-vein 2r-rs present, differentiated from Rs·f4 by presence of Rs·f2–3. Abscissae Rs·f4–5 present, fused in absence of 2rs-m. Abscissa M·f2 in fore wing present, separated from Rs+M by Rs·f2. Abscissa M·f4 in fore wing present, not reaching wing margin. Cross-vein 1m-cu in fore wing present. Cross-vein cu-a in fore wing present, arising from M+Cu and proximal to M·f1 or near M·f1. Vein Cu in fore wing present, with only Cu1 branch prominent. Vein A in fore wing with abscissae A·f1 and A·f2 present. Vein C in hind wing absent. Vein R in hind wing absent. Vein Sc+R in hind wing absent. Abscissa Rs·f1 in hind wing not differentiated in absence of Sc+R. Abscissa Rs·f2 in hind wing present, not reaching wing margin. Cross-vein 1rs-m in hind wing fused with M·f1. Vein M+Cu in hind wing present. Abscissa M·f1 in hind wing present. Abscissa M·f2 in hind wing absent. Cross-vein cu-a in hind wing present. Vein Cu in hind wing present. Vein A in hind wing with abscissae A·f1 and A·f2 present; The latter a stub.

***Gyne.*** Alate, ergatoid, or subdichthadiigyne. Alate gynes are known in an undescribed species from Africa (Brown 1975) and in *Zasphinctus occidentalis* (Clark 1924a). In *Zasphinctus asper*, *Zasphinctus duchaussoyi*, and *Zasphinctus steinheili* known gyne specimens are wingless ergatoids that possess eyes and ocelli. The gyne of *Zasphinctus imbecilis* can be considered a ‘subdichthadiigyne’; it possesses only vestigial eyes and one or no ocelli in addition to enlarged gaster. Descriptions and extensive discussions of gyne morphology in *Zasphinctus* can be found in Brown (1975), Clark (1924a), and Wheeler (1918).

***Larva.*** Cocoons present.

#### Distribution.

The twenty described species of *Zasphinctus* are distributed throughout Australasia, including New Caledonia and New Guinea, and the Afrotropics. Most species are known from Australia, with only three taxa described from Africa. Recently a species has been described from Thailand (Jaitrong et al. 2016), and unidentified *Zasphinctus* males are also known from Myanmar (author’s unpublished observations).

#### Taxonomy and phylogeny.

This name is here revived from synonymy with *Sphinctomyrmex*. For a brief account of taxonomic history and justification see under *Eusphinctus*.

Brown (1975) gave a preliminary key to Indomalayan and Australasian species.

The position of *Zasphinctus* within dorylines appears to be well established as the sister group to *Parasyscia*, and it is reasonably certain that this lineage was derived independently from the Neotropical *Sphinctomyrmex* (Figure 1; Brady et al. 2014, Borowiec, in prep.).

#### Biology.

Wilson provided notes on the biology of *Zasphinctus caledonicus* from New Caledonia and *Zasphinctus steinheili* from Australia. The former was observed raiding a nest of *Stigmacros* ants in the field, and the latter was feeding on ant brood of several species in the laboratory. Both were reported to have colonies containing multiple ergatoid gynes and synchronized brood. Buschinger et al. (1990) studied a species related to *Zasphinctus steinheili* in more detail under laboratory conditions. They largely confirmed Wilson’s preliminary observations and further demonstrated functional polygyny, since most dissected queens were fertilized with well-developed ovaries. The ants would indeed take brood of several ant species, including European forms, but the colonies ceased producing new eggs after two brood cycles were completed and thereafter slowly declined. Briese (1984) described a nest evacuation response in a *Monomorium* species raided by *Zasphinctus* in Australia. Hölldobler et al. (1996) described the metatibial gland of *Zasphinctus steinheili*.

#### Species of *Zasphinctus*

*Zasphinctus asper* (Brown, 1975): Australia, **comb. n.**

*Zasphinctus caledonicus* (Wilson, 1957): New Caledonia, **comb. n.**

*Zasphinctus cedaris* (Forel, 1915): Australia, **comb. n.**

*Zasphinctus chariensis* (Santschi, 1915): Chad, **comb. n.**

*Zasphinctus clarus* (Forel, 1893b): Australia, **comb. n.**

*Zasphinctus cribratus* (Emery, 1897): Papua New Guinea, **comb. n.**

*Zasphinctus duchaussoyi* (André, 1905): Australia, **comb. n.**

*Zasphinctus emeryi* (Forel, 1893b): Australia, **comb. n.**

*Zasphinctus froggatti* (Forel, 1900a): Australia, **comb. n.**

*Zasphinctus imbecilis* (Forel, 1907c): Australia, **comb. n.**

*Zasphinctus mjobergi* (Forel, 1915): Australia, **comb. n.**

*Zasphinctus myops* (Forel, 1895b): Australia, **comb. n.**

*Zasphinctus nigricans* (Clark, 1926): Australia, **comb. n.**

*Zasphinctus occidentalis* (Clark, 1924a): Australia, **comb. n.**

*Zasphinctus rufiventris* (Santschi, 1915): Benin, **comb. n.**

*Zasphinctus septentrionalis* (Crawley, 1925): Australia, **comb. n.**

*Zasphinctus siamensis* (Jaitrong, 2016): Thailand, **comb. n.**

*Zasphinctus steinheili* (Forel, 1900a): Australia, **comb. n.**

*Zasphinctus trux* (Brown, 1975): Australia, **comb. n.**

*Zasphinctus turneri* (Forel, 1900a): Australia, **comb. n.**

## Supplementary Material

XML Treatment for Acanthostichus

XML Treatment for Aenictogiton

XML Treatment for Aenictus

XML Treatment for Cerapachys

XML Treatment for Cheliomyrmex

XML Treatment for Chrysapace

XML Treatment for Cylindromyrmex

XML Treatment for Dorylus

XML Treatment for Eburopone

XML Treatment for Eciton

XML Treatment for Eusphinctus

XML Treatment for Labidus

XML Treatment for Leptanilloides

XML Treatment for Lioponera

XML Treatment for Lividopone

XML Treatment for Neivamyrmex

XML Treatment for Neocerapachys

XML Treatment for Nomamyrmex

XML Treatment for Ooceraea

XML Treatment for Parasyscia

XML Treatment for Procerapachys

XML Treatment for Simopone

XML Treatment for Sphinctomyrmex

XML Treatment for Syscia

XML Treatment for Tanipone

XML Treatment for Vicinopone

XML Treatment for Yunodorylus

XML Treatment for Zasphinctus

## Appendix

**Table 1.** Anatomical terms used here as annotated by Hymenoptera Anatomy Ontology (HAO). The first column provides the name of the term, the second is the HAO definition, and the third column provides a Uniform Resource Identifier (URI) that is a link to concept definition.

Term	Definition	URI
abdomen	The tagma that is located posterior to the thorax.	http://purl.obolibrary.org/obo/HAO_0000015
abdominal segment	The segment that is located posterior to the head and does not bear legs.	http://purl.obolibrary.org/obo/HAO_0000016
abscissa	The wing vein that is delimited by its intersection with two other abscissae.	http://purl.obolibrary.org/obo/HAO_0000076
anal vein	The wing vein that articulates proximally with the third axillary sclerite and is located just posteriorly of the claval fold.	http://purl.obolibrary.org/obo/HAO_0000093
annulus	The sclerite that is ring-like and is not attached to muscles.	http://purl.obolibrary.org/obo/HAO_0000096
antenna	The appendage that is composed of ringlike sclerites and the anatomical structures encircled by these sclerites and that is articulated with the cranium.	http://purl.obolibrary.org/obo/HAO_0000101
antennal scrobe	The scrobe that is located dorsally of the antennal foramen and is for the reception of the antenna.	http://purl.obolibrary.org/obo/HAO_0001432
antennal segment	The antennomere that is an appendage segment.	http://purl.obolibrary.org/obo/HAO_0000104
apodeme	The process that is internal.	http://purl.obolibrary.org/obo/HAO_0000142
basitarsus	The tarsomere that is the proximal most annulus of the tarsus, connected proximally with the tibia and distally with the second tarsomere via membranous conjunctivae.	http://purl.obolibrary.org/obo/HAO_0000178
body	The anatomical cluster that is composed of the whole organism but which excludes the antennae, legs and wings.	http://purl.obolibrary.org/obo/HAO_0000182
carina	The process that is elongate and external.	http://purl.obolibrary.org/obo/HAO_0000188
cell	The wing membrane that is delimited by wing veins.	http://purl.obolibrary.org/obo/HAO_0001091
cercus	The sense organ that located apicolaterally on one of the apicalmost abdominal terga and attaches to a large nerve cord.	http://purl.obolibrary.org/obo/HAO_0000191
clypeus	The area that corresponds to the site of origin of the clypeo-epipharyngeal muscle.	http://purl.obolibrary.org/obo/HAO_0000212
costa	The wing vein that is anterior to the subcosta and is connected to the humeral plate.	http://purl.obolibrary.org/obo/HAO_0000225
coxa	The leg segment that is connected to the body and to the trochanter via conjunctivae and muscles.	http://purl.obolibrary.org/obo/HAO_0000228
crest	The projection that is lamella-like and is located on a rim, carina, apodeme or edge.	http://purl.obolibrary.org/obo/HAO_0000344
cubital vein	The longitudinal vein that is posterior to the marginal vein.	http://purl.obolibrary.org/obo/HAO_0000237
cupula	The sclerite that is connected via conjunctiva and attached via muscles to abdominal tergum 9 and the gonostyle/volsella complex.	http://purl.obolibrary.org/obo/HAO_0000238
cuticle	The acellular anatomical structure that is the external layer of the integument (covers the entire body surface as well as lines ectodermal invaginations such as the stomodeum, proctodeum and tracheae) and produced by the epidermal cells.	http://purl.obolibrary.org/obo/HAO_0000240
depression	The area that is external, concave, point-like and does not correspond to an apodeme.	http://purl.obolibrary.org/obo/HAO_0000241
edge	The margin that extends along the border of two areas that are oriented differently.	http://purl.obolibrary.org/obo/HAO_0000285
eye	The compound organ that is composed of ommatidia.	http://purl.obolibrary.org/obo/HAO_0000217
flange	The projection that is lamella-like and is located on a rim, carina, apodeme or edge.	http://purl.obolibrary.org/obo/HAO_0000344
foramen	The anatomical space that is surrounded by sclerites and allows for the passage of haemolymph, nerves and tracheae.	http://purl.obolibrary.org/obo/HAO_0000345
fore wing	The wing that is located on the mesothorax.	http://purl.obolibrary.org/obo/HAO_0000351
gaster	The anatomical cluster that is composed of all abdominal segments posterior to abdominal segment 2. [note: in ants, this definition is applied to all segments posterior to segment 2 or to all segments posterior to segment 3 if the latter is small and differentiated into a postpetiole]	http://purl.obolibrary.org/obo/HAO_0000369
gena	The area that is delimited by the intersection of the interorbital plane, the margin of the compound eye, the margin of the oral foramen, the occipital carina and the malar sulcus.	http://purl.obolibrary.org/obo/HAO_0000371
genitalia	The anatomical system that is involved in copulation, fertilization and/or oviposition.	http://purl.obolibrary.org/obo/HAO_0000374
groove	The line that is located on the sclerite and is impressed.	http://purl.obolibrary.org/obo/HAO_0001525
head	The tagma that is located anterior to the thorax.	http://purl.obolibrary.org/obo/HAO_0000397
head capsule	The anatomical cluster that is composed of the cranium and compound eye cuticle.	http://purl.obolibrary.org/obo/HAO_0000398
hind wing	The wing that is located on the metathorax.	http://purl.obolibrary.org/obo/HAO_0000400
hypopygium	The abdominal sternum that is located on abdominal segment 7.	http://purl.obolibrary.org/obo/HAO_0000044
jugal lobe	The wing region that is delimited by the plica jugalis.	http://purl.obolibrary.org/obo/HAO_0000445
labial palp	The anatomical structure that is distal to the proximal margin of the first sclerite of the labial palp.	http://purl.obolibrary.org/obo/HAO_0000450
labium	The appendage that is encircled by the area that is proximally delimited by the lateral margins of the cardo and the posterior stipital sclerite laterally, and the anatomical line that is tangential to the salivary duct and traverses the salivary orifice anteriorly.	http://purl.obolibrary.org/obo/HAO_0000453
labrum	The sclerite that is situated along the distal margin of the clypeus and is connected along its proximal margin with the distal margin of the epipharyngeal wall.	http://purl.obolibrary.org/obo/HAO_0000456
lamella	The process that is elongate and external.	http://purl.obolibrary.org/obo/HAO_0000188
leg	The appendage that is composed of the coxa and all distal leg segments and is connected to the pectus.	http://purl.obolibrary.org/obo/HAO_0000494
line	The anatomical structure that is linear.	http://purl.obolibrary.org/obo/HAO_0001586
lobe	The evagination that is mostly membranous.	http://purl.obolibrary.org/obo/HAO_0001587
longitudinal vein	The anatomical cluster that is composed of abscissae extending along the long axis of the wing.	http://purl.obolibrary.org/obo/HAO_0000501
mandible	The appendage that is encircled by one sclerite that is connected to the cranium proximolaterally and to the maxillo-labial complex proximomedially via conjunctivae and articulates with the cranium via the anterior and posterior cranio-mandibular articulations.	http://purl.obolibrary.org/obo/HAO_0000506
maxilla	The appendage that is encircled by the area that is proximally delimited by the hypostoma posteriorly, the median margin of the mandible laterally, the labrum anterolaterally and the labium medially.	http://purl.obolibrary.org/obo/HAO_0000513
maxillary palp	The anatomical structure that is distal to the proximal margin of the first sclerite of the maxillary palp.	http://purl.obolibrary.org/obo/HAO_0000515
mesonotum	The area that is limited anteriorly by the pronotum, laterally by the basalare, axillary sclerites, subalare and the mesopectus and posterolaterally by the mesopostnotum and the metanotum.	http://purl.obolibrary.org/obo/HAO_0000556
mesopleuron	The lateral (vertical) area that is anterior to the mesometapleural sulcus and posterior to the pronotum.	http://purl.obolibrary.org/obo/HAO_0002363
mesosoma	The anatomical cluster that is composed of the prothorax, mesothorax and the metapectal-propodeal complex.	http://purl.obolibrary.org/obo/HAO_0000576
metafurca	The furca that is located medioventrally on the metapectus.	http://purl.obolibrary.org/obo/HAO_0000592
metanotal depression	The transverse, dorsal groove on the mesonoto-metanoto-mesopecto-metapecto-propodeal complex posterior to the pronotal spiracle and anterior to the propodeal spiracle.	http://purl.obolibrary.org/obo/HAO_0002362
metapleuron	The lateral (vertical) area that is posterior to the mesometapleural sulcus and anterior to the metapleural carina.	http://purl.obolibrary.org/obo/HAO_0002360
metasoma	The tagma that is connected anteriorly to the metapectal-propodeal complex at the propodeal foramen and consists of abdominal segments.	http://purl.obolibrary.org/obo/HAO_0000626
metathorax	The thoracic segment that is located between the mesothorax and the first abdominal tergum and is delimited by the metanotum and the metapectus.	http://purl.obolibrary.org/obo/HAO_0000630
mouthparts	The anatomical cluster that is composed of the labrum, epipharyngeal wall, hypopharyngeal wall (including the sitophore), mandibles, maxillae, labium and conjunctivae connecting them.	http://purl.obolibrary.org/obo/HAO_0000639
notch	The part of the margin of a sclerite that is concave.	http://purl.obolibrary.org/obo/HAO_0000648
occipital foramen	The foramen that is delimited dorsally by the postocciput.	http://purl.obolibrary.org/obo/HAO_0000347
ocellus	The multi-tissue structure that is located on the top of the head, composed of the corneal lens, pigment cell, rhabdoms and synaptic plexus.	http://purl.obolibrary.org/obo/HAO_0000661
ommatidium	One of the facets of the compound eye.	http://purl.obolibrary.org/obo/HAO_0000666
palp	The anatomical cluster that is composed of the palpal segments.	http://purl.obolibrary.org/obo/HAO_0000683
patch	The area that is round and differs from surrounding regions in sculpture, setae, and/or pigmentation.	http://purl.obolibrary.org/obo/HAO_0000704
penisvalva	The sclerite that is in the middle of the external male genitalia, surrounds the distal part of the ductus ejaculatorius and the endophallus.	http://purl.obolibrary.org/obo/HAO_0000707
posttergite	The area that corresponds to the narrow postcostal lip of a postnotal thoracic plate.	http://purl.obolibrary.org/obo/HAO_0000797
prementum	The sclerite that is median, is connected via conjunctiva along its proximolateral margins to the stipites, is articulated with the labial palps, is continuous along its distal margin with the ligula and distolateral margins with the distal hypopharynx and receives the site of attachments of the extrinsic labial palp muscles.	http://purl.obolibrary.org/obo/HAO_0000804
process	The area on the sclerite that is raised.	http://purl.obolibrary.org/obo/HAO_0000822
pronotum	The notum that is located in the prothorax.	http://purl.obolibrary.org/obo/HAO_0000853
propodeal spiracle	The abdominal spiracle that is the anteriormost in the body.	http://purl.obolibrary.org/obo/HAO_0000329
propodeum	The tergum that is located on abdominal segment 1.	http://purl.obolibrary.org/obo/HAO_0000051
prora	The carina that is transverse and is located anterior to the antecostal sulcus on the abdominal sternum 3.	http://purl.obolibrary.org/obo/HAO_0001341
pterostigma	The patch on the wing that is sclerotized and is on the anterior margin of the fore wing.	http://purl.obolibrary.org/obo/HAO_0000957
pulvillum	The area that is located laterally on the pretarsus and arises beneath the proximal part of the tarsal claw.	http://purl.obolibrary.org/obo/HAO_0000884
ridge	The process that is elongate and external.	http://purl.obolibrary.org/obo/HAO_0000188
row	The anatomical cluster that is composed of repeated units of anatomical structures.	http://purl.obolibrary.org/obo/HAO_0000901
scape	The antennal segment that is proximal to the pedicel and is connected to the head via the radicle.	http://purl.obolibrary.org/obo/HAO_0000908
sclerite	The area of the cuticle that is strongly sclerotised, with thick exocuticle and is surrounded by conjunctivae.	http://purl.obolibrary.org/obo/HAO_0000909
scrobe	The area that is impressed and is for the reception or concealment of another sclerite.	http://purl.obolibrary.org/obo/HAO_0000912
segment	An anatomical structure that is metameric and is connected to other metameric subdivisions by muscles and is delimited by its sclerites.	http://purl.obolibrary.org/obo/HAO_0000929
spiracle	The anatomical cluster that is composed of the distal end of the trachea and the margin of the sclerite or conjunctiva surrounding the spiracular opening.	http://purl.obolibrary.org/obo/HAO_0000950
spur	The process that is surrounded by conjunctiva and evaginated and that is basally sclerotized.	http://purl.obolibrary.org/obo/HAO_0000951
sternite	The sclerite that is located on the sternum.	http://purl.obolibrary.org/obo/HAO_0000955
stipes	The appendage that is connected posteroproximally to the hypostoma, anteroproximally and lateroproximally to the mandible and medioproximally to the labium and the hypopharynx via conjunctiva, is connected to the cranium via muscles and that bears the maxillary palp.	http://purl.obolibrary.org/obo/HAO_0000958
tarsus	The leg segment that is apical to the tibia.	http://purl.obolibrary.org/obo/HAO_0000992
tegula	The sclerite that is located laterally of the preaxilla and obscures the anterior mesonoto-first axillary articulation and the mesopleuro-second axillary sclerite joints.	http://purl.obolibrary.org/obo/HAO_0000993
tergite	The sclerite that is located on the tergum.	http://purl.obolibrary.org/obo/HAO_0001005
tibia	The leg segment that is proximal to the tarsus and distal to the femur.	http://purl.obolibrary.org/obo/HAO_0001017
tooth	The projection that is located distally on the mandible.	http://purl.obolibrary.org/obo/HAO_0001019
venation	The anatomical cluster that is composed of abscissae.	http://purl.obolibrary.org/obo/HAO_0001096
volsella	The anatomical cluster that is composed of the sclerites on the ventral part of the male genitalia that are not connected to the cupula via muscles.	http://purl.obolibrary.org/obo/HAO_0001084
wing	The appendage that is between the notum and the pectus and is connected to the body by the axillary sclerite muscles.	http://purl.obolibrary.org/obo/HAO_0001089
